# Click Chemistry for Biofunctional Polymers: From Observing to Steering Cell Behavior

**DOI:** 10.1021/acs.chemrev.4c00251

**Published:** 2024-12-02

**Authors:** Krishanu Ghosal, Swarup Krishna Bhattacharyya, Vivek Mishra, Han Zuilhof

**Affiliations:** † Research & Development Laboratory, Shalimar Paints Limited, Nashik, Maharashtra 422403, India; ‡ Materials Science Centre, Indian Institute of Technology Kharagpur, Kharagpur 721302, India; § Amity Institute of Click Chemistry Research and Studies, Amity University, Noida, Uttar Pradesh 201313, India; ∥ Laboratory of Organic Chemistry, Wageningen University, Stippeneng 4, 6708 WE Wageningen, Netherlands; ⊥ College of Biological and Chemical Sciences, Jiaxing University, Jiaxing 314001, China

## Abstract

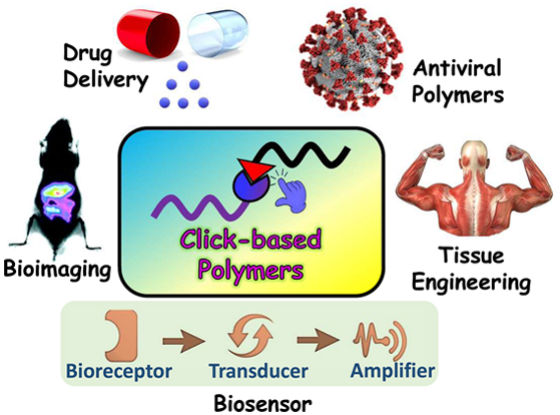

Click chemistry has become one of the most powerful construction tools in the field of organic chemistry, materials science, and polymer science, as it offers hassle-free platforms for the high-yielding synthesis of novel materials and easy functionalization strategies. The absence of harsh reaction conditions or complicated workup procedures allowed the rapid development of novel biofunctional polymeric materials, such as biopolymers, tailor-made polymer surfaces, stimulus-responsive polymers, etc. In this review, we discuss various types of click reactions—including azide–alkyne cycloadditions, nucleophilic and radical thiol click reactions, a range of cycloadditions (Diels–Alder, tetrazole, nitrile oxide, etc.), sulfur fluoride exchange (SuFEx) click reaction, and oxime-hydrazone click reactions—and their use for the formation and study of biofunctional polymers. Following that, we discuss state-of-the-art biological applications of “click”-biofunctionalized polymers, including both passive applications (e.g., biosensing and bioimaging) and “active” ones that aim to direct changes in biosystems, e.g., for drug delivery, antiviral action, and tissue engineering. In conclusion, we have outlined future directions and existing challenges of click-based polymers for medicinal chemistry and clinical applications.

## 1 Introduction

**Figure 1:**
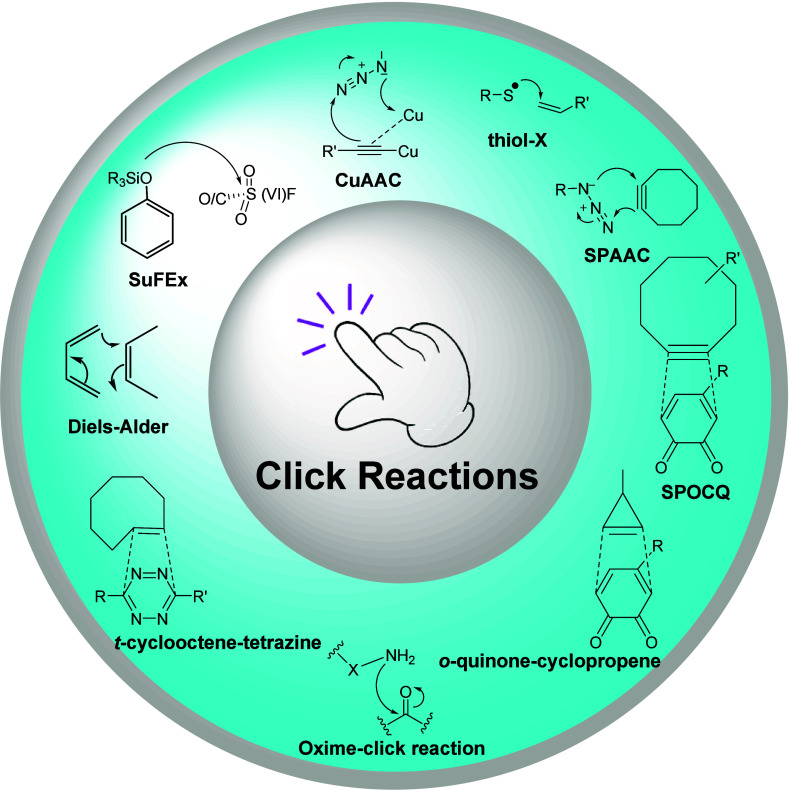
Schematic representation of various types of click reactions. CuAAC: copper-catalyzed azide–alkyne cycloaddition, SPAAC: Strain-promoted azide–alkyne cycloaddiotion, SPOCQ: Strain-promoted oxidation-controlled cyclooctyne–1,2-quinone cycloaddition, SuFEx: Sulfur fluoride exchange click reaction.

“Click chemistry” involves a series of modular, high-yielding, and smoothly proceeding reactions that are widely used in organic synthesis, medicinal chemistry, materials science, and biofunctionalization. The broad applications of this series of reactions rely upon its intrinsic selectivity and efficacy along with facile reaction setup, applicability in both aqueous as well as organic solvents, tolerance to a wide range of functional groups, respectable product yield, and minimal post synthesis workup.^1,2^ Since its introduction in 2001 great strides have been made in this field, and it continues to be a hot topic for researchers, as it offers unique platforms for the synthesis of, e.g., biomolecules and functional polymers. Click chemistry reactions include, by now, several large classes of reactions, including azide–alkyne cycloadditions,^3−5^ thiol click reactions,^6,7^ Diels–Alder cycloadditions,^8^ tetrazole cycloadditions,^9^ nitrile oxide cycloadditions,^10^ oxime/hydrazone reactions,^11,12^ tetrazine bioorthogonal reactions,^13,14^ and sulfur(VI) fluoride exchange (SuFEx) reactions^15^ (see Figure 1 for a schematic overview of several of the most prevalent click reactions).

These facile yet versatile reactions enable a wide range of syntheses as well as easy structural modification of polymer materials.^16−19^ Well-established examples include terminally functional polymers, pendent-functional polymers,^20^ star polymers,^21^ polymer brushs, graft polymers, hyperbranched polymers, dendrimers, hydrogels, as well as polymer-conjugated nanomaterials. Most of these polymeric materials, made for further click chemistry-based modifications, were originally synthesized by controlled polymerization techniques,^22^ i.e., atom transfer radical polymerization (ATRP),^22−29^ reversible addition–fragmentation chain transfer (RAFT) polymerization,^30−33^ and nitroxide-mediated polymerization (NMP).^34−36^ Controlled polymerization delivers several advantages, like control over molecular weight, a narrow polydispersity (*Đ* or PDI), a well-controlled architecture, and control over the chain-end functionality.^37,38^ These advantages rely upon the fast dynamic equilibrium between a dormant and propagating state in these reactions,^39−41^ and have been proven to be highly compatible with the presence of “clickable” functionalities. Many different post polymerization modification strategies have been developed to synthesize functional polymeric materials. These include catalyzed cross-coupling reactions,^42,43^ active ester^44^ and amide coupling reactions,^45^ and photochemical reactions.^43,46^ However, click chemistry has become one of the most efficient methods for the functionalization of polymers, due to its ease of synthesis, mild reaction conditions, high yield, simple workup, selectivity, and specificity.^47^

From an application point of view, click chemistry has several unique advantages, especially in the biomedical field, as it combines the enabling of architectural control over polymer synthesis with features like easy conjugation of small biomolecules to the polymers, post synthetic modifications, and a high yield of a single reaction product. Postpolymerization modification of polymers enables conjugation of functional groups linked to either any side chains or to the termini of polymers, which thus allows for fine-tuned multifunctional polymers. This strategy also permits the development of smart combinatorial materials specific to chemical, biological, or biomedical applications. For example, for many biomedical applications, the development of functional bioresorbable polymers is of high priority, as bioresorption facilitates both biocompatibility and biodegradability along with the role of such polymers as, e.g., cell support or drug delivery agent. These properties can be improved as well as fine-tuned through the incorporation of functional groups using post polymerization modification.^5,20,26,48−51^ Polymeric materials with improved biocompatibility allow achieving the desired function with minimal inflammation responses. Similarly, biodegradation plays a crucial role, as controlled degradation can provide interconnected space within the polymeric scaffold to permit cell proliferation, migration, or matrix remodeling to better mimic the extracellular matrix (ECM), and infiltration of blood vessels throughout the ECM. Click chemistry allows for the highly efficient and specific inclusion of the moieties that can achieve this, often in the presence of a wide variety of other functional groups. Furthermore, cell adhesion onto polymer substrates is greatly influenced by functional groups present on such substrates. For example, the functionalization of polymeric materials with cell-adhesive motifs like the arginine-glycine-aspartate (RGD) tripeptide moiety can facilitate specific surface interactions and a better cellular environment, in which cells experience minimal stress and can differentiate with their functional characteristics.^52−54^ In the case of drug and gene delivery, post polymerization modification of polymeric materials by click chemistries has become a major chemical strategy to develop biofunctional materials with smart capabilities, such as both active and passive targeting, stimuli-responsive behavior (i.e., pH, temperature), and sustained or burst release of drugs according to prerequisite conditions.^5,20,55,56^

There is, of course, broad recognition of the power of click chemistry, as exemplified by the Nobel Prize 2022 in Chemistry to Bertozzi, Meldal, and Sharpless.^57^ The above-mentioned unique advantages of click chemistry have led to numerous reviews that outline one or more of these click reactions, and since 2010, which focus on applications in the field of polymer chemistry. For example, a comprehensive overview of various click reactions, their roles in the synthesis of polymeric architectures, and their applications are provided by Golas et al.^1^ and Geng et al.^58^ However, no reviews have been published that focus on *biofunctional polymers* and their wide range of biomedical applications. This field is rapidly growing by enriching research areas that already exist for a while (e.g., drug delivery, tissue engineering) and by strongly driving the development of nearly fully novel fields (e.g., antiviral polymers). This growth of click chemistry-based biofunctional polymers is partially due to the increasing number of click reactions that can be used for this purpose, which allows the detailed tailoring of the syntheses to the specific situation at hand, and partially also due to the successes achieved by such polymers, as these show the potential for further investigations.

**Figure 2:**
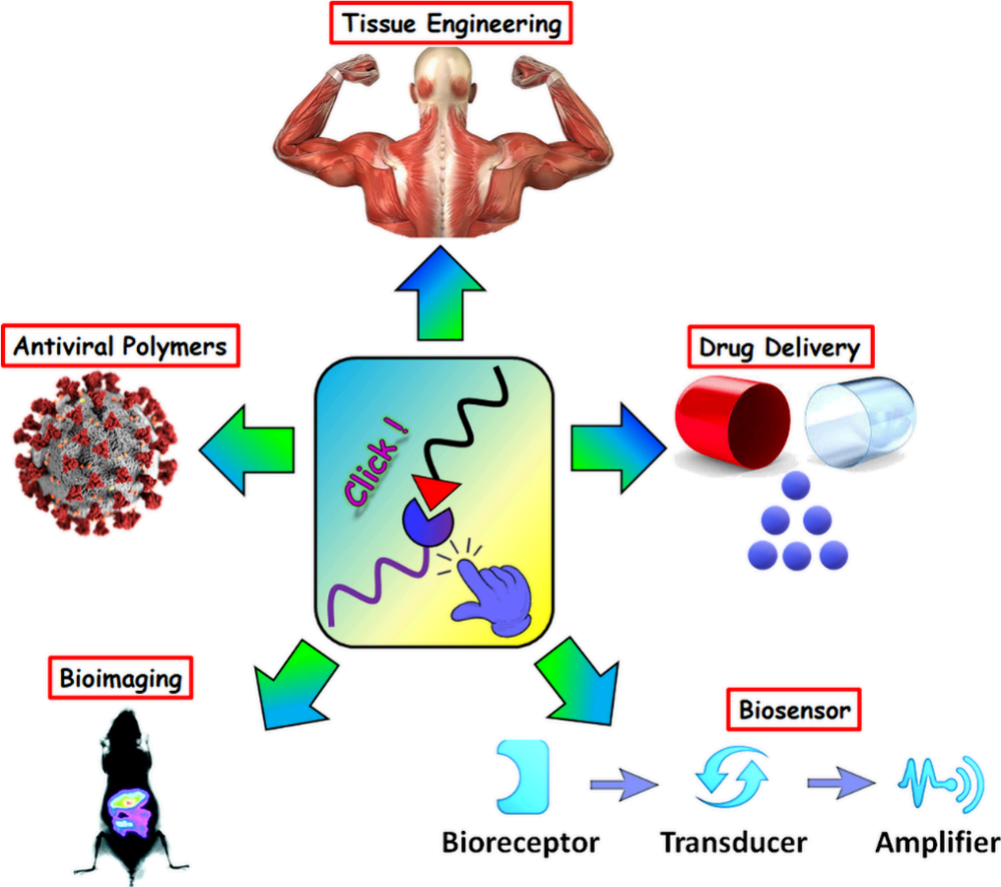
Schematic representation of various biomedical applications using functional polymers produced by click reactions.

In this review, we discuss the role of various click chemistry reactions in the synthesis of functional polymeric materials, and a range of “click-functionalized” biomedical applications of such materials, including drug delivery, antiviral action, biosensing, bioimaging, and tissue engineering (Figure 2), covering the literature up to the first quarter of 2024. We will also highlight the effects of the specific click chemistry that was used for those applications. Finally, we outline existing challenges and future perspectives of this field, with a personal tinge on where we think this field can best develop and some of the opportunities that will provide.

## 2 Different Strategies for the Synthesis of Functional Polymers by Click Chemistry

As discussed above, click reactions offer a versatile and powerful synthetic platform owing to their unique advantages over other traditional organic reactions. The three most popular approaches for incorporating clickable moieties into polymer backbones are 1) the use of clickable macroinitiators or transfer agents; 2) post-polymerization modification of end groups, which generally yields terminal functionality; and 3) premodification of monomers with a clickable moiety, followed by polymerization, which yields side-chain functionalization, or pendent functional polymers. Even though such side chains or pendent functionalizations sometimes affect the polymerization efficiency and reaction rate, still, they are central in one of the most prevalent strategies to obtain functional polymers. In the case of approach 2), in principle, the end groups obtained by either ATRP (a halogen) or RAFT (a dithiobenzoate) can also be reacted with a wide range of functional groups. However, to obtain a substantial amount of well-defined post polymerized product, it is required that the modification process proceeds with high efficacy, and this is unfortunately typically not easy to accomplish.

**Figure 3:**
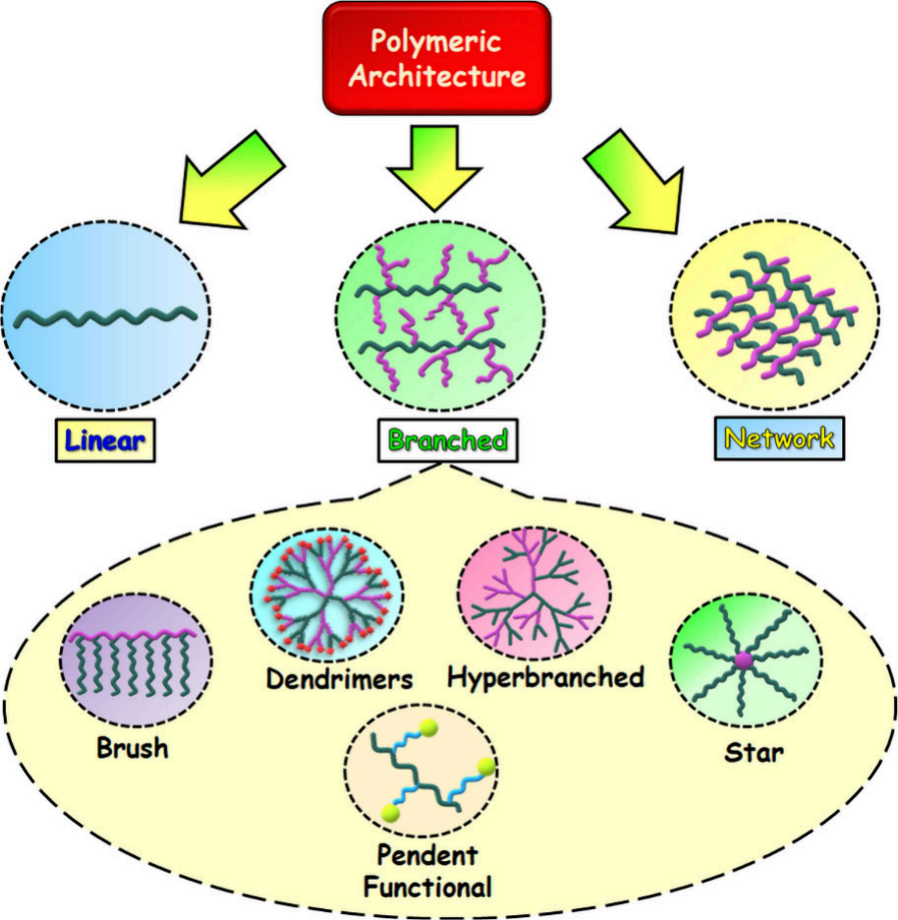
Classification of various polymers based on architectural complexity. Pink and green segments represent the fundamental polymeric building blocks, which are either formed or modified by click chemistry.

In this section of the review, the different polymers are classified according to their increasing order of architectural complexity, from linear polymers to branched polymers and highly functionalized polymer networks (Figure 3). The field has become too big to include even the majority of examples, so the focus in this section is on providing an overview with a focus on seminal and prototypical examples, so that—after working through this section 2—the reader is properly prepared for the applications that form the main focus of this review.

### 2.1 Linear Polymers

#### 2.1.1 Linear Polymers with Clicked Moieties in Backbone

**Figure 4:**
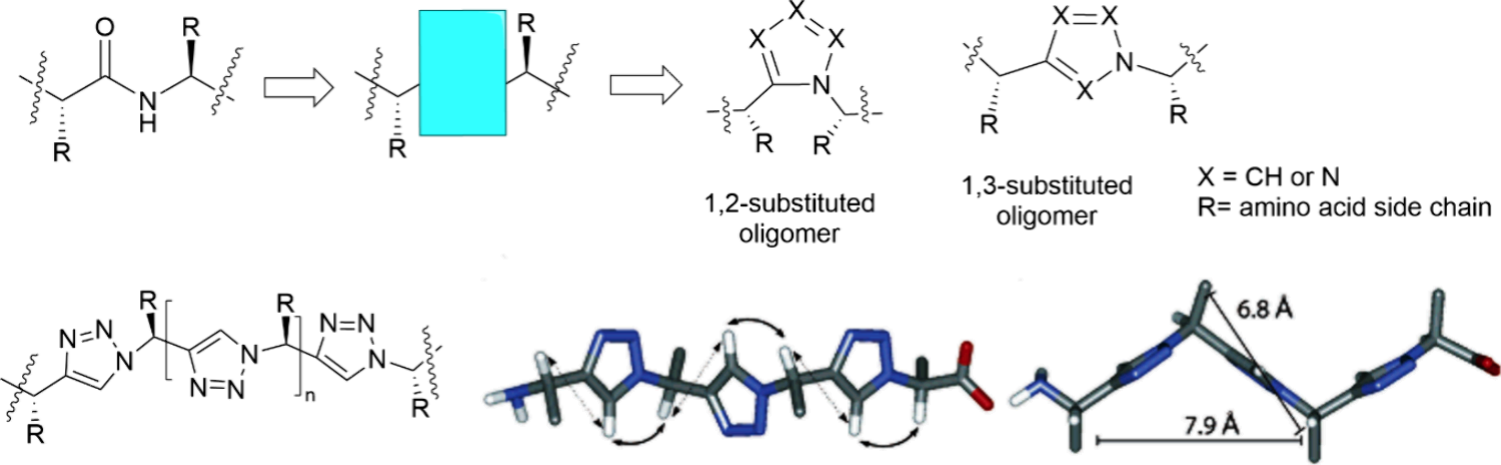
Linear polymers prepared by CuAAC connection of small molecule building blocks. Triazole units made by CuAAC events are highlighted in the blue box. Adapted with permission from ref (61). Copyright 2005, ACS.^61^

Since the progress of click chemistry, various different complicated polymeric architectures have been achieved, but it all started from the synthesis of linear polymers or oligomers. Fokin, Finn, and co-workers pioneered CuAAC-mediated linear polymer synthesis.^59,60^ In 2005, the first biorelevant example was published by Angelo and co-workers, with the development of peptidomimetic oligomers that possessed a “zigzag” secondary structure (Figure 4) using an iterative diazo-transfer/CuAAC process.^61^ In the resulting polymer the R groups would take the role of the traditional amino acid side chains, leading to a click-based analog of peptides.

**Figure 5:**
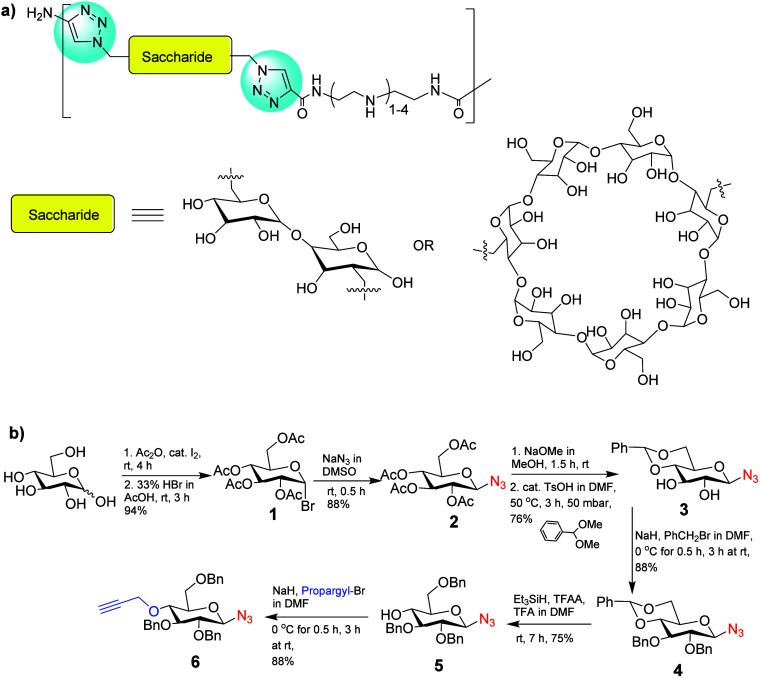
(a) Linear carbohydrate-oligoamine polymers prepared by CuAAC. Adopted with permission from ref (67). Copyright 2012 Springer.^67^ (b) Synthesis of the glucose-derived azido-alkyne monomer for CuAAC-clicked polyglucose. Adapted with permission from ref (68). Copyright 2022, Wiley.^68^

Since then, several other groups reported the preparation of a wide variety of peptide mimetics utilizing CuAAC.^62^ Reineke and his group contributed remarkably to the development of CuAAC-mediated linear polymer synthesis.^63−66^ They reported a series of copolymerizations in which trehalose or cyclodextrin molecules functionalized with two azide moieties were coupled to linear oligoamine monomers with two terminal alkyne units (see Figure 5a). The introduction of carbohydrates improved the biocompatibility, water solubility, and stability against aggregation of pre-DNA in serum for delivery.^67^

Meudtner and Hecht reported a triazole polymer with secondary structure and metal-binding pyridine units. A linear polymer with 2,6-bis(1,2,3-triazol-4-yl)pyridine units on the backbone was constructed by step-growth polymerization of 2,6-diethynylpyridines with a bifunctional aromatic azide.^69^ These linear helical polymers possess an *anti*-*anti* conformation, which was further confirmed with circular dichroism. They also observed that upon introduction of various metal salts (Zn^2+^, Eu^3+^, Fe^2+^) to the dilute solutions of these linear helical polymers, cross-linking occurred, and a supramolecular metallogel structure was formed. It was suggested that these polymers could be helpful as magnetic and/or emissive materials.

In 2007, Wang and co-workers constructed high-molecular weight linear PEG polymers with pendent functionalities by coupling alkyne-telechelic PEGs with 2,2-bis(azidomethyl)propane-1,3-diol via CuAAC.^70^ The biomineral-binding study of this novel PEG conjugate suggested strong osteotropicity *in vivo* and demonstrated its potential application in tissue-specific delivery of therapeutic agents to the skeleton. On a different note, unlike copper recently ruthenium catalyzed azide–alkyne cycloaddition (RuAAC) and iridium catalyzed azide–alkyne cycloaddition (IrAAC) reaction also exploited for synthesis of linear polytriazoles.^71−73^ However, the application of these reactions in biomedical field is still limited due to the toxicity associated with these heavy metals.

More recently, SuFEx has been effectively used for polymer formation, with an increasing biological potential. For example, Sharpless, Wu, and Dong introduced HF_2_^–^ catalysis for the formation of a wide range of polysulfates and polysulfonates.^74^ This strategy, provided significant potential for adaptation to biological functionalities, as SuFEx has been proven to be highly compatible with a wide range of functionalities. Subsequently, the groups of Wu, Zuilhof, Moses, and Sharpless developed multidimensional polymer click chemistry, which allowed both the formation of the polymers from monomers and the post polymerization modification to occur with high efficiency,^75^ while Zuilhof's group subsequently introduced the use of intrinsically chiral SuFEx reactions to get biomimetic polymers with backbone chirality^76^ and gently degradable SuFEx polymers.^77^ Michaudel and co-workers also exploited SuFEx click reactions to design linear degradable polysulfamides.^78^ Briefly, they synthesized AB type monomers by mono-Boc protection of aliphatic and aromatic diamines, and subsequently reacted the other amine with a storable “^+^SO_2_F” agent to get an −SO_2_F functionalized monomer. Removal of the Boc group then allowed efficient polymerization via the SuFEx click reaction. The versatility of SuFEx polymerizations enabled a wide variety of the main chain structure by starting from different aliphatic or aromatic diamines.

#### 2.1.2 Pendent-Functional Polymers

As discussed earlier, the incorporation of clickable pendent functionalities throughout the polymer chain is usually achieved by polymerizing a functional monomer. One of the pioneer examples of this method involves a synthesis of 3-azidopropyl methacrylate by Tsarevsky et al., followed by polymerization using ATRP.^79^ Additionally, they showed that the azido group present in the pendent position of each monomer provides easy access to post polymerization modifications using the CuAAC reaction with a range of alkynes in gram-scale amounts. Some studies also reported direct ATRP of commercially available propargyl methacrylate,^76,80^ but the results are less promising given the high *Đ* values and multimodal molecular weight distributions. This is likely due to the presence of the acetylene moiety, which affects the polymerization process.

To partially circumvent this, Haddleton and co-workers reported the synthesis of a polymer containing pendent alkyne groups using ATRP of a trimethylsilyl-protected alkyne methacrylate.^81^ After synthesis of the polymer, the −SiMe_3_ protecting groups were removed using tetrabutylammonium fluoride, which resulted in CuAAC-clickable alkyne-labeled polymers that were further conjugated with azide-modified sugar derivatives.

**Figure 6:**
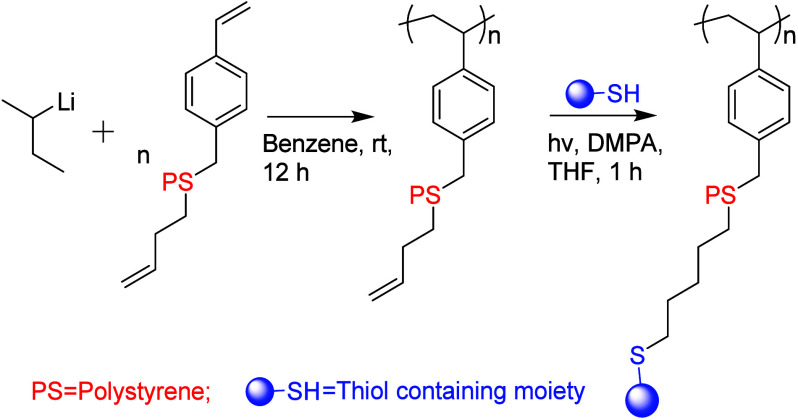
Synthesis of pendent-functionalized polymer using thiol–ene click chemistry (based on ref (84). Copyright 2012 ACS^84^).

Such pendent alkyne functionalities can also be obtained from other polymerization techniques. The first alternative to be investigated was ring-opening metathesis polymerization (ROMP). Here either the alkyne-functionalized poly(oxynorbornenes) can be synthesized first using ROMP, followed by azide–alkyne click chemistry, or the clicked monomer can be synthesized first by reacting alkyne-bearing oxynorbornene and azide, after which ROMP can take place. Studies revealed that both these strategies are equally applicable for the incorporation of hydrophilic and hydrophobic moieties within the polymer backbone.^82^ Analogously, nitroxide-mediated radical polymerization (NMP) was also employed, which could be further modified using diverse azide-bearing moieties with the help of click reactions.^83^ Third, the combination of living-anionic polymerization and thiol–ene reactions was used for pendent functionalization, e.g., via the preparation of an ene-functionalized macromonomer that can be polymerized as well as further functionalized with thiol-bearing moieties as shown in Figure 6.^84^ This method provides an efficient platform for the synthesis of comb polymers with precisely controlled architectural details and a wide selection of functionalities (ranging from, e.g., −OH and −CO_2_H, to −C_8_F_17_). On top of that, this strategy offers at least three key advantages for synthesis of comb polymers: control over the molecular weight with narrow weight distributions, highly efficient post polymerization functionalization, and easy modulation of functionalization levels. Furthermore, it enables the synthesis of more complex comb architectures, such as those with two distinct functionalities within the same chain, and allows precise control over the spacing between side branches. In a different approach, the thiol–ene reaction was also utilized to install pendent hydroxyl ethyl thioethers in dehydrogenated polyethylene and polycyclooctene.^85^ Polycyclooctene functionalization with mercaptoethanol resulted via thiol–ene click chemistry in 1.4 to 22.9% functionalization, based on the number of ethylene monomeric units.

In addition to CuAAC and thiol–ene click reactions, tetrazine bioorthogonal chemistry also offers an excellent platform for the synthesis of pendent functional polymers.^86^ This is attributed to its mild catalyst free reaction conditions, excellent selectivity, and broad compatibility with various functional groups. For example, Kahveci and co-workers reported synthesis of pendent functional polymers by reacting tetrazine functionalized poly(*n*-isopropylacrylamide-*co*-acrylonitrile) (poly(NIPAAm-*co*-AN)) and poly((ethylene glycol) methyl ether methacrylate-*co*-acrylonitrile) (poly(NIPAAm-*co*-AN)) with trans-cyclooctene functionalized groups.^87^ This novel approach may contribute synthesis of tetrazine functional polymers by the means of simplicity and effectiveness. More importantly, this simplicity and effectiveness make tetrazine biorthogonal chemistry an ideal method for manipulating functional polymers and nanomaterials in both *in vitro* and *in vivo* settings.^88^ For instance, Weissleder and co-workers demonstrated that fluorescent tetrazine-functionalized dextran can react with anti-CD45 monoclonal antibodies labeled with trans-cyclooctene under *in vivo* conditions, highlighting its potential for bioimaging applications.^89^

**Figure 7:**
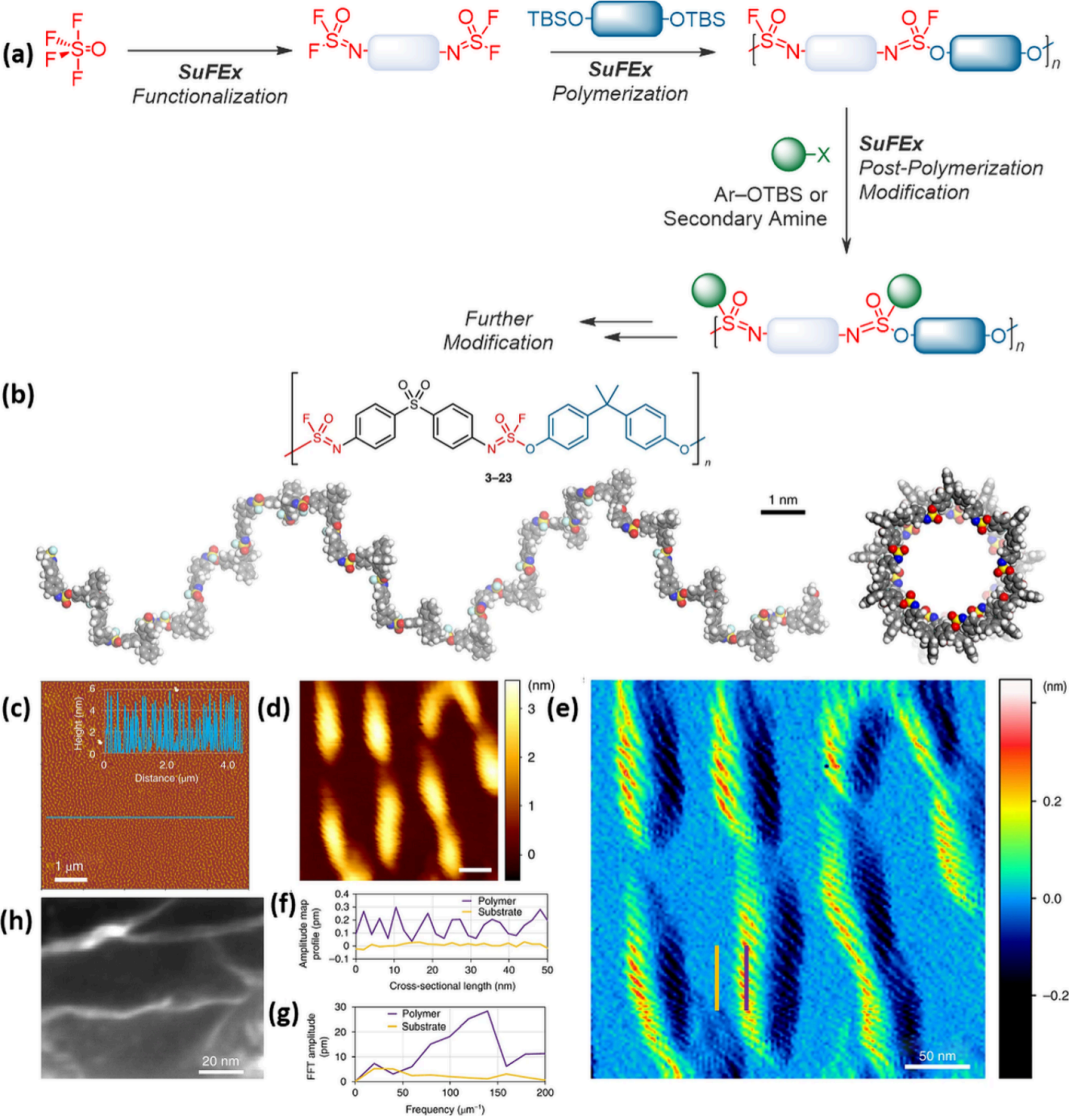
(a) Multidimensional SuFEx click chemistry to synthesize pendent functional polymers with helical structures as displayed in single polymer-chain studies. (b) Polymer consistent force field (PCFF) optimized structure of the synthesized polymers in side and top views. (c) AFM height image (5 × 5 μm), with inset topographic height profile, showing the structural organization and periodicity of the helical polymer. (d) Zoomed-in topographic image (scale bar 50 nm) and (e) amplitude map of polymer on hexadecyne-coated Si(111) surfaces. (f) Cross sections were taken over a polymer chain (vertical purple line in e) and over the substrate (parallel vertical yellow line in e), giving cross-sectional amplitude profiles acquired on the polymer (purple) and substrate (yellow) and fast Fourier transforms of the cross-sectional amplitude profiles on the polymer (purple) and substrate (yellow). (g) Dominant frequencies in the signal. (h) High-resolution SEM image of the synthesized polymer deposited on a TEM grid. Adapted with permission from ref (75). Copyright 2021, Nature.^75^

More recently, multidimensional SuFEx dimers [F_2_(=O)S=N ∼ R ∼ N=S(=O)F_2_] were created, in which one of the S–F bonds was used to efficiently form the polymer backbone (Figure 7a), while the second S–F bond was subsequently used to append a wide range of functionalities.^75^ The latter included for example nucleosides and redox-active moieties, displaying the exceptional versatility of this approach as also the backbone could be varied easily. This two-step approach worked, since the first SuFEx reaction is much faster than the second, yielding no observable network formation. The resulting polymers displayed an elegant helical structure (Figure 7b), as born out by single polymer-chain observations with high-resolution AFM (Figure 7c,d,e,f,g), and TEM as depicted in Figure 7h.

Key advantages of these polymers lie in their unique [−N=S(=O)F–O−] backbone linkages, which are highly stable, and inherently SuFExable so as to allow precise SuFEx-based postmodification with phenols or amines to yield branched functional polymers. Such post- modification strategy thus enables the creation of wide ranges of new polymers, with functionalities such as ferrocene, tetraphenyl ethene (endowing the resulting polymer with aggregation-induced emission characteristics) or thymidine, which hold significant potential for the development of polymer-drug conjugates.^75^ Additionally, studies on individual polymer chains of these new materials suggested the presence of helical structures brought about by the [−N=S(=O)F–O−] linkages in the backbone. The robustness of SuFEx click chemistry provides a powerful platform for post polymerization modifications, offering precise control over both the composition and conformation of the resulting materials.

### 2.2 Branched Polymers

The exploitation of click chemistry in polymer science offers vast opportunities to design and manipulate polymer chains into more complex architectures. The next section thus describes the use of click reactions to synthesize polymers with more complex architectures, such as dendrimers, bottlebrushes, graft and star polymers, as well as surface-bound polymer brushes.

#### 2.2.1 Dendrimers

Dendrimers are synthetic polymers with a structure of repeatedly branching chains, typically forming (near-)spherical macromolecules. Such macromolecules hold the promise of a large number of functionalities in a compact space, but suffer from time-consuming and complex purification cycles, and increased heterogeneity with increasing generations due to the noncomplete character of the coupling reactions. To guarantee quantitative conversion of the geometrically increasing number of functionalities, highly efficient reactions are essential in each iterative step. Since the early days, click reactions are considered ideal for dendrimer synthesis, given their potential for quantitative conversion.^90−95^ Inspired by their earlier “convergent-growth” mediated dendrimer synthesis,^92,96^ Hawker and co-workers obtained triazole-linked dendrimers using the CuAAC click reaction in 2004.^95^ Moreover, in 2005 they managed to efficiently and chemoselectively prepare multivalent asymmetrical bifunctional dendrimers as sensors for the inhibition of hemagglutination using a CuAAC reaction.^97^ The final structures of these dendrimers included 16 surface-active mannose units and two fluorescent coumarin units derived from two dendrons. These units were coupled via triazole linkages, using a CuAAC reaction, to the chain ends of the dendrimers. The resulting dendrimers were 240 times more potent that the monomeric mannose in a standard hemagglutination assay.

The combination of CuAAC with ATRP polymerization method to synthesize miktoarm stars was first demonstrated by Monterio et al.^99^ The authors prepared 3-miktoarmstar and dendrimers with miktoarm compositions consisting of polystyrene, poly(*tert*-butyl acrylate), poly(acrylic acid), and poly(methyl acrylate) using a combination of ATRP and click reactions. Azidotelechelic polystyrene (PS) was prepared from a bifunctional initiator. Each end of this polymer was capped with tripropargylamine using CuAAC to produce a tetra-alkyne-terminated PS. Subsequently, they reacted this alkyne-functionalized polymer with azido-terminated PAA, to obtain a branched structure with a hydrophobic PS core and a hydrophilic PAA shell.

**Figure 8:**
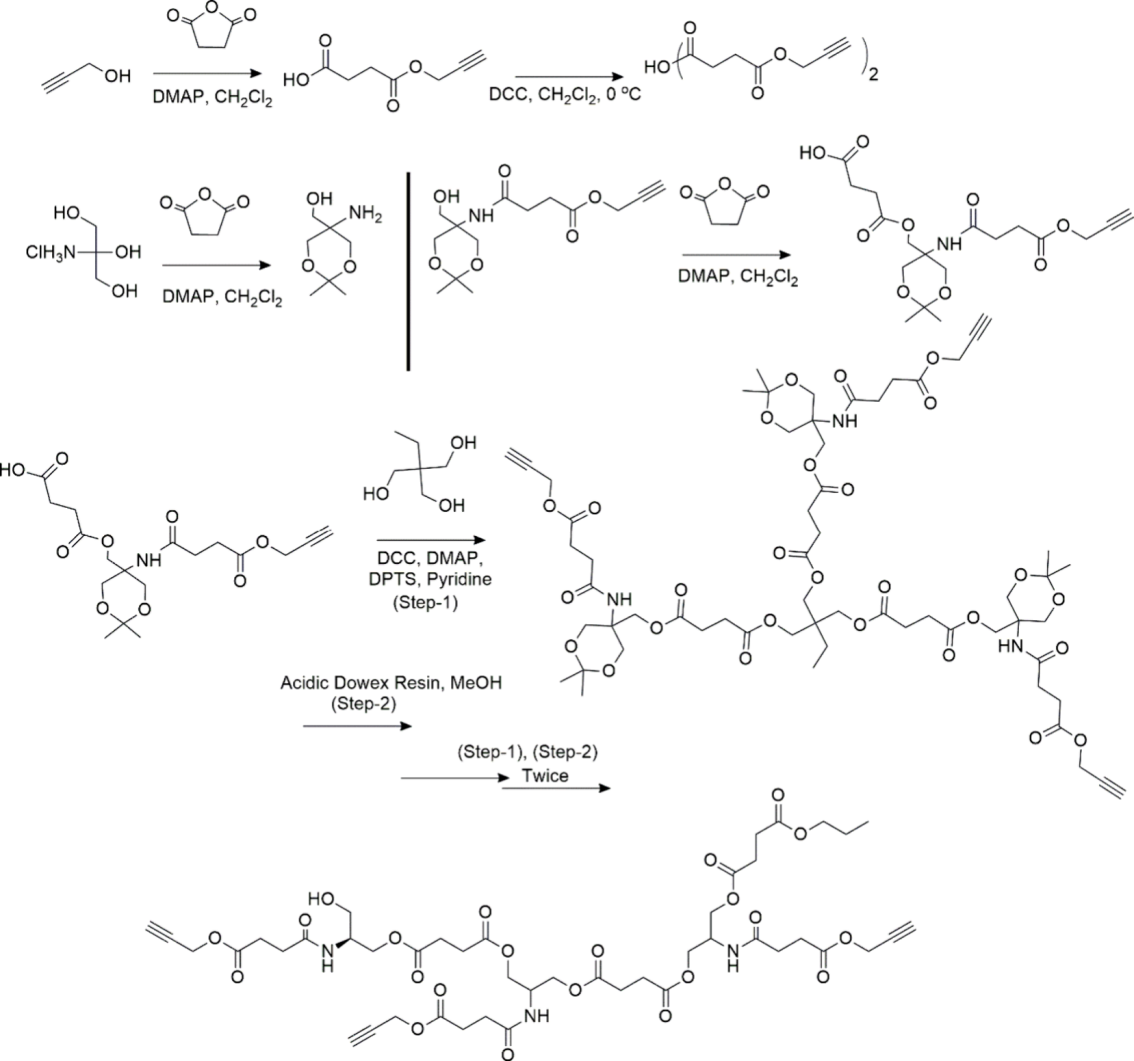
Representative examples of bifunctional dendrimers. Adapted with permission from ref (98). Copyright 2009, Wiley.^98^

Next, the relevance for biomaterials research was increased by the introduction of degradable ester-based branching units to yield G3 generation dendrimers with a hydrolytically cleavable periphery.^100^ Malkoch and co-workers also reported bifunctional dendrimers of G1–G3 generation with 24 hydroxyl groups at the periphery and 21 internal alkyne/azide groups. These groups were distributed all over the dendrimer’s backbone, and accessible for further functionalization by means of CuAAC (shown in Figure 8).^98^ Such bifunctional dendrimers can be used as drug delivery vehicles, where the dendron wedge having A-type functionality, allows the target to reach its destination, whereas the second dendron wedge having B type functionality can be used to conjugate multiple active drug compounds^101,102^ or fluorescent dyes for theranostics applications.^97^ Moreover, the versatility of these dendrimers can be harnessed for the fabrication of dendritic nanoparticles and hydrogels on the basis of dendritic cross-linkers.

**Figure 9:**
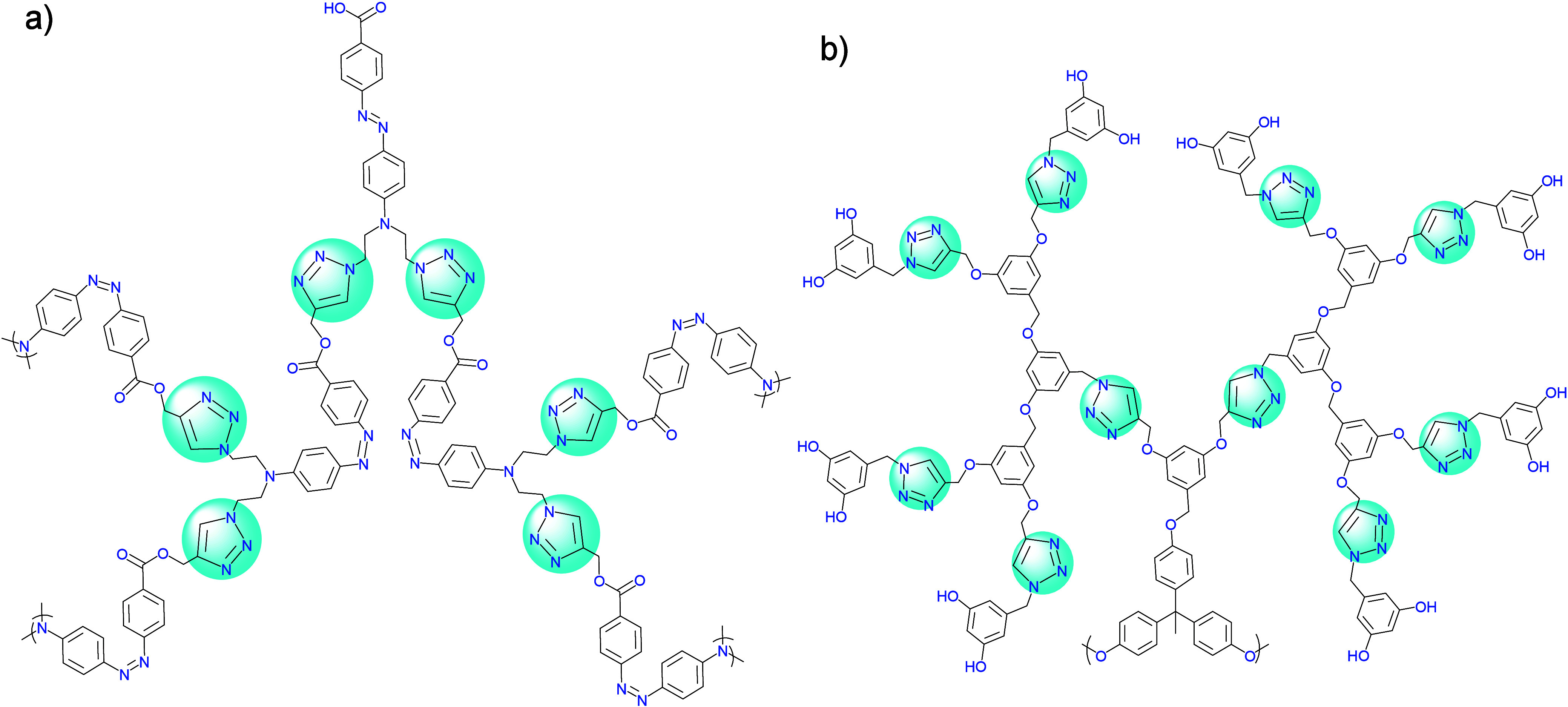
(a) Azobenzene-centered dendrons (G4 generation) prepared by Liu and co-workers via CuAAC. Adapted with permission from ref (103). Copyright 2008 ACS.^103^ (b) Triazole/benzyl-ether dendrimer (G4 generation) prepared by Malkoch and co-workers. Adapted with permission from ref (104). Copyright 2007, RSC.^104^

As mentioned earlier, traditional dendrimer synthesis typically involves iterative activation-functionalization steps requiring two synthetic steps per generation. However, using click chemistry, researchers managed to generate several generations in one step per generation. For instance, Liu and co-workers reported a novel four-step synthesis of triazole dendrons (G4 generation; Figure 9a), which were prepared by iterative CuAAC/S_N_2 reactions with as many as 15 azobenzene moieties in the main structure.^103^

Similarly, a highly efficient route to click chemistry-based G4 dendrimers was introduced by Malkoch et al. using the sequence CuAAC–ether formation–CuAAC–ether formation, or CuAAC–ester formation–CuAAC–ester formation.^104^ Using this protection group-free strategy, they prepared two dendrimers, 2,2-bis(methylol)propionic acid (bis-MPA)-triazole and benzyl ether (Frechet type)-triazole, having 24 surface hydroxyls in only four steps (Figure 9b). Moreover, Kakkar and his group combined two different click reactions [CuAAC (CuSO_4_, ascorbate, THF/H_2_O, microwave, 65 °C) and Diels–Alder (EtOAc, 50 °C)] in a sequential manner for G3 level dendrimer formation.^105^ This strategy was further modified by the Malkoch and Hawker groups by the combination of thiol–ene (UV light, 365 nm, THF) and CuAAC (CuSO_4_, ascorbate, THF-H_2_O) reactions, which allowed them to make even G6 level dendrimers in a single day.^106^

In nature, innumerable pathological and biological processes are controlled by carbohydrates. Many recognition events such as hormone mediation, pathogen invasion, fertilization, and toxin–cell and cell–cell interactions depend on multivalent carbohydrate–receptor interactions.^107^ Henceforth promoting/inhibiting these natural carbohydrate–receptor interactions has gained much attention. Glycodendrimers and other synthetic multivalent glycoconjugates were recognized as potential candidates for interacting with these receptor targets.^108^ Groundbreaking work in the field of glycodendrimers development using unprotected carbohydrates came from the groups of Liskamp/Pieters,^109^ Finn/Fokin/Sharpless/Hawker,^97^ and Fernandez-Megia/Riguera.^110^ The group of Fernandez-Megia and Riguera developed gallic acid triethylene glycol (GATG) dendrimers by incorporating terminal azide groups on their periphery. Typical reaction conditions involved a little excess of alkynated carbohydrates (CuSO_4_, ascorbate, *t*-BuOH-H_2_O), followed by isolation of the resulting functionalized dendrimers using ultrafiltration. This efficient synthesis route of glycodendrimers and PEG-dendritic block copolymers deliberately excluded the scope of dendrimer dimerization. CuAAC also exhibited its potential to functionalize solid supports.^111−116^ Santoyo-Gonzalez demonstrated for instance a glycol dendrimer with a silica core and showed the potential of the materials toward chromatographic protein separation.^117^ In 2007, Zuilhof et al. successfully coupled the ideal cholera toxin (CT) ligand, a GM1-oligosaccharide (GM1os), to dendritic structures with long spacer arms by very effective CuAAC click chemistry coupling. The best structure was an astounding 380,000-fold more powerful ligand for the toxin than a monovalent GM1os derivative in the inhibition experiments.^118^ Similarly in a recent work, Tiwari et al. reported the successful development of a new series of galactosylated dendrimers bearing 6, 12, or 18 peripheral galactose units by exploiting a CuAAC reaction strategy.^119^ Moreover, Kannan et al. employed CuAAC and photoinduced thiol–ene click chemistry reactions to develop a novel neuroinflammation-targeting polyethylene glycol-based dendrimer (PEGOL-60) with a high hydroxyl surface density.^120^ This PEGOL-60 dendrimer is an effective vehicle to specifically deliver compounds to sites of the central nervous system (CNS), and can thereby enhance the therapeutic outcome of a range of neuroinflammatory diseases.^120^ A final example of bioactive glycodendrimers was recently provided by the Hernaiz group,^121^ which developed glycodendrimers via the CuAAC reaction of aromatic scaffolds, functionalized with terminal ethynyl groups and glucuronic acid azides. They first confirmed that these glycodendrimers show molecular docking activity against the Dengue virus envelope protein 2 (DENV2). Additionally, they observed in simulation studies that glycodendrimers could act as a potential Dengue virus antagonist.

**Figure 10:**
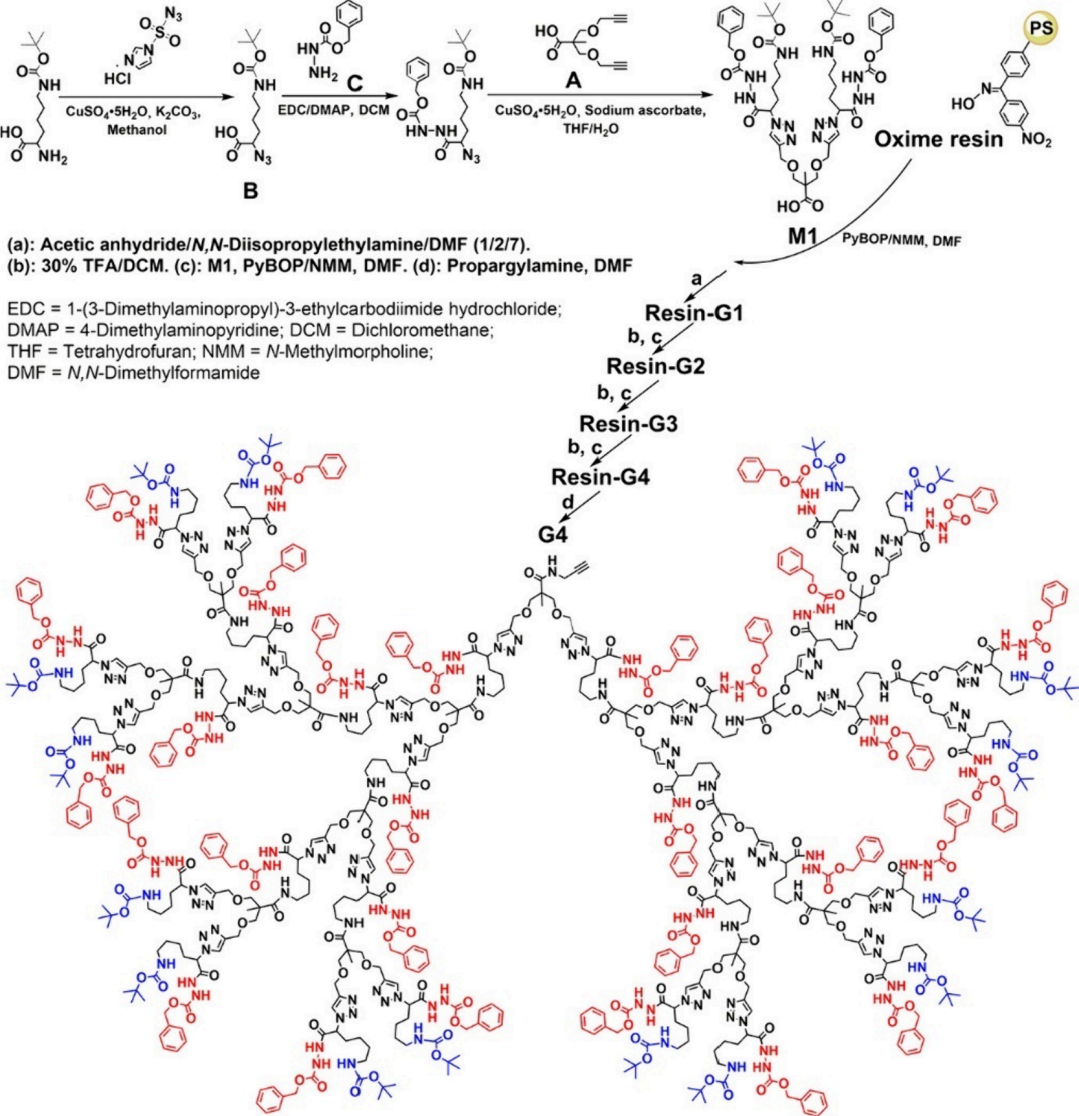
Scaffold-modifiable bifunctional dendrons prepared by Wu and co-workers using clickable precursors up to the 4th generation via an orthogonal amino protection strategy and solid-phase synthesis. Adapted with permission from ref (122). Copyright 2022, ACS.^122^

In a recent study, Wu and co-workers^122^ prepared a new class of scaffold-modifiable bifunctional dendrons using clickable precursors up to the fourth generation via an orthogonal amino protection strategy and a solid-phase synthesis method. The azido-bearing intermediate formed by amidation between 2-azido-6-*tert*-butoxycarbonylamino-hexanoic acid and carbobenzoxyhydrazide was then coupled with 2,2-bis(propargyl) propionic acid through a CuAAC reaction to form the G4 dendrons (Figure 10). Subsequently, conjugation of DOX and PBA groups was achieved to the scaffold and periphery of the dendrons, respectively. The PBA-modified dendron-based prodrug had a hydrodynamic diameter of ∼7 nm, a drug loading content larger than 30%, and displayed a desirable water solubility. Thiol–ene chemistry was also used for the final functionalization step in the synthesis of amine-functionalized antibacterial dendrimers, using trimethylolpropane (CH_3_CH_2_CH(CH_2_CH_2_OH)_3_) as a branching agent.^123^ As example of yet another click chemistry used to decorate dendrimers, the McReynolds group used the oxime-hydrazone click reaction to synthesize a series of oligosialic acid-based glycodendrimers.^124^ The oxime bonds were formed chemoselectively and in good to excellent yields (58–100%) between unprotected sugars (sialic acid or α-2,8-linked di-through tetrasialic acids) and the aminooxy-terminated dendrimer core in a microwave-mediated reaction, to provide dendrimers designed as broad-spectrum inhibitors of viral pathogens.

#### 2.2.2 Hyperbranched Polymers

**Figure 11:**
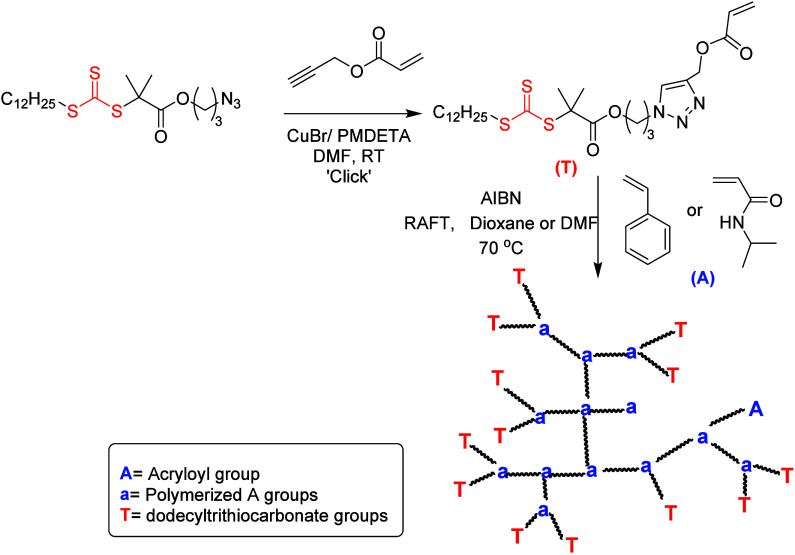
Synthesis of an acryloyl trithiocarbonate chain transfer agent via CuAAC and subsequent hyper-branched polymer preparation using RAFT polymerization. Adopted with permission from ref (128). Copyright 2007, CSIRO.^128^

Hyperbranched polymers possess similar properties to dendrimers, with many functional groups distributed throughout the polymeric backbone and side chains.^125^ Their highly branched structure influences the physical and chemical properties by yielding a low glass transition temperature, a low intrinsic viscosity, and improved solubility because of the low degree of crystallinity and chain entanglement.^126^ The main benefit of these hyperbranched polymers over dendrimers is their ease of synthesis, while they display a concomitant loss of order and definition. A common two-step method to prepare hyperbranched polymers involves living radical polymerization (LRP) reactions followed by click chemistry-based post polymerization modifications. The hyperbranched copolymers afforded by Sumerlin and co-workers using azido-functionalized RAFT chain transfer agent [CTA]^127^ by esterification of carboxyl-containing CTA precursors with 3-azidopropanol. Further, azido-functionalized RAFT CTA was converted into a triazole group containing acryloyl trithiocarbonate by treating with propargyl acrylate utilizing the CuAAC reaction. The resulting acryloyl CTA also acts as a monomer and was copolymerized with styrene and *N*-isopropylacrylamide through a subsequent RAFT polymerization to afford hyper-branched copolymers (Figure 11).^128^

**Figure 12:**
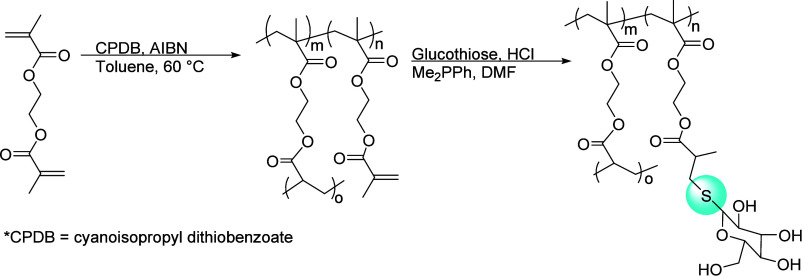
Synthetic strategy for the preparation of hyperbranched glycopolymers. Adapted with permission from ref (126). Copyright 2010, ACS.^126^

Later in 2010, Perrier and co-worker reported a synthesis strategy to produce densely functionalized branched and hyperbranched glycopolymers by combining RAFT polymerization of ethylene glycol dimethacrylate (EGDMA) and various click chemistries (CuAAC, thiol–ene, and thiol–yne; see an example with thiol–ene chemistry in Figure 12).^126^

**Figure 13:**
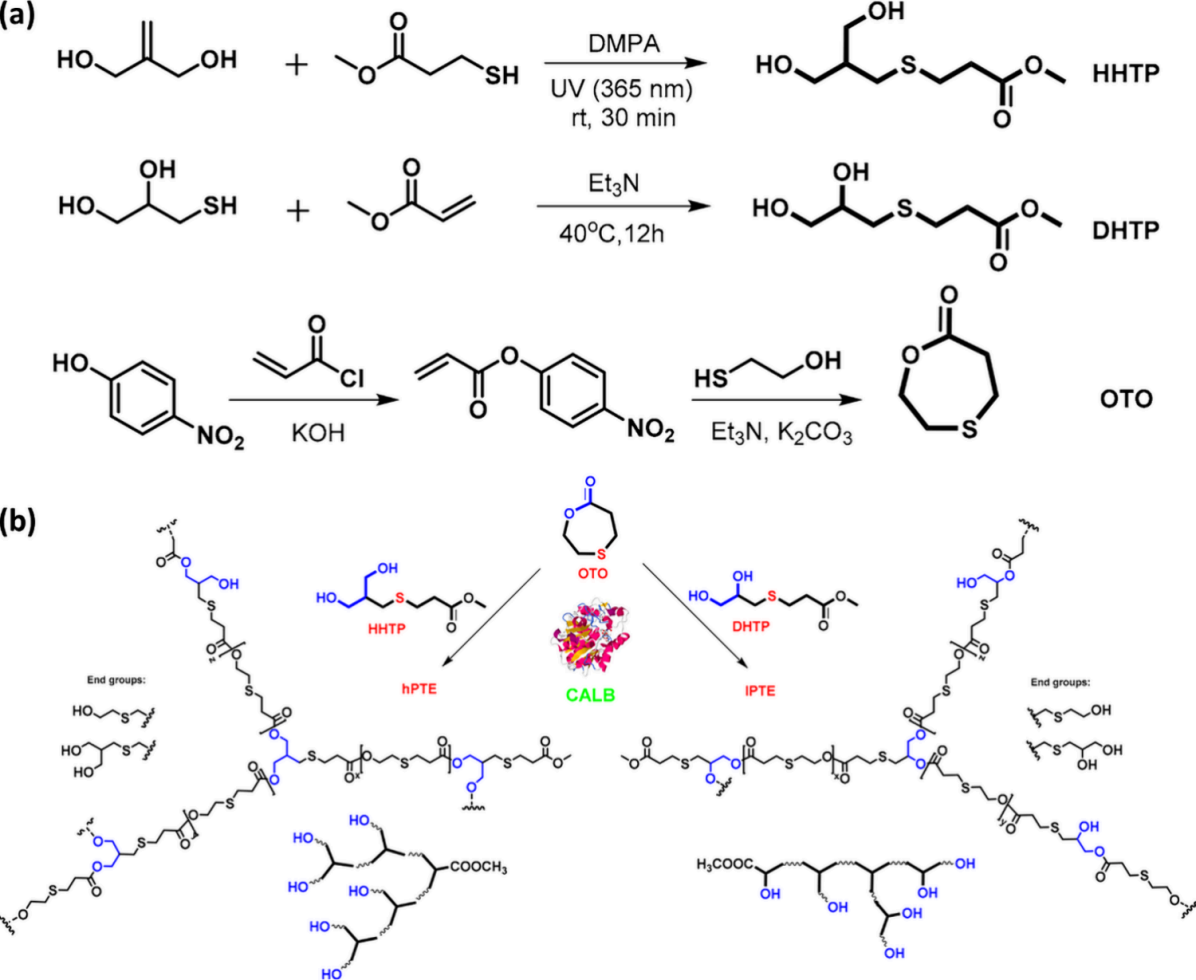
(a) Synthesis of click based monomers OTO, HHTP, and HTP using thiol–ene chemistry. (b) Enzymatic ring-opening polycondensation of 1,4-oxathiepan-7-one (OTO) and HHTP or DHTP. Adapted with permission from ref (129). Copyright 2020, MDPI.^129^

More recently, Liu and co-worker developed hyperbranched aliphatic poly(β-thioether ester)s by the enzymatic ring-opening polycondensation of 1,4-oxathiepan-7-one (OTO) and 3-((3-hydroxy-2-(hydroxymethyl)propyl)thio)propanoate (HHTP) or methyl 3-((2,3-dihydroxypropyl)thio)propanoate (DHTP) (see Figure 13).

These HHTP (primary alcohols) and DHTP (secondary alcohols) monomers were synthesized by thiol–ene click chemistry and thiol-Michael addition, respectively.^129^ In 2017, Wei and co-workers developed a hyperbranched, disulfide bond-containing block-statistical copolymer-based prodrug by RAFT-self-condensing vinyl polymerization (RAFT-SVCP) and post olymerization click coupling, to create a drug carrier that was sensitive to both pH and S–S bond reduction.^130^ Here, glycidyl methacrylate-derived hyperbranched polymers were reacted with sodium azide, to obtain an azide-functionalized hyperbranched polymer. This could then be successfully conjugated with the alkyne-modified anticancer drug camptothecin (CPT) using a CuAAC reaction.

#### 2.2.3 Bottlebrush Graft Copolymers and Surface-Bound Polymer Brushes

Bottlebrush polymers are a subclass of graft copolymers where side chains are densely packed onto a polymeric backbone to construct a wormlike or bottlebrush-like conformation.^131−133^ The conformation of individual polymers mainly depends upon the grafting density and conformational entropy forces,^134^ while the overall mechanical properties are highly influenced by both this average grafting density and the dispersity. Depending on the synthetic approaches, three main grafting strategies have been used: grafting to, grafting from, and grafting through.^135^ A highly effective coupling method is the main concern for achieving high grafting densities and a low dispersity *Đ*. While more methods for this are under development and displaying great promise,^136^ here we aim to illustrate the state-of-the-art by focusing on click-mediated grafting-to strategies based on CuAAC and thiol–ene reactions.

##### 2.2.3.1 Grafting Using CuAAC Reaction

**Figure 14:**
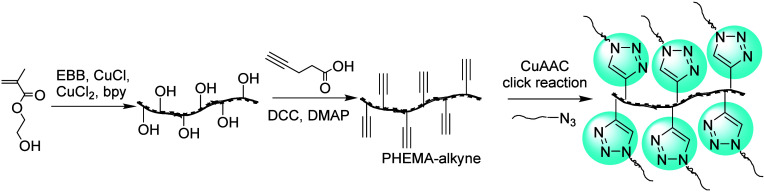
Grafting-to bottlebrush synthesis of graft copolymers containing PS, PBA, PBA-*b*-PS, and PEG side chains. Adapted with permission from ref (137). Copyright 2007, ACS.^137^

The first click chemistry-based breakthrough in the grafting-to strategy was made by Matyjaszewski et al. in 2007, in their synthesis of graft copolymers using the combination of ATRP and a CuAAC reaction as shown in Figure 14.^137^ They synthesized a linear poly(2-hydroxyethyl methacrylate) (PHEMA) polymers by ATRP, and then used post polymerization esterification using a DCC coupling reaction to append a high density of alkyne moieties. Five varieties of azido-terminated polymeric side chains such as poly(ethylene glycol)-N_3_ (PEG-N_3_), polystyrene-N_3_, poly(*n*-butyl acrylate)-N_3_, and poly(*n*-butyl acrylate)-*b*-polystyrene-N_3_ were subsequently linked onto the polymeric backbone by CuAAC reactions.

**Figure 15:**
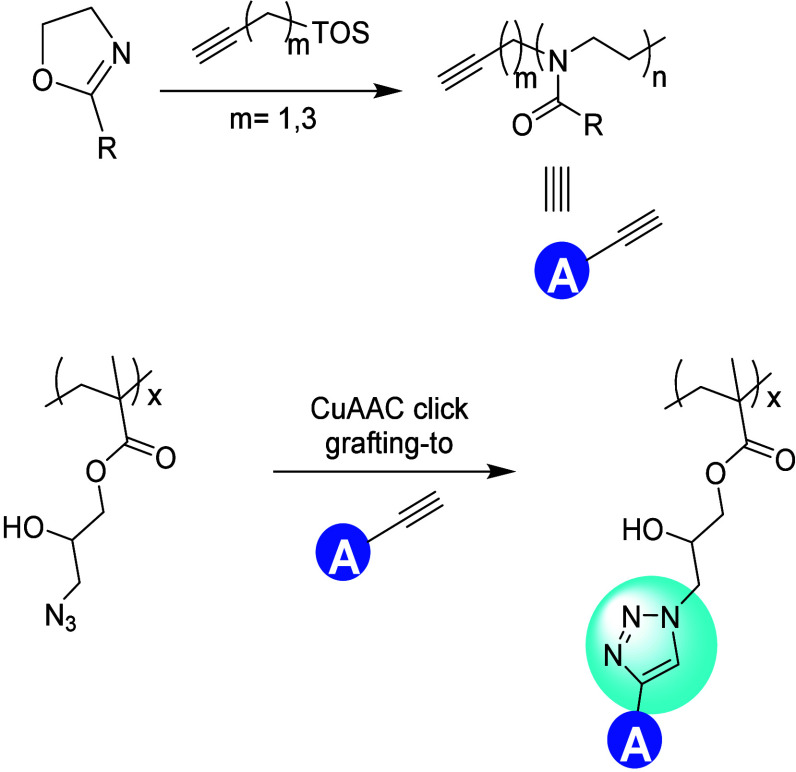
Bottlebrush synthesis by sequential formation of an alkyne-functionalized poly(2-oxazoline) and reacting that by a CuAAC coupling with a poly(3-azido-2-hydroxypropyl methacrylate). Adapted with permission from ref (138). Copyright 2018, Wiley.^138^

Along similar lines, Chu et al. have also prepared polymer brushes with the grafting-to strategy using a CuAAC coupling reaction (Figure 15).^138^ Poly(glycidyl methacrylate) backbones were labeled with azide-containing side chains that were ready for CuAAC reactions. Then, a set of poly(2-oxazoline)s was synthesized from different initiators (such as pent-4-ynyl tosylate or propargyl tosylate), and reacted to obtain CuAAC-linked polymer brushes. They noted that the initiation efficiency depended on the alkyne used to initiate the poly(2-oxazoline) formation, and also showed how the grafting density varied with the degree of polymerization (DP or *n*) and alkyl chain length (*R*) of the poly(2-oxazoline). Specifically, shorter *R* and lower *n* values reduced the steric hindrance and significantly improved the grafting density.

**Figure 16:**
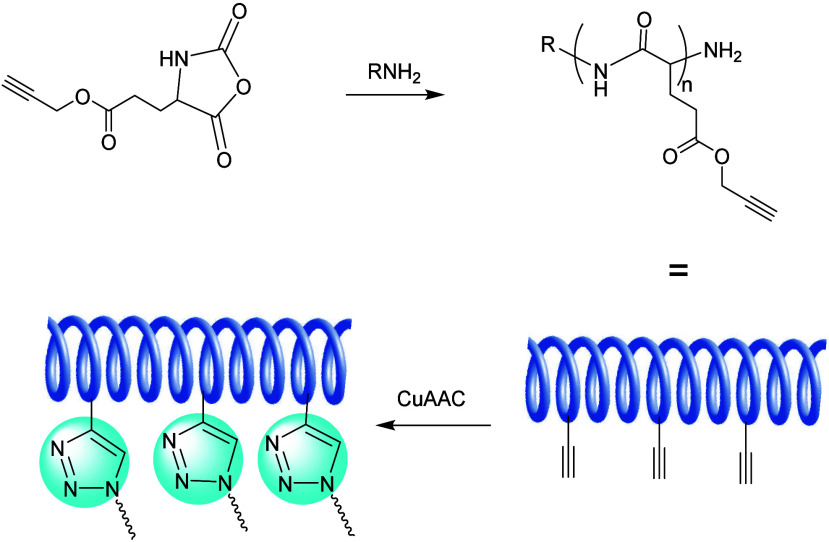
Synthesis strategy of graft copolymers consisting of PPLG backbone and PEG side chains through a CuAAC click reaction.^139^

This influence of the chemical structure of the polymeric backbone on the grafting density was also demonstrated by Hammond et al.^139^ Graft copolymers were synthesized from a poly(g-propargyl-l-glutamate) (PPLG) backbone and PEG side chains through the combination of ROP of *N*-carboxyanhydride and a CuAAC reaction (Figure 16). In this work a high grafting density was achieved, partially because of the rigid α-helical structure of the PPLG backbone, in which the alkynyls on the backbone project outward, and as such increased their accessibility to couple with the azido groups in the PEG side chains.

**Figure 17:**
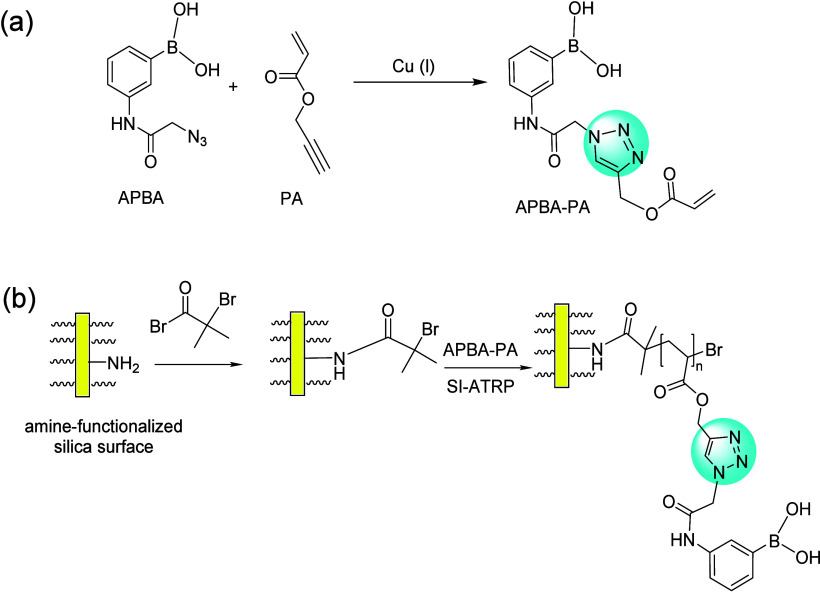
(a) Synthesis of boronic acid monomer APBA-PA. (b) Synthesis of poly(APBA-PA) on silica using surface-initiated ATRP.^140^

Whereas bottlebrush polymers are typically constituted by functionalizing chains onto a one-dimensional polymer chain, analogous functionalization can be performed onto a two-dimensional surface as well. If the grafting density is high enough, the polymer chains will be directed away from the surface, yielding what is labeled a polymer brush. Analogous to bottle-brush strategies, grafting of a surface can also be achieved via surface-initiated ATRP. Xu et al. used this approach to graft a fluorogenic boronic acid polymer onto amino-functionalized silica (Figure 17).^140^ They first prepared a fluorogenic boronic acid monomer by conjugating an azide-terminated phenylboronic acid with propargyl acrylate through a CuAAC reaction. Amino-functionalized silica particles were then used to anchor the ATRP initiator, from which the polymer brush was grown. This new composite material displays a fascinating fluorescence intensity shift, when linked with sugar moieties at physiological pH conditions, and allowed for the fast separation of a model glycoprotein via a selective boronate affinity interaction.

Wu et al. worked on an alternative “grafting-to”-based click chemistry strategy for designing a polymer brush with thermally stable and hydrophobic cellulose nanocrystals (CNCs) in the core and polycaprolactone diol (PCL-diol) in the branched brush.^141^ First, CNCs were modified with azides, while PCL-diol was modified with alkyne moieties. Next, a CuAAC reaction was performed to generate the CNC-*g*-PCL polymer brush, showing that CuAAC is an appealing modification strategy for grafting polyester chains onto CNCs. Recently, Tasdelen et al. reported polypropylene-*graft*-poly(l-lactide) (PP-*g*-PLAs) copolymers prepared by CuAAC using alkyne end-functionalized poly(l-lactide) (PLA-alkyne) and azide side-chain functionalized polypropylene (PP-N_3_).^142^ Here, chlorinated polypropylene (PP) was modified by using an azido-trimethyl silane-tetrabutylammonium fluoride (TMS-N_3_/TBAF) system. The polymer (PLA-alkyne) was polymerized via ROP of *L*-lactide in the presence of a tin(II) octanoate catalyst and a propargyl alcohol (PA) as an initiator. The CuAAC reaction was applied to produce PP-*g*-PLAs with two different grafting densities by varying the feed ratios of alkyne/azide-functionalized polymers at room temperature. In continuation of their previous work, Tasdelen and co-worker also developed the graft copolymers PP-*g*-PEG and PP-*g*-PCL by a CuAAC “click” reaction.^142^ They used the same strategy to modify chlorinated polypropylene (PP) with an azide functionality and PEG and poly(ε-caprolactone) (PCL) with an alkyne functionality.

**Figure 18:**
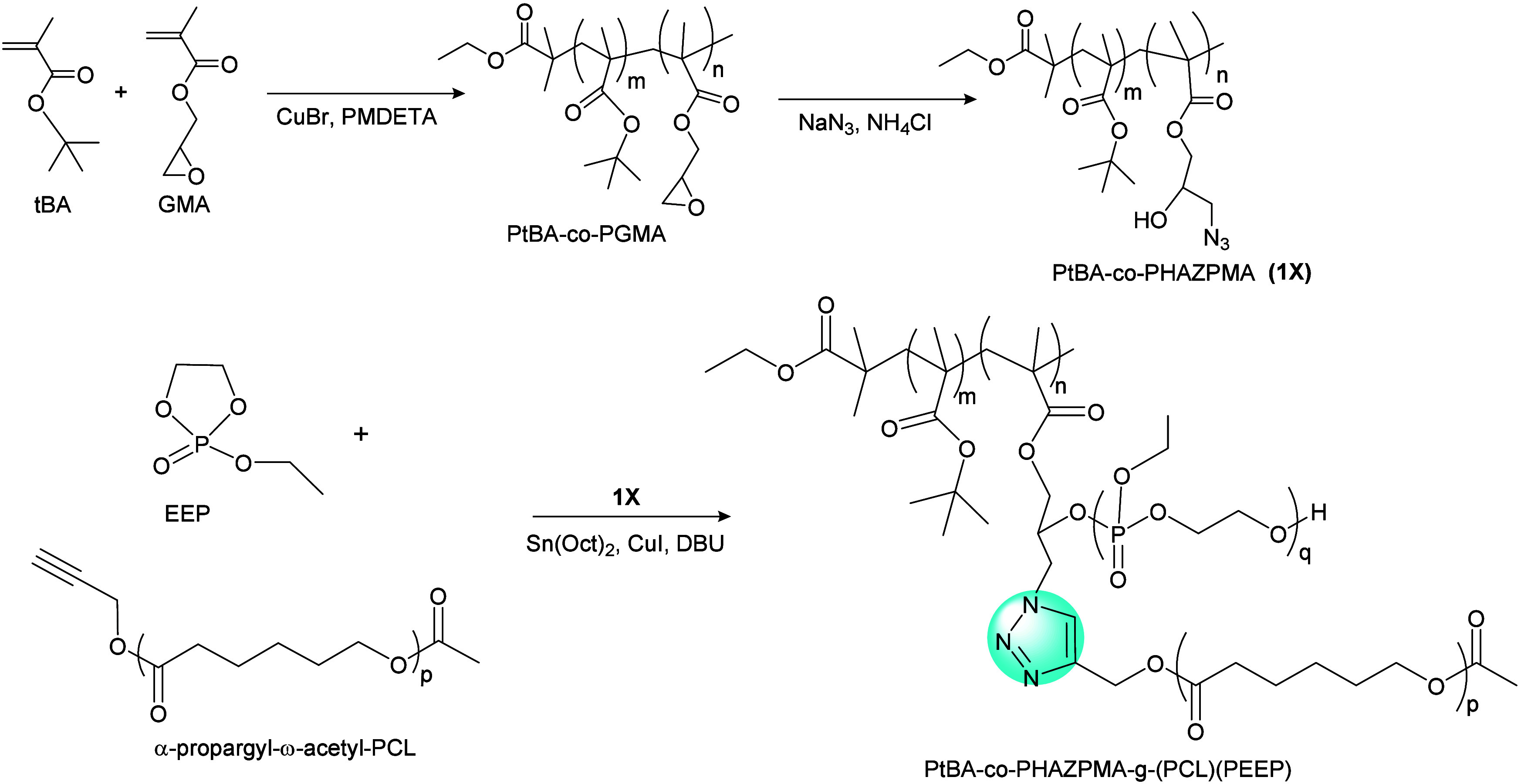
Synthesis route of amphiphilic centipede-like brush copolymer P*t*BA-*co*-PHAZPMA-*g*-(PCL)(PEEP) by a combination of ROP and CuAAC.^143^

A combination of grafting-to and grafting-from polymerization was reported by Yuan et al. to construct highly branched multifunctional polymers. First, they prepared an azide-functionalized copolymer (poly(*tert*-butyl methacrylate)-*co*-poly(2-hydroxy-3-azidopropyl methacrylate (**1X**; Figure 18). Branching of this azide-functionalized polymer was then achieved in a ROP reaction, to yield amphiphilic centipede-like brush copolymers with hydrophobic PCL and hydrophilic poly(ethyl ethylene phosphate) (PEEP) side segments and a backbone of PtBA-*co*-PHAZPMA.^143^ The combination of ROP of 2-ethoxy-2-oxo-1,3,2-dioxaphospholane together with hydroxyl and azide groups on each repeating unit of poly(glycidyl methacrylate) (PGMA) was used for CuAAC click chemistry with a-propargyl-ω-acetyl-PCL.

**Figure 19:**
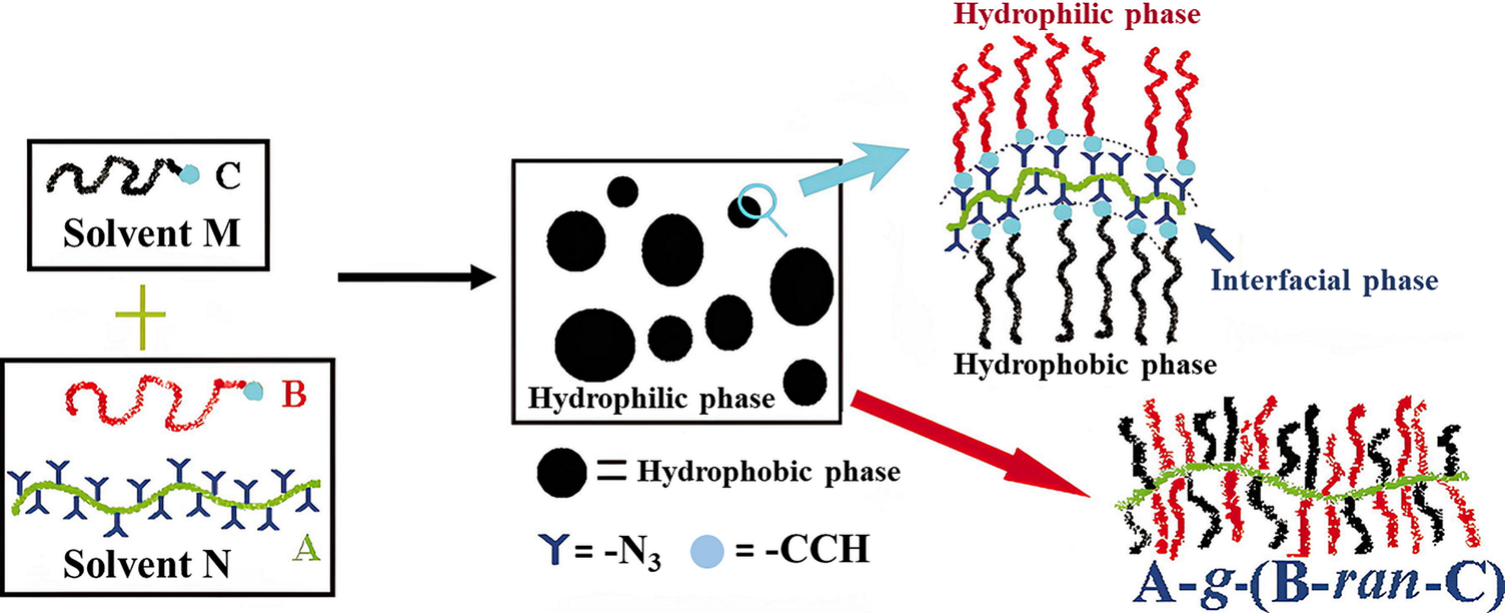
Fabrication of CBPs in two immiscible solvent pairs by interfacial CuAAC click reaction. Adapted with permission from ref (144). Copyright 2007, Wiley.^144^

Lu et al. described an efficient strategy for fabricating distinct cylindrical polymers (CBPs) in two immiscible solvents by using interfacial CuAAC chemistry with hydrophobic and hydrophilic side chains linked to the linear backbone (Figure 19).^144^ Interestingly, when these CBPs were synthesized from the CuAAC click reaction in a single solvent in a homogeneous state, they showed a low grafting density of less than 55% and a *Đ* of more than 1.78. These results suggest that here an effective click reaction can take place at the interface of both solvents, and each of these two solvents should at least dissolve one of the reaction substrates.

##### 2.2.3.2 Grafting Using Thiol Click Reactions

Thiol click reactions include a set of click reactions where thiol groups are involved such as thiol–ene, thiol–yne, thiol–epoxy, thiol–bromo, thiol–isocyanate, and thiol–*para*-fluoro reactions.^145^ However, among all these thiol-click reactions, the thiol–ene reaction is most prevalently used for the synthesis of (bio)functional polymers, owing to its metal-free, radical-mediated, and base-promoted features.^146^ In addition, thiol–ene reactions are also not so sensitive to moisture and oxygen/air, and display a high degree of regioselectivity in the thioether formation.

**Figure 20:**
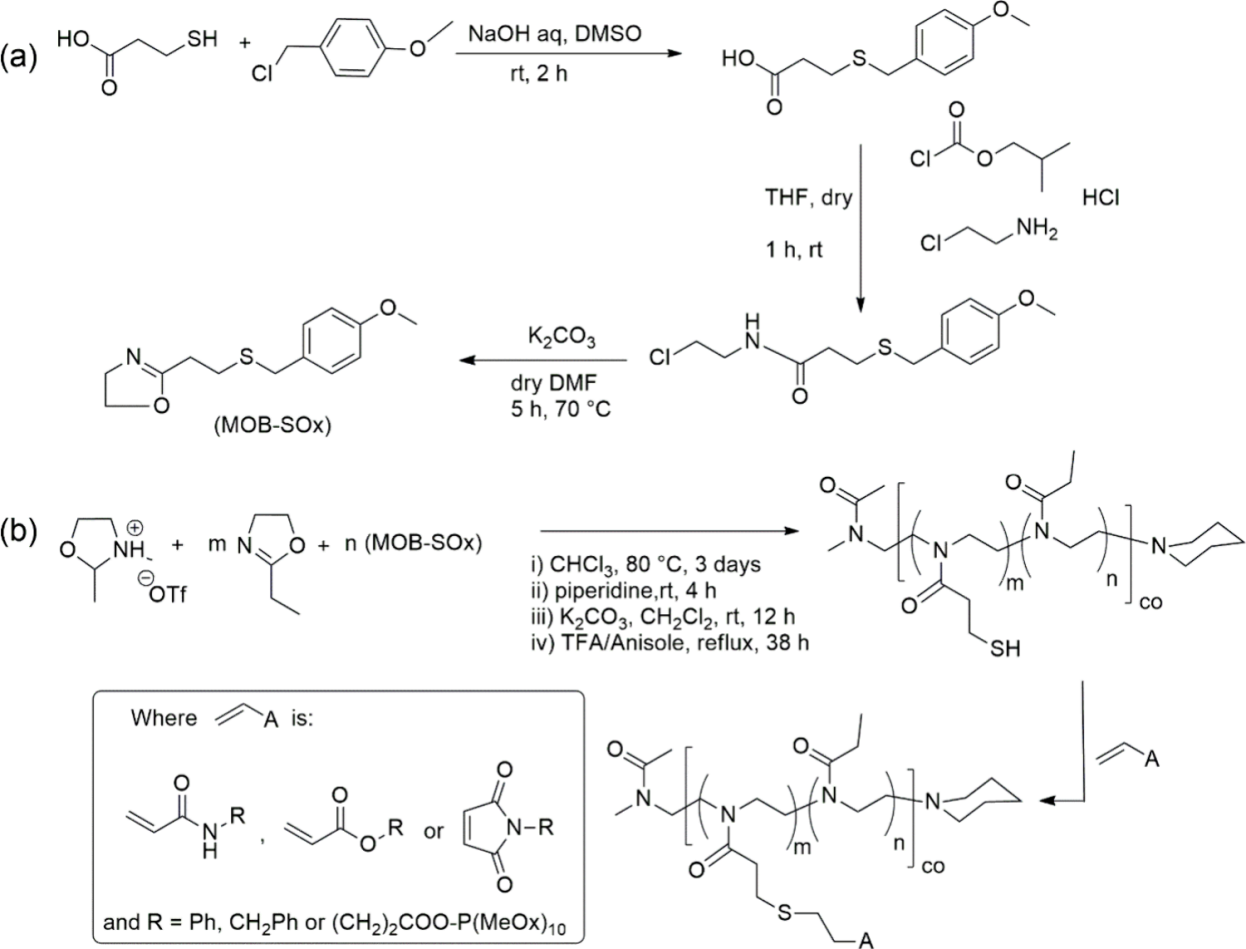
(a) Synthesis of 2[2-(4-methoxybenzylsulfanyl)ethyl]-2-oxazoline (MOB-SOx), and (b) its polymerization and subsequent thiol–ene-based functionalization.^147^

In this regard, Nuyken et al. reported a poly[2-(4-methoxybenzylsulfanyl)ethyl-2-oxazoline]-*co*-poly(ethyl-2-oxazoline) copolymer using a thiol click reaction (Figure 20).^147^

**Figure 21:**
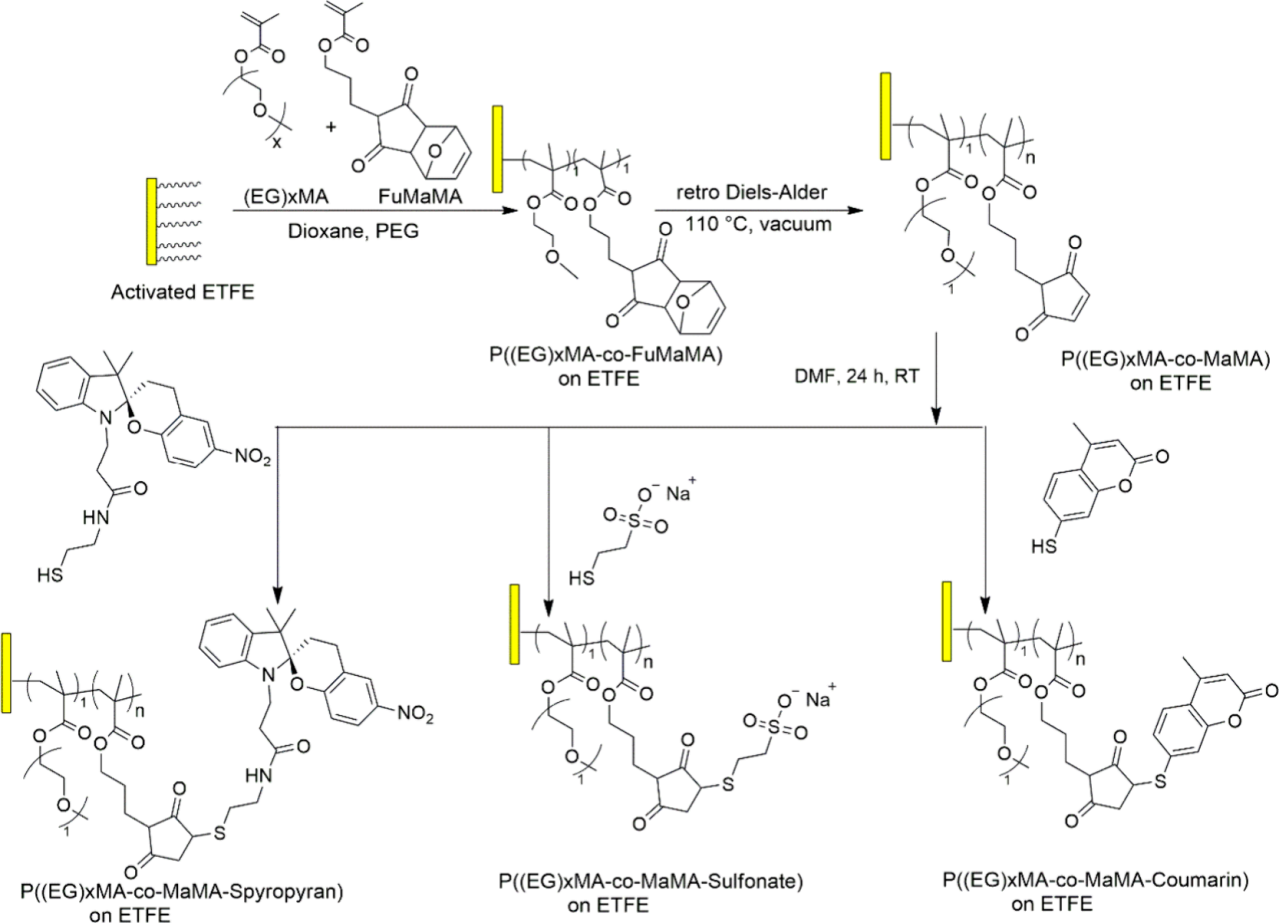
Synthetic strategy for copolymer brushes via grafting of P((EG)_*x*_MA-*co*-FuMaMA) and the subsequent retro Diels–Alder (rDA) reaction used to activate the maleimide groups.^148^

This graft copolymerization started with the synthesized 2[2-(4-methoxybenzylsulfanyl)ethyl]-2-oxazoline (MOB-SOx) together with comonomers *N*-methyl-2-methyl-2-oxazolinium triflate (MeOx-OTf) and 2-ethyl-2-oxazoline. The subsequent reaction was a thiol–ene coupling reaction of *N*-phenyl-acrylamide (PhA) and benzylmaleimide after quantitative deprotection of the pendant thiol groups (BzM). Graft copolymers were formed by reacting SH-containing polymers with poly(2-methyl-2-oxazoline)s containing acrylamide (PMeOx_10_A) and maleimide (PMeOx_10_M) as terminal reactive groups. Dubner et al. also reported a successful preparation of thiol-reactive polymeric brushes on a polymeric substrate of poly(ethylene-*alt*-tetrafluoroethylene) (ETFE) by extreme ultraviolet (EUV) lithography (as shown in Figure 21).^148^ Specifically, to grow this brush structure on the polymeric substrate, free-radical polymerization of masked maleimide monomers was used, which was induced by the UV light of the EUV interference lithography that also allowed pattern formation. Nucleophilic attachment of thiols to the deprotected maleimide side chains was chemoselective and specific.

Kim et al.^149^ also presented the creation of precisely structured bottlebrush polymers (BBPs) with well-defined chemical configurations in 2022. This group employed an iterative convergent growth approach, and developed a copolyester backbone, incorporating up to 64 repeating units of lactic acid (LA) and 2-hydroxy-4-pentenoic acid in an alternating sequence. Through the application of thiol–ene click chemistry under UV light (λ = 365 nm), discrete side chains such as polylactides (LAn-SH) were selectively attached to the vinyl groups of the 2-hydroxy-4-pentenoic acid residues on the copolyester backbone, leading to the formation of a fully grafted BBP, t-LP(LA_8_)32-LA_8_, with a molecular weight of 34 kDa. Furthermore, an amphiphilic bottlebrush block copolymer (BBCP) with a molecular weight of 19 kDa was synthesized, integrating hydrophobic dodecanethiol and discrete poly(ethylene glycol) side chains. The resulting BBCP exhibited self-assembly into well-defined micelles in water. These discrete BBCPs offer a promising avenue for studying the influence of branched architecture on the physical and chemical characteristics of BBCPs in both bulk and solution phases.^149^

Hence, we can summarize that both radical-based and nucleophile-based thiol–ene click chemistry offers a versatile strategy to couple different polymers to develop graft and brush copolymerization. Also compared to, e.g., CuAAC, each technique has its own pros and cons (e.g., a strong biomimetic triazole might be preferred in some cases, while in others a weaker thioether bond or the complete absence of metals could be desirable), and as such insight into the full set of click reactions is preferred to make a proper choice.

##### 2.2.3.3 Grafting Using Other Click Reactions

**Figure 22:**
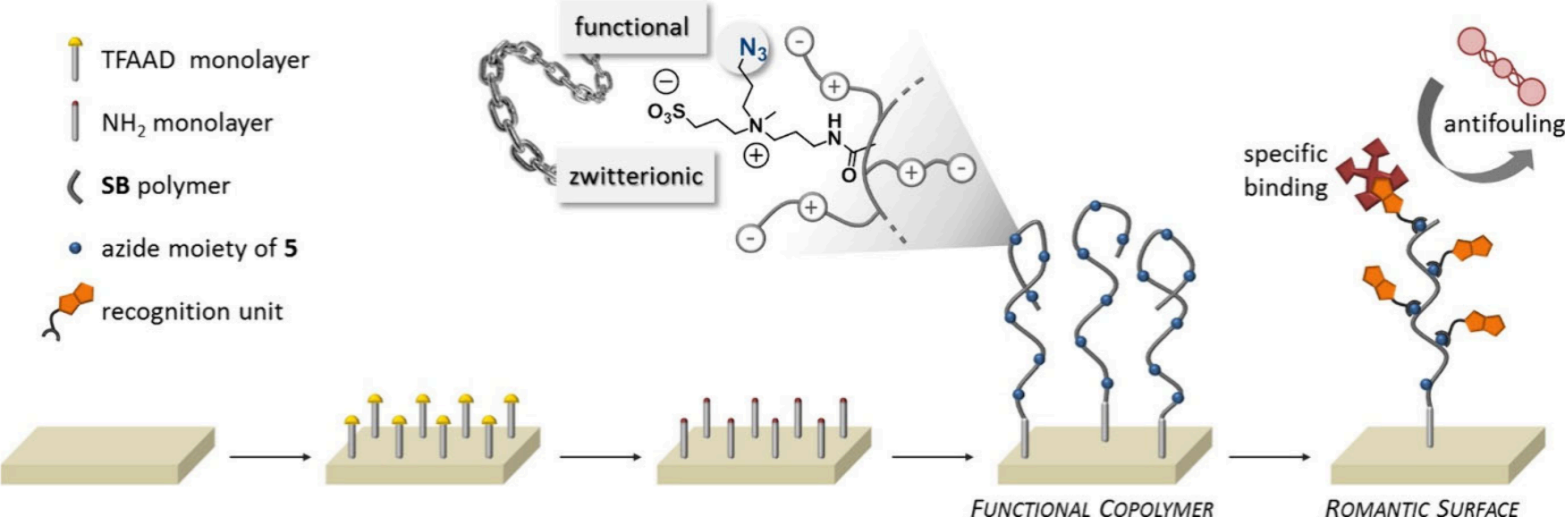
Schematic illustration of functional antifouling polymer brushes as derived from the copolymerization of an azido-group containing a clickable monomer with a standard sulfobetaine monomer. Adapted with permission from ref (150). Copyright 2016, ACS.^150^

While CuAAC and thiol click reactions have been used most often, several other strategies are worth mentioning for the fabrication of graft or brush polymers. Zuilhof and his team recently developed a new metal-free approach by combining zwitterionic and clickable moieties in a single monomer.^150^ In this technique, they have attached newly designed sulfobetaine-based zwitterionic monomer furnished with a clickable azide moiety to produce highly charged but overall strictly neutral antifouling surface coatings without compromising the sensing capability of the brush. To this aim, they copolymerized the N_3_-functionalized monomer with a standard sulfobetaine monomer to attain a brush with large numbers of clickable groups for possible further modifications. In this work, strain-promoted alkyne–azide click (SPAAC) reactions were used to functionalize these azido brushes to obtain fully zwitterionic 3D-functionalized coatings with a recognition unit of choice (Figure 22), while avoiding the need for (intrinsically cytotoxic) copper salts that might be difficult to fully remove from the brush afterward.

**Figure 23:**
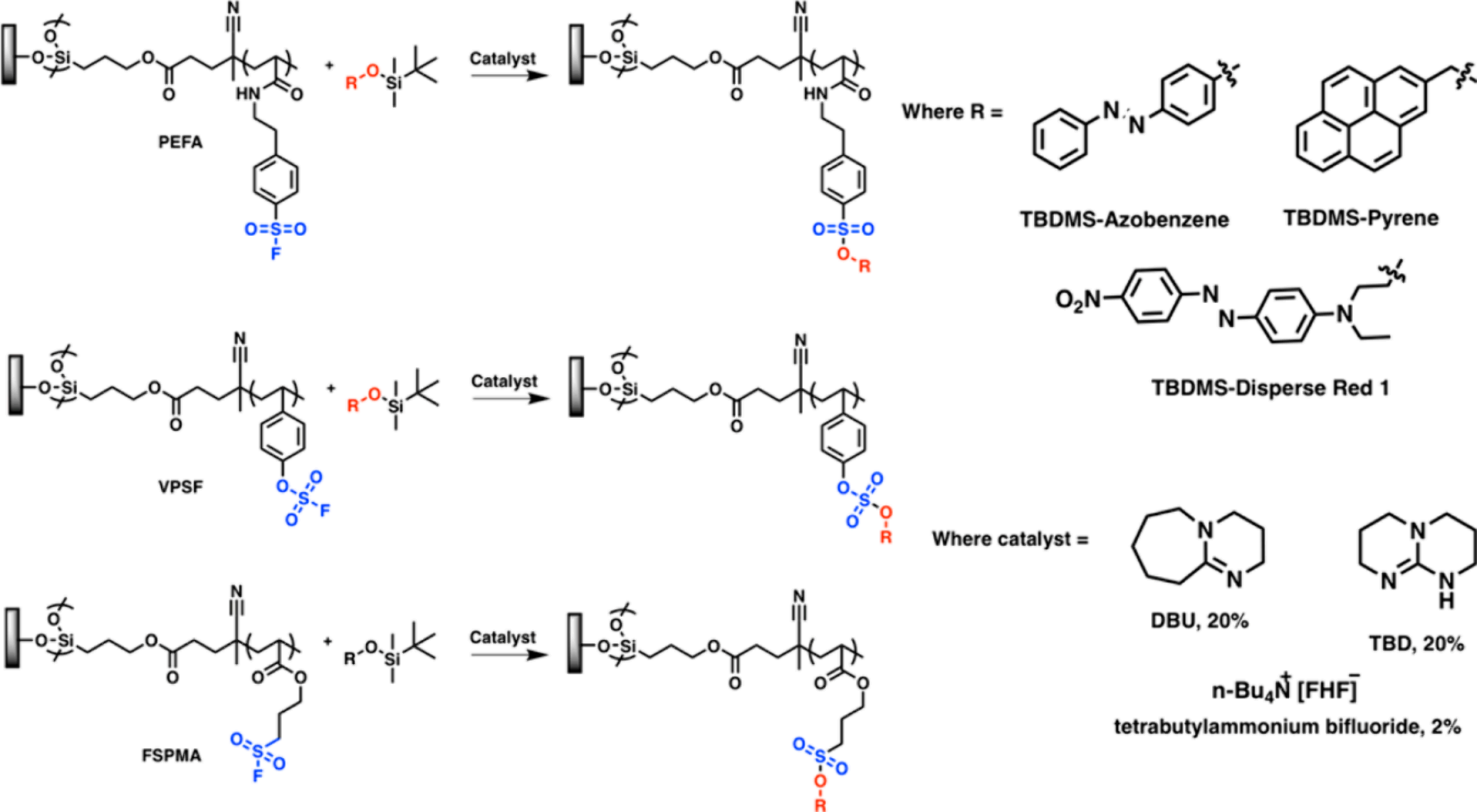
Synthetic scheme for SuFEx post polymerization modification on aryl sulfonyl fluoride, aryl fluorosulfonate, and alkyl sulfonyl fluoride polymer brushes. Adapted with permission from ref (151). Copyright 2021, ACS.^151^

Since the sulfur(VI) fluoride exchange (SuFEx) reaction inception in 2014, its has emerged as a promising addition to polymer chemistry, facilitating both polymerization across diverse backbones and post polymerization modification (PPM). Bottle brush polymers formation will likely be explored more extensively with SuFEx in the future as well. Both multimodal SuFEx polymers^75^ and demonstrated SuFEx-based grafting onto polymer brushes^151^ demonstrate the ease and potential of this technique (Figure 23).

Locklin and co-workers successfully demonstrated the utility of SuFEx chemistry in surface derivatization via PPM of sulfonyl fluoride-containing polymer brushes.^152^ The authors presented an investigation utilizing three distinct polymer brush systems—alkyl sulfonyl fluorides, aromatic sulfonyl fluorides, and aromatic fluorosulfonates—each subjected to a SuFEx reaction with three different silyl ether derivatives (aryl, alkyl, and benzyl). Furthermore, they delved into the utilization of TBDMS brushes and their interaction with fluorosulfonate derivatives, revealing an unexpected absence of surface reaction. Such detailed studies provide significant light onto the often complex kinetics and constraints of the use of click reactions for surface derivatization.^152^ Given this initial success and the array of conditions, substrate varieties, and catalyst options available with this reaction, there likely exists a significant potential for further explorations of the SuFEx reaction for PPM on polymer brushes, aiming to delineate its advantages and constraints in surface conjugation.

**Figure 24:**
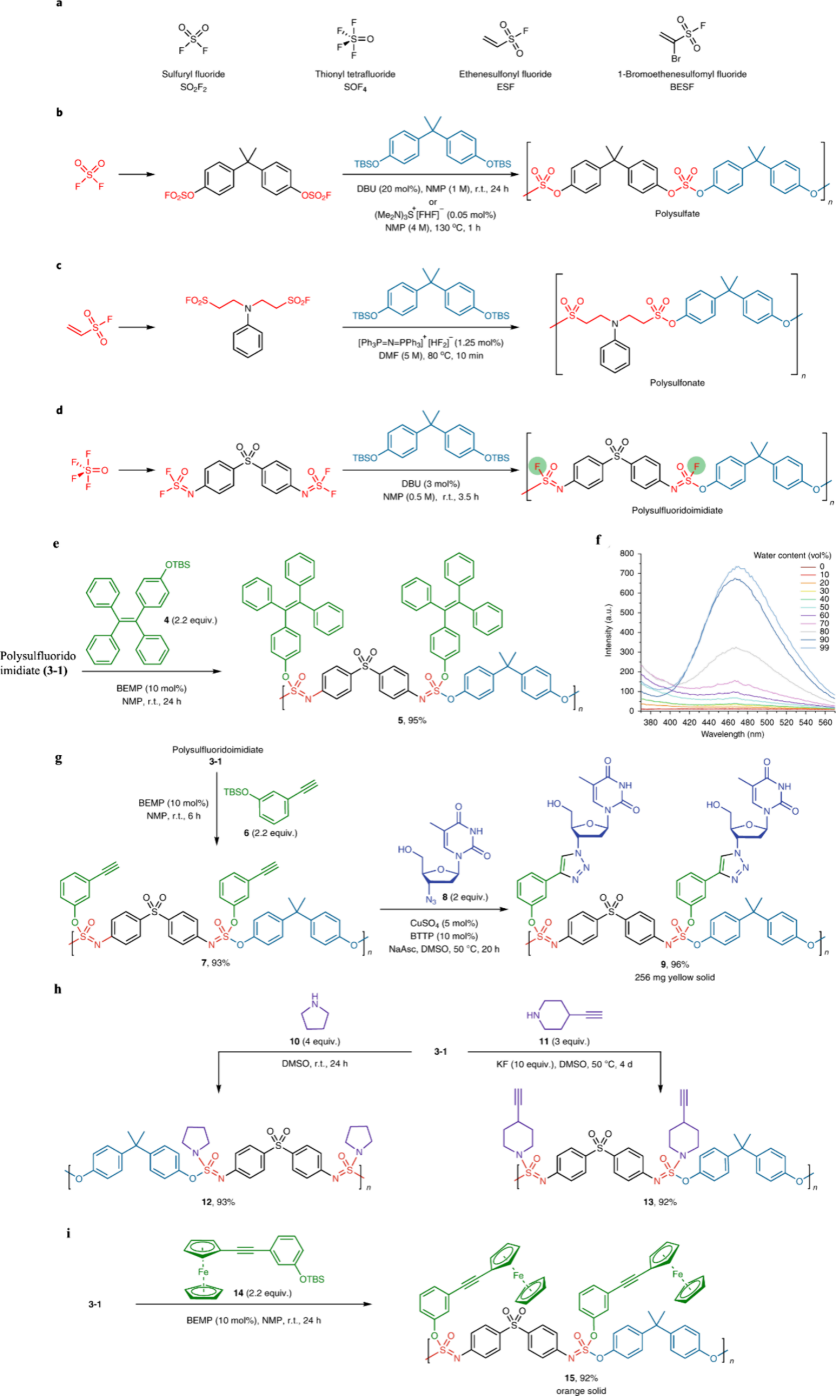
(a–c) Various SuFEx click chemistry approaches for polymer synthesis via Connective SuFEx hubs from SO_2_F_2_. (SuFExable S–F bonds for post polymerization backbone modification are highlighted in green.) (e,f) Postpolymerization modification of polysulfluoridoimidiate (3-1) using sequential SuFEx and CuAAC click chemistry. Adapted with permission form ref (75). Copyright 2021 Nature.^75^

The Sharpless group^75^ conducted SuFEx click chemistry to synthesize a range of structurally diverse copolymers derived from SOF_4_, employing bis(iminosulfur oxidifluorides) and bis(aryl silyl ethers) in the polymerization process. This class of polymers exhibits two notable characteristics. First, the [−N=S(=O)F–O−] linkages constituting the polymer backbone are themselves SuFExable, allowing for precise postmodification with phenols or amines to generate branched functional polymers. Second, examination of individual polymer chains from several of these novel materials reveals helical polymer structures. The robustness of SuFEx click chemistry presents opportunities for post polymerization modification, facilitating the synthesis of materials with controlled composition and conformation (Figure 24).^75^

#### 2.2.4 Star Polymers

**Figure 25:**
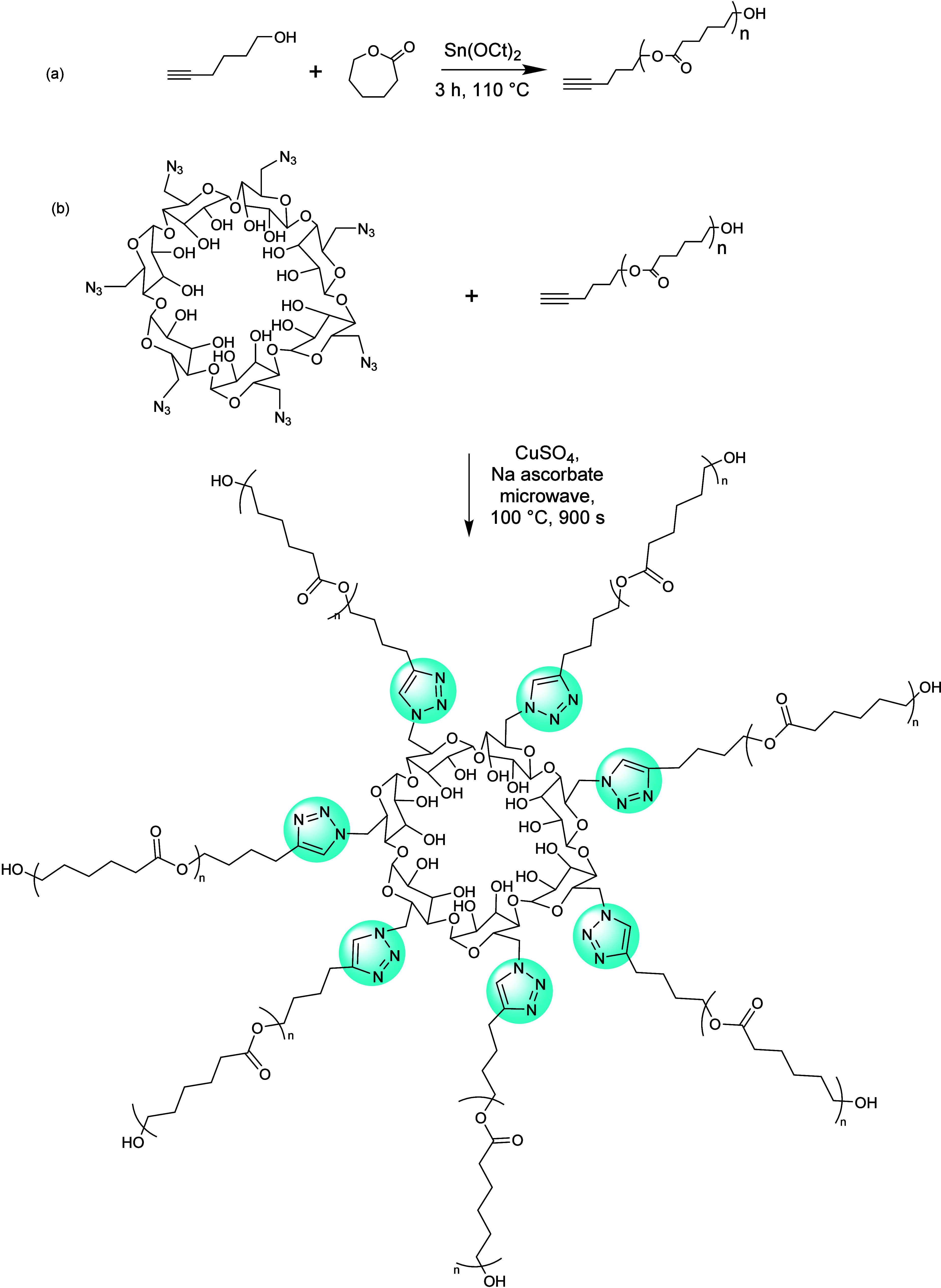
(a) Schematic representation of the synthesis of the acetylene-functionalized poly(ε-caprolactone). (b) Schematic representation of the CuAAC reaction of acetylene-functionalized poly(ε-caprolactone) to heptakis-azido-*b*-cyclodextrin resulting in heptakis-poly(ε-caprolactone)-*b*-cyclodextrin.^160^

The star architecture belongs to a special type of branched polymers where multiple arms are anchored on a central core. These arms could be further decorated with side chains to produce a complex topological architecture. Depending upon the grafting density of the star polymer, it could be either classified as a loosely or densely grafted star copolymer. Synthesis of these star polymers with precise control over their microstructure, composition, and topology is a challenging task. In this context, click chemistry reactions serve a vital role and offer versatile star architectures with great selectiveness and yield.^153−159^ Most of these star polymers are synthesized in several steps combining CuAAC reactions with ROP, ATRP, and RAFT. Schubert et al. prepared a star-shaped poly(ε-caprolactone) by azide–alkyne cycloaddition (Figure 25b).^160^ To prepare the star-shaped polymer, acetylene-functionalized poly(ε-caprolactone) was coupled to a heptakis-azido-*b*-cyclodextrin by a CuAAC reaction. The acetylene functionalization was carried out via ROP in the presence of a tin(II) octanoate catalyst and unprotected 5-hexyn-1-ol initiator (Figure 25a).

**Figure 26:**
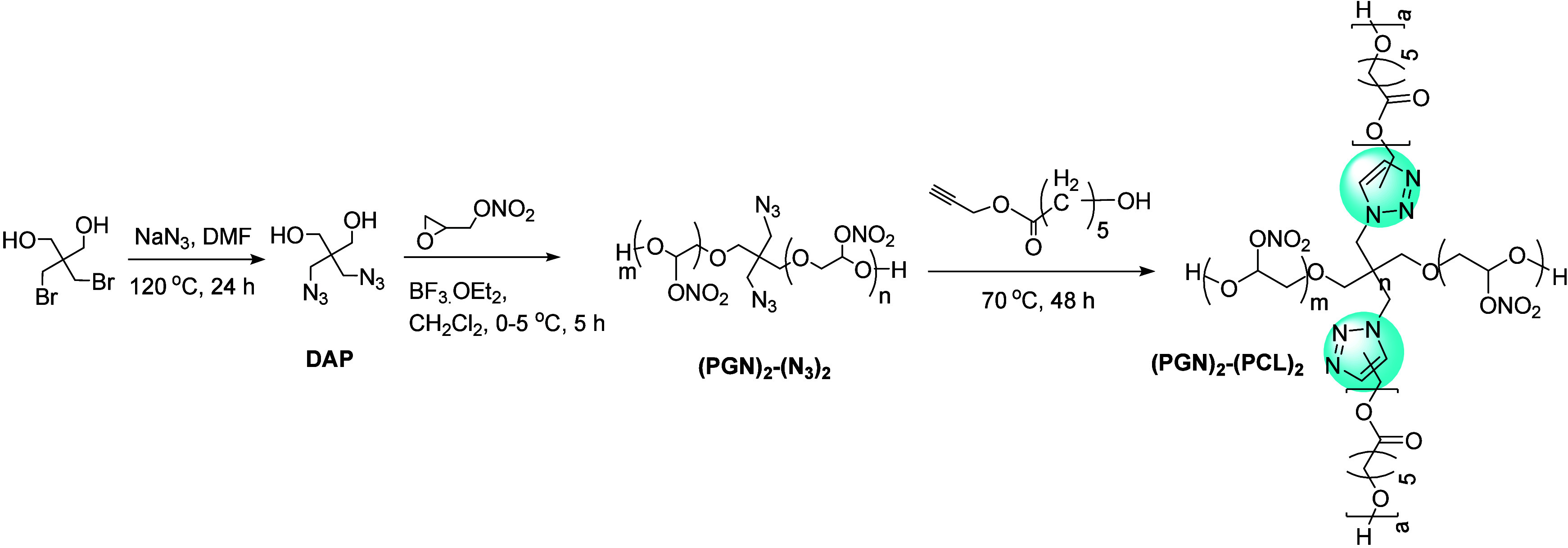
Synthesis route of miktoarm (PGN)_2_-(PCL)_2_ polymers.^159^

In 2009, Qiao et al. pioneered the synthesis of a multiarm star brush copolymer by a so-called “arm first” strategy.^161^ In this technique, ε-caprolactone was polymerized by ROP in the presence of a 2-hydroxyethyl 2′-methyl-2′-bromopropionate initiator. This was followed by ATRP of a protected acetylene monomer, such as (trimethylsilyl)propargyl methacrylate, to obtain a block copolymer macroinitiator. Then a bislactone monomer (4,4′-bioxepanyl-7,7′-dione) was used to cross-link this hydroxyl end group-containing block copolymer macroinitiator under ROP conditions. Finally, a deprotection reaction was employed to produce a degradable core cross-linked star (CCS) polymer with a high degree of active clickable sites in the core. Takata et al. reported a stepwise solvent-free nitrile *N*-oxide-mediated 1,3-dipolar cycloaddition click reaction for the synthesis of a PEG-containing star polymer composed of a nitrile *N*-oxide-bearing PEG via living anionic polymerization.^158^ In this manner they developed three, four, and six-armed star polymers by simply altering the clickable alkenyl group-containing core moieties. Recently, Bodaghi et al. used polyglycidyl nitrate (PGN) and poly(ε-caprolactone) (PCL) to develop a novel A_2_B_2_-type energetic miktoarm star-shaped copolymers made by the combination of ROP and CuAAC.^159^ To prepare a copolymer, at first, a diazido end-functionalized two-arm PGN was developed by ROP from glycidyl nitrate monomers followed by a CuAAC reaction with propargyl-terminated PCL to obtain the (PGN)_2_-(PCL)_2_ star copolymer. This strategy to synthesize copolymer overcomes the PGN binder problem (high glass transition temperature) along with the energy constraints of PCL (Figure 26).

**Figure 27:**
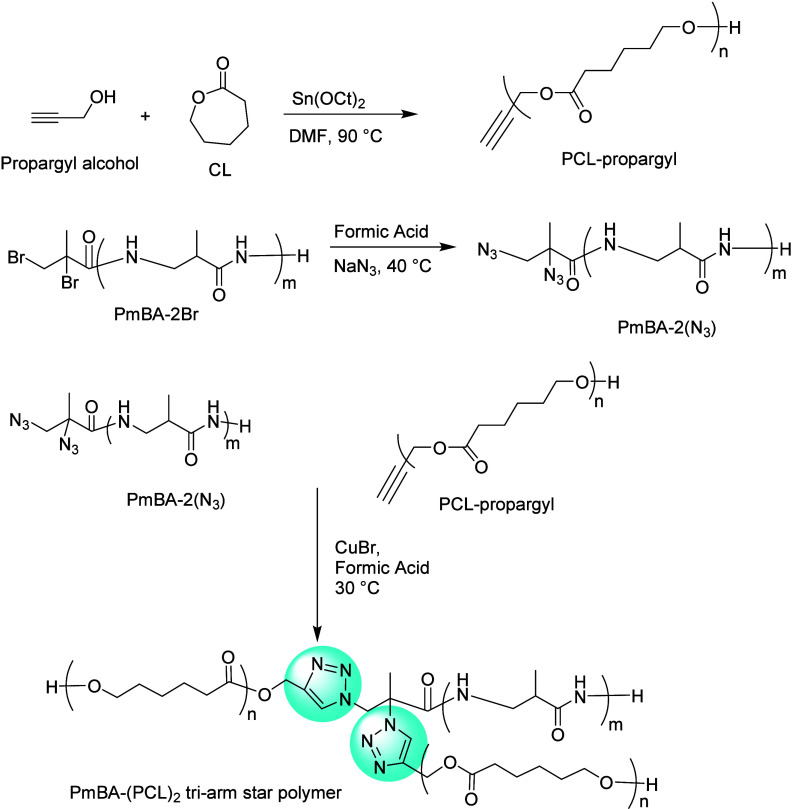
Reaction pathways in the synthesis of PmBA-N_3_ and PCL-propargyl. Reaction outline for the synthesis of PmBA-(PCL)_2_ triarm star polymer.^162^

Somewhat analogously, Meyvaci et al. designed a triarmed star poly(α-methyl-β-alanine)-poly(ε-caprolactone) [PmBA-(PCL)_2_].^162^ ROP between propargyl alcohol and ε-caprolactone was used to obtain propargyl poly(ε-caprolactone) [PCL-propargyl]. In parallel, they employed hydrogen transfer polymerization (HTP) to create PmBA-2Br from methacrylamide. This also provides two Br atoms in the end group, and conversion of these two Br atoms in PmBA-2Br with sodium azide, forms terminally difunctionalized azide poly(α-methyl-β-alanine) [PmBA-2N_3_]. Finally, a triarmed star polymer PmBA-(PCL)_2_ was formed by the reaction of PCL-propargyl and PmBA-2N_3_, through a CuAAC reaction (shown in Figure 27). The resulting polymer showed great prospects in the development of future therapeutics delivery systems. In another recent work, Tang and co-workers employed a combination of hydroxyl–yne and thiol–ene click reactions for the synthesis of sequence-defined miktoarm star oligo(monothioacetal) polymers.^163^ The star oligo(monothioacetals) were synthesized using both divergent and convergent strategies. The synthesis starts with easily accessible starting materials: *p*-tolylmethanol, methyl propiolate, and 2-mercaptoethanol. Initially, *p*-tolylmethanol undergoes a reaction with methyl propiolate in the presence of 1,4-diazabicyclo[2.2.2]octane (DABCO), resulting in the formation of a β-alkoxyacrylate-containing compound. Subsequently, this β-alkoxyacrylate compound reacts with the thiol groups of 2-mercaptoethanol to produce the target sequence-defined macromolecule. This approach is efficient and eliminates the need for conventional protecting and deprotecting steps commonly associated with the synthesis of similar star polymers.

In general, methods of star copolymer synthesis are often complicated, and precise control over the inclusion of clickable sites onto the core and side chains of the start copolymer remains challenging. Considering this and their potential for further applications given their controlled multifunctionality, this area prvides ample potential for future research.

### 2.3 Network Polymers (Hydrogels)

Among all the network structures, hydrogels are getting special attention owing to their vast applications in the biomedical field. An adequately designed hydrogel can imitate living cell conditions or be a vehicle for drugs. Although the precise application may dictate specific features, in general hydrogels should possess critical features like: biocompatibility, minimal toxicity, stability in water and air, orthogonal reactivity of cross-linking moieties with the other functional groups in the material. This requires that their formation is chemoselective, controllable and displays fast cross-linking kinetics. The latter are, of course, among the characteristics of click chemistry.^164^

Click chemistry has thus been employed extensively for the formation of polymeric networks. Simple model networks with homogeneous cross-linking density and well-defined pore size could be produced by cross-linking telechelic polymers using their multifunctional end groups. In 2006, Turro and co-workers developed such kind of model network by the combination of ATRP and CuAAC.^165^ They synthesized α-ω-diazido-poly(*tert*-butyl acrylate) (PtBA) from tBA using a difunctional initiator by ATRP, followed by bromine end groups substitution with azide. The resulting diazido-PtBA was then cross-linked via a CuAAC reaction with tri- and tetra-alkyne coupling agents. Size exclusion chromatography suggested that the amount of unreacted material was higher in the network generated from the tetra-arm cross-linker over the network from the triarm coupling agent, which was explained in terms of increasing steric hindrance in the tetra-arm system. A similar strategy was employed by Kornfield et al., when they synthesized a liquid crystalline gel by using ring-opening metathesis polymerization (ROMP) to produce azide-terminated functionalized polycyclooctene, which was then followed by CuAAC chemistry by reacting this polymer with a tripropargylamine core.^166^ The resulting swollen gels exhibited fast and reversible optical switching under applied electric fields.

Simultaneous interpenetrating polymer networks (sIPNs), in which different polymers are intertwined, are like an “alloy” of crosslinked polymers, have also been prepared using click chemistries, and are of interest due to their specific bi-functional properties. Such properties endow them with features ideal for applications in biomedical materials, biomaterials for contact lenses, drug delivery systems, and artificial organs.^167^ In 2010, Kang et al. reported simultaneous CuAAC and ATRP reactions from a mixture of poly(ethylene glycol)-diazide (N_3_-PEG-N_3_), tetrakis(2-propynyloxymethyl) methane (TPOM), ethyl-2-bromobutyrate, CuBr, PMDETA, and 2-hydroxyethyl methacrylate (HEMA) in DMF.^168^ These hydrogels demonstrated a fast gelation rate and high gel yield with high swelling ratios and good mechanical and antifouling properties. The authors also observed that the gelation time could be reduced by increasing the amount of the Cu(I) catalyst.

**Figure 28:**
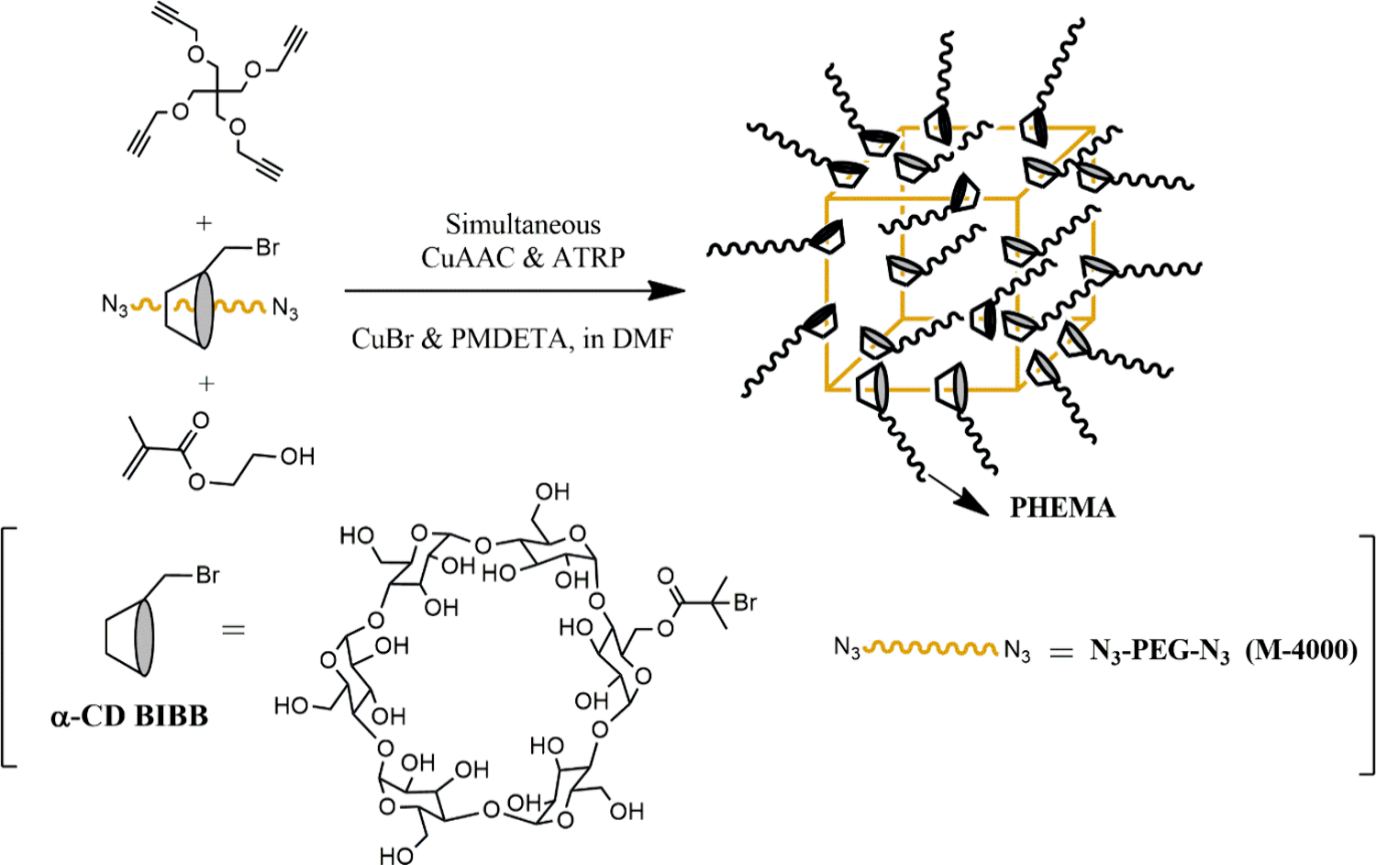
Synthesis of PEG (*M*_n_ = 4000 Da) network with movable sliding-grafted (PHEMA) (s-IPN-PEG/a-CD-sg-PHEMA) via simultaneous ATRP and CuAAC chemistry.^169^

Another sIPN with unique molecular structures was reported by Lin and co-workers. They synthesized a PEG network with tunable sliding-grafted-PHEMA (sIPN-PEG/R-CD-sg-PHEMA) hydrogels by simultaneous CuAAC reaction and ATRP (see Figure 28).^169^ The presence of the R-CD-sg-PHEMA chains allowed to diminish the free volume of the PEG network lattices; moreover, the entanglement of these sliding chains would impede the complete stretching of the network lattices, resulting in a lower degree of swelling, while the sliding itself is a neat way to reduce stresses in the material.

**Figure 29:**
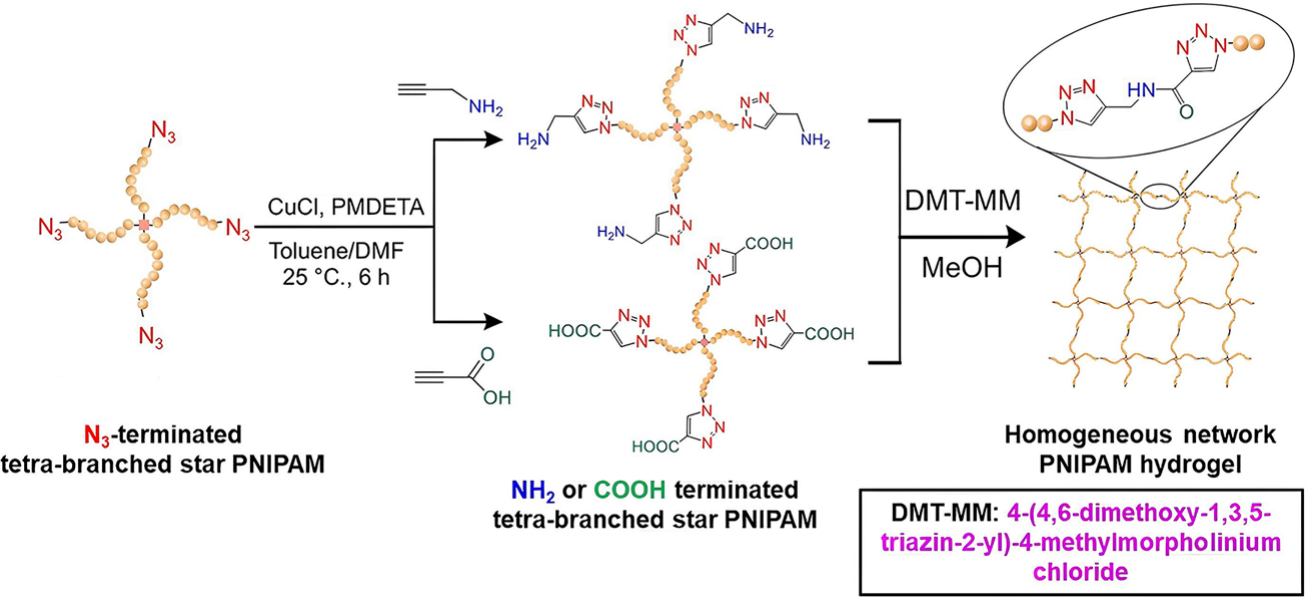
Two-step synthesis of PNIPAM gel: Synthesis of NH_2_ or COOH-terminated tetra-branched star PNIPAM by CuAAC click reactions, followed by an amide-forming condensation reaction between the termini of these tetra-branched star PNIPAMs. Adapted with permission from ref (170). Copyright 2019, ACS.^170^

In another example, Takeoka and group members synthesized a uniform polymer network of a 4-star poly(*N*-isopropylacrylamide) (PNIPAM), using a combination of a single-electron transfer living radical polymerization (SET-LRP), CuAAC click reaction, and amide bond formation.^170^ At first, a 4-armed star polymer of NIPAM was synthesized using a 4-branched initiator through SET-LRP.^171,172^ Then by using a CuAAC click reaction, an amino group or a carboxyl group was incorporated at each of the four ends of the star polymer. Therefore, two types of building blocks were synthesized separately, which were then mixed in equal amounts. Finally, the resulting amide formation reaction leaded to the formation of a star-shaped polymer network of the PNIPAM (see Figure 29). This approach for the preparation of a star-shaped polymer network allows universal application, and can be easily used for other monomers. Moreover, the absence of complicated operations during the synthesis of 4-branched initiator and 4-branched star polymer increased the system’s credibility.

#### 2.3.1 Hydrogel Preparation via CuAAC Click Reactions

**Figure 30:**
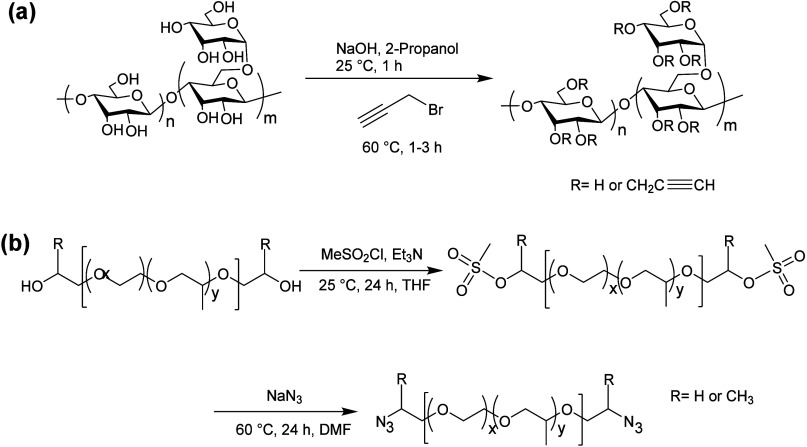
(a) Guar-based hydrogels can be synthesized from alkynylation of the guar polymer, followed by (b) coupling with α,ω-diazido-PEG-*co*-PPG]. Adapted with permission from ref (178). Copyright 2010, Wiley.^178^

In 2006, Ossipov and Hilborn demonstrated one of the first syntheses of polymeric hydrogels with tailor-made mechanical properties using CuAAC, by modifying poly(vinyl alcohol) (PVA) with a fraction of either acetylene or azide groups.^173^ Gao et al. imitated the natural cartilage extracellular matrix by designing a biological hydrogel using CuAAC chemistry.^174^ For this, they modified both hyaluronic acid (HA), and chondroitin sulfate (CS) with 11-azido-3,6,9-trioxaundecan-1-amine (AA), and gelatin (G) functionalized with propionic acid (PA). After proper mixing of HA-AA, CS-AA, and G-PA in the presence of Cu(I) as catalyst, the desired biological hydrogel was formed. Adhesion of chondrocytes on the material and proliferation was confirmed by *in vitro* cell culture assessment. Heinze et al. prepared a novel cellulose-based hydrogel based on CuAAC cross-linking.^175^ In their study, they observed that control over the degree of functionalization and amount of Cu(I) catalyst allows to easily regulate the gelation time of the polymer. This is a general feature used by many reserachers since. Using CuAAC between azide-modified cellulose and an alkyne-modified P(NIPAM-*co*-HEMA), Zhang and co-workers reported the synthesis of a thermosensitive hydrogel with a porous network and temperature-dependent swelling ratio and reswelling kinetics.^176^ Such an approach displays the potential for the *in situ* formation of hydrogels from natural polysaccharides. In another work, Mortisen et al. combined the RAFT polymerization and CuAAC reaction to design a semisynthetic, thermoreversible hyaluronan-poly-NIPAM hydrogel with well-defined molecular architecture and properties.^177^ They explained that the degree of substitution of the PNIPAM chains was solely controlled by a CuAAC reaction, while the RAFT polymerization controlled the molecular weight of PNIPAM to a narrow *Đ*. This control of the critical parameters allowed the optimization of the gel for regenerative medicine applications. In another study, a thermosensitive guar-based hydrogel was prepared through CuAAC coupling cross-linking of alkyne-functionalized guar chains and α,ω-diazido poly[(ethylene glycol)-*co*-(propylene glycol)] (PEG-*co*-PPG; Figure 30).^178^ It was demonstrated that both mechanical and swelling properties could be manipulated easily by changing the experimental parameters, like the CuAAC coupling temperature, mass fraction of the guar/cross-linker chains, and the guar molecular weight.

**Figure 31:**
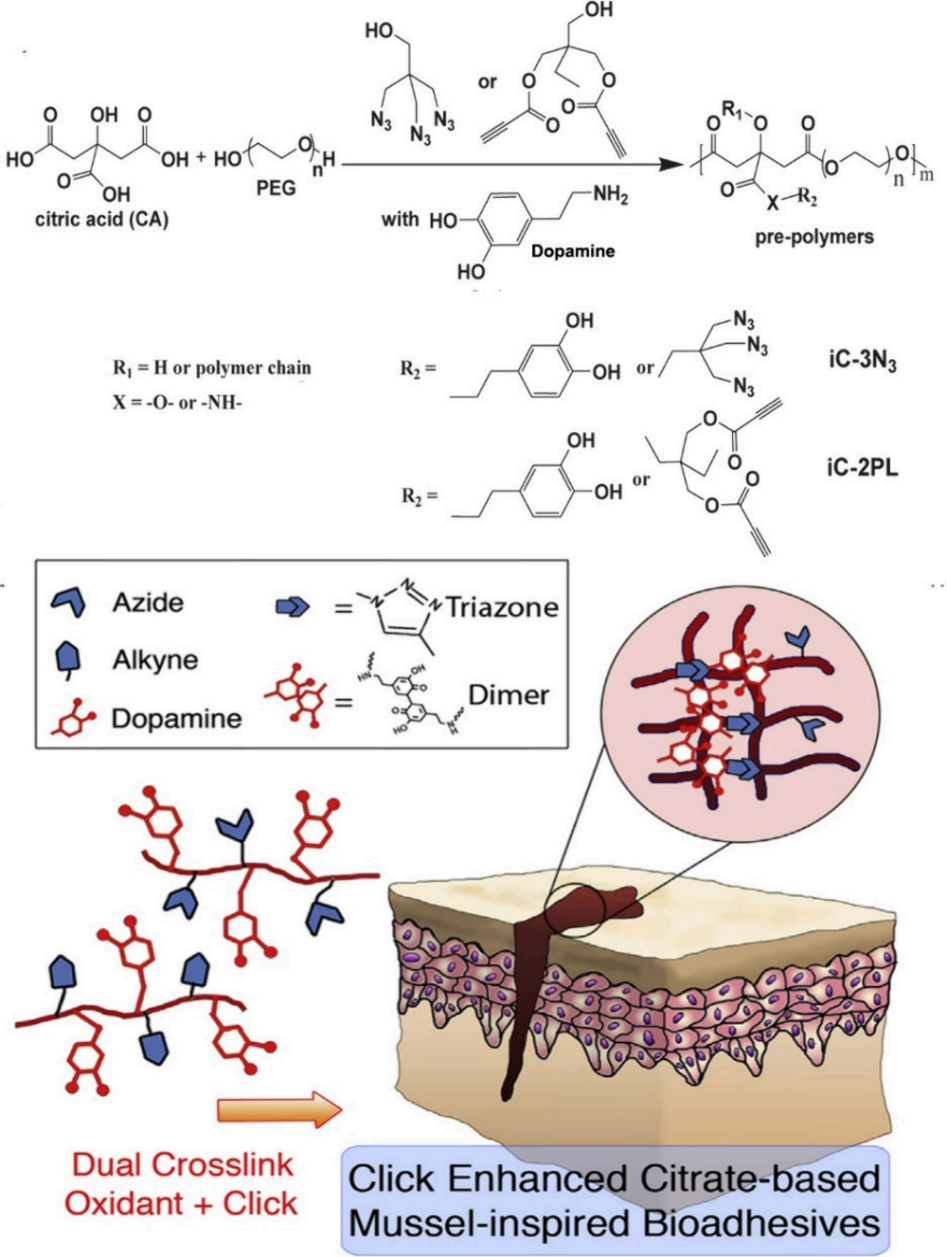
Synthesis of and schematic representation of dual cross-linked (oxidant and click (CuAAC)) hydrogels for forming antimicrobial bioadhesives. Adapted with permission from ref (179). Copyright 2017, Elsevier.^179^

Guo and co-workers reported injectable citrate-based mussel-inspired bioadhesives via a CuAAC reaction.^179^ These hydrogels were conducive to the adhesion and proliferation of human-derived mesenchymal stem cells (MSCs) (Figure 31). In detail, they synthesized azide- or alkyne-functionalized injectable citrate-based mussel-inspired bioadhesives (iC-3N_3_ or iC-2PL) by adding pentaerythritol triazide (3N_3_) or trimethylolethane dipropiolate (2PL) monomers to the side groups of iCs, respectively. In parallel, they prepared alkyne-functionalized gelatin and incorporated the iCs into it via CuAAC to further enhance the mechanical properties and biocompatibility of the hydrogel.

**Figure 32:**
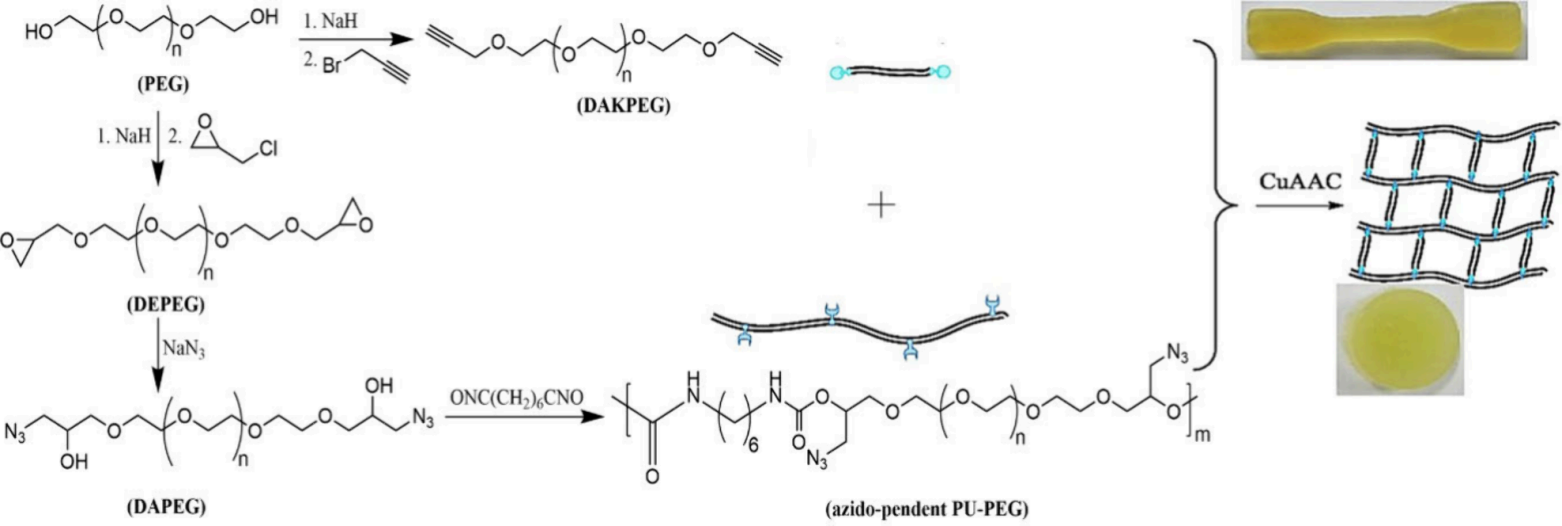
Synthesis of DAKPEG and azido-pendent PU-PEG, and preparation of PU-PEG hydrogels via thermally induced CuAAC. Adapted with permission from ref (180). Copyright 2018, Elsevier.^180^

Li et al. fabricated tough and thermoresponsive polyurethane-poly(ethylene glycol) (PU-PEG) hydrogels based on CuAAC-clicking azido-pendent PU-PEG and dialkynyl PEG (DAKPEG) (see Figure 32).^180^ The synthesized hydrogels not only demonstrated tunable gelation time and good mechanical properties, but also exhibited excellent biocompatibility with low toxicity to human fetal lung fibroblast WI-38 cell line.

Recently, Engkagul et al. prepared triple network hydrogels through CuAAC chemistry.^181^ To this aim, they first functionalized and batches of hyaluronic acid (HA) with either azide or terminal alkynes, and then CuAAC-reacted a mixture of these two compounds with chitosan (CS). This did then not only form triazole-coupled HA, but also yielded embedding of the CS by metal coordination of the Cu ions by the CS, and CS–HA charge-based complexation. Most importantly during the hydrogel formation, Cu (I) catalyst was generated from Cu(II) in the CS–Cu complex, which imparted excellent biocompatibility due to the minimal copper catalyst residue.

#### 2.3.2 Hydrogel Preparation via Metal-Free Click Chemistry

Despite the tremendous application of click chemistry in designing traditional and smart hydrogels, the toxicity of the Cu catalyst and the concomitant difficulty of implementing this approach in biological applications remain strong limitations.^182,183^ It is truly challenging to ascertain that sufficient copper catalyst has been detached from the gels even after rigorous extraction to reach levels that do not affect the outcomes of biological uses. Hence, alternative metal-free cross-linking approaches for the preparation of gels are increasingly used, including strain-promoted azide–alkyne cycloaddition (SPAAC), Michael addition, Diels–Alder, Schiff base, SuFEx click reaction, etc.^184,185^

##### 2.3.2.1 Strain-Promoted Azide–Alkyne Cycloaddition (SPAAC) Reactions

**Figure 33:**
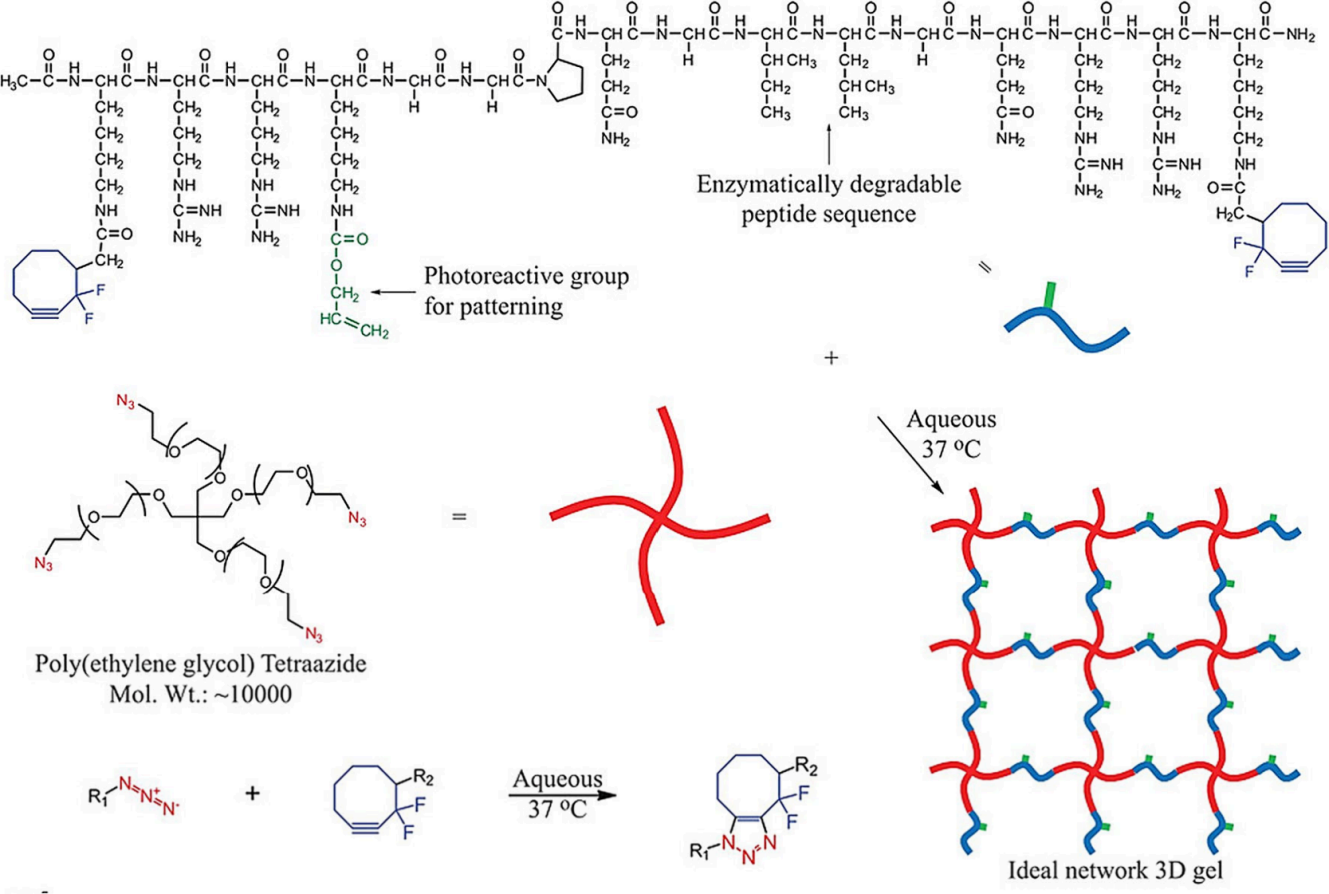
Click-functionalized macromolecular precursors undergo the SPAAC reaction to form a 3D ideal network hydrogel through a step-growth polymerization mechanism. Adapted with permission from ref (188). Copyright 2009 Nature.^188^

Like CuAAC, SPAAC has shown very high chemoselectivity and efficiency, even for *in vivo* applications, making it appropriate for *in situ* cross-linking.^186^ This Cu-free strategy was first explored in hydrogel formation by Turro et al. for the *in situ* cross-linking of an azide-functionalized photodegradable star polymer with linkers carrying two cyclooctynes.^187^ This strategy based on SPAAC proved its supremacy over CuAAC regarding better control upon gelation, and allowed the easy design of novel complex, functional, and biocompatible networks.^156,165^ Biocompatible hydrogels for direct *in situ* encapsulation of 3T3 fibroblasts cells in a clicked PEG-peptide network were achieved by the group of Anseth using a SPAAC click reaction of a highly functionalized polypeptide that bore an activated cyclooctyne moiety on either end, and a four-arm PEG carrying azide terminal groups (Figure 33).^188^ Subsequently, to obtain control over cell growth, alkene groups were added to the peptide backbone, so that thiol-functionalized Arg-Gly-Asp (RGD) tripeptides could be photopatterned onto the scaffold using the thiol–ene coupling reaction. The properties of the extracellular matrix were thus nicely imitated by this highly sophisticated, enzymatically degradable, material via the combination of two orthogonal and biocompatible click reactions.^189^

**Figure 34:**
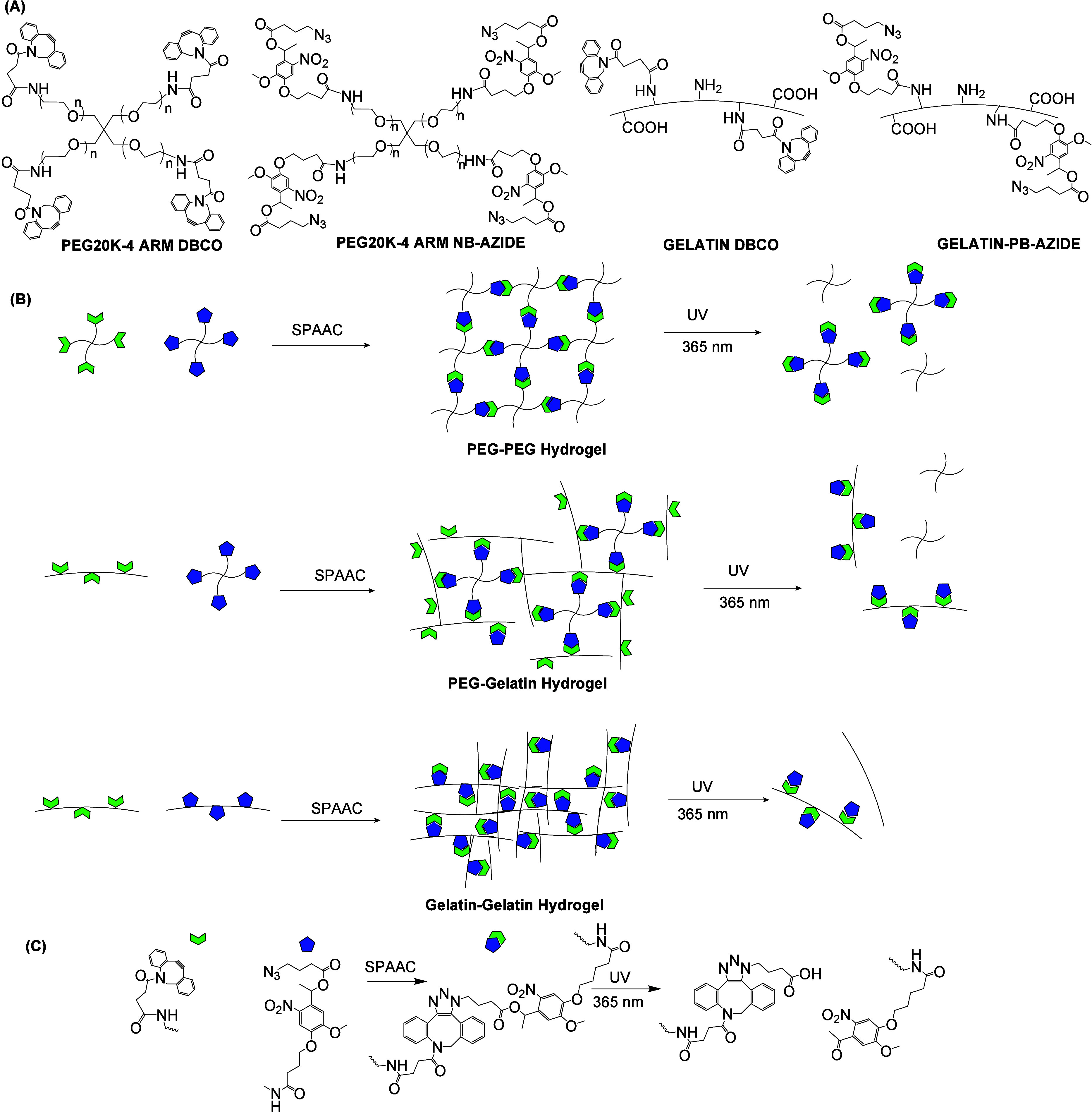
Reaction scheme for SPAAC chemistry, and representative scheme for the formation of PEG-gelatin hydrogels and UV-induced degradation. Adapted with permission from ref (190). Copyright 2015, ACS.^190^

Next to enzymatic degradability, also photodegradability is of interest for some hydrogel applications, such as light-induced drug release. With this in mind, Truong and co-workers produced photodegradable hydrogels using the SPAAC click reaction. To prepare the hydrogel, they first synthesized four different types of hydrogel precursor macromolecules, including a dibenzylcyclooctyne (DBCO)-modified tetra-arm PEG, DBCO-modified gelatin, azide-nitrobenzile modified PEG and azide-nitrobenzile modified gelatin. These syntheses were followed by a SPAAC reaction between DBCO and azide-nitrobenzile-modified PEG/gelatin macromolecules (Figure 34).^190^ The resulting photolabile *o*-nitrobenzyl (NB)-containing hydrogel displayed good cell viability, and provided on-demand, light-induced release of encapsulated fibroblasts due to the cleavage of the adjacent group of the nitrobenzyl moiety upon UV irradiation. Similar SPAAC chemistry was used by Zhong and co-workers to produce injectable HA-PEG hydrogels with tunable gelation times (5–50 min) by varying the azide and cyclooctyne ratio. They observed that, the resulting hydrogels display fast gelation, excellent mechanical strength, and slow degradation behavior, which is attributed to the high reaction rates of the SPAAC reaction,^191^ and the high cross-link density resulting from tetrasubstituted azide-PEG, and cross-linked amide bonds when reacts with Cyclooctyne-HA. The hydrogel might be beneficial in a variety of applications, such as injectable filler materials for cosmetic surgery, because to its good mechanical qualities and biocompatibility.^192^

##### 2.3.2.2 Michael Addition Reactions

**Figure 35:**
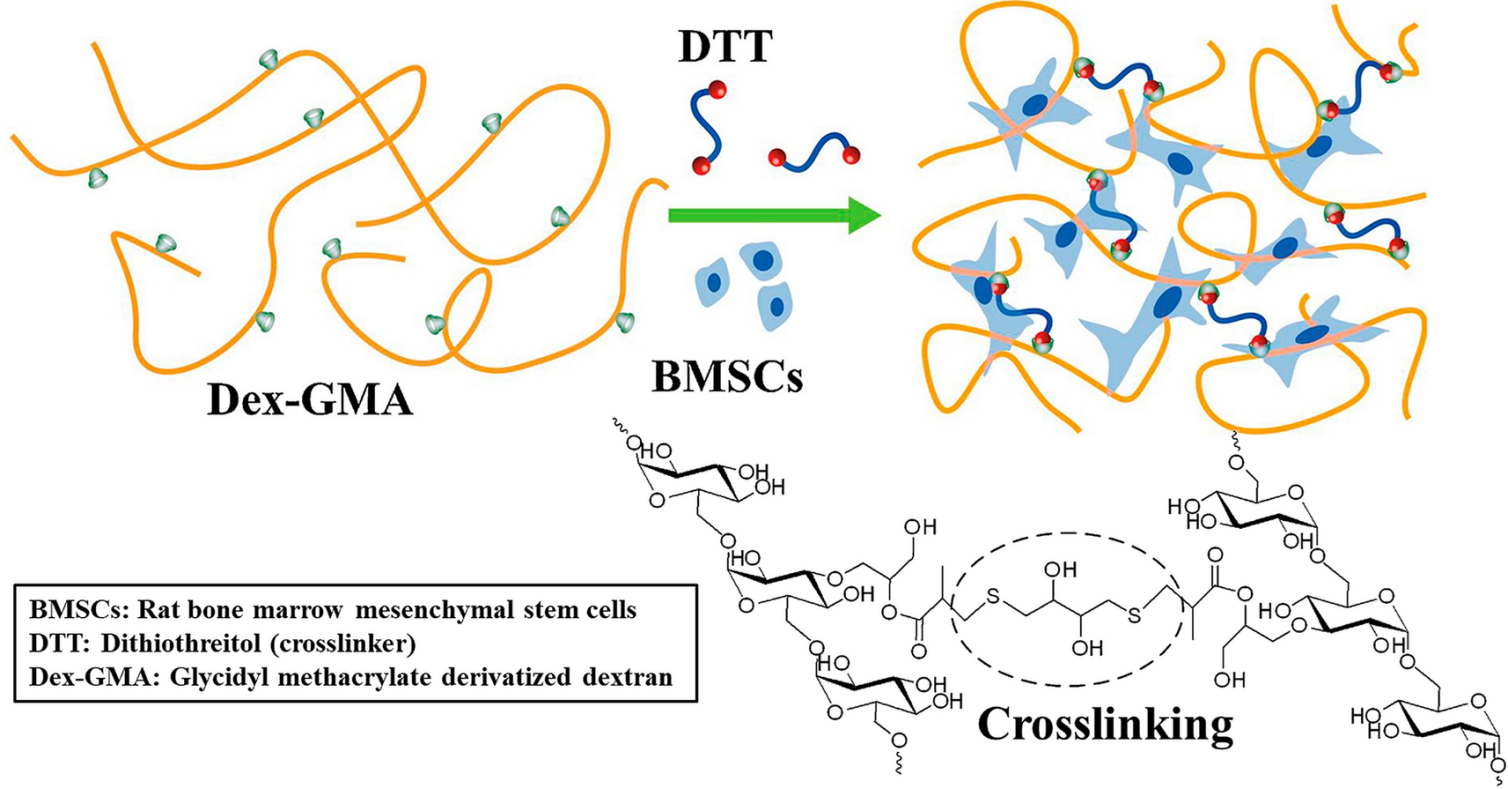
Schematic diagram of hydrogel formation after DTT addition to glycidyl-containing dextran. Adapted with permission from ref (197). Copyright 2015, Elsevier.^197^

The Michael addition to electron-poor alkenes is a great example of a metal-free click reaction that functions well even under congested conditions.^193^ Hubbel et al. were among the first to report this reaction for the successful preparation of cross-linked polymer gels using two aqueous solutions of PEG-dithiols and PEG-multiacrylates, respectively.^194^ They employed the resulting gels for protein drug delivery study, with albumin taken as a model. In this study, the albumin could be released in a controlled manner due to the very slow dissolution of the gel with zero-order kinetics. Similarly, Segura’s group used the Michael addition of thiols to vinyl sulfones (VSs) to promote a matrix metalloproteinase-degradable PEG-based hydrogel for the delivery of DNA/polyethylenimine (PEI) polyplexes to mesenchymal stem cells (MSCs).^195^ A new synthetic extracellular matrix for regenerative medicine applications was accomplished by Chawla et al.,^196^ who employed the Michael addition of cysteine-functionalized and VS-functionalized saccharide-peptide polymers for the encapsulation and 3D culture of MSCs. The elegance of this study was that it initiated the formation of biodegradable synthetic hydrogels primarily composed of natural building blocks, without large synthetic segments, by Michael-type addition. This saccharide-peptide hydrogel acted as a superior bioabsorbable metabolite and offers natural biodegradability and no cytotoxicity due to its natural composition. Analogously, Liu et al. reported the formation of pH-dependent hydrogels for cell encapsulation using cross-linking thiol-Michael-type additions under physiological conditions of a glycidyl methacrylate-derivatized dextran backbone to dithiothreitol (DTT) as shown in Figure 35. pH-dependent gelation times, mechanical properties, and the swelling behavior of the hydrogel were also reported.^197^

Samanta et al. reported thiol-modified collagen and an 8-arm PEG with maleimide moieties for the preparation of an injectable shape-holding hydrogel.^198^ Again, hinging on Michael additions, the resulting hydrogel displayed self-healing, shear-thinning properties, and a low swelling ratio. After a four-day 3D culture period, the hydrogel culture system showed sufficient nutrient and oxygen diffusion for encapsulated MSCs and human umbilical vein endothelial cells (HUVEC), showing the great potential of such easy-to-make materials. Recently, Liu and co-workers designed a thiol–ene photoclickable PEG hydrogel by reacting norbornene terminated 4-arm PEG with a complex of hydrophobic drug ellagic acid (EA) with mono-(6-mercapto-6-deoxy)-β-cyclodextrin (SH-β-CD).^199^ This strategy significantly enhanced the loading capacity of the hydrogel for EA, and endowed the hydrogel with multifunctional properties. Moreover to tailor the mechanical stiffness of the hydrogel, they introduced cross-links-forming dithiothreitol, so that the resulting materials can, e.g., be used for mechanically more demanding applications like wound dressings.

##### 2.3.2.3 Diels–Alder Cycloaddition Reactions

Diels–Alder (DA) reactions may offer high-temperature reversibility, which is convenient in designing self-healing products.^200^ In a pioneering work, Saegusa and co-workers reported the synthesis of hydrogels using the DA reaction between furan and maleimide end-functionalized poly(*N*-acetylethylenimine).^201^ This approach was later utilized by Wei and co-workers for the preparation of hydrogels from furan-functionalized dimethylacrylamide and bismaleimide-PEG under aqueous conditions.^202^ These hydrogels were precisely designed to serve the role of smart injectable material, as they were stable in water with a gelation time that was strongly dependent on temperature. In another work, a hyaluronic acid (HA) hydrogel was obtained by Nimmo et al., by a one-step, aqueous Diels–Alder reaction between furan-functionalized poly-HA and dimaleimide-functionalized PEG.^203^ By tuning the furan to maleimide molar ratio, both the mechanical and degradation properties of the subsequent Diels–Alder hydrogels could be controlled. Similar Diels–Alder chemistry was used recently by Guaresti et al. to yield a stimuli-responsive chitosan-based hydrogel using furan-modified chitosan and a bismaleimide cross-linker.^204^ It was found that the swelling degree of the chitosan hydrogel was strongly dependent on the cross-linking density of bismaleimide group and pH of the medium (25% reduction of swelling value in an acidic medium). Another example of Diels–Alder reactions uses 1,2,4-triazoline-3,5-dione (TAD), which makes the cycloaddition in many cases considerably faster than found in the conventional maleimide-based DA reactions, finishing on a time scale of seconds at ambient temperature,^205^ with high product yield and without use of any catalyst or stimuli. Based on such chemistry, Mondal et al.^206^ developed a novel type of healable fluorescent polymer material using the clicking of anthracene and TAD. In this case, RAFT polymerization was used to create copolymers of 2-hydroxyethyl methacrylate (HEMA) and methyl methacrylate (MMA). The anthracyl derivatives were used to connect the methacrylate copolymers with repeated pendant hydroxyl groups, where the aromatic groups served as the TAD moiety’s “click” partner. Other than these, DA click reactions also successfully employed to synthesize an interpenetrating network (IPN) hydrogel. For example, Wei et al. used Diels–Alder click reactions to design a starch-cellulose-based hydrogel.^207^ To prepare the hydrogel, first they functionalized starch with *N*-maleoyl-β-alanine, and in parallel, decorated cellulose with furfurylamide. Second, they dissolved this functionalized starch and furfurylamide-modified cellulose, together with a polymerizable maleic anhydride-modified β-cyclodextrin, methacrylic anhydride-modified hydroxyethyl starch (cross-linker) and acrylamide in water, and heated to 50 °C for 3 h, which resulted in the formation of the first network via the Diels–Alder reaction. Subsequenly, the second network was formed by photopolymerization of the acrylamide and polymerizable maleic anhydride modified β-cyclodextrin that were now embedded in the first network. The prepared IPN hydrogel possessed a good porous interconnected network structure, and its swelling ratio was lower than that of the two single network hydrogels, additionally with better sustained release of 5-fluorouracil. In another very recent study, Wilson and his team also exploited a pericyclic photoclick reaction for the patterning of a hydrogel for spatially controlled cell behaviors.^208^ The synthesis of this hydrogel involved maleimide-bearing cyclopentadienone–norbornadiene (CPD-NBD) and cysteine as naturally present in extracellular matrix protein, where in the presence of 365 nm UV exposure patterning can be obtained. Moreover, the resulting hydrogel allowed for the precise spatial regulation of extracellular-signal regulated kinase (ERK) activity and cell migration in mammalian cells, while demonstrating programmable cell behavior through patterned chemical modification.

##### 2.3.2.4 Oxime/Hydrazone Click Reactions (Schiff-Base Reactions)

Hydrogels can also be produced at physiological temperature by oxime and hydrazone-based (or Schiff-base) click reactions. In Schiff base reactions, a dynamic covalent bond is formed via the rapid equilibrium between Schiff-base linkages and the aldehyde and amine reactants.^209^ Due to the uncoupling and recoupling of the imine linkage, the resulting hydrogel networks are self-healing in nature, which significantly increase the potential of hydrogels in the field of biomedical applications. The rapid equilibration also addresses another point. In 2009, Kiser et al. observed that the then-known fastest gels cross-link too slowly to be applied in most *in situ* applications.^210^ It is possible to improve the gelation kinetics by adapting cross-linking moieties (e.g., alkynes with increased electron deficiency, and azides with increased electron density for the CuAAC reaction) and consequently the reaction rate, but often at the cost of increased complexity of products and/or reaction conditions. The rapidity of the Schiff-base reaction addresses this point. In 2014, Sarker et al. produced an alginate-gelatin composite using a Schiff-base reaction route between aldehyde moieties created in oxidized alginate and amino groups present in gelatin.^211^

**Figure 36:**
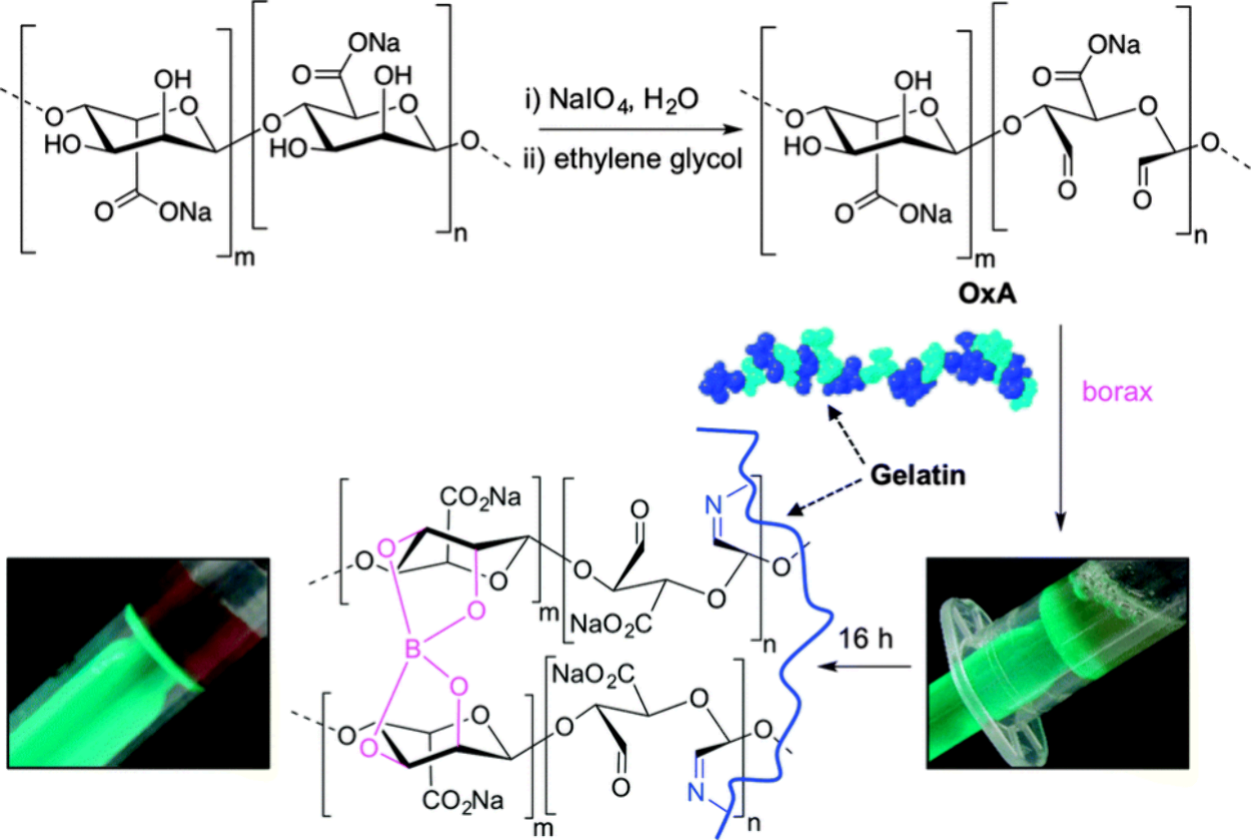
Schematic representation of the cross-linking of oxidized alginate (OxA) and gelatin in the presence of borax. Inset pictures show the darkening and immobilization of the starting solution during the sol-to-gel transition. Adapted with permission from ref (209). Copyright 2017, RSC.^209^

Pettignano and co-workers fabricated a similar, dual-cross-linked biohydrogel (OxA-GB) from gelatin type B (GB) and oxidized alginate (OxA) in the presence of the borax, B(OH)_4_ (Figure 36).^209^ In addition to the Schiff base linkages that can be formed, the borax can bind to two hydroxyl groups in a chain, and then cross-link via a similar interaction to two −OH moieties in an other polymer chain, and these two cross-linking reactions yield a visible sol-to-gel transition. The pH of the medium plays a critical role in the self-healing capability of the hydrogel, validating that it is indeed these dynamic covalent bonds that yield the autorepair of damaged hydrogel interfaces. The activation by relatively mild oxidation was also used by Yin et al. to develop a mussel-inspired injectable hydrogel based on self-cross-linking of hydrazide-modified poly(l-glutamic acid) and dual-functionalized alginate (bearing both *ortho*-quinone and aldehyde moieties) with excellent self-healing properties, strong bioadhesion, and low cytotoxicity.^212^ The self-healing property of the oxidized hydrogels was primarily regulated by the fast intermolecular covalent cross-linking between the surface of the gelatin and the highly reactive quinone groups.

##### 2.3.2.5 Sulfur(VI) Fluoride Exchange (SuFEx) Click Reaction

**Figure 37:**
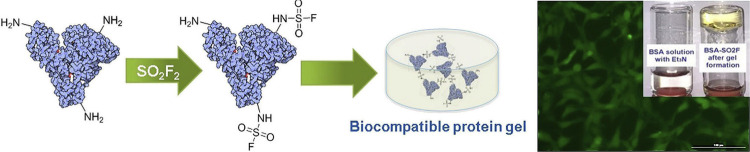
SuFEx modification of a model protein with BSA. Adapted with permission from ref (217). Copyright 2016, Elsevier.^217^

The sulfur(VI) fluoride exchange (SuFEx) reaction between an amine (R-NH_2_) or sulfonyl fluoride (R-SO_2_F) and an aryl silyl ether (Ar-OSiR_3_) is the latest addition in metal-free click chemistry for the improvement in bioconjugation chemistry and drug discovery.^213−215^ In several bio-SuFEx chemistries, a sulfonamide is formed by a coupling reaction between sulfonyl fluoride with aliphatic amines, and the high stability of the sulfonamide linkage meets the expectation of the required driving force to generate a click reaction between two functional units.^216^ In 2016, Averick and co-workers were the first to report SuFEx click chemistry for the modification of biomolecules, for the modification of a model protein, bovine serum albumin (BSA), with sulfuryl fluoride, SO_2_F_2_, to produce BSA-SO_2_F.^217^ Due to the unique and selective reactivity of the −SO_2_F group with amines, BSA-SO_2_F could be self-condensed to form a biocompatible hydrogel that was used in coculture with HEK 293 cells. Analogously, PEG that was functionalized with −OSO_2_F groups could also react with the amine group of BSA under various pH conditions (Figure 37).

**Figure 38:**
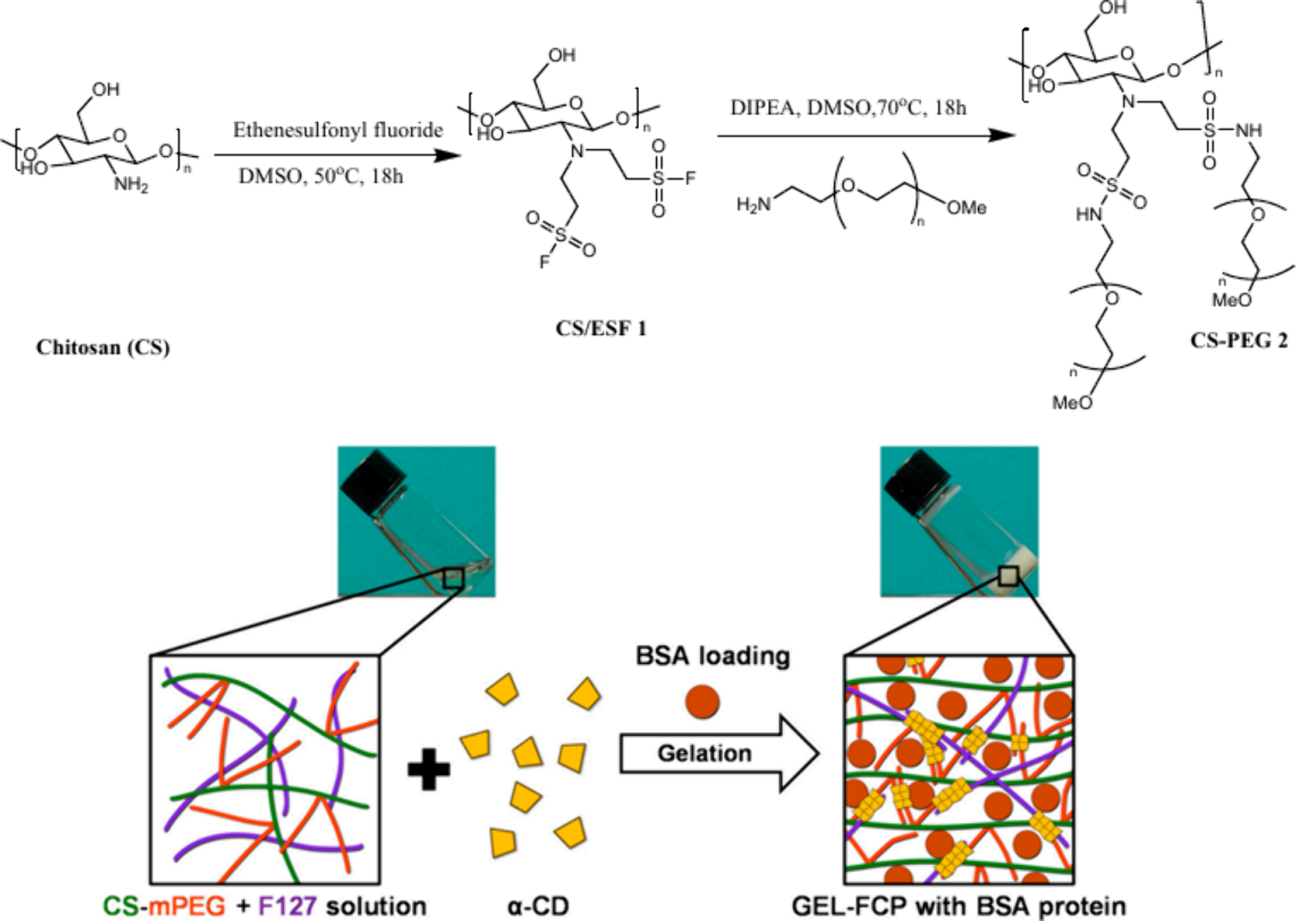
Synthetic route toward chitosan-based hydrogels via SuFEx chemistry and specific supramolecular interactions between PEG chains and pluronic F127 with a-CD. Adopted with permission from ref (218). Copyright 2021 MDPI.^218^

Recently, Jang et al. synthesized supramolecular hydrogels by a host–guest reaction,^218^ where chitosan (CS) derivatives, i.e., CS-mPEG, were fabricated in two steps: First ethene sulfonyl fluoride was smoothly reacted with amines, a reaction that quickly proceeds in a quantitative fashion,^193^ to yield −SO_2_F functionalized CS. Next, by S–N bond-forming SuFEx click chemistry under mild conditions chitosan-mPEG could be formed. This was then mixed with Pluronic-F127 (CS-mPEG/F127) and cyclodextrin (a-CD) to produce the final hydrogel.^218^ Here, the CS-mPEG/F127 acts as a guest whereas a-CD acts as a host. The authors observed that the reological properties, gelation time, mechanical properties and shear viscosity could be easily tuned by simply altering the ratio of CS-mPEG and F127 (see Figure 38).

##### 2.3.2.6 Other Metal-Free Click Reactions

Apart from the above-mentioned strategies, there are a few other metal-free click reactions—such as inverse electron demand Diels–Alder (IEDDA),^219^ strain-promoted oxidation-controlled cycloadditions of *ortho*-quinones with cyclooctynes^220^ or cyclopropenes,^221^ and the amine-yne reaction^222^ are also worth mentioning in the hydrogel preparation. There is an increasing trend of using these bio-orthogonal click chemistries for the development of complex hydrogel materials which provide dynamic, cell instructive microenvironments. Unambiguously, IEDDA click reactions between electron-rich dienophiles (such as norbornene and trans-cyclooctene) and s-tetrazines gain significant attention because of their high rate and bioorthogonality. Moreover, this alternative synthetic approach also possess similar kinds of benefits like SPAAC, but via simpler synthetic routes.^223^ These click reactions act as powerful bioorthogonal chemistries for the formation of block copolymers without involving any extra additives, catalysts, or initiators, which suggest that these techniques could also be employed for the formation of covalently cross-linked polymer networks. For example, in 2013, Alge et al. demonstrated the fabrication of cell-laden hydrogels using a tetrazine-norbornene based IEDDA reaction.^224^ They observed that the IEDDA reaction between a multifunctional PEG-tetrazine macromer with a dinorbornene peptide produced a hydrogel within minutes and could be employed for sequential modification of the network via thiol–ene photochemistry. This strategy could be beneficial for the development of cell-instructive hydrogels for tissue engineering applications. Alge and co-workers also demonstrated that PEG hydrogels cross-linked with tetrazine-norbornene were far better than thiol–norbornene click chemistry in terms of storage modulus values and resistance to hydrolytic degradation.^225^

**Figure 39:**
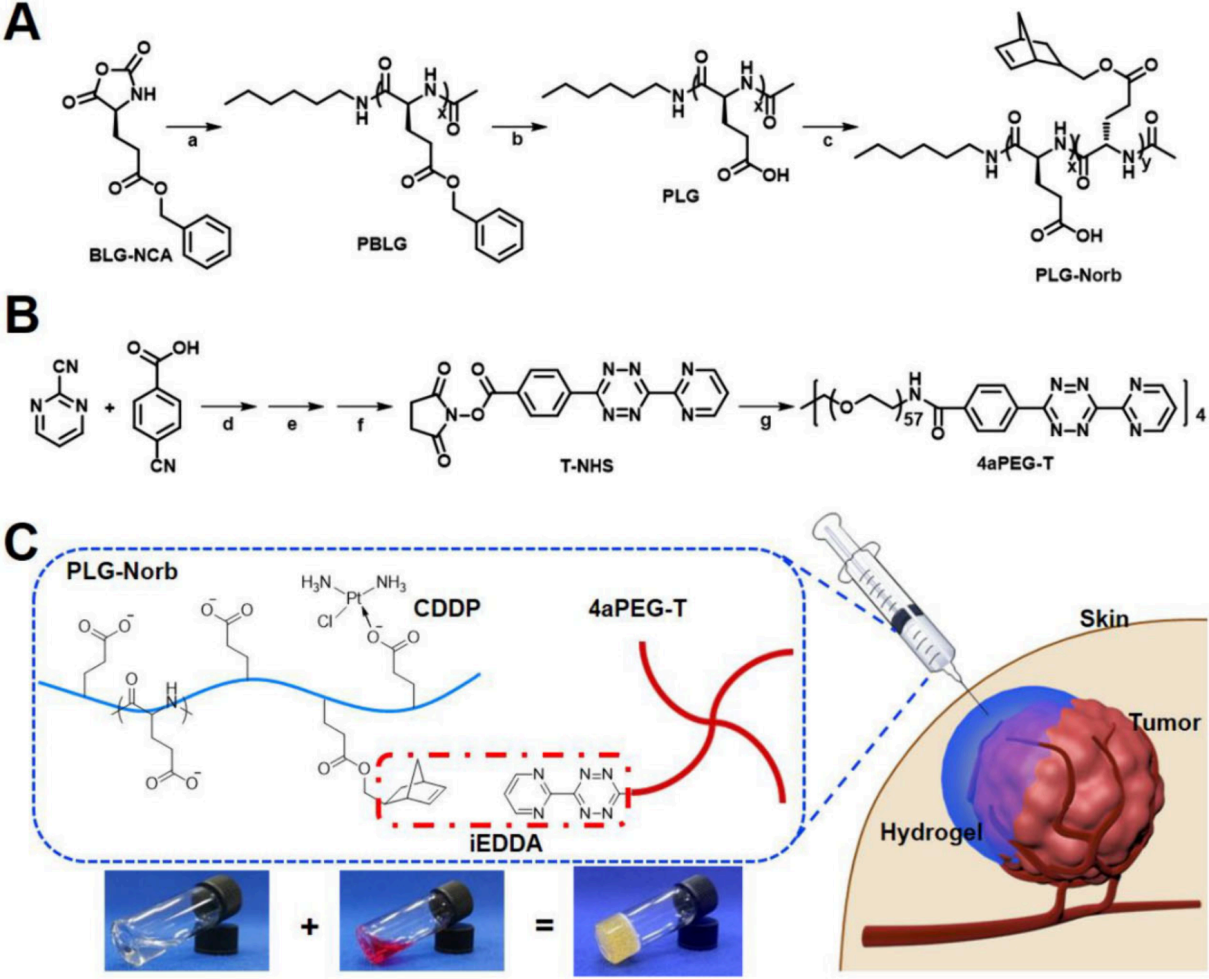
(A, B) Synthetic routes of norbornene-modified poly(l-glutamic acid) (PLG-Norb) and four-arm PEG (4aPEG-T). (C) Schematic illustration of injectable click polypeptide hydrogels for local treatment of tumor. Adapted with permission from ref (226). Copyright 2020, MDPI.^226^

Recently, Zhang et al. reported an injectable polypeptide hydrogel by an IEDDA reaction between norbornene-modified poly(l-glutamic acid) (PLG-Norb) and a tetrazine-functionalized four-arm PEG (4aPEG-T) for cisplatin drug delivery.^226^ This bioorthogonal click reaction again produced the hydrogel within only a few minutes. The carboxyl groups of PLG-Norb could form a stable polymer–metal complex with cisplatin drugs, and controlled the drug release phenomena (Figure 39).

**Figure 40:**
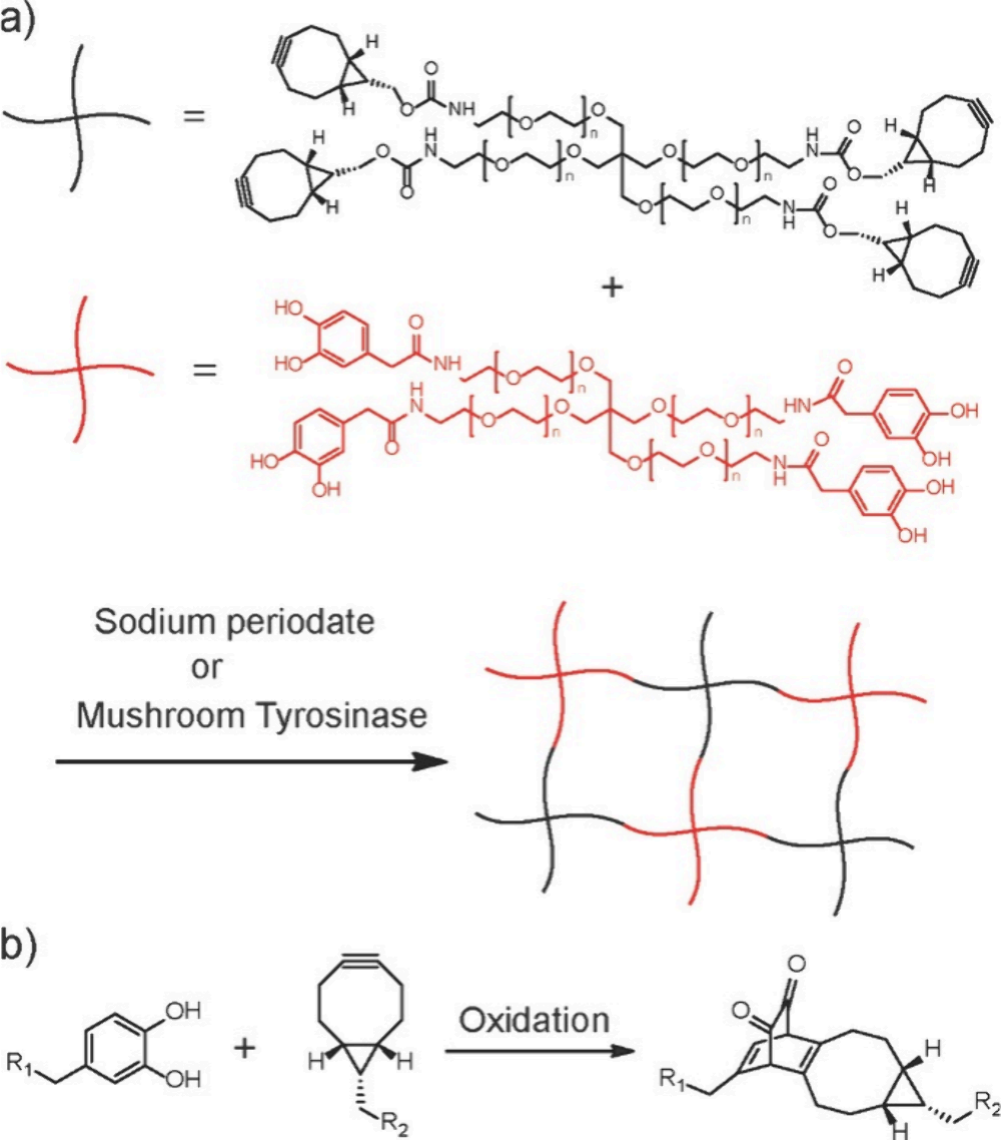
Hydrogel formation using the SPOCQ reaction. a) A mixture of star-PEG-BCN and star-PEG-DHPA is oxidized with sodium periodate or mushroom tyrosinase, to yield a stable hydrogel network; b) SPOCQ reaction between BCN and DHPA upon oxidization. Adapted with permission from ref (220). Copyright 2015, Wiley.^220^

Analogously to IEDDA, strain-promoted oxidation-controlled cyclooctyne-1,2-quinone cycloaddition (SPOCQ) reactions are also emerging as a new metal-free click reaction to produce complex hydrogel materials,^220^ for example for the formation of hydrogels out of four-armed PEG functionalized with cyclooctyne derivative bicyclo[6.1.0]non-4-yne (BCN) and catechol DHPA (3,4-dihydroxyphenylacetic acid). This reaction proceeds quickly after oxidation of the catechol to the *ortho*-quinone,^227^ which can be triggered both chemically and enzymatically. Furthermore, both SPOCQ and SPAAC reaction can be used in concert, due to the large difference in reaction rates which can act as an efficient route for hydrogel synthesis (see Figure 40).^220^

## 3 Biomedical Applications of Functional Polymers Synthesized by Click Chemistry

### 3.1 Drug Delivery

During the last two decades, localized and targeted drug delivery systems have emerged as a prime goal for pharmaceutical scientists to get maximum clinical efficiency due to their minimum side effects compared to conventional drug delivery systems. In this context, click chemistry provide an efficient platform to develop novel targeted drug delivery systems due to its mild reaction conditions and biofriendly nature. For example, a literature search in “PubMed” on March 2024 using the keywords “click chemistry” and “drug delivery” revealed a total of 589 publications, which include research articles, letters, and reviews, which signifies the importance of click chemistry to develop new drug delivery systems. So, in this section of the review, we discuss the main lines in click chemistry to develop such novel polymeric materials. Here we do not only highlight their chemical features, but also their utility toward drug delivery applications *in vitro* as well as *in vivo*, which include preparation and functionalization, and its use so far in therapy.

#### 3.1.1 Polymeric Micelles for Drug Delivery Applications

Polymeric micelles (PMs) are drug carriers that are formed by self-assembly of amphiphilic block copolymers, usually in the nano/micromolar range, in aqueous solutions. They are generally characterized by a core–shell structure with a hydrophobic core and hydrophilic shell. This core can facilitate taking up of hydrophobic drugs, while the hydrophilic shell ensures low protein adsorption, enhanced drug solubility and prolonged circulation time within the human body. Due to the small size and minimized protein adsorption of PMs, they are generally stealth in nature and selectively accumulate at disease sites (such as at tumor tissues) due to the enhanced permeability and retention (EPR) effect.^228^ Various click reactions, mostly CuAAC and the Diels–Alder reaction of maleimides and furan moieties, have been used for the synthesis of amphiphilic block copolymers that can self-assemble into a micellar structure or into functionalized polymeric micelles.

One of the first examples was presented by Sumerlin and co-workers, who developed folate-conjugated thermoresponsive block copolymers that can self-assemble into PMs.^229^ To this aim, they synthesized a thermoresponsive azido-terminated poly(*N*,*N*-dimethylacrylamide)-*b*-poly(*N*-isopropylacrylamide) (N_3_-PDMA-*b*-PNIPAM) by RAFT polymerization using an azido-functionalized chain transfer agent, *N*-isopropylacrylamide (NIPAM) and *N*,*N*-dimethylacrylamide (DMA). This was subsequently decorated with propargyl folate by a CuAAC reaction. The resultant block copolymer can undergo self-assembly, to provide a polymeric micellar structure with an average diameter of ∼46 nm at 34 °C. A similar type of approach was also taken by the Du^230^ and Stenzel^231^ research groups, who independently synthesized block copolymers with pendent azide/alkene in one block of the polymer which was then functionalized with carbohydrates using either a CuAAC or thiol–ene click reaction. While these three groups proposed that their systems can be used as a drug delivery system, no *in vitro* or *in vivo* biological experiments were then reported to show their efficacy.

This changed with the work of Dong and co-workers, who used the CuAAC reaction to obtain PEG-based dendritic block copolymer micellar nanostructures for drug delivery applications.^232−234^ In this manner they, for instance, developed spherical flower-like micellar self-assembled structures of PCL-*b*-PEG-*b*-PCL.^232^ The triblock copolymer was shown to be capable of encapsulating the hydrophobic anticancer drug doxorubicin (DOX) with high efficiency, and to release it in a sustained manner at acidic pH, such as characteristically found in many tumor microenvironments.

**Figure 41:**
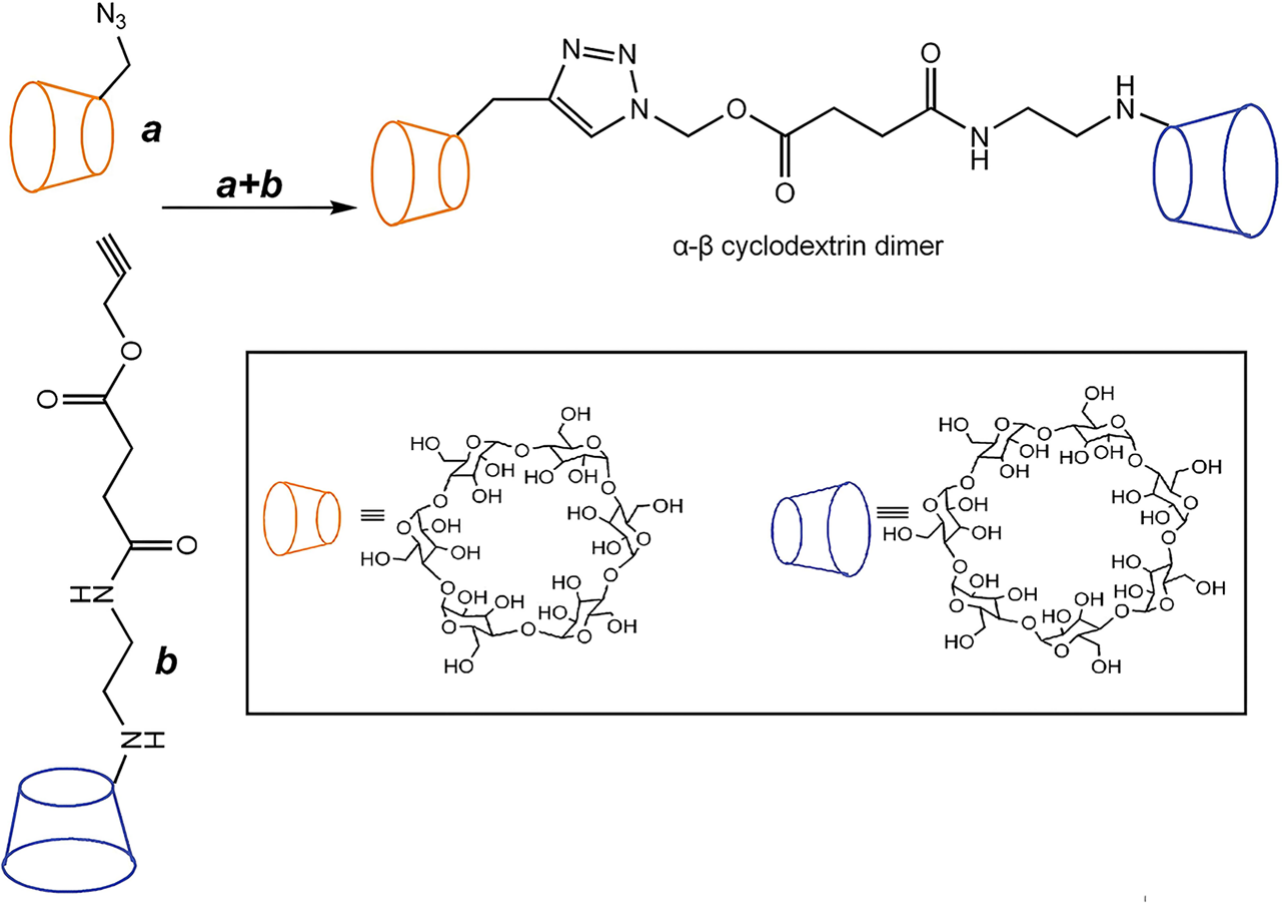
Synthesis of the α–β cyclodextrin dimer. Adapted with permission from ref (236). Copyright 2010, ACS.^236^

Soliman et al. designed an A_2_B type (A = PEG; B = PCL) of miktoarm star polymers using the CuAAC click reaction for the delivery of nimodipine (NIM), a hydrophobic drug used in the prevention and treatment of delayed ischemic neurological disorders.^235^ The micelles obtained from the star polymer can encapsulate NIM as high as 78 weight% at a feed weight ratio of 5.0%. This effectively enhanced the solubility of NIM by ∼200 fold. The *in vitro* release of NIM from the micelle takes place in a much more gradual manner than from its solution counterpart, with evident biological advantages. They observed, for example, that, in the presence of micelle-encapsulated NIM, as well as micelles alone, lipopolysaccharide-induced nitric oxide generation in N9 microglia cells was reduced drastically. Additionally, the treatment of microglia with micelle-encapsulated NIM reduced the release of tumor necrosis factor-alpha (TNF-α), a pro-inflammatory cytokine. This suggests that NIM-loaded miktoarm micelles can be promising drug delivery carriers for the treatment of neuroinflammation. Zhang and co-workers have also used CuAAC to synthesize core–shell nanosized micelles with tumor-triggered targeting properties.^236^ For their synthesis, they used an azido-modified α-cyclodextrin (CD), which acted as a host. The guest was a phenyl group-terminated hydrophilic polymer poly(NIPAM-*co*-*N*-acryloxysuccinimide) that was coupled using CuAAC to an alkynated β-CD, designed to complex hydrophobic PCL bearing a terminal adamantyl moiety. The CD dimer was successfully employed to link hydrophilic and hydrophobic moiety which subsequently assembled into the micellar structure within an aqueous medium (Figure 41). To enhance the cellular uptake efficiency of the nanomicelle, they functionalized the micelle with an Arg-Gly-Asp (RGD) peptide as targeting ligands toward tumor tissue. In addition to that, PEGylation via pH-sensitive imine bonds was also done to protect peptide ligands in normal cells. An *in vitro* cell viability (Hela cells) study in the presence of anticancer drug DOX revealed that the drug-loaded nanomicelles are highly effective to kill cancer cells with low doses (74% dead cells using 1 mg/mL concentration at 39 °C), which suggest its usability for drug delivery applications.

Other than the synthesis of polymeric micelles, the CuAAC reaction is also used to functionalize PMs for specialized drug delivery applications. For instance, Lutz and co-workers reported an *in situ* functionalization strategy of thermoresponsive PMs.^237^ Their polymer was composed of a terminal hydrophobic cholesterol moiety and a hydrophilic polymer chain segment of oligo(ethylene glycol) (meth)acrylates initiated by cholesteryl-2-bromoisobutyrate. After the ATRP polymerization, the bromine chain ends of the surfactants were converted into an azide, and subsequently reacted with propargyl alcohol by CuAAC. Depending on their hydrophilic–hydrophobic balance, these novel surfactants led to different types of structures in an aqueous medium, which can be used for drug delivery purposes. Shoichet and her group members have designed peptide-functionalized PMs using CuAAC as a surface decoration tool.^238^ The polymer consists of a hydrophobic poly(2-methyl-2-carboxytrimethylene carbonate-*co*-d,l-lactide) [P(TMCC-*co*-LA)] and a hydrophilic PEG-N_3_. These terminal azide groups further reacted with alkyne-modified KGRGDS peptides via an aqueous CuAAC reaction to form peptide-modified micelles. Surfaces with such RGD-modified nanomicelles displayed more than a 6-fold increase in their *in vitro* binding affinity toward rabbit corneal epithelial cells: average number of cells per well is 1446 ± 377, which was significantly higher than negative control, i.e., when the plate lacks GRGDS peptide, which displayed 222 ± 70 cells per well. Additionally, these nanomicelles were just small enough (439 ± 11 nm) for sterile filtration and may, for example, be useful for the targeted delivery of drugs to injured eyes.

**Figure 42:**
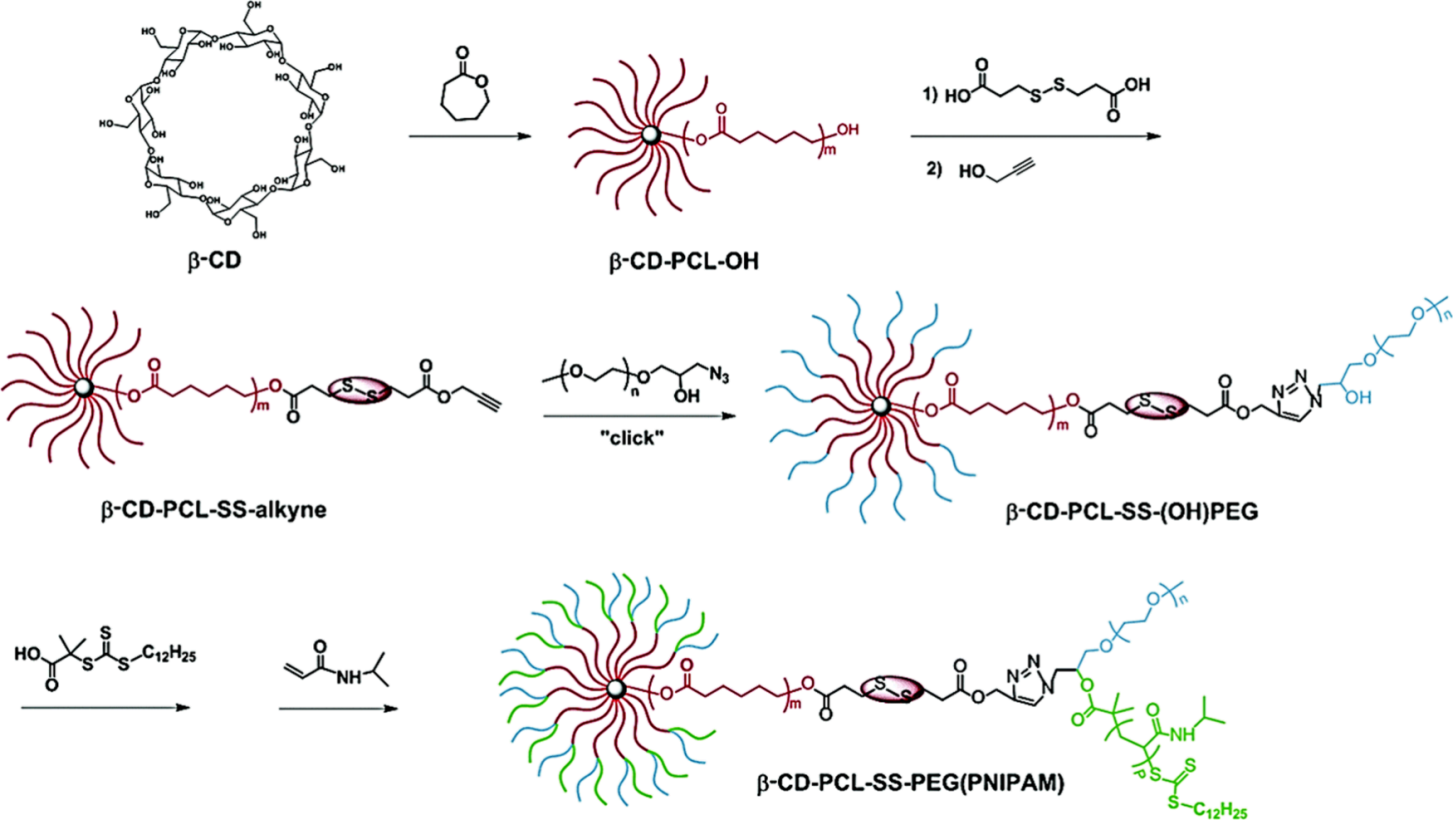
Schematic illustration and synthetic route of the star-like amphiphilic CD-PCL-SS-PEG(PNIPAM) copolymer. Adapted with permission from ref (239). Copyright 2017, RSC.^239^

Wu and co-workers also designed a “Y” shape amphiphilic dual-responsive block copolymer for controlled drug delivery.^239^ For the synthesis of block copolymers, they first decorated β-cyclodextrin (β-CD) with polycaprolactam chains. Esterification of the terminal alcohol with a S–S bond-containing terminal alkyne yielded an alkyne-functionalized material that could react with an azide-modified PEG that has one free −OH moiety using CuAAC click chemistry to get the star-like copolymer named (CD-PCL-SS-(OH)PEG). Furthermore, they performed an esterification reaction between these −OH and a RAFT agent, followed by RAFT polymerization of NIPAM to obtain the desired star-like copolymer (CD-PCL-SS-PEG-PNIPAM; Figure 42). Dynamic light scattering studies revealed that the average size of the CD-PCL-SS-PEG-PNIPAM polymeric micelles ranged in between 103 ± 12 nm with a unimodal distribution. Because of the presence of both the S–S bond and PNIPAM component, the polymer facilitated both redox (glutathione, GSH) and temperature responsiveness, and the resulting micelles showed sustained drug release profiles. More specifically, they found that, presence of 10 mM GSH led to a substantial increase in drug release from ∼40% at 25 °C to 98.8% at 37 °C after 24 h, indicating the significance of using temperature and redox environments as triggers in this dual-sensitive micelle system. Cellular uptake and an *in vitro* stimuli-responsive intracellular doxorubicin (DOX) release studies confirmed a high cellular uptake efficiency and significant intracellular drug release from DOX-loaded CD-PCL-SS–PEG-PNIPAM micelles. The combination of a temperature sensitivity (induced by the PNIPAM) responsiveness to the cellular reduction of S–S bonds, and a high cytocompatibility with both HepG2 and LO2 cells (>90% viability at concentrations up to 200 μg/mL), indicates the potential use of such micelles for controlled drug delivery applications.

**Figure 43:**
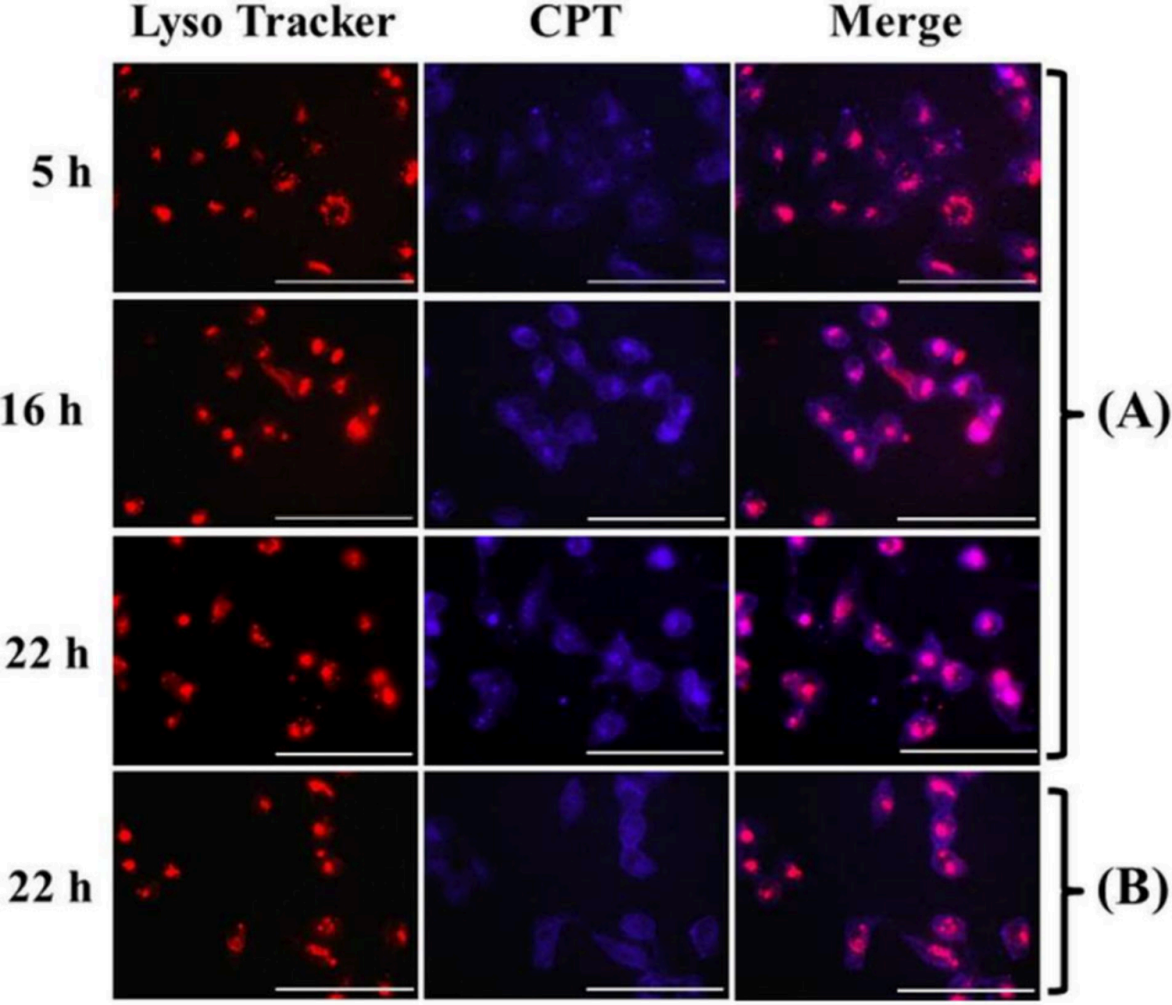
Cellular uptake behavior of (A) P(EAEP-PPA)_13.6_-*g*-ss-CPT_1.5_ micelles following different incubation times in HepG2 cells and (B) free CPT in HepG2 cells. The dosage of CPT was 0.836 mg/Liter. For each panel, images from left to right show cell cytoplasm stained by Lyso-Tracker Red, CPT fluorescence in cells (blue) and overlays of two images. All the images were observed by fluorescence microscopy and the scale bars correspond to 100 μm in all the images. Adapted with permission from ref (240). Copyright 2017, ACS.^240^

In the same year, Ni and co-workers reported poly(phosphoester-*co*-propynylamine) P(EAEP-PPA) functionalized with an anticancer camptothecin (CPT) derivative containing disulfide bonds for targeted delivery of CPT to cancer cells (CPT ∼ ∼S–S ∼ ∼N_3_).^240^ CuAAC was used to attach this CPT moiety to the terminal alkynes in P(EAEP-PPA), while the S–S bonds made the polymer sensitive to reduction by glutathione, which is found in abundant amounts in cancerous cells. MTT assays were used as an indication that P(EAEP-PPA) is cytocompatible for normal (L929) cells as well as cancer (4T1, HepG2) cells (maintaining viability ∼90% at concentrations up to 200 μg/mL). The DLS results confirmed that, these micelles are around ∼141 nm in size and relatively stable under physiological conditions, but could be rapidly disintegraded in the presence of 10 mM GSH medium. About 90% of CPT was released from the prodrug micelles in the reductive medium at 37 °C after 105 h, which indicates that the CPT-modified P(EAEP-PPA) polymeric prodrug micelles can efficiently release CPT into cancer cells to inhibit the cell proliferation (IC_50_ value of prodrug ∼4.5 mg/Liter and ∼0.5 mg/Liter for 4T1 and HepG2 cells respectively). Therefore, it is nicely established that the polyphosphoester-based prodrug can be used for triggered drug delivery system in cancer treatment. Additionally, they observed that the CPT-loaded micelles enter into cells through endocytosis and avoid lysozomal routes, whereas in the case of free CPT, it follows diffusion (as shown in Figure 43).

**Figure 44:**
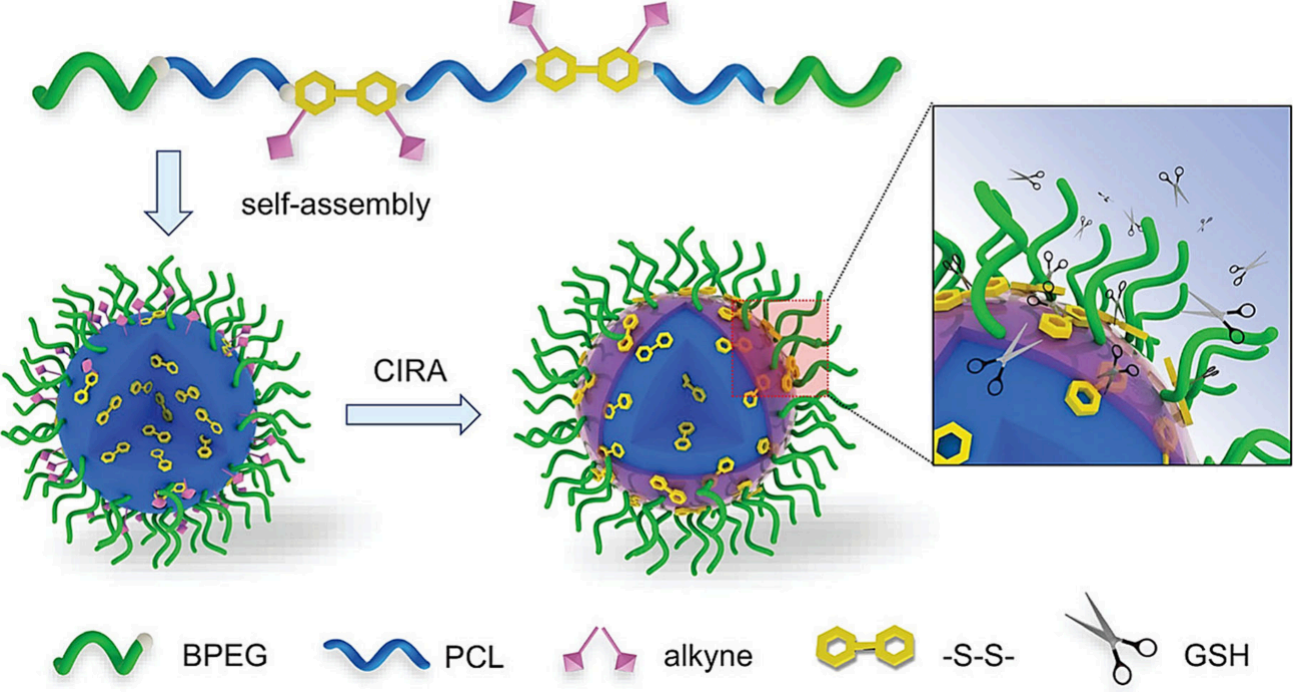
Schematic representation of cross-linking induced reassembly (CIRA) strategy of multiblock polyurethane micelles and its deassembly in the presence of glutathione. Adapted with permission from ref (241). Copyright 2020, Wiley.^241^

In a recent study, Tan and co-workers reported a core-cross-linked multiblock multifunctional polyurethane (MPU) for drug delivery applications.^241^ The MPU consists of a biodegradable PCL unit, a cleavable PEG unit bearing a pH-responsive benzoic-imine linkage (BPEG), l-lysine ethyl ester diisocyanate (LDI), as well as a reducible chain extender generated from l-cystine (Cys-PA). The MPU formed a micellar core–shell structure with a hydrophobic core formed by insoluble PCL soft segments and surrounded by an acid-detachable hydrophilic BPEG corona. To achieve the core cross-linking of the micelle, they synthesized a reduction-cleavable cross-linker (SS-Az) separately, and subsequently reacted this with the alkyne-bearing micellar core. The core cross-linking of the MPU was also confirmed by ^1^H NMR and a fluorescence resonance energy transfer (FRET) study. As a pair of FRET dyes, they encapsulated doxorubicin (DOX, donor) and 3,3′-diethylthiadicarbocyanine iodide (Cy5, acceptor) both into MPU, as well as into CMPU micelles separately, followed by mixing the fluorescent-loaded micelles. Compared to MPU, the core-cross-linked MPU (CMPU) demonstrated an enhanced drug (DOX) loading capacity, improved stability under physiological conditions, and better release behavior in tumor microenvironments. An *in vitro* cytotoxicity study of drug-loaded CMPU revealed that the CMPU displayed a good internalization efficiency and were highly effective to kill drug-resistant cancer cells (MCF7) with a median inhibitory concentration (IC_50_) ca. 2.5 times lower than that of DOX@MPU due to the cleavage of S–S bond in the cancer microenvironment. This was confirmed by confocal laser scanning microscopy (CLSM) and fluorescence-activated cell sorting (FACS). Furthermore, an *in vivo* study in a 4T1 tumor-bearing mice model confirmed that the DOX-loaded CMPU micelles are very effective, to suppress the growth of the tumor with mean tumor weight 1.7-fold and 3-fold lower than those for DOX@MPU and control groups with free DOX, respectively. These results reveal a possible migration and aggregation of Cys-PA moieties from the core to the subsurface layer of micelles due to the azide/alkyne click reaction occurring mainly at the micellar interface, which may induce a cross-linking-induced reassembly process (CIRA) and microphase separation between the soft and hard segments (Figure 44).

**Figure 45:**
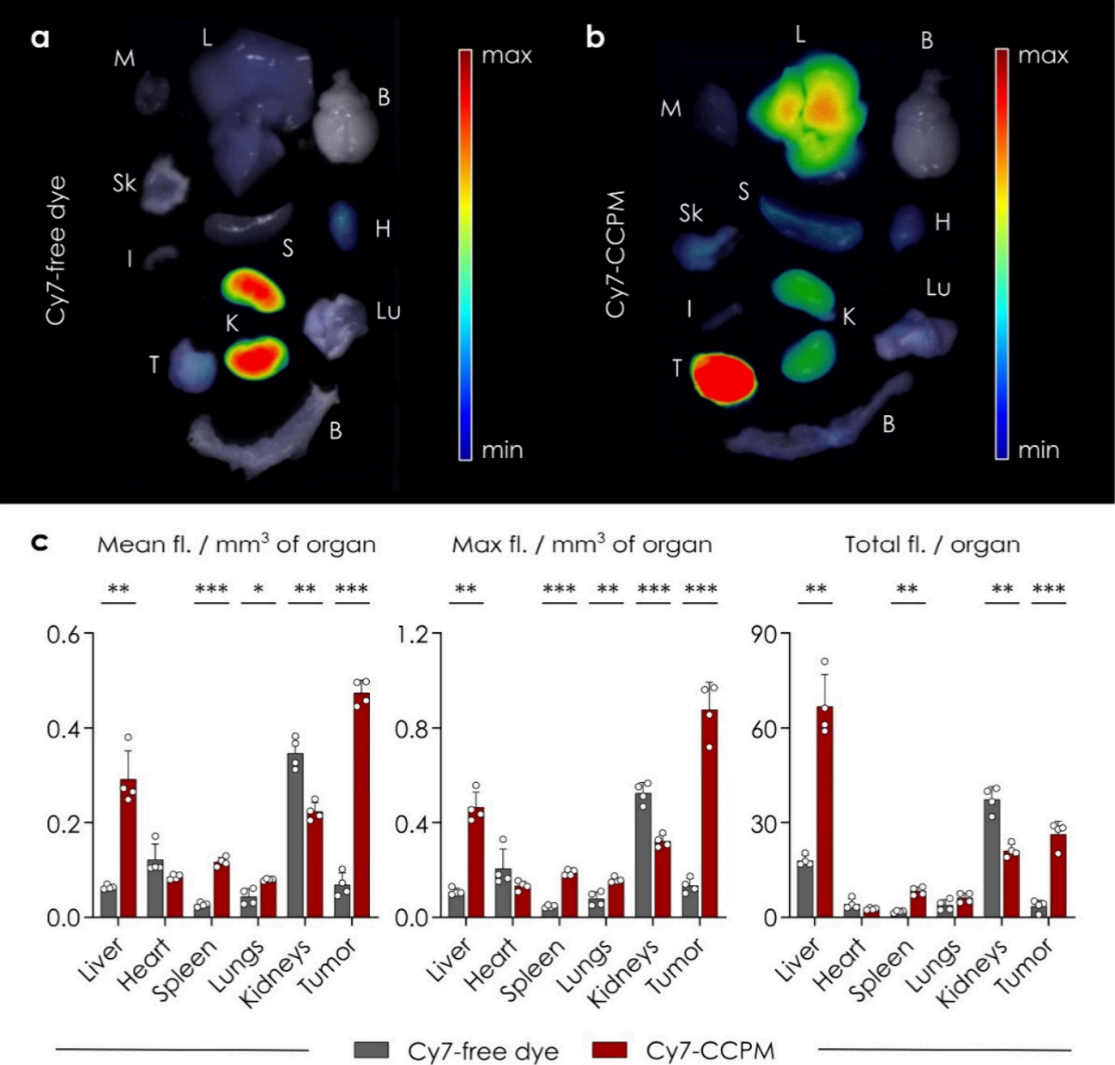
*Ex vivo* organ analysis confirms the efficient tumor accumulation of core-cross-linked polymeric micelles (CCPM) in tumor-bearing mice models for drug delivery applications. (a) 2D fluorescence reflectance imaging (FRI) of resected organs at 48 h post i.v. administration shows high levels of free Cy7 in the kidneys. M = muscle, L = liver, B = brain, Sk = skin, S = spleen, H = heart, I = intestine, K = kidneys, Lu = lung, T = tumor, B = bone marrow. (b) The strongest FRI signals for Cy7-labeled CCPM were observed for tumor. (c) Quantification of the *ex vivo* organ fluorescence for free Cy7 and Cy7-labeled CCPM. *P* values: * < 0.05, ** < 0.01, *** < 0.001. Adapted with permission from ref (242). Copyright 2020, Elsevier.^242^

Similar work was reported by Sofias and co-workers, who also used a core-cross-linked micelle composed of a hydrophilic PEG shell, and of a random copolymer of *N*-2-hydroxypropyl methacrylamide monolactate (HPMAmLac1) and *N*-2-hydroxypropyl methacrylamide dilactate (HPMAmLac2) as a thermosensitive block. They studied the biodistribution of such PMs in tumor-bearing mice models for drug delivery applications.^242^ To observe the biodistribution in a mice model they conjugated Cyanine 7 (Cy7) with azide-functionalized PEG-*b*-pHPMA-lactate using CuAAC, and performed noninvasive 3D microcomputed tomography-fluorescence tomography (μCT-FLT) and 2D fluorescence reflectance imaging (FRI). After intravenous injection of the core-cross-linked micelle in the mice model they noticed that the core cross-linked Cy7-PEG-*b*-pHPMA-lactate achieved highly efficient tumor targeting in the triple-negative breast cancer mice model (18.6%ID/g), with values twice as high as those in liver and spleen (9.1 and 8.9%ID/g, respectively). Furthermore, *ex vivo* assessment of each tissue using FRI images revealed that the free dye primarily accumulated in the kidneys, while insignificant amounts of dye were present in other tissues (Figure 45a). In pleasant contrast, *ex vivo* imaging of organs from mice that were injected with Cy7-PEG-*b*-pHPMA-lactate nicely showed that the micelles were specifically targeting the tumor cells as observed from the intensity of the fluorescence signals (Figure 45b). These results also confirmed a biodistribution pattern that is similar to the *in vivo* results, with apart from tumor most of the micelle signals coming from liver, spleen, and kidney (Figure 45c). In addition to that, they also found that the micelle is efficiently internalized into phagocytic immune cells, which suggest its potential use for immunomodulatory anticancer therapy and drug delivery applications. In another recent study, Li and co-workers reported a duel-function, cyclic brush zwitterionic pH-responsive nanomicelle for both drug delivery and lubrication maintenance for osteoarthritis treatment.^243^ The synthesis of the cyclic brush nanomicelle was achieved by the use of a hybrid approach using both atom transfer radical polymerization (ATRP) and copper-catalyzed azide–alkyne cycloaddition (CuAAC) polymerization techniques. In order to synthesize the cyclic brush polymer, the researchers initially prepared linear precursors (l-P(HEMA)_50_-Br), cyclic polymer templates (c-P(HEMA)_50_), as well as linear and cyclic macroinitiators (l-P(HEMA-Br)_50_ and c-P(HEMA-Br)_50_) using ATRP, intrachain click cyclization, and substitution reactions with propargyl 2-bromoisobutyrate as the initiator. Subsequently, they conducted reactions between [2-(methacryloyloxy)ethyl]dimethyl-(3-sulfopropyl) (SBMA) and cationic *N,N*-dimethylaminoethyl methacrylate (DMAEMA) to obtain the final polymer product. Tribological experiments demonstrated a significant improvement in lubrication when using the cyclic brush in comparison to the bottle brush. The observed improvement may be ascribed to the hydration lubricating mechanism facilitated by the zwitterionic moieties present in SBMA, in conjunction with the distinctive topological effects shown by the cyclic brush structure. The unique architecture of the cyclic brush polymer also allowed the simultaneous encapsulation of the hydrophobic anti-inflammatory medication curcumin (Cur) and the hydrophilic agent loxoprofen (LXP). The architecture of this system enables efficient double drug loading, and enabled the prompt release of LXP and the slow, continuous release of Cur in response to changes in pH. Moreover, the findings from both *in vitro* and *in vivo* experiments demonstrated that the nanomicelles loaded with the superlubricated drug not only provided protection to chondrocytes against degeneration generated by inflammatory factors, but also effectively hindered the advancement of osteoarthritis in the DMM (destabilization of the medial meniscus)-induced osteoarthritis Sprague–Dawley rat model. Significantly, the nanomicelle containing the medication had the ability to increase the expression of anabolic genes related to articular cartilage, while simultaneously reducing the expression of catabolic proteases, thus contributing to its objective to attain a sustained and efficacious therapy for osteoarthritis.

Shoichet and co-workers designed PMs taking advantage of the Diels–Alder cycloaddition click reaction.^244^ The micelle was made of poly(2-methyl-2-carboxytrimethylene carbonate-*co*-d,l-lactide)-*graft*-PEG-furan (poly(TMCC-*co*-LA)-*g*-PEG-furan). In this, the furan groups are present at the outside of the micelles, and can take part in a subsequent Diels–Alder reaction with maleimide-modified anti-HER2, a therapeutic antibody used to treat breast cancer. Under mild conditions, and keeping the efficacy of these sensitive antibodies, these were coupled both efficiently and with a high reaction rate. In their subsequent work, they also used the Diels–Alder reaction to obtain nanoparticles with both DOX and anti-HER2 covalently bound at the outside of the PMs.^245^ These anti-HER2-DOX PMs used the anti-HER2 peptide to focus on the cell nucleus of HER2-overexpressing SKBR-3 cells, after which DOX could be locally delivered with great efficiency (*in vitro* experiments*)*. The nanomicelle with both DOX and anti-HER2 antibody exhibited enhanced intracellular uptake and greater apoptosis (9.5% cells with early apoptosis and 15.4% cells with late apoptosis) in SKBR-3 cells relative to nanomicelle with only DOX (7.2% cells with early apoptosis and 8.8% cells with late apoptosis), and significant specificity to cancerous cells versus healthy cells in terms of cytotoxicity. The enhanced apoptosis induced by nanomicelle antiHER2-DOX is likely due to the enhanced intracellular uptake associated with antibody-mediated endocytosis.

Another way to obtain localized release of drugs was achieved by Lim and co-workers, who developed near-infrared (NIR) light-responsive, diselenide containing core-cross-linked micelles prepared by the Diels–Alder reaction.^246^ The core-cross-linked PM was made of PEG-*b*-poly(furfuryl methacrylate) (PEG-*b*-PFMA) and a separately synthesized diselenide-containing bismaleimide cross-linker. To make the system a NIR-responsive drug delivery system, they loaded the micelle with a NIR-sensitive dye indocyanine green (ICG) and DOX. Under NIR (808 nm) light irradiation ICG generates reactive oxygen species, which cleave diselenide bonds in the core of the PMs. This allows the rapid release of DOX from the decross-linked micelles, leading to significantly enhanced apoptosis in HepG2 cells, as evidenced from cytotoxicity and confocal laser scanning microscopy imaging tests. In another recent study, Mai et al. synthesized pH-sensitive PMs, using the photoinduced copper-mediated reversible deactivation radical polymerization (PI-CMRDRP) to form a copolymer with a PEG block and a poly(diisopropylaminoethyl methacrylate-*co*-furfuryl methacrylate)s block (PEG-*b*-P(DPA-*co*-FMA)), and subsequently involved a Diels–Alder reaction using 1,1′-(methylenedi-4,1-phenylene)bismaleimide as cross-linking agent.^247^ The cross-linked PMs could encapsulate hydrophilic model drugs, such as rhodamine B, and release it in a pH-dependent manner, which suggests its potential applicability as a pH-responsive drug carrier.

Apart from CuAAC and Diels–Alder reactions, other click reactions are not yet popular for the synthesis of polymeric micelle for drug delivery applications. Evidently they do display potential and these reactions are thus very much under consideration for the synthesis of novel drug delivery platforms. For example, a recent study by Wu and co-workers reported a late-stage drug functionalization strategy based on the SuFEx click reaction.^248^ In their study, they used SuFEx click chemistry to perform the late-stage functionalization of a panel of known anticancer drugs to generate the corresponding arylfluorosulfates from their phenolic precursors. Furthermore, they tested *in situ*-generated arylfluorosulfates in a cancer-cell growth inhibition assay in parallel with their phenolic precursors. This revealed that the fluorosulfate derivative of Fulvestrant possesses significantly enhanced activity to down-regulate estrogen receptor (ER) expression in the ER+ breast cancer cell line MCF-7 (IC_50_ = 5.5 nM), whereas the fluorosulfate derivative of Combretastatin A4 shows a large increase in potency in the drug-resistant colon cancer cell line HT-29 (IC_50_ = 70 nM) compared to the phenol precursors of fluorosulfated products (IC_50_ = 4947 nM).

#### 3.1.2 Hydrogel (Microgel and Nanogel) for Drug Delivery Applications

Micrometer-sized hydrogels (microgels, 1–350 μm) and nanometer-sized hydrogels (typically 20–250 nm)^182^ are considered to be among the prime contenders for drug delivery applications, due to their good biocompatibility, high water content, large surface area for biofunctionalization and targeted delivery, and abundant space to accommodate bioactive molecules, such as drugs, genes, etc. In addition to that, due to their small sizes they can access those areas of our body that are inaccessible to traditional hydrogels. In this context, click chemistry provides a unique platform to synthesize microgels and nanogels and with tunable sizes and functionalities for drug delivery applications. We will first discuss the use of microgels, then that of nanogels.

**Figure 46:**
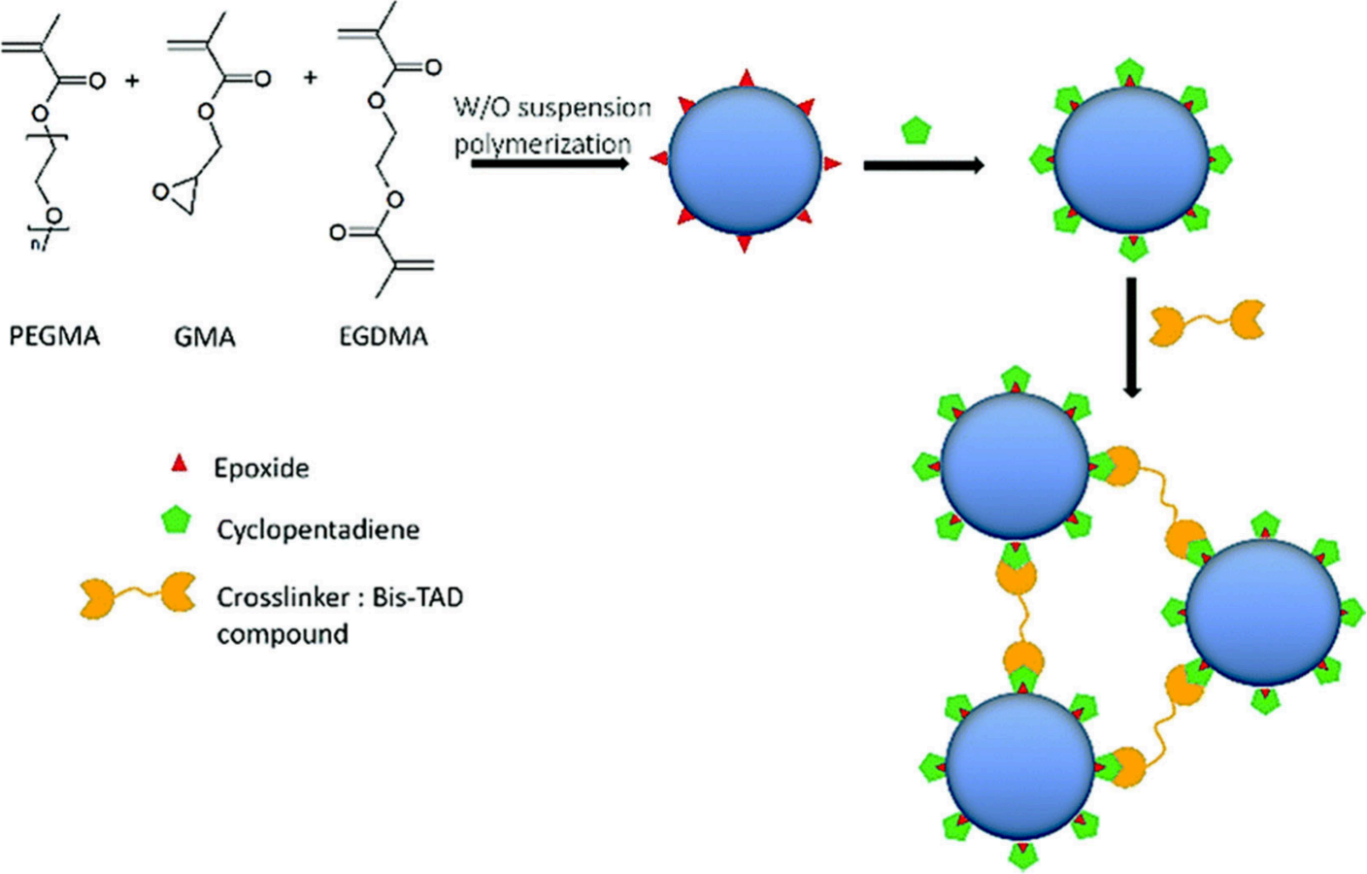
Synthetic steps for the design of doubly cross-linked injectable microgels via click chemistry between cyclopentadiene and triazolinedione groups. Adapted with permission from ref (252). Copyright 2016 RSC.^252^

Paradossi and co-workers reported a core–shell poly(vinyl alcohol)-hyaluronic acid (PVA-HA) microgel with a diameter of ∼2 μm by combining an inverse emulsion technique and CuAAC.^249^ The hydrogel displayed a good DOX-loading efficiency of 3.3 × 10^–5^ moles DOX/g of dry microgels, and rapid release behavior *in vitro*: about 60% of the initially loaded DOX is released in about 3 h. Additionally, due to the presence of HA, the microgels exhibited a remarkable specificity to CD44 receptor-overexpressing adenocarcinoma colon HT-29 cells, and released DOX effectively caused cell death. Sivakumaran et al. designed a hybrid hydrogel consisting of thermoresponsive microgels physically entrapped or covalently cross-linked to nonthermoresponsive dextran hydrogels via a hydrazide-aldehyde reaction to facilitate the long-term release of a small molecule drug bupivacaine.^250^ Here they efficiently used Schiff base click chemistry to cross-link the microgel particles, so as to modulate drug release. They observed that both the overall rate of drug release and the magnitude of the burst release was significantly reduced when the microgels had higher internal cross-linking densities and/or were covalently cross-linked with the bulk hydrogel. Cao and co-workers developed cell-laden microgels based on a new HA derivative, i.e., furylamine and tyramine-grafted HA molecules, which are cross-linked via either enzymatic cross-linking or Diels–Alder click chemistry or both, for cell encapsulation and delivery.^251^ The microgel did not demonstrate any cytotoxicity toward ATDC-5 cells and nicely showed a degradation response that depended on a tunable degree of cross-linking. Mespouille and co-workers developed click-reactive double cross-linked microgel for injectable purposes.^252^ In particular they used the fast 1,2,4-triazoline-3,5-dione-based Diels–Alder click chemistry (see Section 2.3.2.3) for the synthesis,^205^ with high product yield and without use of any catalyst or stimuli. To further highlight the practical nature of this reaction, it can be noted that the reddish color of the TAD compound vanishes after coupling, as the product is colorless, offering visual feedback for the progress of the reaction. The synthesized microgel is composed of PEG-methyl ether methacrylate, glycidyl methacrylate and ethylene glycol dimethacrylate. To cross-link the microgel, they performed a post-polymerization modification by reacting the glycidyl functions with sodium cyclopentadienide (NaCp), which resulted in Cp-functionalized microgels. Finally, they mixed the microgels with a bis-TAD functionalized cross-linker, resulting in cross-linking with adequate kinetics to form a doubly cross-linked microgel network (Figure 46). Due to the double cross-linked nature of the microgel, it can release drug molecules in a much-sustained manner compared to a singly cross-linked gel, pointing to the usefulness of this approach for drug delivery.

Astrain et al. studied the drug release profile of hydrogels composed of various PEG-based bismaleimides and trismaleimides—with different molar masses, chemical architectures and functionalities—as cross-linkers for furan-modified alginate chains via Diels–Alder click reactions.^253^ They observed that the use of short PEG-based cross-linkers resulted in a burst release of the loaded vanillin as a model drug, whereas the use of long-chain PEG-based cross-linkers and trismaleimide-based cross-linkers leads to a more sustained drug release over time. So, based on the required drug release profile for specific applications, these hydrogels can be employed selectively. Galan and co-workers reported a facile synthesis strategy of chitosan-based hydrogels and microgels through thiol–ene photoclick cross-linking.^254,255^ For their synthesis they used norbornene-functionalized chitosan, Irgacure 2959 as a photoinitiator and the dithiolated cross-linker 2,2′-(ethylenedioxy)diethanethiol. The cross-linked particles are within the range of 100–400 nm, i.e., large nanogels. These particles did not display obvious toxicity toward human dermofibroblasts cells, and showed tunable nanopores within their structures.

Haag and co-workers reported biodegradable dendritic polyglycerol nanogels by inverse nanoprecipitation and CuAAC for the encapsulation and release of pharmaceutical biomacromolecules.^256^ The hydrogel was composed of azide- and alkyne-substituted dendritic polyglycerol with acid-labile acetal linkers. The mild synthetic conditions of CuAAC enabled encapsulation of asparaginase with no adverse effect on protein structural integrity and activity. *In vitro* release profiles of asparaginase from the gel revealed that the release at pH 7.4 is very slow, while on the other hand almost 100% of the protein is released after 5 h at pH 4 or in 35 h at pH 5. Taking advantage of click chemistry, Ossipov et al. developed a multifunctional hyaluronic acid (HA) nanogel.^257^ For their synthesis they used, an aldehyde/thiol dual-functionalized clickable HA, acid-labile PVA-carbazate DOX prodrug, and pyridyl disulfide-modified pyrene (to release 2-thiopyridine as a result of the formation of a new S–S bond between the HA backbone and the pyrene ligand). The size of the nanogels was about ∼200 nm. Upon examining the *in vitro* release behavior of DOX-loaded nanogels they found that 50% of DOX was released in 100 h at pH 5.0, which is almost similar to the pH of the tumor environment, while only 19% DOX was released after that time at pH 7.2. Furthermore, they observed that DOX-loaded nanogels were taken up more efficiently by CD44 receptor-overexpressing MDA-MB-31 cells than by MCF-7 cells (which display a low expression of CD44), indicating that HA-presenting nanogels could selectively recognize CD44-positive cells. This is a nice result, as it suggests that such highly functionalized nanogels could be potential drug nanocarriers for triple-negative breast cancer treatment. Anderson and his group members developed bisphosphonate-functionalized dextran nanogels (∼70 nm) via a stoichiometric CuAAC click-chemistry inverse emulsion method for targeted therapeutic applications toward bone disease.^258^ They discovered that functionalization of these nanogels with bisphosphonate ligands reduced the distribution of nanogels in liver and kidney tissues by 43%, and targeted binding to the inner walls of the marrow cavity in both the femur and spine. This led to the depletion of osteoclasts and contributed to an overall antiosteoporotic effect.

Fu et al. reported the synthesis of well-defined, functionalized PEG-fluorescent nanogels via a reverse emulsion CuAAC (REM-CuAAC) reaction.^259^ In this process an alkyne-functionalized gallium (Ga)-porphyrin complex and an azide-functionalized PEG were reacted to yield gels of 30–120 nm, which showed a fluorescence emission maximum at a wavelength of 700–800 nm, indicating that they might have potential application in near-infrared imaging and drug delivery purposes. Matyjaszewski and his group developed HA-nanostructured hydrogels through a thiol-Michael reaction of thiolated HA with acrylate-functionalized nanogels that were made via REM-CuAAC (a combination of ATRP and inverse miniemulsion), followed by converting pendent hydroxyl groups into acrylate groups. Azido-functionalized polymers that were generated using ATRP, were further reacted via a CuAAC reaction with an alkyne precursor through a reverse emulsion process, which yielded nanogels in the dispersed phase with a 30–120 nm diameter. The hydrogel could be used for the controlled release of a model drug, as the gel degraded upon hydrolysis into polymeric sols.^260^ Molinos et al. reported a hybrid dextrin-based hydrogel encapsulating a dextrin nanogel for protein delivery application using oxime/hydrazone click chemistry.^261^ Briefly, they synthesized the hydrogel by reacting oxidized dextrin and adipic acid dihydrazide, where adipic acid dihydrazide acts as a cross-linker. They used this, a.o., for the controlled release of insulin, and noted that ghe gradual release of insulin was around 12% or 35% (depending on the precise formulation of the gel) after 20 days under physiological conditions. Such easily tunable and prolonged sustained release of the protein is beneficial for injectable delivery systems, as it might provide slow but continuous protein delivery. This minimizes cyclic fluctuations in blood protein levels over time, and thereby maximizes the pharmacological efficacy at lower drug doses, reduces the frequency of administration, and enhances patient compliance with the therapy.

**Figure 47:**
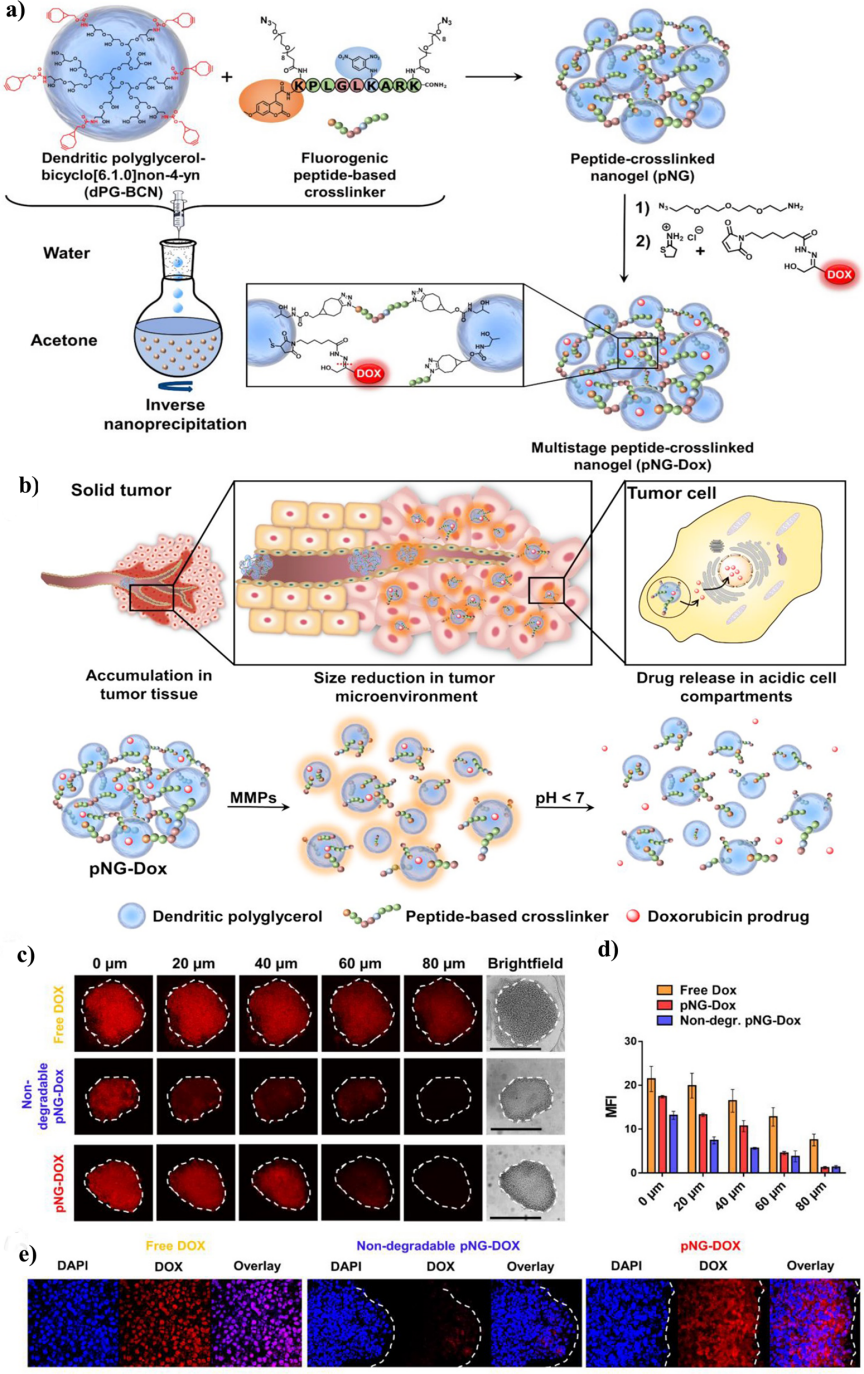
a) Schematic representation of the building precursors, and multistage peptide cross-linked nanogels (pNG-Dox) fabricated by conjugating doxorubicin (DOX) drug through a pH-sensitive linker to the pNG scaffold. b) Schematic representation of the mechanism proposed for the multistage drug delivery by pNG-DOX. c) Penetration of pNG-DOX into multicellular tumor spheroids (MCTS) highlighting the area with white dashed lines: comparison of free DOX, degradable multistage pNG-Dox, and the nondegradable control. The black bars in the brightfield images represent 500 μm; confocal laser scanning microscopy (CLSM) images with 20-fold magnification. d) Mean fluorescence intensity of DOX over the area of the MCTS for different penetration depths. e) CLSM images of cryosections with 64-fold magnification. Adapted with permission from ref (263). Copyright 2020, Ivyspring International Publisher.^263^

Wang et al. reported pH-sensitive hydrogels with *ortho*-ester linkages synthesized via thiol–ene click chemistry for intracellular drug delivery application in cancer cells *in vitro*.^262^ The nanogels were prepared by reacting *ortho*-ester diacrylamide (OEAM), pentaerythritol tetra(3-mercaptopropionate), and methoxyl-PEG acrylate (mPEGA), with a size distribution of 100–200 nm. The hydrogel demonstrated a high DOX loading efficiency of 74%, with a pH-sensitive release behavior–because of the orthoester bond–as three-quarters of the loaded DOX was released from the nanogel within 24 h at pH 5.0. Cellular uptake studies confirmed that the drug-loaded nanogel was readily internalized by SH-SY5Y cells and exhibited excellent antitumor activity toward cancer cells. More specifically a dose-dependent decrease in cell viability was noticed when treated with free DOX and the nanogel loaded with DOX. In this, the IC_50_ value of nanogel/DOX (7.8 μg/mL) was higher than observed for free DOX (3.2 μg/mL), which is likely due to the controlled drug release from the nanogel. Furthermore, when three-dimensional multicellular tumor spheroids (3D-MCTS) were used as an *in vitro* tumor model, the nanogel/DOX exhibited a significantly improved penetration and growth inhibition in 3D-MCTS, compared to free DOX. In particular, MCTS treated with the (empty) nanogel and nontreated MCTS increased in diameter from 209 to 289 μm and from 203 to 282 μm, respectively. Whereas, MCTS treated with free DOX or with a DOX-loaded nanogel displayed a sustained decrease in the spheroid diameter from 213 to 100 μm and from 218 to 71 μm, respectively. Such pH-sensitive nanogels thus dispayed significant potential as drug delivery vehicles for cancer therapy. Calderon and co-workers also developed an efficient nanogel for enhanced drug delivery in the 3D tumor model.^263^ However, unlike Wang et al.^262^ their hydrogel was not only pH sensitive, but also specifically sensitive toward an enzyme known as matrix metalloproteinase (MMP). The nanogel was prepared by reacting cyclooctyne-functionalized dendritic polyglycerol (dPG) with a modified MMP-sensitive fluorogenic peptide cross-linker using SPAAC click chemistry as shown in Figure 47a. They noticed that the size and cross-linking density of the nanogels could be controlled efficiently by tuning the functionalization degree of dPG with cyclooctyne groups, and by the peptide cross-linker fraction. DOX molecules were attached to the nanogel through a pH-sensitive linkage to dPG, which make the nanogel a multistage drug delivery system. As the nanogel contains a MMP-sensitive moiety, the nanogel readily degrades in the presence of the MMP, whereas showing enough stability in PBS and cell media. Additionally, the acidic pH-labile conjugation of DOX within the hydrogel made it a pH-sensitive drug release system (pNG) (Figure 47b). In the presence of MMP, digestion of the nanogels led to a size reduction to polymer–drug fragments, which more efficiently penetrated agarose gels as well as MCTS compared to its nondegradable control and free DOX, as confirmed by CLSM images (Figure 47c) and mean fluorescence intensity (Figure 47d). The confocal images at higher magnification (Figure 47e) also confirm the enhanced penetration of DOX for the degradable pNGs, and show that the controlled size reduction of the pNGs enhances the transport of the therapeutic agent into the tumor-resembling 3D model. More importantly, when they accessed the therapeutic efficacy in terms of intracellular ATP measurements in a 3D-MCTS model, the MMP-responsive pNGs significantly outperformed the nondegradable control, by effectively delivering the drug to the inner regions of the tumor model.

**Figure 48:**
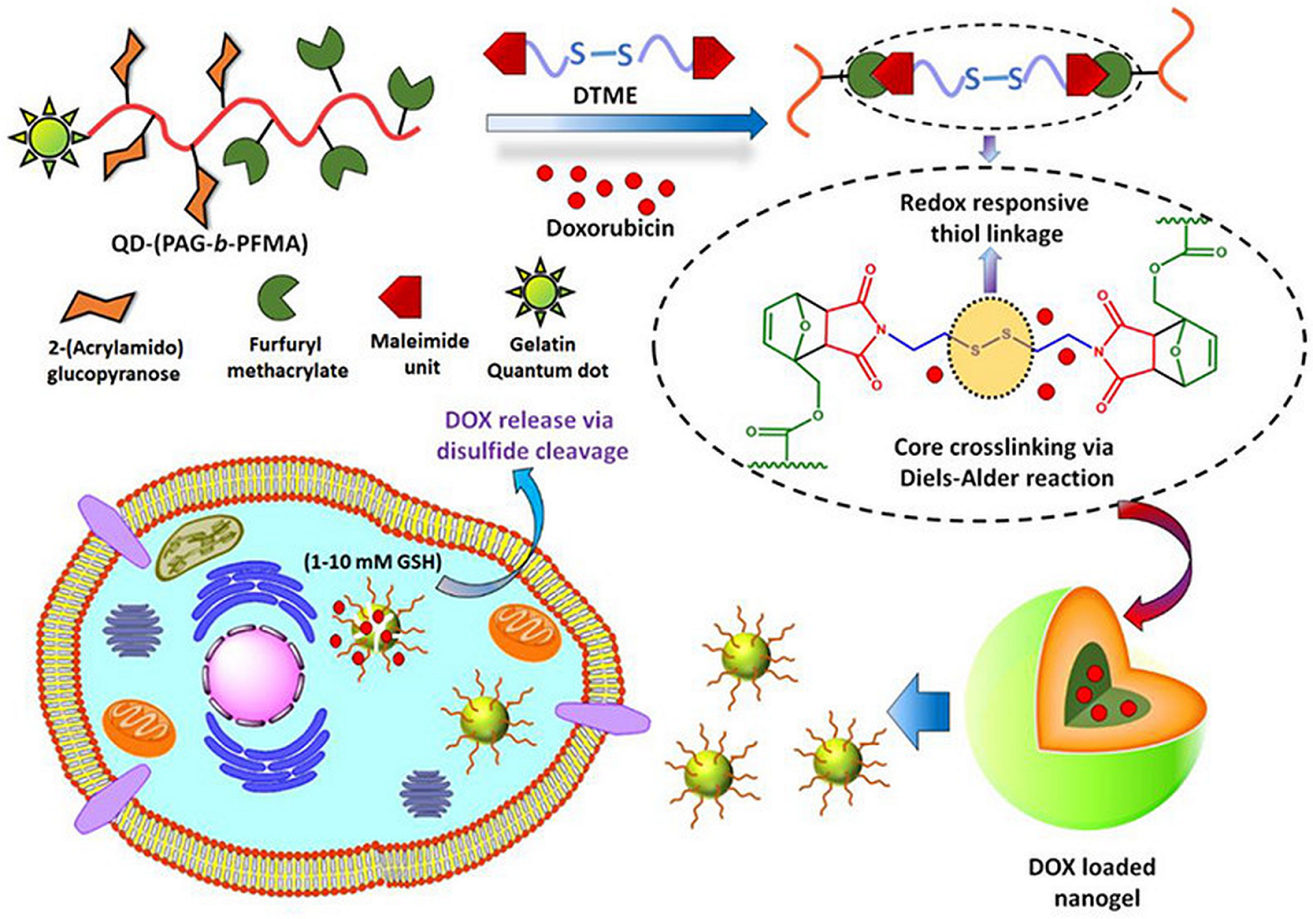
Development of a well-defined glycopolymer-based fluorescence-active nanogel via the combination of RAFT polymerization and Diels–Alder (DA) chemistry, and subsequent DOX-loading and release behavior. Adapted with permission of ref (264). Copyright 2019, ACS.^264^

Analogously for the delivery of anticancer medication, Singha and his group members, reported a redox-responsive, fluorescence-active glycopolymer-based nanogel using Diels–Alder click chemistry.^264^ To synthesize the nanogel, first they prepared a functional AB block copolymer poly(pentafluorophenyl acrylate)-*b*-poly(furfuryl methacrylate) (PPFPA-*b*-PFMA), in which the pentafluorophenyl ester groups act as activated leaving groups. Subsequently, the pentafluorophenyl moiety was replaced by the amine functionality of glucosamine, to prepare the amphiphilic poly[2-(acrylamido) glucopyranose]-*b*-poly(furfuryl methacrylate) (PAG-*b*-PFMA). Additionally, the terminal carboxylic groups of the RAFT reagent (4-cyano-(phenylcarbonothioylthio) pentanoic acid (CPPA)) were modified using gelatin quantum dots (GQDs) to make the polymer fluorescent. After that, the finished product was cross-linked by dithio-bismaleimidoethane (a redox-responsive cross-linker) utilizing furan-maleimide Diels–Alder chemistry (see Figure 48). To check the anticancer efficacy of the DOX-loaded nanogel, a MDA-MB-231 human breast cancer cell line was, in two separate experiments, loaded with unloaded and DOX-loaded nanogel. It was observed that the nanogel itself was noncytotoxic toward MDA-MB-231 cells up to 62.5 μg/mL concentrations, while the drug-loaded nanogel was highly active to kill the cancer cells. The two compositions of DOX loaded nanogel PAG_20_-*b*-PFMA_20_ and PAG_20_-*b*-PFMA_32_ showed an IC_50_ value of 1.08 μM and 0.91 μM, respectively, which is higher than free DOX (0.63 μM), suggesting controlled drug release from the nanogel. Furthermore, the authors noticed that the DOX-loaded nanogel induced cell death through an apoptotic mechanism, indicating a controlled and targeted approach to cell death.

In another recent study, Ki and co-workers developed a pectin-based nanogel using thiol–norbornene photoclick chemistry for transcutaneous antigen delivery.^265^ In order to synthesize the nanogel, the researchers conducted a thiol–norbornene photoclick reaction using norbornene-functionalized pectin, dithiothreitol as the dithiol cross-linker, and thiolated ovalbumin (OVA). It was observed that the nanogel exhibits uniformity in size, with an approximate diameter of 200 nm, and displayed exceptional storage stability when maintained at a temperature of 4 °C. It was discovered that the OVA-loaded nanogel had the ability to permeate the stratum corneum and accumulate inside both the epidermis and dermis layers. Furthermore, the nanogels were successfully absorbed by THP-1 monocytes that were generated from dendritic cells, resulting in the increased expression of maturation markers. The findings of this study indicate that the nanogel exhibits promise as a possible nanocarrier for the delivery of antigens via the skin. Such an example, like several of the ones before, shows the potential therapeutic applicability of drug-loaded hydrogels for anticancer drug delivery applications, as they can combine a steady, side effect-lowering release of drugs with a myriad of other functionalities that can be used to, e.g., provide control over drug release in term of location (targeting specific receptors) and time (for example, upon incorporation of light-induced release features). Ki et al. hypothesized that dual-mode deterioration—bulk degradation with surface erosion—was to responsible for this. As per there conclusion, initially, swelling and a reduction in cross-linking density were the outcomes of lysozyme’s cleavage of pectin chains. After 8 h, the nanogel’s size shrank and surface erosion took over. The concentration of pectin-NB had an impact on the size alterations. The initial size increase was comparatively minimal at higher doses, indicating slower deterioration. Therefore, adjusting the cross-linking density can influence the rate of antigen liberation.

#### 3.1.3 Polymeric Capsules and Microspheres for Drug Delivery Applications

Other than polymeric micelles and hydrogels (nanogels and microgels), polymeric capsules and microspheres are attractive candidates for drug delivery applications. Generally, polymeric capsules are prepared using a layer-by-layer (LBL) assembly technique followed by deposition of alternating polymeric layers over sacrificial cores, which are subsequently sacrificed at the end-stage of the synthesis. While the formation and stability of traditional LBL-assembled microcapsules was usually driven by noncovalent interactions (hydrogen bonding, electrostatic interaction, π–π stacking, van der Waals forces, etc.), the use of click chemistries opened up the field of covalently bound LBL-based microcapsules, which offer greater stability due to their covalently cross-linked polymeric structure. For instance, in one of the early works in this field, Caruso and co-workers employed CuAAC-based LBL assembly for the synthesis of pH-responsive microcapsules.^266^ To prepare the microcapsules they synthesized functionalized poly(acrylic acid) chains, with either azide or alkyne modifications (PAA-Az/PAA-Alk) and subsequently assembled these on silica nanoparticles in alternate fashion. Furthermore, remaining alkyne moieties present in the coated film could afterward be functionalized upon exposure to an azide-modified rhodamine dye. The silica nanoparticles can be used as sacrificial core, and so could be removed at the final stage to leave a hollow polymeric capsule. As poly(acrylic acid) itself possesses pH-responsiveness, the microcapsules also showed pH-responsive swelling/shrinking by reversible size-changing behavior in basic or acidic conditions. In a follow-up study, such a combination of click chemistry and LBL assembly was used with alkyne- and azide-modified poly(l-lysine) (PLL) and poly(l-glutamic acid) (PGA) as covalently reacting LBL components.^267^ The resulting microcapsules were stable within a wide pH range of 2–11 with concomitant pH-responsive shrinking/swelling behavior. In addition, rather than appending a dye, here they demonstrated that the microcapsulates could be further functionalized by a monolayer of heterobifunctional PEG, which can act as an antifouling outer layer and endows the capsule stealth character. This combination of stealth character and options for further functionalization make such microcapsules attractive candidates for drug delivery applications. Geest et al. reported the synthesis of hollow microcapsules via CuAAC of azide-modified and alkyne-modified dextran over a precoated LBL-assembled surface of poly(acrylic acid)-propargylamine on CaCO_3_ microparticles.^268^ The CaCO_3_ microparticles at the core of the capsules dissolved in aqueous EDTA solution at pH 5.2, which resulted in hollow microcapsules. Usage of carbonate linkages for the incorporation of azide and alkyne functionalities along with a dextran polymer chain confirmed the biodegradability of the capsules, and ensured gradual drug delivery capabilities. In successive work, they designed a biodegradable microcapsule from azido- and alkyne-modified-dextran by microemulsion using an external aqueous PEG phase and monitored the release of dextran from the microcapsules.^269^ They noticed that the release of dextran occurred in a controlled fashion, and that the release rate could be modulated by altering the cross-linking density of the microcapsules.

Connal et al. employed thiol–ene click chemistry to prepare polymeric microcapsules without the use of any catalysts.^270^ Here, they constructed an LBL assembly of thiol-functionalized and alkene-functionalized poly(methacrylic acid)(PMA) and poly(*N*-vinylpyrrolidone) (PVP) using H-bonding on a sacrificial silica core. Similar to the study of Caruso above,^266^ this thiol–ene click approach also allows remaining thiol or ene functional molecules to be used to click functionalities onto the capsule in a late-stage functionalization. To demonstrate this, they functionalized the outer corona of PVP/PMA-coated particles with two different functionalized PEG chains: a monofunctional PEG-acrylate (PEG-1) and a bifunctional maleimide-PEG-succinimidyl ester (PEG-2). Functionalization of these two PEG chains on the outer surface of microcapsules introduced antifouling properties (in the case of PEG-1) or enhanced—albeit nonspecific—protein affinity (in the case of PEG-2), which allows finetuning of these microcapsules in any use as novel delivery system. In a similar type of work, a CuAAC-based strategy was used by Kamphuis et al. to prepare antifouling polymeric capsules modified with antibodies that can specifically target colorectal cancer cells.^271^ Here also, PVP, PMA, and PEG were used to synthesize the capsules and make them antifouling, while the capsules were finally functionalized with an azide-modified huA33 mAb antibody, which endows it with the potential to exclusively bind to colorectal cancer cells. The antibody-functionalized capsules specifically bind to colorectal cancer cells even if the target cells constitute less than 0.1% of the total cell population. Such biospecific or “romantic” surfaces (meaning: repelling all agents, but strongly attracting one specifically),^272^ make these capsules promising contenders for colorectal anticancer targeting drug delivery applications.

A significant step in such studies was made in 2010, when Ochs et al. showed for the first time the *in vitro* use of biodegradable click capsules with anticancer drug-loaded multilayers to kill cancer cells.^273^ This was the first study that truly evaluated the anticancer efficacy of drug-loaded capsules, whereas in all previous studies researchers only suggested or predicted that microcapsules could be used as drug delivery vehicles. Briefly, this group synthesized alkyne-modified PGA and DOX-conjugated alkyne-modified PGA and assembled a mixture of these into a LBL assembly with PVP via H-bonding. The LBL assembly was cross-linked using diazide cross-linkers via CuAAC click chemistry. Here also they used silica as a sacrificial template. Finally, the PVP layer was removed by altering the solution pH to disrupt the H-bonding, which resulted in the formation of single-component PGA microcapsules that were used for drug delivery application. The PGA microcapsules are well stable within a wide pH range of 2–11 and exhibited reversible swelling (a maximum swelling of 83%)/shrinking behavior. In addition to that, the microcapsules demonstrated a sustained drug release profile with good enzymatic degradability. Cellular uptake and cell viability studies revealed that in the presence of DOX-loaded PGA capsules the viability of LIM1899 cells significantly decreased (32% in comparison to untreated cells), which confirmed its applicability for drug delivery application. While not displaying perfect drug efficacy, this clearly set the bar upon which further research could improve.

While many studies focused on the preparation of polymeric capsules based on preorganized structures or templates, only few studies focused on the synthesis of self-assembled nanosized capsules without the use of any template. In this context, Kim and co-workers developed polymeric nanocapsules without the use of any template through irreversible covalent bond formation.^274^ In detail, they employed a photoinitiated thiol–ene click reaction between (allyloxy)_12_ cucurbit[6]uril, a cavity-containing rigid disk-shaped molecule with polymerizable allyl groups at the periphery, and oligoethylene oxide or alkyl-based dithiols. This approach is applicable for any building unit with a flat core and multiple polymerizable groups at the periphery that can direct grow polymer in lateral directions. Although they did not demonstrate any applications of these nanocapsules, potential application for drug delivery purposes seems feasible.

Thayumanavan and colleagues devised polymer–drug nanoconjugates comprising poly(salicylhydroxymate)-*b*-poly(methacrylate-PEG) conjugated with bortezomib drugs via boronic acid salicylhydroxamate reversible click chemistry. These nanoconjugates effectively induced cytotoxicity in cancer cells, including HeLa, MDA-MB-231, and MCF-7, at low dosages, while exhibiting minimal toxicity from the polymer component alone.^275^ Further, in 2023 Yan et al. developed polymeric nanomicelles composed of PEG-*b*-poly(amino acid) through thiol–ene click/thiol–yne click reactions. Through *in vitro* drug release studies, they demonstrated a notably accelerated release rate of oxaliplatin in the acidic intracellular environment characteristic of cancer cells (pH 5.0) compared to physiological conditions (pH 7.4). These drug-loaded nanomicelles exhibited concentration-dependent inhibition of cancer cell proliferation against HepG2 and MCF-7 cells.^276^ Vermonden et al. synthesized a hydrogel via a Diels–Alder cycloaddition reaction, using poly(NIPAM-*co*-2-hydroxyethyl acrylate/furan)-PEG6000-poly(*N*-isopropylacrylamide-*co*-2-hydroxyethyl acrylate/maleimide). This hydrogel demonstrated the sustained release of dexamethasone (DEX) and an anti-VEGF antibody fragment (FAB) protein over 35 and 13 days, respectively. Moreover, it exhibited favorable cytocompatibility with macrophage-like mural cells (RAW 264.7) and human retinal pigment epithelium-derived (ARPE-19) cells.^277^ Sanyal et al. engineered redox-active nanogels using PEG methyl ether methacrylate, furan-protected maleimide, and disulfide-bearing methacrylate via thiol–maleimide chemistry. These nanogels exhibited robust internalization and anticancer efficacy against MDA-MB-231 tumor cells, facilitated by sustainable release of docetaxel (DTX).^278^ Yuan et al. in 2023 developed polymeric micelles comprised of poly[3-dimethyl(methacryloyloxyethyl) ammonium propanesulfonate] (PDMAPS) conjugated with keratin through a thiol–maleimide reaction. These micelles, loaded with DOX, displayed triple responsiveness to pH, glutathione (GSH), and trypsin within the tumor microenvironment. They demonstrated excellent activity against A549 cancer cells while exhibiting minimal toxicity in the absence of DOX against normal cells.^279^

As a final example in this category, Zhang and co-workers developed a lactobionic-loaded chitosan microcapsule using CuAAC for drug delivery with a liver-targeting capability.^280^ For the synthesis of microcapsules, they exploited the LBL assembly of azide-functionalized and alkyne-functionalized chitosan to form the microcapsules around sacrificial CaCO_3_ microparticles. To introduce the liver-targeting capability they conjugated lactobionic acid on the surface of microcapsules, which led to improved internalization within HepG2 cells compared to unmodified chitosan microcapsules.

Finally, a short-hand overview over a series of related studies is given in Table 1, which displays the use of click chemistries for drug delivery in specified polymeric systems and the results thereof.

**Table 1:** List of Click Chemistry-Based Polymeric Systems Used for Drug Delivery Studies

Polymeric systems used as drug delivery carrier	Click chemistry used	Delivered drugs	Results and uses	Ref.
Micellar system made of dextran-*block*-β-cyclodextrin/benzimidazole-modified poly(ε-caprolactone) copolymer	CuAAC	Doxorubicin (DOX)	DOX release from DOX-loaded micelles; release enhanced in acidic conditions, mimicking the endosomal/lysosomal compartments; improved intracellular DOX release was observed in HepG2 cells, showed higher cellular proliferation inhibition toward HepG2 cells than pH insensitive micelles	(281)
Micellar system made of biotin-conjugated PEG-poly(lactide-carbonate) block copolymer	CuAAC	DOX	pH-dependent DOX-release due to oxime cross-linker. Cell culture studies of biotin and DOX-conjugated nanocarrier showed increased intracellular DOX accumulation at cancer cell, inhibiting tumor cell growth at low concentrations *in vitro*	(282)
Multiblock polyurethane-based micelles, with soft blocks (obtained via detachable PEG and degradable poly(ε-caprolactone)), and hard segments (constructed from Lys- and Cys-derivatives bearing reduction-responsive disulfide linkages and click-active -C≡CH moieties)	CuAAC	DOX	Cleavage of PEG corona bearing a pH-sensitive benzoic-imine linkage worked as an on–off switch under extracellular acidic conditions, triggering core degradation and payload release within tumor cells, leading to death of tumor cells	(283)
Matrix metalloproteinase-2 (MMP-2)-sensitive micelles prepared of copolymer of PEG and partially hydrolyzed poly(β-benzyl l-aspartate	CuAAC	DOX	IC_50_ value of DOX-loaded micelles = 0.38 μg/mL compared with 0.66 μg/mL of free DOX. Release via MMP-triggered dePEGylation. RGD-mediated cellular uptake, and rapid drug release inside cells.	(284)
			HT1080 multicellular tumor spheroids confirmed high affinity and deep penetration of micelles in tumor tissues	
MMP-sensitive micelles made of PEG-*b*-poly(d,l-lactide) copolymer	CuAAC	PTX	Cytotoxicity of PTX-loaded micelles against 4T1 cells significantly enhanced compared to free PTX; *in vivo* studies displayed prolonged blood circulation with tumor targeting capability of such micelles	(285)
Micelles of polyphosphoester-conjugated camptothecin prodrug containing disulfide bond (PBYP-*g*-ss-CPT)-*b*-PEEP	CuAAC	Campto-thecin (CPT)	Intracellular uptake of prodrug micelles efficiently releases CPT into HepG2 cells. Prodrug micelles efficiently inhibited proliferation of HepG2 cells.	(286)
Micelles of disulfide-containing dextran-CPT (Dex-ss-CPT) and hydrazone-containing dextran-DOX prodrug	CuAAC	CPT, DOX	Combination of micelle-bound prodrugs exhibited excellent anticancer activity with reduced toxicity as compared to the free drugs *in vitro* and *in vivo*	(287)
Micelles of polyphosphoester-camptothecin prodrug (P(EAEP-PPA)-*g*-ss-CPT)	CuAAC	CPT	Good cytocompatibility of P(EAEP-PPA), not only for normal cells, but also for tumor cells. Prodrug micelles efficiently release CPT into 4T1 or HepG2 cells and inhibit cell proliferation	(288)
Micelles made of PEG)-*b*-poly(5-methyl-5-propargyl-1,3-dioxan-2-one) grafted with reduction-sensitive paclitaxel prodrug (PEG-*b*-PMPMC-*g*-PTX)	CuAAC	PTX, DOX	Dual drug-loaded micelles exhibited significant growth-inhibition for HeLa cells and drug-resistant MCF-7/ADR cells compared with the single drug-loaded nanoparticles, especially for drug-resistant tumor cells.	(289)
			DOX-loaded micelles exhibited synergistic effect of cell-growth inhibition on HeLa cells	
Micelles of pseudopoly(amino acid) copolymer containing PEG end blocks (mPEG-HRSCP-mPEG)	CuAAC	DOX	DOX-loaded NPs internalized into HeLa cells through an endocytosis mechanism, and displayed significant anticancer efficacy	(290)
Micelles of poly(ε-caprolactone) and poly(oligoethylene glycol methacrylate) block copolymer (PCL-SS-POEGMA)	CuAAC	DOX	Nanomicelles demonstrated good therapeutic efficiency to kill cancer cells	(291)
Micelles of cyclodextrin, polycaprolactone, PEG), poly(NIPAM) triblock copolymer (CD-PCL-SS-PEG(PNIPAM))	CuAAC	DOX	High cellular uptake efficiency and significant intracellular drug release using DOX-loaded micelles via controlled temperature or reduction, with good therapeutic efficiency	(239)
CCPMs of disulfide-containing (CPT)-poly(tyrosine(alkynyl)-OCA) conjugate, CH_3_O-PEG-*b*-poly(tyrosine(alkynyl)-OCA) diblock polymer and bis(azidoethyl) disulfide cross-linker	CuAAC	CPT	Presence of disulfide bonds in micelles induced redox-responsive drug release, and enhanced cytotoxicity against human breast cancer cells (MCF-7) *in vitro* compared to free drugs.	(292)
Micelles made of PEG-*block*-poly(g-propargyl-l-glutamate) (PEG–PPLG) copolymer	CuAAC	DOX	Intracellular drug release into HeLa cells from DOX-loaded micelles was increased with an enhanced intracellular glutathione level.	(293)
			*In vitro*, DOX-loaded micelles were biocompatible, and showed higher cellular proliferation inhibition toward GSH-pretreated HeLa cells compared to nonpretreated cells	
Core-cross-linked micelles of poly[2-(2-methoxyethoxy)-ethyl methacrylate)-*co*-(*N*-methacryloxy succinimide)]-*block*-poly(*N*-(2-hydroxypropyl) methacrylamide) ((P(MEO2MA-*co*-MASI)-*b*-PHPMA) conjugated with cisplatin and cypate	CuAAC	Cisplatin and cypate	Drug-loaded micelles showed redox-responsive cross-linker cleavage and cisplatin drug release in the presence of reductants. Irradiation (805 nm) leads to temperature increase and ROS generation. Significant synergistic effects of combined photothermal therapy and chemotherapy against cisplatin-resistant human lung cancer cells A549R.	(294)
Core-cross-linked micelles of PEG)-*b*-poly(5-methyl-5-propargyloxycarbonyl-1,3-dioxane-2-one) [PEG-*b*-poly(MPC)] amphiphilic block copolymer and bis(azidoethyl) disulfide as cross-linker	CuAAC	DOX	Efficient uptake and low cytotoxicity of micelles in HeLa cells and 4T1 cells (MTT assay). With drug-resistant ADR/MCF-7 cells, drug-loaded micelles displayed enhanced cytotoxicity.	(295)
Nanoparticles of peptide-based dendronized heparin-DOX conjugate	CuAAC	DOX	Nanoparticles resulted in strong antitumor activity, high antiangiogenesis and induced apoptosis on 4T1 breast tumor model.	(281)
CH_3_O-PEG-modified peptide dendrimer conjugate with DOX	CuAAC	DOX	Conjugation of DOX to dendrimer leads to significantly high antitumor activity, specifically induced apoptosis on 4T1 breast tumor model	(296)
Dendrimer-CPT prodrug of polyamido-amine-PEG and CPT	CuAAC	CPT	CPT prodrug displayed dose-dependent toxicity (IC_50_ = 5 μM), a 185-fold increase relative to free CPT.	(297)
			Conjugated CPT resulted in G_2_/M arrest and cell death, while PEGylated dendrimer without CPT displayed minimal toxicity.	
β-Cyclodextrin-based dendrimer	CuAAC	Metho-trexate (MTX)	MTT test on T47D cells showed MTX-loaded dendrimer showed slightly lower IC_50_ to kill cancer cells than free MTX	(298)
Hydrogel of DBCO-modified PEG and benzyl azide-modified PEG	SPAAC	DOX	DOX-loaded hydrogel system showed a drastic reduction in cell viability of MDA-MB-231 cells (25% survival versus 80+% survival for bare hydrogel)	(299)
CCPMs prepared of PEG-*b*-poly(furfuryl methacrylate) block copolymer	Diels–Alder	DOX	Block copolymer not cytotoxic for normal HEK293 cell line	(300)
			DOX-loaded CCL micelles exhibited high antitumor activity toward HepG2 cells	
Core cross-linked nanoparticles (CCL NPs) made copolymer of PTX-poly(butynyl phospholane)-*block*-poly(ethylethylene phosphate) (PTX-PBYP-*b*-PEEP)	Thiol–yne click reaction	Paclitaxel (PTX), DOX	Efficient internalization of CCL NPs within HeLa cells, and dual pH and redox responsive behavior, with efficient DOX and PTX release under tumor microenvironment, leading to more efficient apoptosis compared to free drug	(301)
Micelles of PEG-poly(butylene mercaptosuccinate) block copolymer	Thiol–ene	PTX	CCK-8 assay revealed that reduction-sensitive PTX-loaded micelles showed higher antitumor activity toward HeLa cells compared to reduction-insensitive counterparts and free PTX	(302)
Heparin-based dendrimer	Thiol–ene	DOX	DOX-loaded dendrimer efficiently killed SKOV-3 ovarian cancer cells, whereas bare dendrimer displayed minimal cytotoxicity.	(281)
Micelles of triblock CH_3_O-PEG-hydroxyl-terminated polybutadiene-PEG-OCH_3_ (MPEG-HTPB-MPEG) copolymer	Thiol–ene	Ibuprofen (IBU)	*In vitro* drug release <10.0% IBU under artificial gastric fluid after 2 h, but immediate release under artificial intestinal fluid. XTT assay showed cytocompatibility of the micelles with epithelial cells displaying potential as oral drug delivery carrier	(303)
Micelles made of PEG)-poly(acetal urethane)-PEG) triblock copolymer	Thiol–ene	DOX	DOX-loaded micelles showed high *in vitro* antitumor activity in both RAW 264.7 and drug-resistant MCF-7/ADR cells, via micelle-mediated cytoplasmic DOX delivery	(304)
Micellar system of PEG-oxime-tethered poly caprolactone- PEG (PEG-OPCL-PEG) triblock copolymer	Oxime click reaction	DOX	DOX release at pH 5.0; DOX-loaded micelles easily internalized by living cells; MTT assay against HeLa cancer cells showed DOX-loaded PEG-OPCL-PEG micelles had high anticancer efficacy	(305)
Matrix metalloproteinase-2 -sensitive cleavable peptide and hyaluronic acid-modified poly amidoamine dendrimer	Oxime	DOX	DOX-loaded dendrimer showed excellent tumor-penetrating efficiency and significant antitumor activity both *in vitro* and *in vivo* compared to free DOX.	(306)
Micelles made of PEG, poly(ε-caprolactone) block copolymer (PEG-*b*-PCL)	Amino-yne	DOX, Chlorin e6 (Ce6)	Drug-loaded micelles efficiently deliver DOX and Ce6 within cancer cells; high therapeutic effect when the cells were subjected to light. ROS-assisted bond cleavage induced the maximal therapeutic effect by specifically releasing DOX into cytosol	(307)
Hydrogel of PAA, norbornene-functionalized chitosan and bistetrazine-poly(*N*-isopropylacrylamide)	IEDDA	5-amino-salicylic acid	Biodegradable hydrogels, nontoxic against human fibroblast cells. Considerable potential for a colon-targeted drug delivery system, and for oral insulin delivery.	(308)
Hydrogel of norbornene-functionalized car-boxy-methyl cellulose and diselenide based-PEG cross-linker possessing two terminal tetrazine moieties	IEDDA	DOX	Hydrogel demonstrated stimuli-responsive and fast release of DOX (99% after 12 h) in presence of 10 mM of glutathione, as compared to the normal PBS solution (38% DOX release); hydrogel not cyctotoxic against HEK-239 cells, whereas DOX-loaded hydrogel showed anticancer activity in BT-20 cancer cells	(309)
Hydrogel of norbornene-bearing alginate-norbornene and PEG- based disulfide cross-linker with two terminal tetrazine moieties	IEDDA	DOX	Hydrogels released <90% loaded DOX in presence of 10 mM glutathione in 11 d, whereas <35% release was noticed in physiological buffer (PBS, pH = 7.4) after 11 d; DOX-loaded hydrogel displayed anticancer activity toward HeLa cells, whereas hydrogel itself did not show cytotoxicity	(310)
Hydrogel of hydrazide-grafted hyaluronic acid (HA-ADH) and aldehyde-modified dextran (Dex-ALH)	Schiff-base reaction	Dexa-methasone acetate-loaded ROS-erasable PEG)-*b*-polythio-ketal-*b*-PEG) (PEG-PTK-PEG) (PDM) micelles	The PDM gave the hydrogel improved antioxidant capacity, which gave the drug a longer retention time and sustained release; injected hydrogel inhibited inflammatory cytokines, and downregulated the ratio of pro-inflammatory M1 macrophages	(311)

### 3.2 Antiviral Polymers

Since its discovery, click chemistry has been frequently used in virus-related research and applications, such as viral tracking, design of antiviral agents, as well as virus-based delivery systems. Most of these applications focused on either modifications of organic small molecules or of inorganic nanoparticles, or on engineering the virus outer layer with small organic molecules using click reactions. In contrast, there are only a few research articles that focus on click-based polymers for virus-related applications. However, research into the delivery of the Covid RNA vaccines, which led to the now used ionizable cationic lipids that form lipid nanoparticles as drug delivery systems, shows the vital importance of such work, and the need for further improvements, both in view of improved loading of the vaccines and potentially further reduction of any immune response to the drug delivery system itself.^51,312^ Therefore, it has become evident that there is not just the need but also a huge opportunity for biofunctionalized polymers for virus-related applications, particularly as antiviral agents.

From the discovery of viruses to today, scientists have encountered the complexity of viral infections, their mutations, and frequent lack of solid proof of interaction between the virus and host cells. Viruses are the prime cause of several life-threatening diseases, including AIDs, influenza, hepatitis, gastroenteritis, COVID19, and monkey pox. To protect from viral infections and diseases, researchers, virologists, and clinicians are continuously working on the development of novel antiviral agents that can modulate our immune system so as to protect our bodies against viral infections without causing any damage to our bodies. This is complicated as by evolutionary pressure a significant percentage of viruses display a progressive resistantance to the presently available antiviral agents in the market. So, there is an urgent and ongoing need for the development of novel antiviral agents with high efficacy, and click chemistry-based functional polymers can add to the toolbox toward efficient platforms for novel antiviral agents.

**Figure 49:**
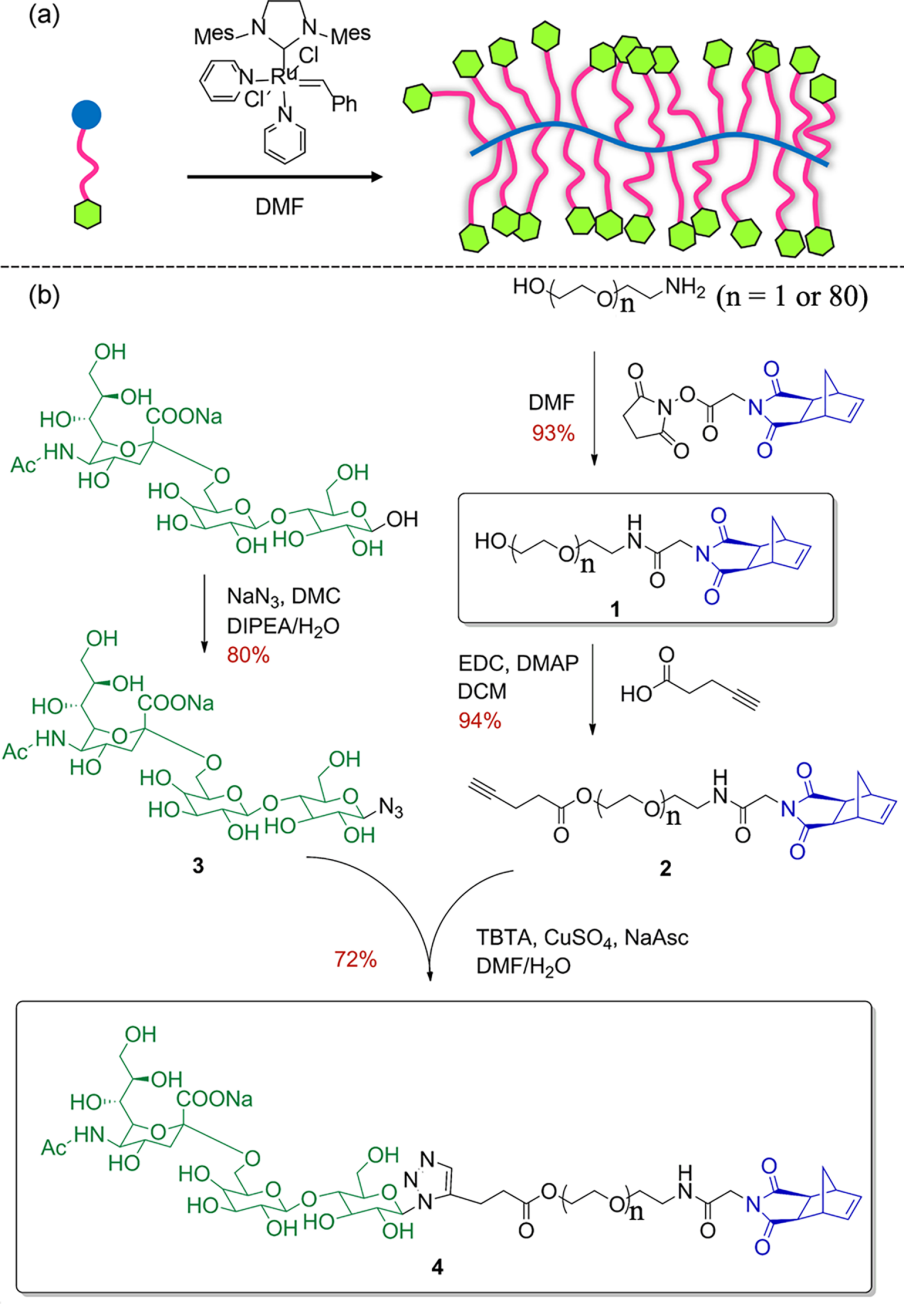
Antiviral click chemistry-based polymers. (a) Preparation of bioinspired brush polymers containing terminally attached sialic acids via ring-opening metathesis polymerization. (b) Synthetic route to norbornenyl (in blue) macromonomers containing sialic acids (in green). Adapted with permission from ref (315). Copyright 2016, ACS.^315^

A good illustration can be given with antiviral agents that hinge on click-based functional polymer systems against influenza infection. Aminidase and hemagglutinin are the most significant antigenic determinants that are present on the outer surface of the influenza virus. Thus, aminidase and hemagglutinin are among the prime targets for the design of anti-influenza agents. For instance, Miura and co-workers developed a sialyl oligosaccharide-modified glycopolymer using CuAAC for the recognition and inhibition of the influenza virus.^313^ Among their synthesized glycopolymers, one (GP4-6′) showed promising antiviral activity (IC_50_ value = 320 μM) along with specific recognition. More interestingly, they noticed that clicking multivalent antibodies or ligands within the polymer structure enhances the interaction of polymers with a virus, leading to an improved antiviral activity. Using such multivalency, Yang et al. designed S-sialoside human serum albumin (HSA)- and bovine serum albumin (BSA)-modified glycopolymers using CuAAC.^314^ The multivalent system exhibited excellent hemagglutinin blocking activity, as well as moderate amidase inhibition activity. Furthermore, these glycoconjugates did not exhibit any major cytotoxicity on HUVEC cells, which suggested its potential application toward an anti-influenza agent. Analogously, Olsen and his team members also reported antiviral agents based on multivalent brush polymers modified with sialyl oligosaccharides using CuAAC.^315^ To control activity, they varied the degree of polymerization, the number density of sialic acids, and the length of side chains in the brush polymers (see Figure 49 for synthesis). Subsequent hemagglutination inhibition assays and *in vitro* infection assays revealed that optimization of the three-dimensional sialoside spacing plays a critical role in the multivalent binding of hemagglutinin trimers and subsequent antiviral activity of the polymer system.

Tian et al. also reported a series of multivalent pentacyclic triterpene-functionalized per-O-methylated cyclodextrin systems for inhibition of the influenza virus.^316^ Using CuAAC to conjugate the triterpene derivative with a cyclodextrin, they synthesized a range of compounds, one of which displaying an anti-influenza activity with IC_50_ = 4.7 μM. Interestingly, a time-of-addition assay revealed that this compound restricted the entry of influenza virus into a host cell. Further hemagglutination inhibition and SPR assays indicated that this compound was tightly bound to influenza HA protein (*K*_dissociation_ = 4.0 μM), making it a potential antiviral lead against influenza. Similarly, Wolff and co-workers synthesized a multivalent sialylated polyglycerols system using CuAAC that showed a broad-spectrum antiviral activity against influenza A viruses, especially when specifying the inhibitor functionalization to 2,6-α-sialyllactose (SL) and using a heterobifunctional 3.4 kDa acetal-PEG-NHS linker.^317^

**Figure 50:**
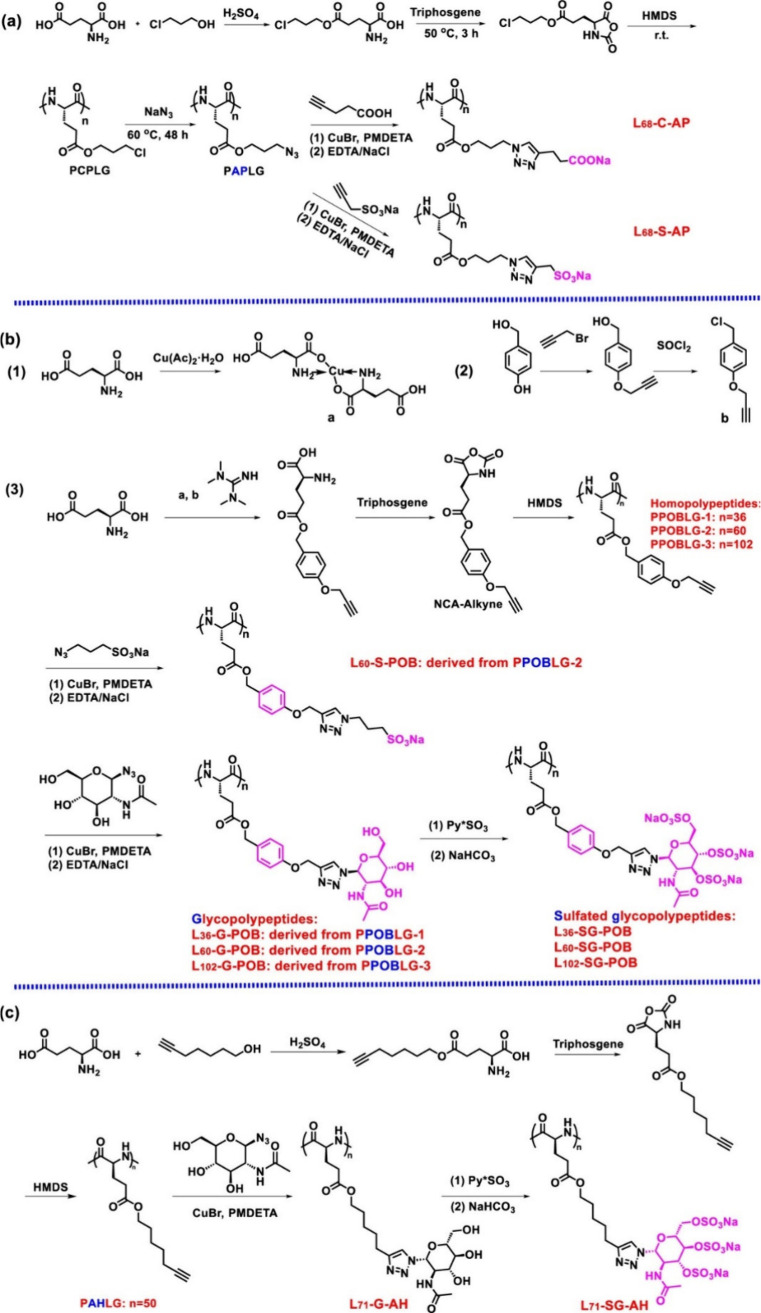
Synthesis and structures of polypeptides for antiviral application against SARS-CoV-2. Adapted with permission from ref (327). Copyright 2023, ACS.^327^

Hepatitis is another life-threatening virus that causes inflammation of the liver and ultimately leads to liver cancer if not diagnosed and treated within an appropriate time. It has several variants including hepatitis A, B, C, D, and E. Among all these variants hepatitis B and C are the most dangerous ones. A recent survey reported that hepatitis B and C cause 1.4 million deaths per year, i.e., more than HIV/AIDS and/or malaria. Currently, the key target for the treatment of chronic hepatitis C virus (HCV) is the entry of the virus into our bodies. In this regard, Xiao et al. synthesized a series of α- or β-cyclodextrin pentacyclic triterpene-based anti-HCV entry agents functionalized with water-soluble triazole groups using CuAAC.^318,319^ Their results revealed that several α-cyclodextrin derivatives possess good anti-HCV entry activities at the postbinding step, with IC_50_ values down to 0.25 μM.^319^ To address the unsolved issues related to HCV treatment such as high cost and genotype dependency, Bronich and co-workers, using CuAAC, synthesized direct-acting antiviral agents based on galactosylated peptide nanocomplexes.^320^ These nanocomplexes exhibited specific internalization in hepatoma cell lines *in vitro*, suppressed the intracellular expression of HCV proteins *in vitro* (see Figure 50), and yielded preferential liver accumulation *in vivo*, all in all suggesting the power of such a antiviral multivalent click approach. In a similar study, Brathelemy and co-workers also demonstrated that the conjugated compound can have a strong antiviral effect; by synthesizing a lipid-conjugated oligonucleotide using CuAAC that can successfully inhibit HCV translation.^321^ They found that the lipid conjugation significantly increased the cellular uptake, causing these nontoxic lipid conjugates to efficiently inhibit the hepatitis C virus internal ribosome entry site (IRES)-mediated translation in human hepatic Huh7 cells.

Other than against influenza and hepatitis C virus, antiviral agents to tackle human immunodeficiency virus (HIV) remain a significant challenge, partially due to the rise in the nonnucleoside reverse transcriptase inhibitor resistance of HIV.^322,323^ To overcome this problem, Liu and co-workers used CuAAC to conjugate a nonspecific antiviral sapogenin with a specific HIV fusion inhibitor that has a helix zone-binding domain (HBD)-containing the T20 peptide (enfuvirtide).^324^ They found that neither the sapogenin nor the peptide part alone can deliver strong activity against HIV-1 Env-mediated cell–cell fusion, while the hybrids generated a strong synergistic effect. Among their synthesized HBD-containing peptides, P26-BApc exhibited promising anti-HIV-1 activity against both T20-sensitive and T20-resistant HIV-1 strains and interesting pharmacokinetic properties. Arnaiz and co-workers reported the synthesis of anionic carbosilane dendrimers via CuAAC, and evaluated their antiviral efficacy against HIV.^325^ Briefly, they synthesized four families of anionic carbosilane dendrimers bearing carboxylate, phosphonate, naphthylsulfonate, and sulfate terminal groups using CuAAC. These anionic dendrimers did not exhibit any cell toxicity *in vitro* until a concentration up to 20 μM. While the phosphonate and carboxylate dendrimers yielded no and intermediate effects, respectively, the sulfate and naphthylsulfonate group-bearing dendrimers showed significant anti-HIV activity in a dose-dependent manner. Analogous anionic carbosilane dendrimers were used by Galan et al. and functionalized using thiol–ene click chemistry instead of the CuAAC reaction.^326^ Interestingly, they found a strong dependence of the antiviral actions on the dendrimer: where a silicon-core dendrimer showed 85–90% HIV inhibition without any host response in mice, an analogous polyphenoxo core did not display any promising results.

In a recent study conducted by Zheng et al., a comprehensive collection of sulfated glycomimetic polypeptides was synthesized with a significantly elevated sulfated degree, reaching up to 99%. This was achieved by the use of a CuAAC click reaction and subsequent sulfation modification. The primary objective of this research was to investigate the potential of these polypeptides as powerful antiviral agents against SARS-CoV-2.^327^ In order to produce polypeptides with varying side chains, the researchers used a combination of *N*-carboxylanhydrides (NCA), ring-opening polymerization (ROP), the CuAAC click reaction, and *O*-sulfation modification techniques. The various side chains included carboxylate, sulfonate, glycosyl, and sulfated glycosyl (see Figure 50 for the synthesis). *In vitro* experiments indicated that both the α-helical conformation and the presence of sulfated sugar are important factors in the inhibition of SARS-CoV-2. This is supported by the improved performance shown in all sulfated glycopolypeptides in terms of their ability to suppress a SARS-CoV-2 infection. The level of inhibitory effectiveness achieved was as high as 85%. Among the sulfated glycopolypeptides, it was observed that L_60_-SG-POB had the most significant inhibitory effectiveness, as shown by its IC_50_ value of 0.71 μg/mL, and an inhibitory efficacy of as high as 86% against enteroviruses.

In summary, one can note that the unique bioorthogonality of click chemistry provides access to a wide range of novel polymer-based antiviral agents. In the search for better drug delivery systems for intravenous, pulmonary or other forms of administration (such as microneedles), and for antiviral activity itself, it is hoped that polymer-based click-functionalized systems can contribute to the development of improved antiviral drugs. It is noted that up to now predominantly CuAAC has been used, likely given its long-known compatibility with sugar chemistry,^118^ but that the triazole unit itself seems not involved in its action, thereby opening the way to other, metal-free click chemistries to add to this field. In addition, zwitterionic polymer brushes originally made for their antifouling behavior were also shown to be effective against both SARS-Cov-2 and the avian flu virus.^328^ Such brushes have been shown to be highly amenable to further functionalization via a range of click chemistries,^272^ and as such would provide options for further optimization and dual-action modes of operation.

### 3.3 Biosensors

A biosensor is an analytical device used to detect biologically important chemical substances such as biomarkers (antibodies, enzymes, nucleic acids, etc.) or living cells (microorganisms, specific types of cells, etc.) with the help of a physicochemical detector.^329,330^ Such detection can be based on, e.g., electrochemical,^331,332^ optical,^333,334^ or field-effect transistor (FET)^335^ phenomena. This field has made significant progress by taking advantage of many novel materials that are specifically designed for biosensing applications. These novel materials include various functionalized inorganic nanoparticles,^336^ carbon-based nanomaterials (graphene oxide, carbon dots, etc.),^337^ inorganic quantum dots^338^ and polymeric materials.^339,340^ Polymeric materials have some advantages over other types of materials used in the biosensor because of their being lightweight, ultraconformable (bendable, stretchable, foldable), portable, disposable, highly sensitive, and (for electrical measurements) responsive to a small applied potential, which makes them extremely useful materials in real-time applications.^341,342^ To design novel polymeric biosensors “click chemistries” can take a crucial role due to their flexibility and minimal workup procedure. However, this field is still emerging and not many studies are reported on click-based functional polymers for biosensing applications. The next part summarizes these developments.

Yagci and co-workers developed an electrochemical glucose biosensor based on polyaniline (PA) grafted PEG (PA-*g*-PEG) using a combination of oxidative polymerization and CuAAC click chemistry.^343^ The synthesis of PA-*g*-PEG involves two steps: First, Cu(II) is reduced to Cu(I) during the oxidative copolymerization of aniline and aminophenyl propargylether. This Cu(I) catalyzes the CuAAC reaction between alkyne-functionalized polyaniline and independently prepared azide-functionalized PEG. The combined electrostatic polyanion, polycation, and hydrogen bond interactions yield strong bonding between PA-*g*-PEG and glucose oxidase (GOx), which is advantegous given the high water solubility of this PA-*g*-PEG. As a result, this polymer can function as an excellent immobilization matrix for the enzyme, resulting in outstanding analytical characteristics with a linear range of 0.05–1.0 mmol L^–1^ (*R*^2^ = 0.9971), and sensitivity down to 47.72 μA mM^–1^ cm^–2^. Additionally, the PA-*g*-PEG-GOx biosensor was fast, stable (up to 12 days) and displayed highly reproducible data in a series of measurements. Samitier and his team members fabricated a label-free electrochemical DNA sensor using CuAAC-functionalized PEDOT electrodes.^344^ For the fabrication of the sensor, they immobilized an acetylene-terminated DNA probe, complementary to a specific Hepatitis C virus sequence onto azide-modified, conducting PEDOT electrodes using CuAAC chemistry. Using differential pulse voltammetry a promising limit of detection of 0.13 nM for the specific HCV-target sequence was reached along with a high selectivity. In another work, Kong and co-workers reported a hairpin probe rearrangement for label-free electrochemical detection of human T-lymphotropic virus types II (HTLV-II).^345^ To construct the biosensor, azide-terminated hairpin DNA probes were first attached to a gold electrode surface by an S–Au bond, bringing the azido terminals of hairpins close to the electrode surface, where they were unreactive toward subsequent functionalization. In contrast, after hybridizing with HTLV-II, specific unfolding of the hairpins was achieved, which made the azido termini of the hairpins available to react with an alkynyl-containing polymer via CuAAC. This process yielded a good electrochemical signal with excellent signal amplification, down to a limit of detection of 0.17 pM, with a good linearity in the range of 1 pM–1 nM, and application of virus detection in real serum samples. Leal et al. reported a polydiacetylene (PDA)-based “click glycoliposome” biosensor for the specific optical detection of lectins (based on color changes of PDA upon conformation changes).^346^ For the synthesis of the biosensor, they employed a regioselective CuAAC click ligation of the triacetylenic *N*-(2-propynyl) pentacosa-10,12-diynamide with a variety of mannose- and lactose-tethered azides. After the topochemical polymerization of the diyne-moieties that yields PDA-based clicked glycoliposomes, this material displayed rapid colorimetric transitions upon binding to lectins. This visual change of color in the presence of analytes gives wider accessibility of these type of sensor in terms of on-site recognition without any delay or the use of any high-end instrument. Hoven and co-workers developed a clickable and antifouling platform of poly[(propargyl methacrylate)-*ran*-(2-methacryloyloxyethyl phosphorylcholine)] (p-PgMAMPC) for biosensing of streptavidin (SA) and mismatched DNA.^347^ To synthesize this copolymer, they employed a combination of CuAAC-based conjugation and RAFT polymerization. Briefly, they reacted an alkyne moiety of propargyl methacrylate (PgMA) with azide-containing molecules (biotin-conjugated azide and peptide nucleic acid-conjugated azide) via a click reaction, followed by copolymerization of the resulting biofunctionalized methacrylate with 2-methacryloyloxyethyl phosphorylcholine (MPC) as hydrophilic monomeric unit, in which the MPC strongly diminishes the nonspecific adsorption of biomolecules. Furthermore, to make the biosensor platform SPR-active, they immobilized the copolymer onto a gold-coated substrate using a grafting-to method via Au–S bond formation between thiol end groups of the copolymer and the gold surface. Targeted detection of streptavidin (SA) in 0.14% blood plasma was achieved with a biotin-conjugated p-PgMAMPC copolymer by SPR with a detection limit of 0.95 nM. The peptide nucleic acid-conjugated copolymer detected the highest amount of complementary DNA sequence, with 71% hybridization efficiency, and a reasonable degree of mismatch discrimination (58%MD) between the fully complementary DNA and single-mismatched DNA.

**Figure 51:**
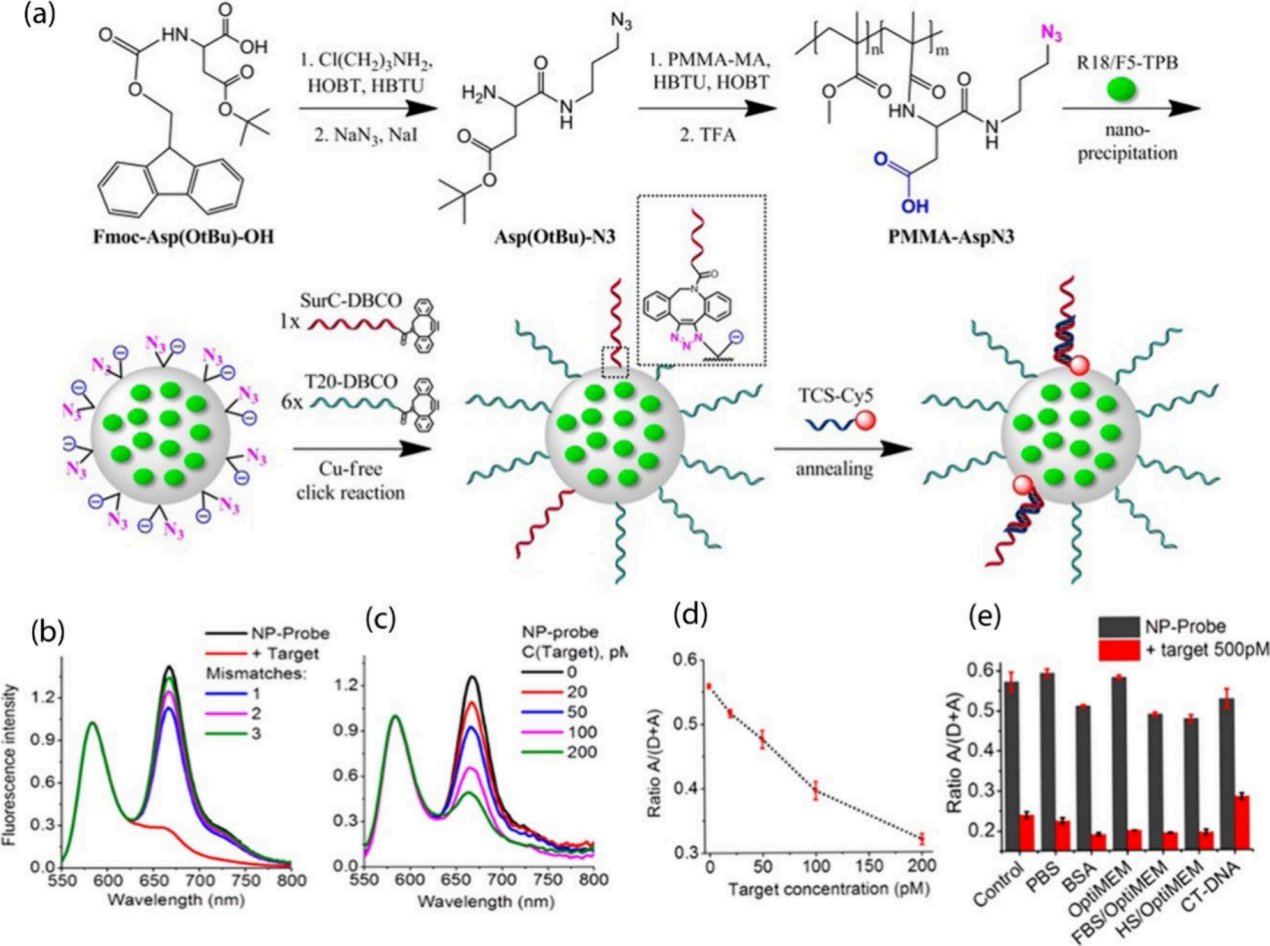
(a) Synthesis of nanoprobes of nucleic acid using a SPAAC reaction followed by nanoprecipitation. (b-e) Detection of targeted nucleic acid: (b) Fluorescence spectra of nanoparticle (NP)-probe (100 pM of TCS-Cy5) after incubation for 20 h at 4 °C without and with survivin nucleic acid target (1 nM). Fluorescence spectra (c) and values of FRET response A/(A + D) (d) of NP-Probe after incubation with the target at different concentrations. (e) FRET response of the NP probe (100 pM of TCS-Cy5) to the NA target (500 pM) in different biological media (concentration of Mg^2+^ was adjusted to 12 mM). Adapted with permission from ref (348). Copyright 2018, ACS.^348^

Melnychuk et al. reported a DNA-functionalized dye-loaded polymeric nanoparticle-based fluorescence biosensor for the amplified detection of nucleic acids.^348^ To design the biosensor, they first modified carboxylic group containing poly(methyl methacrylate-*co*-methacrylic acid (PMMA-MA) with azide group, then the resultant, PMMA-MA-N_3_ convert into the azido-group bearing NPs after the nanoprecipitation and further reacted with an oligonucleotide-bearing dibenzocyclooctyne (DBCO) unit using SPAAC. This was followed by annealing with a shorter target-competitive sequence-bearing FRET acceptor Cy5 (TCS-Cy5) (Figure 51a).

In the sensor system, functionalized polymer nanoparticles (containing ∼3200 rhodamine molecules) acted as an energy donor, where hybridization of the analyte nucleic acid (encoding a survivin cancer marker) with ca. 23 grafted oligonucleotides/Cy5-acceptors regulates on/off FRET from of the nanoprobe. This led to a dose-dependent, sequence-specific two-color ratiometric response to the presence of specific nucleic acids, with a limit of detection of 0.25 pM (Figure 51b–d). Moreover, the biosensor is capable of recognizing mismatched nucleic acids in different biological media including PBS, FBS, BSA, etc. with high sensitivity (Figure 51e).

Shuo and co-workers developed a novel molecular imprinting fluorescent biosensor for the ultrafast detection of glycoproteins.^349^ To fabricate the sensor, first they synthesized a thiol-bearing polymer made of *N*-(2-disulfanylethyl)-2-methylprop-2-enamide followed by a thiol–ene click reaction with 4-vinylphenylboronic acid. The incorporation of 4-vinylphenylboronic acid improves the imprinted recognition of glycoproteins and converts recognition behavior into a fluorescent signal in a single step. Furthermore, the sensor exhibited a good linear response within the range of 0.1–10 mg mL^–1^, which confirms its applicability over a wide range of detection. On a different note, Yamaguchi and co-workers in 2023 reported a membrane-based colorimetric immunosensor that exhibits great sensitivity and versatility. This sensor is designed for the detection of interleukin-6 (IL-6) and utilizes an SPAAC click reaction.^350^ IL-6 is a versatile cytokine that has significant influence on several physiological processes, including immunological responses, inflammation, bone metabolism, and reproduction. The sensor was designed by reacting azide-functionalized poly(glycidyl methacrylate) (PGMA) with cyclooctyne-modified antibodies by the use of strain-promoted azide–alkyne cycloaddition (SPAAC) chemistry. The sensor has a high efficiency in detecting IL-6 at concentrations of up to 1000 pg/mL, while exhibiting a detection limit of 4.5 pg/mL. Similarly, Fenoy et al. used the technique of strain-promoted azide–alkyne cycloaddition (SPAAC) to develop a biosensor for detecting thrombin, using a polymeric electrochemical transistor.^351^ The researchers utilized an azide-modified thrombin-sensitive aptamer (HD22) and poly-l-lysine derivatized by dibenzocyclooctyne (PLL-DBCO) to create the biosensor. The analyte is effectively detected by the thrombin-sensitive HD22-PLL-DBCO-functionalized organic electrochemical transitor, exhibiting a dissociation constant *K*_D_ of 88 ± 20 nM and a LOD of 22 nM, in agreement with earlier findings.^352,353^

From the above studies, it is clear that click-based polymers have great potential to act as active components in novel biosensors for real-life clinical applications. The variety of topics mentioned compared to the number of topics that can be studied in this manner suggests that there is much room to investigate and improve here, so as to contribute to enhanced sensing with increased access options (faster and more economical route to the market). Below we have enlisted a few more click-based polymers for biosensing applications, that further illustrate the current state of the art. In Table 2, below we have summarized the use of additional state-of-the-art click based polymers for biosening applications.

**Table 2:** Click-Based Polymers as Active Component of Biosensors

Sensing element/Analyte	Sensor type	Click chemistry	Polymeric platform	Limit of detection (LOD)	Detection range	Ref.
DNA	Electrochemical	CuAAC	Chloromethyl- and azidomethyl-substituted PEDOT	–	0.2–4.8 nM	(354)
Sequence-specific DNA	Fluorescence	CuAAC	Surface-bound hairpin DNA-modified with fluorescein o-acrylate polymers	4.3 × 10^–15^ (M)	1 × 10^–13^–1 × 10^–6^ (M)	(355)
*S. aureus* and *E. coli*	Electrochemical	CuAAC	Ferrocenylmethyl metharylate (FMMA) polymer	4–6 CFU/mL	10^2^ to 10^7^ CFU/mL	(356)
Tobacco mosaic virus	Electrochemical	CuAAC	hairpin DNA	3.5 fM	0.1 pM–10 nM	(357)
l-Cysteine, glutathione, d-penicillamine	Photoelectrochemical	Thiol–yne	Poly(1,3,5-triethynylbenzene)	0.3 μM (l-Cys), 1.1 μM (glutathione), 0.4 μM (d-penicillamine)	10–2000 μM (l-Cys), 10–1200 μM (Glutathione), 10–2000 μM (d-penicillamine)	(358)
Carcino-embryonic antigen	Electrochemical	SPAAC	PEDOT functionalized with antibody that was clicked onto antifouling peptide	40 fg/mL	40 fg/mL–700 ng/mL	(359)
Tetracycline	Fluorescence	Thiol–ene	Silk fibroin-functionalized thiol-branched graphene oxide quantum dots	45–55 nM for range of complex biofluids, incl. human serum	up to 90 nM	(360)

### 3.4 Bioimaging

In recent years, modern biology has focused with increasing detail on understanding how components of living cells are organized to form complex systems, how they interact with each other, what changes take place at the cellular level when our body is affected by any diseases, and—when treated with drugs/genes/any other biomolecules—in which organs these molecules accumulate and how they are excreted from our body.^361−363^ All of these phenomena can be identified and visualized at various levels of resolution by bioimaging techniques. The primary and foremost step of any bioimaging method is to specify the target that is to be imaged. Second when a target is selected the next step is to develop techniques to image that target *in vitro* and *in vivo*. This process involves the administration of bioimaging probes with specific physical, chemical, and biological properties that render the agent visible to imaging systems but uninvasive in every other sense to the biological system. Currently, most of the bioimaging agents used for clinical applications are small molecules that are directly used for bioimaging or labeled with specific chemical moieties according to the imaging modality such as fluorescent probes for optical imaging, paramagnetic agents for magnetic resonance imaging (MRI), radiolabeled probes for positron emission tomography (PET), or acoustically active microbubbles for photoacoustic imaging.^364,365^ Most of these imaging agents show promising results both *in vitro* and *in vivo*, but they also display limitations. In most of the cases these small-molecule bioimaging probes display limited stability upon prolonged measurements under normal temperature and physiological conditions, are nonspecific to the target of interest and rapidly cleared from the body, and sometimes also cytotoxic. In addition to that, the often limited functionality of small bioimaging probe molecules restricts modification that could improve the specificity for the target.^366^ Moving from individual sensing elements to polymeric ones may thus improve both ease of use, sensitivity (multiple probes per polymer chain) and specificity (multiple targeting moieties per polymer chain). In this section we will first discuss optical bioimaging, and subsequently PET and MRI.

**Figure 52:**
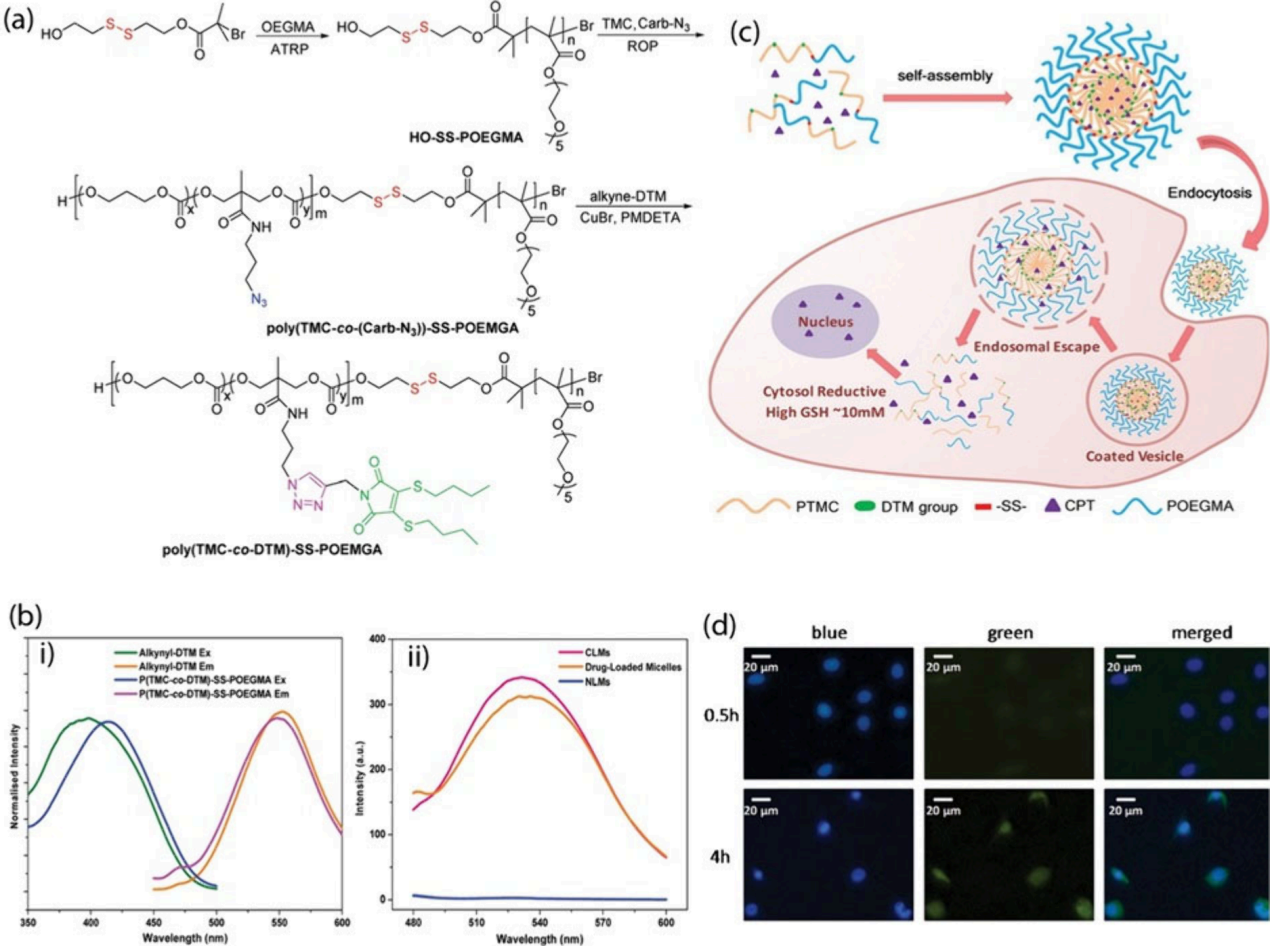
(a) Synthetic routes of the poly(TMC-*co*-DTM)-SS-POEGMA amphiphilic block copolymers. (b) Fluorescence spectra of the alkyne-DTM monomer and the poly(TMC-*co*-DTM)-SS-POEGMA block copolymer. (c) Schematic illustration of CPT-loaded CLMs and in vitro release of CPT. (d) Fluorescence images of A549 cells after incubation of CLMs for 0.5 and 4 h. Adapted with permission from ref (371). Copyright 2017, RSC.^371^

In the last two decades, polymeric materials have emerged as a highly promising class of materials for bioimaging for their comparative ease of synthesis, prolonged plasma half-lives, enhanced stability, good biocompatibility and targeting capability.^366^ In this regard, click chemistry provides a unique platform to synthesize specifically functionalized novel polymeric bioimaging probes. As an early example, Yang et al. reported a water-soluble PEG-functionalized-iridium complex using CuAAC click chemistry for cellular bioimaging.^367^ In this, they reacted an azide-modified PEG with an alkyne-functionalized water-soluble iridium(III) complex to form a phosphorescence-active polymer. The polymer demonstrated bright red phosphorescence (λ_max_ = 625 nm; quantum yield = 1.4%) in a PBS solution. Additionally, the polymer exhibited low cytotoxicity and good membrane permeability, which led to exclusive staining mainly in the cytoplasm of KB cells (human epithelial carcinoma cells) that was retained for a long time. Another early work, by Liu and co-workers, reported a fluorescent core–shell nanosphere of hyperbranched conjugated polyelectrolyte (HCPE) for live-cell bioimaging.^368^ To design the fluorescent active polymer, they chose hyperbranched cationic polyfluorene (PF) as the stable light-emitting center of the HCPE, whereas linear PEG was selected for the periphery of the HCPE to passivate the macromolecular surface and to improve the overall cytocompatibility. To conjugate the inner PF core and outer PEG periphery, CuAAC was chosen over the Pd-catalyzed coupling reactions that was used for the synthesis of the PF core, due to the drawback of poorly controlled growth of the propagating species. To check the applicability of this HCPE in biological imaging, they treated human breast cancer cells (MCF-7) with HCPE nanospheres. Confocal laser scanning microscopy (CLSM) images revealed that HCPE nanospheres are capable of good cytoplasm-targeted bioimaging without significant cellular autofluorescence. In addition to that, a cytotoxicity test on NIH3T3 fibroblast cells revealed that the HCPE polymer is noncytotoxic up to 24 h, suggesting HCPEs displayed good opportunities for bioimaging applications. In subsequent work this group then synthesized such HCPEs using CuAAC wand then functionalized them with anti-HER2 antibodies via a carbodiimide-activated coupling reaction. This transformed the HCPEs into a fluorescent probe for targeted cellular imaging of HER2-overexpressed cancer cells such as SKBR-3.^369^ Zhang and co-workers developed aggregation-induced emission (AIE) active fluorescent polymeric nanoparticles (FPNs) through a catalyst-free thiol–yne click reaction for bioimaging applications. Briefly, they synthesized a thiol-modified PEG methacrylate and an AIE-active dye with two alkynyl end groups, and coupled these via a thiol–yne click reaction to obtain the AIE-active amphiphilic copolymer (PEGMA-PhE). This readily assembled into luminescent nanoparticles (PEGMA-PhE FPNs with red fluorescence) with high water dispersity, uniform size (diameter: 100 ± 20 nm) and nanogranular morphology. These PEGMA-PhE FPNs showed remarkable AIE features, high biocompatibility, low toxicity and easy internalization by cells through nonspecific routes.^370^ Bai et al. developed amphiphilic block copolymers of poly(TMC-*co*-DTM)-SS-POEGMA using a combination of ATRP of oligo(ethylene glycol) monomethyl ether methacrylate (OEGMA) followed by ROP, to get an N_3_-functionalized polymer. [In addition, the same polymer without the azide moiety was also prepared.] This was then followed by CuAAC-based postfunctionalization with alkyne-dithiomaleimide (DTM).^371^ The poly(TMC-*co*-DTM)-SS-POEGMA polymer formed strongly green fluorescent micellar structures in methanol solution. Cellular uptake study followed by fluorescence imaging confirmed that the polymer as fluorescent micelles can be used for bioimaging. In addition, the authors nicely “sneaked in” a disulfide functionality in their original building block, which thus provides the option for reduction-induced micelluar breakdown, and as such drug delivery application (Figure 52).

**Figure 53:**
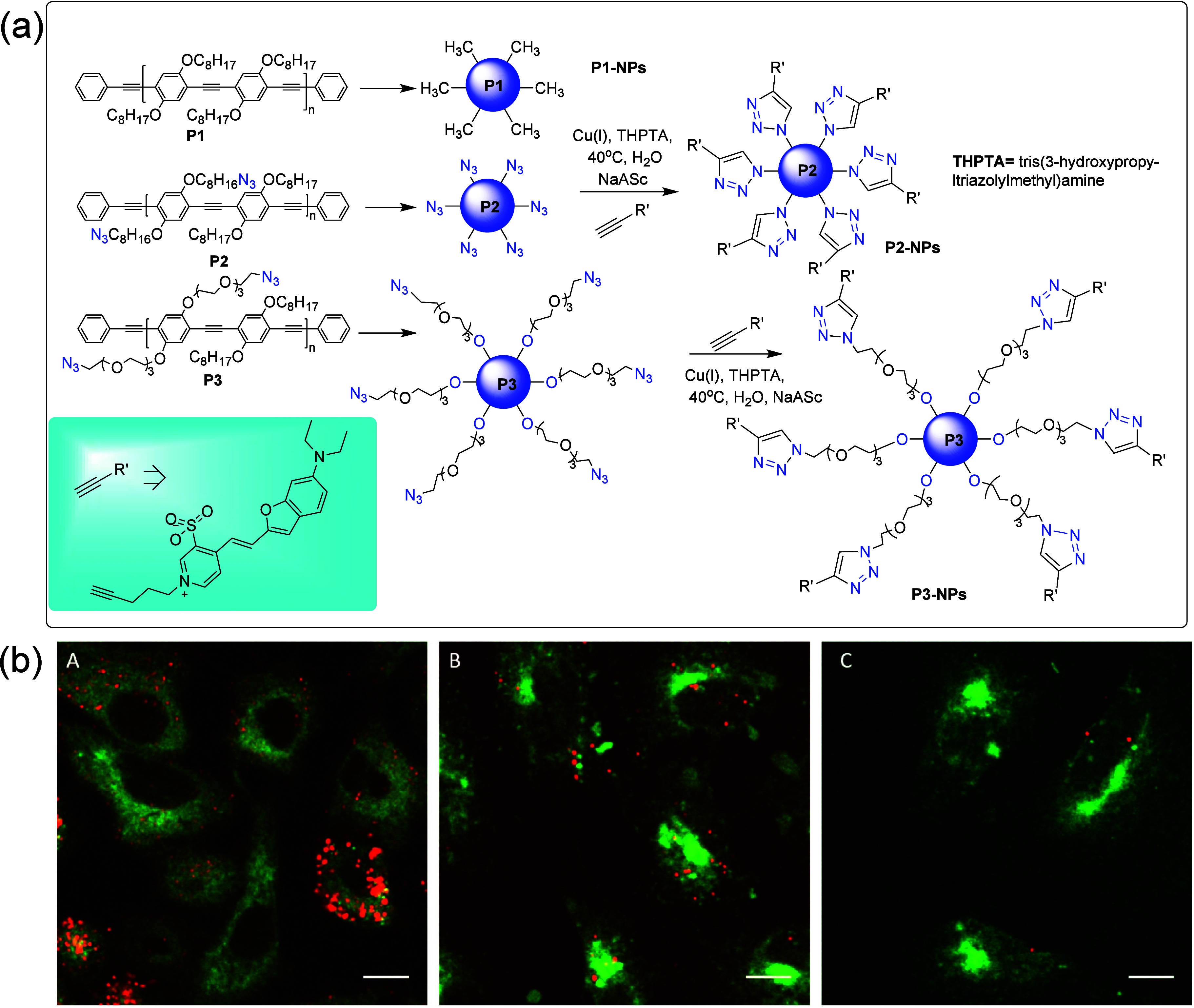
(a) Synthesis from PPE copolymers to NPs of P1, P2, and P3, and functionalization of the latter two using the CuAAC reaction. (b) Confocal microscopy images of A549 cells treated with P1- (A), P2- (B), and P3-NPs (C) for 24 h (scale bar = 10 mm). The NPs (red) are seen distributed within the volume of the cell. The cell membrane is seen labeled in green. The images are single optical sections with the plane of imaging passing through the middle of the cells. Adapted with permission from ref (285). Copyright 2017, Elsevier.^285^

Conjugated polymers are well-known for their bioimaging capability due to their intrinsic optical properties, resulting from extensively delocalized π-electrons. However, the application of conjugated polymers as a bioprobe has long been hampered by their hydrophobic nature and poor biocompatibility. To overcome these problems, Ethirajan and co-workers used CuAAC to develop side chain-functionalized poly(p-phenylene ethynylene) with improved cytocompatibility for bioimaging application.^285^ To this aim, they conjugated azide-terminated tetraethylene glycol to enhance the hydrophilicity of the polymer NPs (Figure 53 a). It was shown that the introduction of azides significantly increased the quantum yield of the NPs (from 8 to 13%). Moreover, these click functionalities enabled (bio)molecule binding onto the NP surface, which can be beneficial for specific cell targeting. As an example, CLSM images revealed that—in contrast with the original hydrophobic NPs—these conjugated polymers easily internalized at a very low concentration of 75 mg/mL within A549 cells and allowed easy bioimaging (Figure 53 b).

**Figure 54:**
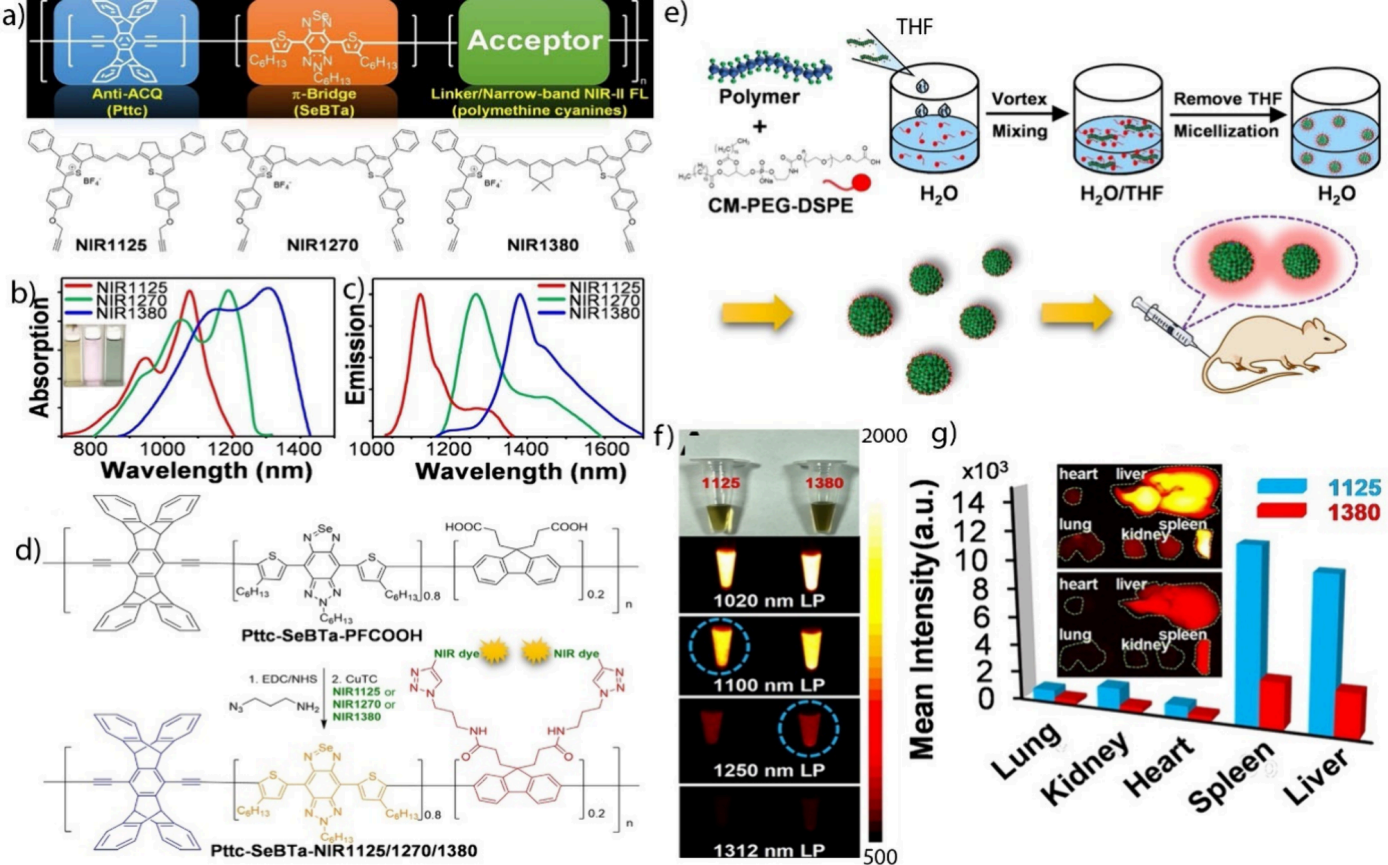
a) Chemical structures of polymethine dye-conjugated semiconducting polymers. Three types of NIR-II fluorescent polymethine dyes (NIR1125, NIR1270, and NIR1380) were synthesized with C≡C—H groups for further conjugation with the semiconducting polymer. b,c) Absorption and emission spectra of NIR1125 (red line), NIR1270 (green line), and NIR1380 (blue line) in CH_2_Cl_2_. d) Synthesis of semiconducting polymer bearing carboxylic acid groups for further transformation to azide groups, followed by CuAAC click reactions with polymethine dyes. e) Schematic showing the preparation of lipid-protected Pdots for *in vivo* bioimaging. f) Photographs of Pttc-SeBTa-NIR1125 (left) and Pttc-SeBTa-NIR1380 (right) Pdots under ambient light (first panel), and under 808 nm laser excitation with a 1020-, 1100-, 1250-, and 1312 nm long-pass filter (second to fifth panel). g) Accumulation of Pttc-SeBTa-NIR1125 (upper panelin the inset) and Pttc-SeBTa-NIR1380 (bottom panel in the inset) Pdots in major excised organs at 24 h postinjection with their corresponding quantitative mean fluorescence intensities. Adapted with permission from ref (372). Copyright 2021, Wiley.^372^

In another work, Chan and co-workers synthesized a series of near-infrared (NIR) active conjugated polymers in a form of polymer dots (Pdots) named Pttc-SeBTa-NIR1125, Pttc-SeBTa-NIR1270, and Pttc-SeBTa-NIR1380 with narrow emission bands (at 1125, 1270, and 1380 nm, respectively) for *in vivo* bioimaging applications.^372^ To make the conjugated polymers NIR-II fluorescence-active, they functionalized NIR-II-fluorescent polymethine dyes (NIR1125, NIR1270, and NIR1380) with the conjugated polymer using CuAAC. All three functionalized polymers showed emission maxima beyond 1000 nm, which is beneficial for *in vivo* vascular bioimaging as this minimizes any contribution from autofluorescence. As evidenced from the fluorescence images in Figure 54, these NIR-II active polymers showed excellent noninvasive, through-skull brain imaging capabilities in live mice with remarkable spatial resolution as well as signal-to-background contrast.

**Figure 55:**
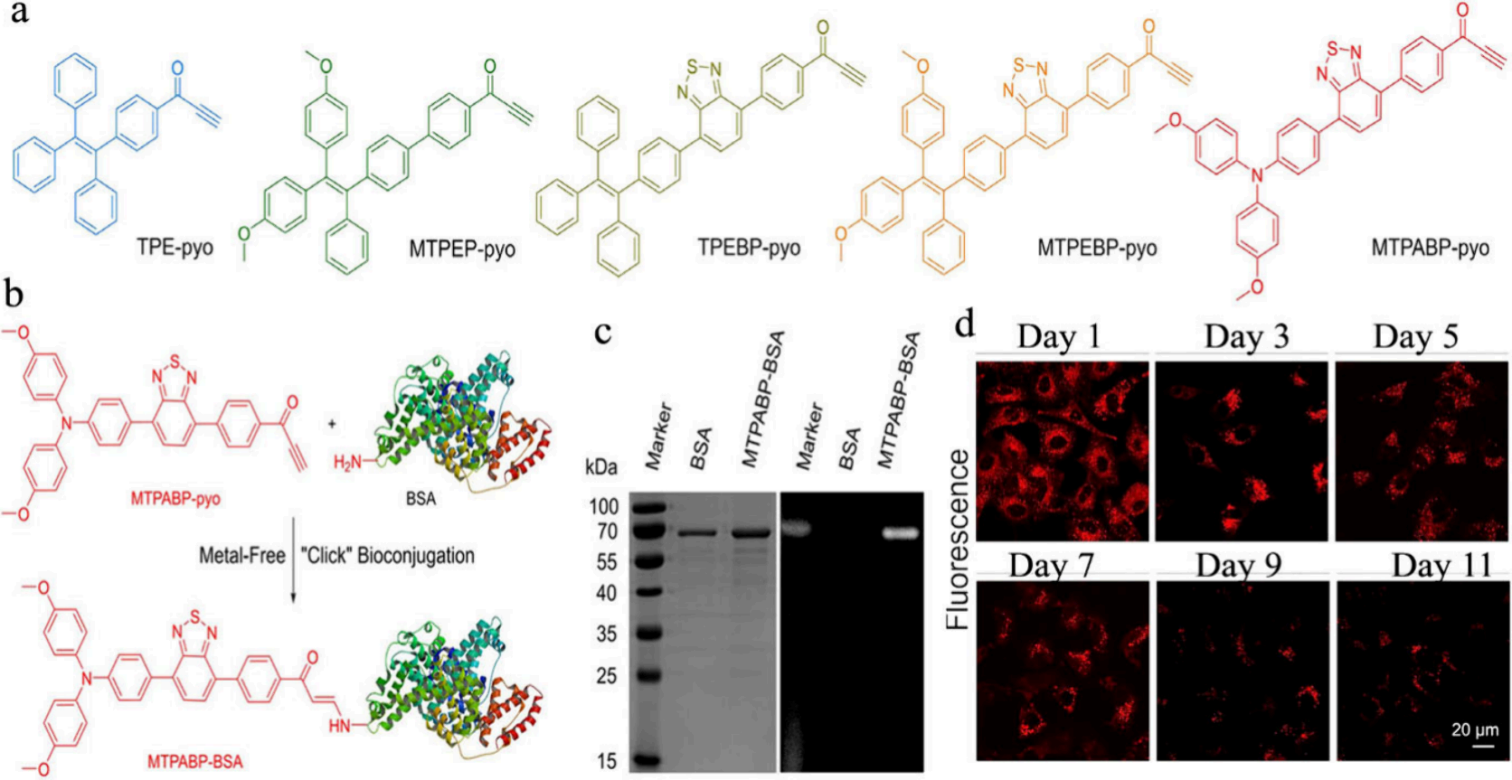
a) Molecular structures of five different AIE-pyo luminogens, b) Metal-free “click” reaction of MTPABP-pyo with native BSA without prefunctionalization at room temperature. c) Verification of bioconjugation between MTPABP-pyo and BSA by SDS-PAGE separation: (left) bright field and (right) fluorescent image; d) Long-term fluorescence imaging of A549 cells by MTPABP-functionalized hydrolyzed silk; (λ_ex_ = 488 nm and (λ_em_ = 565–700 nm). Adapted with permission from ref (376). Copyright 2023, Wiley.^376^

The variety of synthetic avenues and the potential of the resulting polymer can further be seen from three recent studies. Tan et al. synthesized a dye-based water-soluble fluorescent polymer based on perylene bisimides (PBIs), with a water-solubilizing polyelectrolyte modification via a one-pot reaction (the reduction of a trithioester and subsequent thiol–ene click reaction) and investigated its *in vitro* bioimaging capability.^373^ Their synthesized polymer displayed excellent water solubility along with strong red fluorescence in aqueous solution. *In vitro* cytotoxicity test and fluorescence imaging on HeLa cells revealed that the polymer combined a low cytotoxicity with excellent bioimaging capabilities. Wei and his group members developed NPs for biological imaging from fluorescent organic polymers using aggregation-induced emission (AIE) via CuAAC.^374^ For the polymer synthesis, they conjugated azide-modified (PEG-methacrylate)-*co*-poly(allyl glycidyl ether) (PEGMA-AGE-N_3_) and alkyne-terminated AIE-dye 10-cetyl-10H-phenothiazine-3,7-(4,4′-aminophenyl)acetonitrile (PhE-OE). The final PEGMA-AGE-PhE copolymers containing the AIE-active dye can self-assemble into NPs with intense fluorescence due to their AIE feature. The NPs combined a high fuorescence with good cytocompatibility and ready cellular uptake when interacting with L929 cells. Another recent study by He et al. reported using the amino-yne click reaction toward poly(aminomaleimide)s (PAMIs) with intrinsic luminescence for cellular bioimaging.^375^ To synthesize these PAMIs they reacted dimethyl acetylenedicarboxylate with various diamines using the amino-yne click reaction at room temperature. Interestingly, the resulting PAMIs emit intensely in both dilute solution and aggregated state. In addition to that, cytotoxicity tests on HeLa cells revealed that the PAMIs exhibit a low cytotoxicity. As a third example, in 2023 Tang and co-workers similarly reported silk functionalized with aggregation-induced emission luminogens (AIEgens) for long-term bioimaging of cells.^376^ They designed five different AIEgens (see Figure 55a) containing activated alkynes, which were easily reacted with silk fibers by metal-free amino-yne click bioconjugation (Figure 55b,c). The resulting polymer conjugates exhibited bright full-color emissions and high stability. More interestingly, the MTPABP-pyo functionalized silk has been successfully employed for real-time and long-term two-photon fluorescence imaging (Figure 55d) of A549 human lung cancer cells without inducing cytotoxicity. Even after 11 days, the fluorescence activity of the AIEgen-functionalized cells remained high, which suggests its potential application for long-term, deep tissue imaging.

The bioimaging provided by fluorescence and NIR imaging offers high-quality bioimaging with great spatial resolution for cells and small animal models. While typically being an essential first step, their clinical relevance is somewhat limited as they are not effective for deep tissue imaging particularly in large animal models and humans. In this regard, they are complemented by the imaging of deep tissue anatomy as well as physiological processes provided by, e.g., PET and MRI. PET is widely used for clinical imaging due to its high sensitivity and requirement of only minute amounts of tracer molecules. However, the short half-life of the tracer molecules (radionucleotides, such as ^11^C, ^64^Cu, and ^68^Ga) limit its applications in clinical settings. The use of bioorthogonal click chemistries for PET and related applications has been reviewed recently,^377^ with a focus on largely on inorganic (iron oxide, etc.) nanoparticles. Polymers, also mentioned in this review, can play a crucial role in nuclear imaging too, due to their enhanced specificity, extended circulation time, signal amplification, and ease of modification. Click chemistry can further enhance these properties as it offers fast, stable, and high-yield radiosynthesis, enables the use of prosthetic groups for radiolabeling reactions, facilitates the formation of coordination scaffolds for radiometals, and supports the site-specific radiolabeling of proteins and peptides.^378^ In addition to that, it can also serve as a foundation for innovative strategies in *in vivo* pretargeting, where radiotracers are selectively delivered to specific tissues before imaging.

For example, taking advantage of click chemistry Wängler and co-workers stuided three different types of click reactions to determine the most efficient multimerization approach toward obtaining multimeric cyclic RGD (cRGD) peptides.^379^ They noticed that oxime formation and 1,3-dipolar cycloadditions were not much effective to synthesize high multimeric probes (octamers and hexadecamers, specifically) in a stepwise fashion, whereas the Michael addition was much more effective toward this goal. Using this approach, they next coupled such multimers via a PEG spacer to a DOTA (dodecane tetraacetic acid) derivative for accessible ^68^Ga radiolabeling. These cRGD multimers could easily be labeled with ^68^Ga in radiochemical yields between 95% and 98% and radiochemical purities between 96 and 99%. The high binding affinity of cRGD moieties multimers was further amplified with increasing number of peptide moieties in the *in vitro* studies, gaining for the hexadecamers more than to orders of magnitude increase in the affinity toward α_ν_β_3_ integrin-expressing U87MG cells, compared to the corresponding monomer, respectively.

**Figure 56:**
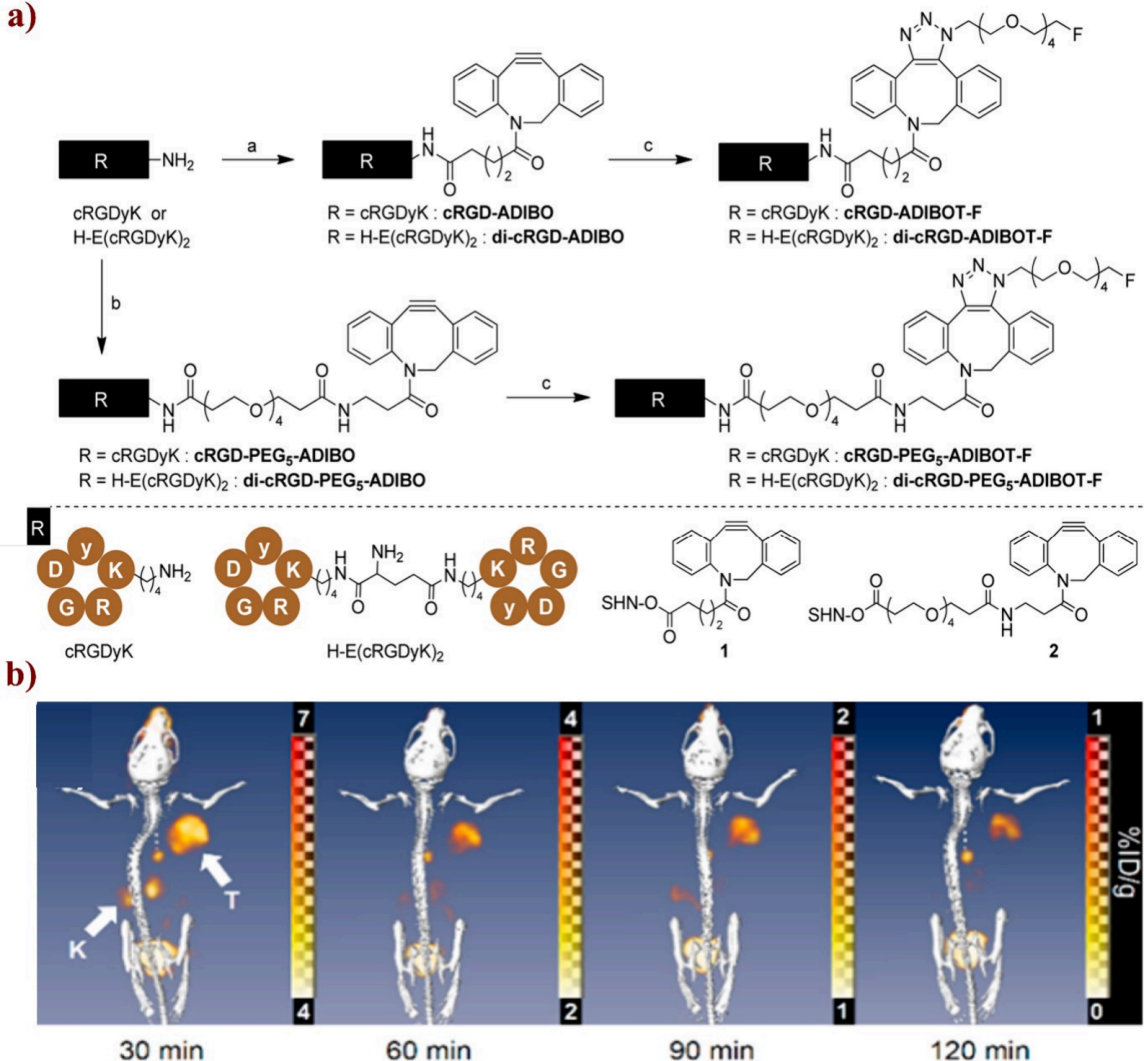
a) Preparation of nonradioactive cRGD peptide derivatives; cRGD-ADIBOT-F, di-cRGD-ADIBOT-F, cRGD-PEG5-ADIBOT-F, and di-cRGD-PEG5-ADIBOT-F; b) *In vivo* evaluation of di-cRGD-PEG5-ADIBOT-^18^F using PET-CT images of U87MG tumor-bearing mice at 30, 60, 90, and 120 min postinjection of dicRGD-PEG5-ADIBOT-^18^F (1.8 MBq). Note: T = tumor, K = kidney. Adapted with permission from ref (382). Copyright 2014 ACS.^382^

In 2012 Zeng et al. reported the synthesis and use of ^64^Cu core-labeled polymeric nanoparticles with high specific activity.^380^ Briefly, they compared the efficacy of both CuAAC and SPAAC reactions to prepare polymeric nanoparticles, and found the latter one to be more effective. To prepare the nanoparticles they employed sequential nitroxide-mediated radical polymerization and synthesized a Boc-protected precursor diblock copolymer named, poly(*tert*-butyl acrylate)-*b*-poly(chloromethylstyrene-*co*-styrene) (PtBA-*b*-P(CMS-*co*-S)). Subsequently, a Boc deprotection reaction was followed by a grafting amidation reaction of a 2 kDa methoxy-terminated PEO-amine, which resulted in a deprotected diblock copolymer, poly(acrylic acid)-*g*-mPEO-*b*-poly(chloromethylstyrene-*co*-styrene) (PAAg-mPEO-*b*-P(CMS-*co*-S)). This material self-assembled to form core–shell micelles in water. Next, azide functionalities were introduced into the core of the micelle by reacting the benzyl chloride moieties of the chloromethylstyrenes with sodium azide. Finally, shell functionalization wth alkyne moieties and cross-linking of the core yielded core/shell click-ready nanoparticles. Compared with CuAAC chemistry, the SPAAC reaction with a ^64^Cu-DOTA-DBCO complex provides three advantages for the ^64^Cu labeling of the core of these nanoparticles: (1) it eliminated copper exchange between the nonradioactive Cu of the catalyst and the DOTA-chelated ^64^Cu; (2) it elimination internal “click” reactions between the azide and acetylene groups within the same NP; and (3) it increased the efficiency of the click reaction, as the water-soluble Cu(I) now does not need to reach the hydrophobic core of the NPs. This SPAAC strategy increased the specific activity of ^64^Cu-labeled nanoparticles to 975 Ci/μmol at the end of the synthesis, compared with the 8.25 Ci/μmol specific activity previously reported for ^64^Cu-labeled cross-linked iron oxide nanoparticles. Although the authors did not report any *in vivo* bioimaging, they suggested that the nanoparticles could serve as valuable PET agent for bioimaging applications, given the specific activity of the radiolabeled product at 975 Ci/μmol (4420 Ci/μmol at the end of bombardment). In 2015, Lewis and co-workers reported an ^18^F-pretargeted PET imaging approach based on the IEDDA reaction between tetrazine (Tz) and *trans*-cyclooctene (TCO).^381^ Briefly, as a proof of concept, they use an Al[^18^F]-triazacyclononane-1,4,7-triacetic acid (NOTA)-labeled tetrazine radioligand to interact with a selected antibody. This 5B1 antibody is a fully human IgG that targets a promising biomarker for pancreatic ductal adenocarcinoma: carbohydrate antigen 19.9 (CA19.9). In order to arm the antibody with the reactive bioorthogonal moiety, they incubated purified 5B1 with an activated succinimidyl ester of TCO (TCO-NHS, 35 equiv) at room temperature for 1 h. Then the IEDDA reaction takes place (>94% based on consumption of the reagents, 15 min, room temperature), and although the authors do not highlight this, the IEDDA reaction is probably the best choice for this given its superior reaction rate over other strain-promoted click reactions, which is highly relevant here given the short lifetime of the ^18^F isotope. The ^18^F-based pretargeted PET imaging system displayed a promising biodistribution and produced tumoral activity concentrations of up to 6.4%ID/g at 4 h postinjection in BxPC3 xenograft-bearing mice. Additionally, small-animal PET imaging experiments revealed that this methodology clearly visualized CA19.9-expressing tissues with high tumor-to-background activity ratios. In a similar type of study, Kim and co-workers reported ^18^F-labeled cRGD probes based on SPAAC chemistry for PET imaging.^382^ Via this route they prepared a series of fluorine-substituted monomeric and dimeric cRGD peptide derivatives, such as cRGD-ADIBOT-F (ADIBOT = azadibenzocyclooctatriazole), di-cRGD-ADIBOT-F, cRGD-PEG5-ADIBOT-F, and di-cRGD-PEG5-ADIBOT-F. Among these cRGD derivatives, di-cRGD-PEG5-ADIBOT-F exhibited the highest binding affinity in a competitive binding assay compared to other derivatives and even the original cRGDyk. *In vivo*^18^F-PET imaging studies yielded biodistribution data on U87MG tumor-bearing nude mice models that revealed that this radiotracer allowed successful visualization of tumors with good tumor-to-background contrast ratio and a high uptake by the tumor compared to other major organs (such as the kidney; see Figure 56).

In 2018, Li and his group members reported a multifunctional polymeric carrier for simultaneous PET imaging as well as combination therapy.^383^ Their triblock copolymer POEG-*b*-PVBA-*b*-PFTS (POVF) composed of a poly(oligo(ethylene glycol) methacrylate) (POEG) hydrophilic block, a poly(4-vinylbenzyl azide) (PVBA) middle block, and a polyfarnesylthiosalicylate (PFTS) hydrophobic block. The azide groups in the middle block allow via a SPAAC reaction the incorporation of a PET-imaging (containing ^64^Cu for measurements up to 8 h or ^89^Zr for measurements up to 96 h) modality that was linked to DBCO. Their nanocarrier system was capable of codelivering paclitaxel (PTX) and farnesylthiosalicylate, and imaging by PET of the drug delivery process. The radiolabeled nanocarrier exhibited excellent serum stability, rapid tumor uptake, and slow clearance in a 4T1.2 tumor-bearing mice model. They observed that PTX/POVF micelles started to accumulate in tumors at as early as 1 h post injection, and facilitated increased tumor accumulation over time from 1 to 24 h post injection, although also near-equal doses were observed in other organs at 24 h post injection: in the tumor 13.6 ± 2.3% ID/g, in the liver 12.4 ± 3.4% ID/g, and in the kidney 16.7 ± 2.7% ID/g. These data suggests, once improved tumor specificity can be obtained, such polymeric carriers can be used both for PET imaging as well as for combination therapy. A SPAAC reaction was also central in a report by Gupta et al. on polyethylenimine (PEI)-decorated calcium alginate microspheres (PEICAMSs) that were radiolabeled with ^68^Ga for PET imaging.^384^ These PEICAMSs, which are biodegradable, were reacted with azadibenzocyclooctyne-*N*-hydroxysuccinimide ester (ADIBO-NHS). In parallel, azide-functionalized NOTA chelator (N_3_-NOTA) was labeled with ^68^Ga, and then reacted with the surface-modified PEICAMSs using SPAAC. The radiolabeling efficiency of such ^68^Ga-NOTA-PEI-CAMSs was found to be 99%, and the microspheres exhibited a high stability (92%) in human blood serum. More importantly, PET images demonstrated the stability and biodistribution of the microspheres in C57BL/6 mice for up to 2 h post injection, which, the authors highlight, suggests the usefulness of such biodegradable PET microspheres for preoperative imaging and targeted radionuclide therapy.

MRI is also widely used to generate high-resolution images of the body’s internal structures. Unlike PET, which provides functional imaging by tracking the distribution of radiotracers, MRI offers detailed anatomical images using strong magnetic fields and radio waves. MRI is particularly effective in visualizing soft tissues, such as the brain, muscles, and internal organs, and is often used for diagnostic purposes, treatment planning, and monitoring disease progression, via location-specific changes in the spin–lattice relaxation time *T*_1_, or the spin–spin relaxation time *T*_2_ of water in the body. The key advantages of MRI include its noninvasiveness, low radiation dose, and excellent soft-tissue resolution and discrimination across all imaging planes, and as such is widely used in hospitals. With the introduction of MRI bioimaging probes in 1988, MRI has also been used for angiography and perfusion imaging.^385^ The most commonly used MRI imaging probes are low-molecular weight chelates of metal ions like Mn^2+^ or Gd^3+^, along with iron oxide particles. However, their transient tissue retention time, toxicity, and unfavorable pharmacokinetic profiles result in a narrow imaging window and low signal-to-noise ratio, which limits their clinical applications. To address these challenges, various polymer-based imaging modalities have been proposed to enhance their therapeutic potential. Polymer-based MRI bioimaging probes have several advantages over the small molecule-based and metallic MRI probes, and these include reduced toxicity, extended retention time in blood, significantly increased contrast, and the possibility to easily attach various functionalities to the polymer particles. In this regard, click chemistry offers a versatile and efficient approach for attaching such functionalities.

Taking advantage of click chemistry, Liu and co-workers reported a Gd(III)-chelated hyperbranched conjugated polyelectrolyte (HCPE-Gd) and its application in fluorescence and magnetic resonance (MR) dual imaging in live animals.^386^ For the synthesis, first a neutral hyperbranched polymer (HCP) was prepared from 4-(9,9′-bis(6-bromohexyl)-7-ethynylfluorenyl)-7-ethynylbenzothiadiazole by alkyne polycyclotrimerization using CpCo(CO)_2_ as a catalyst. Subsequent reaction between HCP and trimethylamine (TMA) in tetrahydrofuran (THF)/methanol yielded water-soluble HCPE-N^+^, which was further clicked via a CuAAC reaction with an azide-terminated-PEG amine, which resulted in HCPE-NH_2_. Next, this HCPE-NH_2_ was reacted with diethylenetriaminepentaacetic (DTPA) dianhydride that resulted in HCPE-DTPA, which can chelate with Gd(III) to form HCPE-Gd(III). The synthesized HCPE-Gd forms nanospheres with an average diameter of ∼42 nm as confirmed by DLS with a fluorescence quantum yield of 10% in an aqueous solution. More importantly when they compared the MRI capabilities of HCPE-Gd(III) with that of commercially available MRI agent Magnevist, they noticed that HCPE-Gd provided superior sensitivity: the relaxivity (*R*_1_), which refers to the amount in 1/*T*_1_ per unit concentration of agent, was found to be 16.8 mM^–1^ s^–1^ for HCPE-Gd, which is ∼3 times higher than that of Magnevist (5.7 mM^–1^ s^–1^). Furthermore, fluorescence and MR imaging studies in H22 hepatoma tumor-bearing mice revealed that HCPE-Gd functions as an effective dual-modal optical/MR imaging nanoprobe, extending the toolbox for *in vivo* cancer studies.

**Figure 57:**
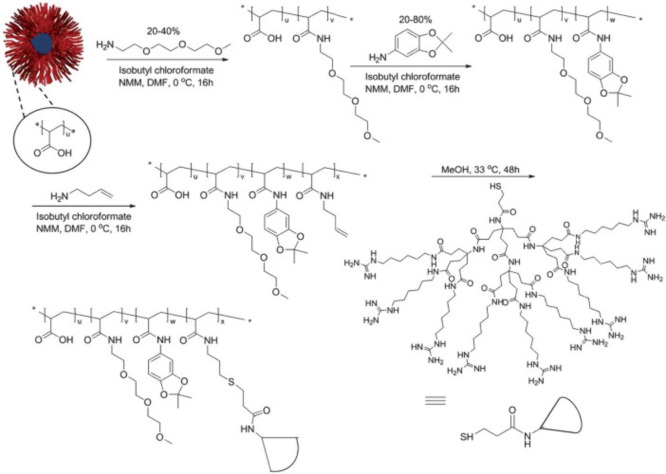
Synthesis of star polymer-based MRI contrast reagent using thiol–ene click chemistry. Reproduced with permission from ref (387). Copyright 2012 RSC.^387^

Around the same time, Adkins et al. reported the synthesis of MRI-active bimodal acrylate star polymers (10 ± 2 nm) using thiol–ene click chemistry.^387^ These polymers were built around fluorene-based core units and were further modified with dopamine derivatives to chelate lanthanides such as Gd^3+^ and Eu^3+^ for MR imaging. The detailed synthesis of the MRI-active star polymers is shown in Figure 57. The multifunctional polymeric architecture allows for a high loading via the small catechol complexing units; this resulted in high relaxivity *R*_1_ values of 85 mM^–1^ s^–1^ in an applied magnetic field of 0.5 T at 37 °C confirming the rapid water exchange of the highly hydrated star polymer. Incorporation of the Eu^3+^ then allowed for a multimodal study of these materials (fluorescence at two wavelengths and MRI).

In another recent study, Xiao et al. developed a polymeric dual-modal imaging nanoprobe with two-photon aggregation-induced emission for fluorescence imaging as well as gadolinium-chelation for MRI.^388^ Briefly, they synthesized an amphiphilic block copolymer consisting of (ethylene glycol) methyl ether methacrylate (OEGMA) and *N*-(2-hydroxypropyl) methacrylamide (HPMA) derivatives, as a carrier for conjugating an MRI contrast agent, Gd-DOTA, and a two-photon fluorophore 2,6-bis(4-(diphenylamino)phenyl)-1,4-dihydropyrazine) (TPBP) with aggregation-induced emission (AIE) properties. Incorporation of Gd in the hydrophilic chain segment of the OEGMA-based carrier, yielding Gd-DOTA-TPBP, resulted in a high *R*_1_ value of 7.19 mM^–1^ s^–1^, which was more than twice the *R*_1_ value of reference compound DTPA-Gd. In addition, due to the larger particle size and higher molecular weight, Gd-DOTA-TPBP exhibited a prolonged blood circulation time and superior performance in vascular imaging of BALB/c mice compared to Gd-DTPA. Finally, the enhanced accumulation of Gd-DOTA-TPBP in tumors via the EPR effect, along with its higher R_1_ relaxivity, provided superior and prolonged contrast enhancement in MRI scans, again highlighting the potential of block polymer-based contrast agent as multimodal imaging contrast agents.

From the above discussion, it becomes clear that substantial amounts of work are being undertaken in the field of click-based polymers for bioimaging. Both the frequently mentioned intense intrinsic emission and/or easy functionalization with dyes, PET-isotopes or MRI agents, and low *in vitro* toxicity suggest significant potential for such polymers. Here specifically three features deserve further attention: 1) The autofluorescence of biological materials demands extensive red-shifts in bioimaging dyes. The synthetic flexibility of click-based polymers suggests that the embedding of such dyes will likely be feasible without intrinsic problems. 2) The already low cytotoxicity—often hidden by the intrinsically low concentrations needed for bioimaging—should be even further reduced if *in vivo* use comes into play, especially when used for human clinical purposes. 3) For PET and MR imaging, further long-term *in vivo* toxicity studies are required to assess safety profiles and gain a clearer understanding of their clearance mechanisms from the body. Again, the tremendous ease of variation and the ever-increasing toolbox of click reactions will likely allow reaching this goal.

### 3.5 Hydrogels for Tissue Engineering

Tissue engineering is an emerging field of research where principles of chemistry, biology, and engineering work together to develop new functional tissues for treating diseased or damaged tissue.^389^ The concept of tissue engineering was proposed by Langer and Vacanti in the early 90s. After that, significant progress was made in this field, e.g., by developing novel scaffolding materials, new strategies to deliver growth factors, and new techniques of cell culture growth on the scaffold. For successful tissue engineering, scaffolds play a vital role as they act as an extracellular matrix (ECM), onto and into which cells are attached, proliferate, differentiate, and ultimately form the tissue. In this context, polymeric biomaterials are the primary choice for tissue-engineering applications due to several advantageous properties over metallic and ceramic-based scaffolds. These include ease of synthesis, great flexibility to regulate the physical, chemical, and biological properties, tunable biodegradation behavior and good control over processability. In this regard, click chemistry has offered a wide range of design and cross-linking strategies to develop multifunctional hydrogel polymeric materials. Functional hydrogels are one of the potential candidates for tissue engineering application due to their tunable material properties and their similarity to natural ECMs, as both ECMs and hydrogels retain a significant amount of water within their cross-linked polymeric network. Until now, both naturally as well as synthetic polymers are used to synthesize hydrogels, and both types up to now have characteristic advantages and disadvantages. Natural polymers offer an adhesive surface for cell attachment and proliferation, and both they and their degradation products are generally nontoxic; however, their application is somewhat restricted, as many of such natural polymers display both relatively poor mechanical properties and fast degradation. On the other hand, synthetic polymers, provide control over their physical and chemical properties and degradation behavior, e.g., by modifying their Flory interaction parameter, by changing the monomeric unit(s), or by changing monomer feed ratios. The latter suggest significant potential for synthetic polymers in this regard, but the main problem with synthetic polymers is that their degradation (by)products can cause secondary acute inflammation.

To control the degradation behavior of hydrogels, it is important to regulate the cross-linking density within the polymer structure. Generally, two types of cross-linking strategies are employed: a) (semipermanent) chemical cross-linking via covalent bonds, or b) (more temporary) physical cross-linking, via physical entanglements, hydrogen bonding, or metal complexation. In this context, click-based reactions provide a unique platform to customize the cross-linking density of hydrogels via either of these two routes, which is very important in terms of different types of tissue engineering applications. Frequently (also) photochemical cross-linking reactions are used–sometimes in combination with thermal reactions–to allow photopatterning, as this allows detailed investigations on the effects of the (degree of) cross-linking in such polymeric materials on what is to become the microenvironment of novel tissues.

In this section of the review, we highlight the use of different click reactions for the synthesis as well as the cross-linking of different hydrogel systems and their application in the field of tissue engineering.

#### 3.5.1 CuAAC Click Chemistry-Based Hydrogels

CuAAC is probably the most used click reaction for the synthesis of hydrogels for tissue engineering applications. The key advantage of CuAAC-based hydrogels is their quick gelation and adequate yield of production. However, there are also some disadvantages, which include nonhomogeneous viscosity and accumulation of copper catalyst inside the hydrogel. There are several methods to stop the accumulation of excess copper ions inside the hydrogel network, such as *in situ* reductions or photochemical methods to reduce copper ions, but it was observed that all of these methods are not that effective to extract the excess copper ions from the hydrogel. The remaining traces of copper catalysts display, however, significant cytotoxicity and restrict the usability of CuAAC for tissue engineering applications.

**Figure 58:**
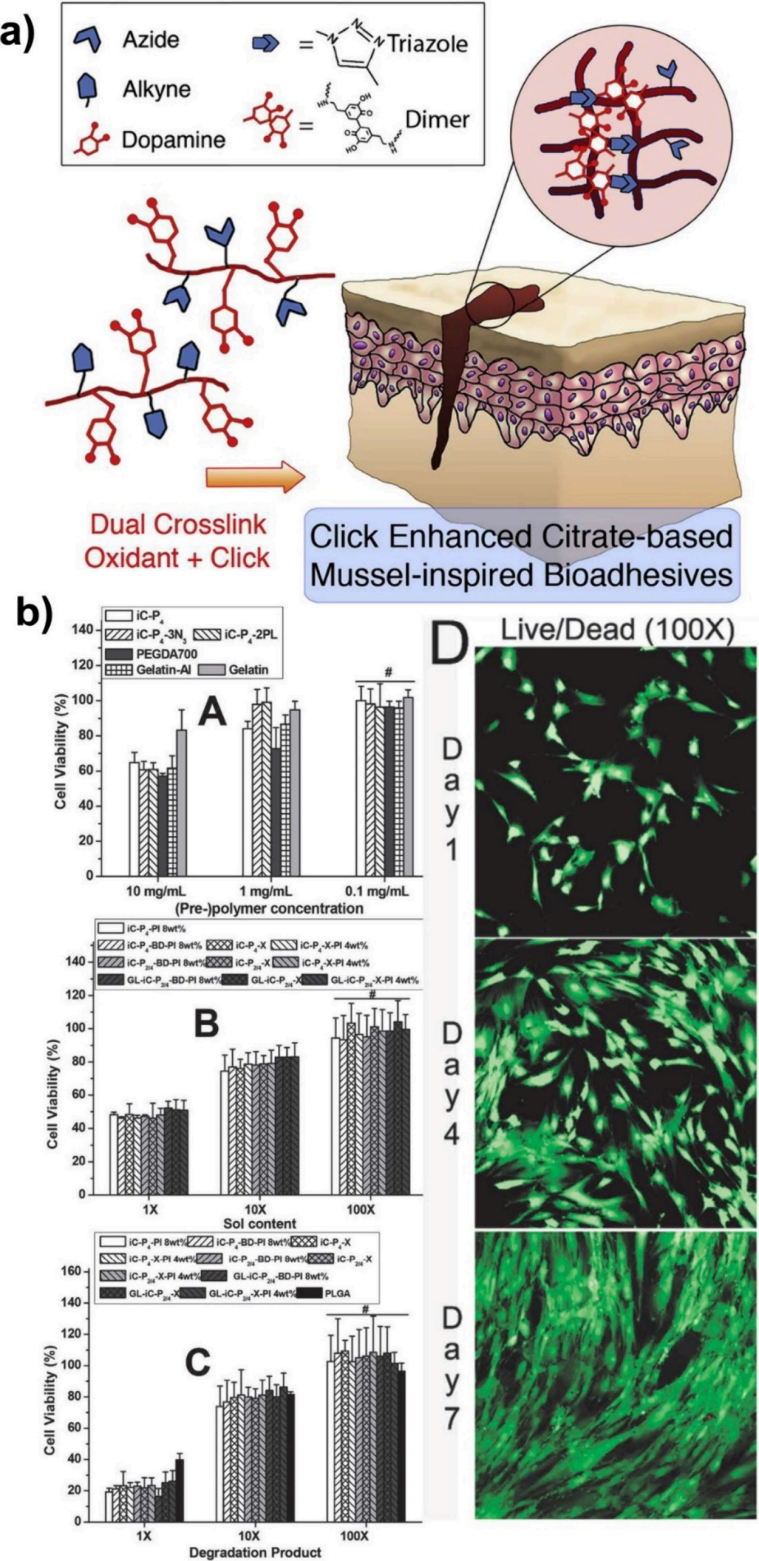
a) Clickable injectable citrate-based mussel-inspired bioadhesive prepolymers (see Figure 31 for structural details). b) Cytotoxicity evaluation (MTT assay) of clickable bioadhesive hydrogel family for: (A) hMSCs cultured with bioadhesive hydrogel-click prepolymers and Gelatin-Al (GL); (B) leachable part (sol content), (C) degradation product of clickable bioadhesive hydrogel cross-linked through different routes for 24 h. (D) Live/Dead assay for hMSCs seeded on dual-cross-linked bioadhesive hydrogel casted glass slides 1, 4, and 7 days post cell seeding. Adapted with permission from ref (179). Copyright 2017, Elsevier.^179^

Therefore, several recent studies focused on the extraction of these copper ions through water-soluble ligands/chelating agents, such as bis(l-histidine), tris(hydroxyl propyl triazolyl methyl) amine, bipyridine, 4-(2-hydroxy ethyl)-1-piperazine ethanesulfonic acid, etc. For example, Yang and co-workers demonstrated the use of 4-(2-hydroxy ethyl)-1-piperazine ethanesulfonic acid as a chelating agent for copper ions in CuAAC reactions to synthesize a mussel-inspired citrate-based antimicrobial bioadhesive hydrogel (see Figure 31 for synthetic details). The schematic representation of the bioadhesive hydrogel is shown in Figure 58a. The hydrogel showed excellent bioadhesion properties, with a strength of 223 kPa, along with good antimicrobial properties, both advantages for skin tissue engineering or any other invasive applications. In addition, upon culturing human mesenchymal stem cells (hMSCs) in the presence of a polymer hydrogel a steady increase in cell number was observed (see Figure 58b), along with a leachable part (sol content) and degradation products, without affecting the cell morphology. This increase in cell number is similar to a control with a PEG diacrylate (PEGDA) hydrogel and with the use of gelatin as such, and taken together with the other data suggests that the synthesized gel is not only mechanically superior over these alternatives, but also cytocompatible, allowing next-phase use for tissue engineering applications.^179^

**Figure 59:**
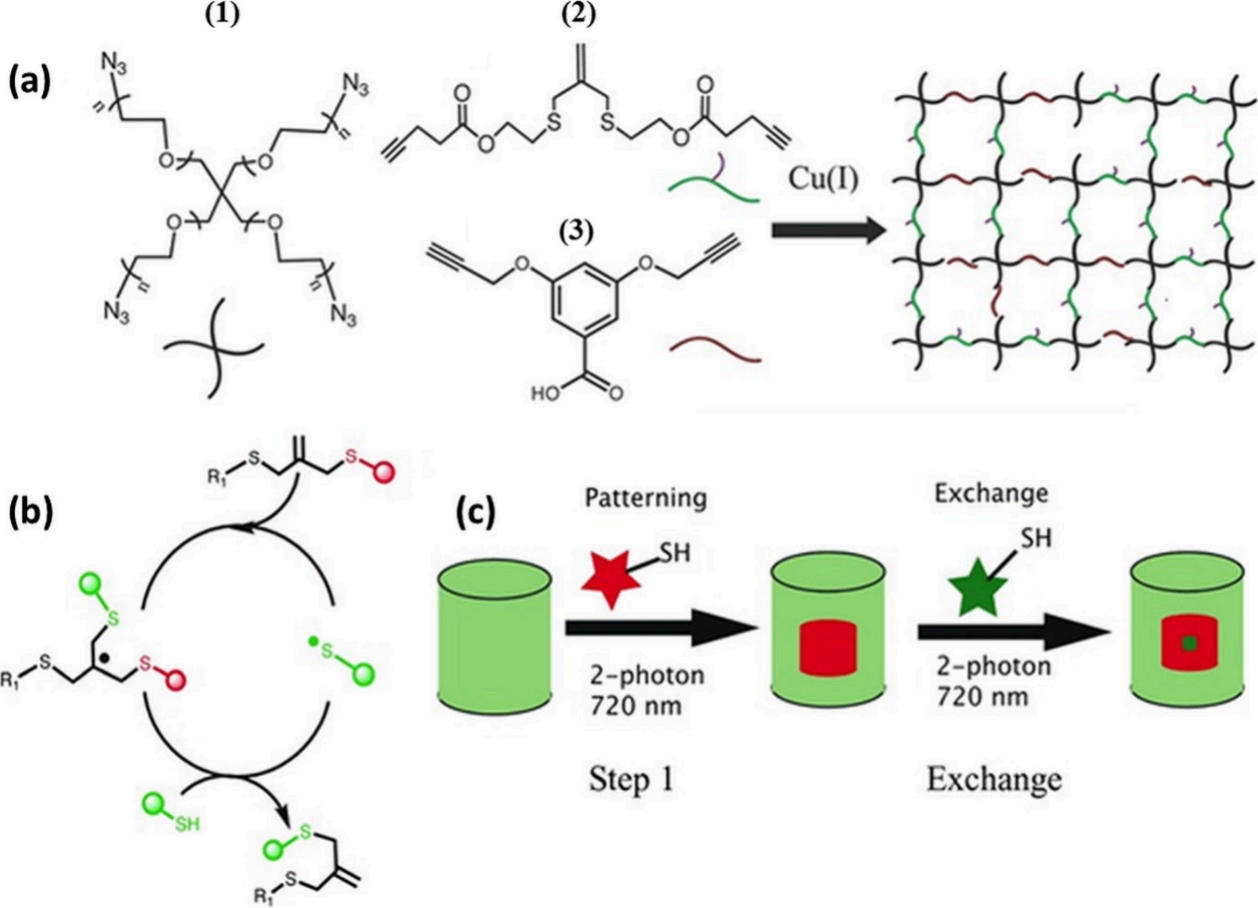
a) Monomers used to form the hydrogel networks, and mechanism of patterning fluorescently tagged CRGDS to the allyl sulfide-functionalized hydrogel. b) Mechanism of replacement of a thiol-containing compound on the allyl sulfide. c) Schematic of replacement of biochemical ligand. Adapted with permission from ref (391). Copyright 2014, Wiley.^391^

In another work, Li et al. reported a PEG-based hydrogel with enhanced mechanical properties prepared using a facile thermally induced copper-catalyzed azide–alkyne cycloaddition (CuAAC) click reaction of α,ω-diazido PEG along with tetrakis(2-propynyloxymethyl)methane, using AIBN as the reducing agent for the Cu(II) → Cu(I) conversion.^390^ The clicked hydrogel showed good mechanical properties with a tensile strength of 2.51 MPa with 500% elongation at break, which is much higher than PEG-based hydrogels prepared from CuSO_4_/NaSac-mediated or CuBr/PMDETA-catalyzed CuAAC reaction. Additionally, the as-synthesized PEG-based clicked hydrogel displays a low *in vitro* cytotoxicity (>85% cell viability after 72 h) toward HL-7702 cells, while *in vivo* implantation of the hydrogel into a porcine model demonstrated good biocompatibility (no signs of tissue swelling or ichors), which makes it a suitable candidate for further tissue engineering studies. Anseth and co-workers also developed a novel RAFT-capable allyl sulfide-functionalized PEG hydrogel. For their synthesis, they used a 4-arm PEG5000 tetra-azide and two different cross-linkers (2-methylene-propane-1,3-bis(thioethyl 4-pentynoate) and 3,5-bis(propargyl) benzoic acid), and reacted these in the presence of a Cu(I) catalyst followed by a thiol–ene reaction using the photoinduced reactivity of a thiyl radical ligand, to achieve photopatterning in the hydrogel networks. Briefly, to synthesize the hydrogel, first a CuAAC reaction (Figure 59a) was performed between 4-arm PEG tetra-azide and difunctional alkyne cross-linkers that proceeds quantitatively to form step-growth networks. In the second stage, to regulate the cell attachment and morphology, they used a cysteine-modified cell-adhesive RGDS peptide as a model biochemical agent for photoconjugation using a thiol–ene exchange reaction (Figure 59b). It was observed that, upon seeding of hMSCs, the cells readily attached to the hydrogel without changes in the hMSC morphology, suggesting the hydrogels are cytocompatible. In addition to that, they also showed that upon exchanging of the fluorescent RGD to a biologically inactive nonfluorescent RGD peptide by exposing the gel to a laser did not adversely affect the local cells. This suggests that this strategy can be effectively employed to temporally and spatially control the local microenvironment of cells, reversibly and multiple times using single and multiphoton wavelength light, which can be very useful for tissue engineering application.^391^

In another recent study, Wong and her team developed a novel hydrogel by simple modification of the collagen backbone lysine groups with a *N*-hydroxysuccinimide-modified PEG featuring a terminal azide group.^392^ To obtain the final hydrogel, the azide groups were further functionalized with specific sortase-recognizing peptides featuring a complementary alkyne group using CuAAC chemistry. The presence of different enzymatic recognition peptides onto the collagen backbone facilitated the utilization of highly specific and orthogonal sortase-mediated cross-linking strategies, and enabled the formation of blood and lymphatic capillaries without any exogenous vascular endothelial growth factor. This hydrogel showed a very good cytocompatibility toward both blood endothelial cells (BECs) and lymphatic endothelial cells (LECs) at 0.5% and 1% of hydrogel concentrations, with a viability more than 90% after 1 day and 3 days, respectively. This was rather dose dependent, as the 2% hydrogel exhibited low viability for both the BECs and LECs (ca. 33% and 68%, respectively) already after 1 day. The engineered hydrogel scaffolds efficiently facilitated rapid vascularization as assessed after 14 days, and readily established a primary network of blood vessels and lymphatic vessels, both *in vitro* as well as *in vivo* in an athymic Nu/Nu immune-compromised rats model. Such enzymatic cross-linking offers several advantages particularly in terms speed and higher material versatility, making it an attractive alternative to conventional physical cross-linking methods, such as base- or heat-mediated fibrillogenesis.^393,394^ Additionally, the rapid cross-linking kinetics, even at low biopolymer concentrations, make the material likely well-suited for support-free, high-resolution 3D extrusion bioprinting of vascularized constructs.

All in all, great strides have been made CuAAC-based click hydrogels, also driven by the ease of synthesis of the reactants, although it is clear that the toxicity associated with copper ions motivated the use of other, metal-free click reactions, as discussed in the next paragraphs.

#### 3.5.2 SPAAC Click Chemistry-Based Hydrogels

**Figure 60:**
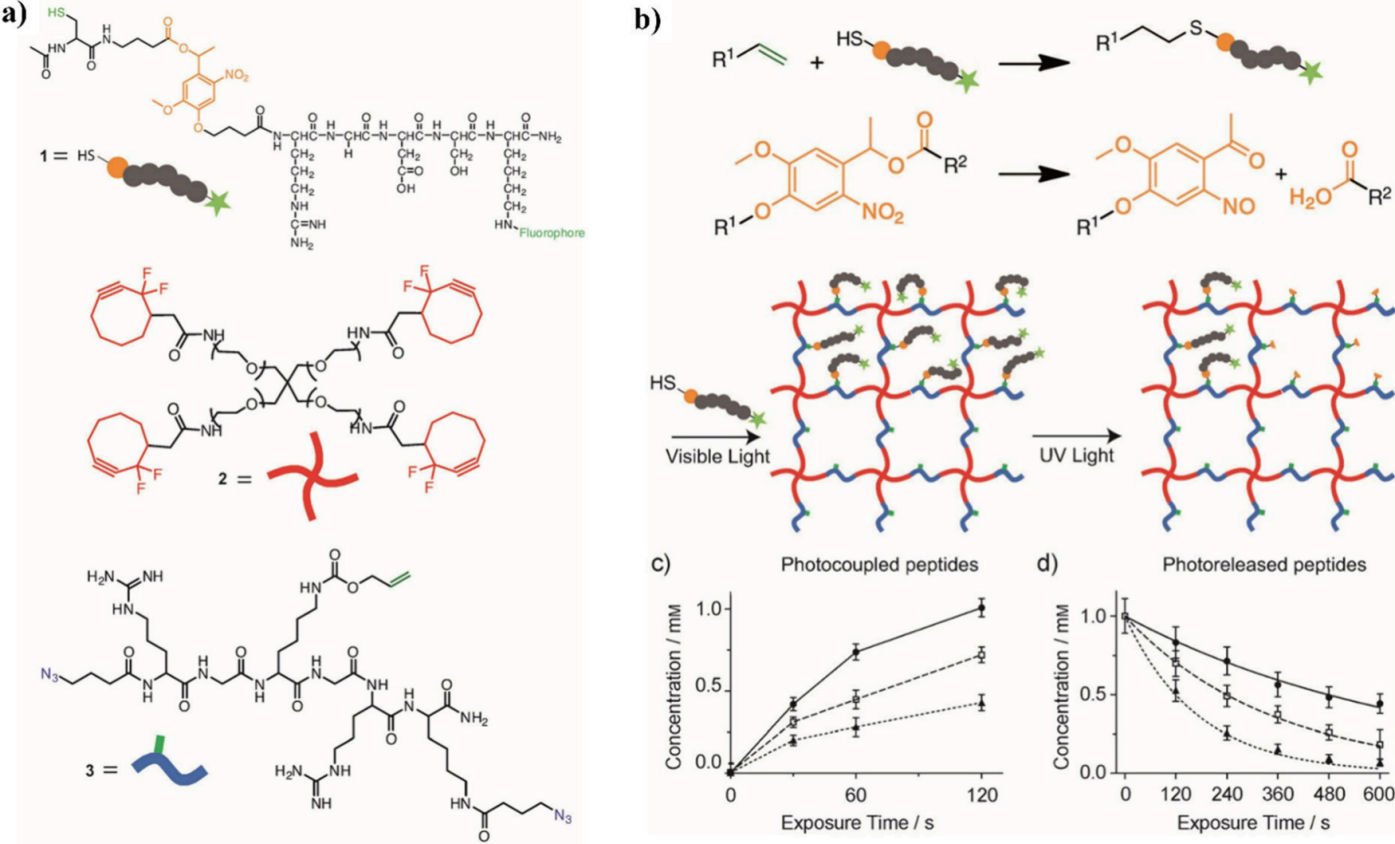
a) Hydrogels formed from 4-arm PEG-cyclooctyne (*M*_n_ ∼ 10 kDa, **2**) and N_3_-RGK(alloc)GRK(N_3_)-NH_2_ (**3**). The alloc groups were functionalized with the fluorescent peptide Ac-C-(PL)-RGDSK(AF488)-NH_2_ (**1**) through thiol–ene photoreaction and defunctionalized through *o*-nitrobenzyl ether photocleavage. b) Representation of the thiol–ene conjugation reaction (top), the *o*-nitrobenzyl ether cleavage reaction (middle), and the overall hydrogel structure and reversible patterning approach (bottom). c) Concentrations of peptide **1** patterned by thiol–ene reaction as a function of the initiator and visible light exposure time. d) Experimentally determined and predicted concentrations of **1** based on the photocleavage kinetics as a function of UV light and exposure time. Adapted with permission from ref (395). Copyright 2012, Wiley.^395^

To avoid this use of copper, DeForest et al. synthesized a robust hydrogel by reacting a four-arm azide-modified PEG, with a bis(difluorinated cyclooctyne) polypeptide—which was also endowed with terminal alkene moieties—for encapsulating cells using SPAAC.^188^ Subsequently, using those alkene groups, they also performed a photochemical thiol–ene click reaction using a fluorescent peptide Ac-C-(PL)-RGDSK(AF488)-NH_2_ that enables visible patterning of biological functionalities within the gel in real-time with micrometer-scale resolution. This strategy enables to control the biophysical and biochemical properties of the cell culture microenvironments *in situ*. In a subsequent study, they also used a copper-free, strain promoted, azide–alkyne cyclooaddition (SPAAC) click reaction to synthesize hydrogels containing patterned biomolecules with photoreversibility by the photocleavage of the fluorescent peptide through *o*-nitrobenzyl ether.^395^ To synthesize the hydrogel, they used a 4-arm PEG molecule with cyclooctyne termini, along with two azide-termined peptide moieties. The resultant hydrogel demonstrated good cytocompatibility. It could be functionalized with fluorophore-labeled peptides using a thiol–ene photoreactions, and then this peptide could be released upon irradiation with UV light, induced by the forementioned breakdown of the *o*-nitrobenzyl ether (Figure 60). This level of control enables dynamic cell functions that can be assayed selectively, and on-demand programming of the cellular microenvironment. A similar approach was published later, in which also an enzymatically degradable part was embedded in the peptide sequence, to permit degradation by different cell types with a host of secreted matrix metalloproteinases (MMPs), including MMPs 1, 2, 3, 8, and 9.^396^ The photoresponsive functional group enabled the formation and later modification of well shapes, whereas the enzyme cleavage sites enabled cell-based matrix remodelling during long-term cell culture. An *in vitro* mouse lung epithelial cell culture (primary mouse alveolar type II cells, ATII cells) was studied over a period of 7 days on the hydrogel surface. By shaping the hydrogel, taking inspiration from the human alveolar geometry, using the light-induced local degradation of the hydrogel, the cell differentiation of these ATII cells was followed by a range of immune-cytochemistry tests and confocal image analysis. It was shown—by following the production of T1α, a ATI cell indicator for 7 days—that the T1α production was relatively high (Figure 5B), with many elongated cells near the tops of the wells staining positive for a.o. T1α. This indicated an intermediate phenotype of alveolar epithelial cell that was transitioning from ATII to ATI. This experiment shows how such dual-degradable patternable hydrogels can be used to steer the development toward specific cell cluster shapes, and thereby influence cell–cell interactions, and ultimately function and fate in tissue development or regeneration.

Anseth and co-workers also synthesized a hydrogel using SPAAC by reacting a photolabile, enzyme-labile peptide-functionalized with azides with poly(ethylene glycol) tetracycloctyne.^396^ To make the photolabile azide-functionalized peptide, an *o*-nitrobenzyl ether photoresponsive group was introduced, while an artificial variant of an enzymatically degradable peptide sequence from collagen I was introduced to permit degradation by different cell types with a series of secreted MMPs. The photoresponsive functional group enabled the formation and later modification of well shapes, whereas the enzyme cleavage sites enabled cell-based matrix remodelling during long-term cell culture. Here SPAAC offers unique control over the reaction as well as provide delivers a hassle-free synthesis of a hydrogel. An *in vitro* mouse epithelial cell culture studied over a period of 7 days on the hydrogel surface revealed that the hydrogel is cytocompatible and can be used to understand how static or evolving physical cues influence cell cluster shapes, cell–cell interactions, and ultimately function and fate in tissue development or regeneration.

Song and her team members also developed cytocompatible PEG-*co*-polycarbonate hydrogels cross-linked by SPAAC click chemistry.^397^ Briefly, they prepared the hydrogel by reacting the azido-functionalized PEG-*co*-polycarbonate macromers with dibenzocyclooctyne- functionalized PEG under physiological conditions within minutes. Furthermore, they found that bone marrow stromal cells encapsulated in these gels exhibited higher cellular viability than those encapsulated in photo-cross-linked PEG dimethacrylate, demonstrating increased cytocompatibility. A live–dead assay also nicely confirmed that most of the cells are viable after 24 h of incubation within the gel.

**Figure 61:**
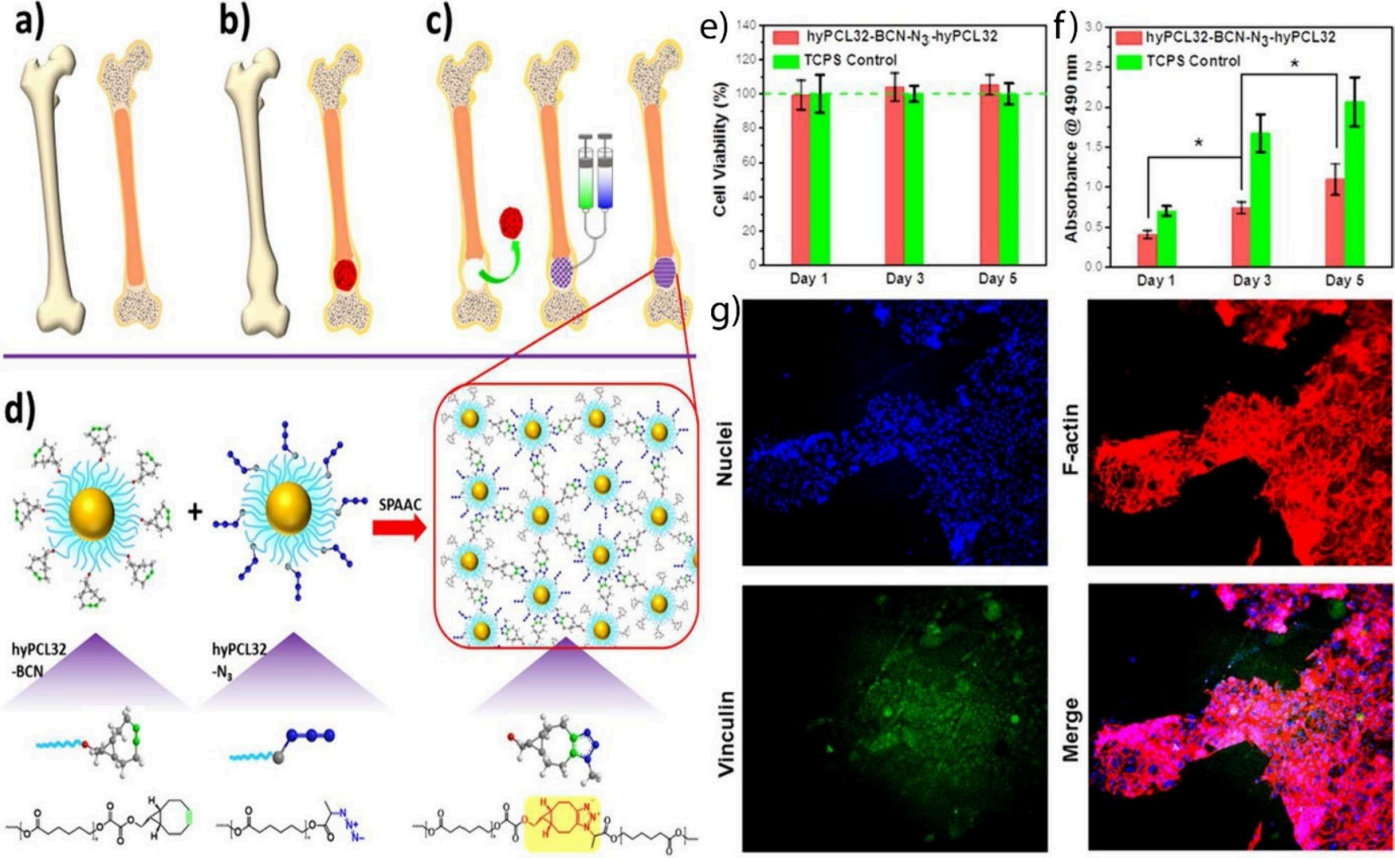
SPAAC-based hydrogels for bone tissue engineering studies. (a) Morphological and cutting view of normal bone. (b) Bone with metastatic disease. (c) Removal of diseased tissue and injection of cross-linkable polymer system for filling lytic defect. (d) Cross-linking of hyPCL32-BCN and hyPCL32-N_3_ dendrimers through SPAAC reaction. (e/f) MC3T3 preosteoblasts under coculture with cross-linked hyPCL32-BCN-N3-hyPCL32 scaffold for 1, 3, and 5 days: (e) cell viability; (f) cell proliferation. (g) Immunofluorescent imaging of intracellular nuclei (blue), F-actin (red), and vinculin (green) of MC3T3 preosteoblast cells after 3 days of growth on the cross-linked scaffold. Adapted with permission from ref (401). Copyright 2016, ACS.^401^

Zheng et al. also synthesized hydrogels based on a SPAAC cross-linking strategy by reacting a DCBO-functionalized PEG with glycerol exytholate triazide, and evaluated their cytocompatibility toward hMSCs.^398^ Live–dead assays revealed that the hydrogel is cytocompatible as 89% of the cells were viable on the hydrogel. Interestingly, their results suggested that the potential variability in the molecular mass of the respective components and the number of branching units, and the observed compatibility with both hMSCs and cell media can provide versatility in applications. Specific examples may be syringe-injectable materials or *in situ* formation of hydrogels, such as is necessary for the development of nerve glues or for cardiac tissue regeneration. In a similar type of work, Jiang et al. reported an injectable and fast degradable PEG hydrogel synthesized using SPAAC.^399^ To this aim, they functionalized the PEG chain with azide and cyclooctyne groups, and used SPAAC for the formation of the hydrogel (PEG-MFCO) within several minutes. Due to the presence of ester groups in the resulting hydrogel, the hydrogel demonstrated pH-dependent hydrolysis as well as efficient biodegradation. An *in vitro* cytocompatibility study toward COS7 cells revealed that more than 85% cells are viable 48 h after incubation concentration of 5 mg/mL PEG-MFCO hydrogel. Moreover an *in vivo* biocompatibility study in a Kungming mice model revealed that the hydrogel does not induce any severe host response. Not only that, in the presence of the hydrogel, the surrounding tissue at the implantation site fully recovered within a week after the injection, which indicates its applicability for tissue regeneration and related studies. In another similar type of study, Ito and co-workers reported an *in situ* cross-linkable hydrogel of hyaluronan via SPAAC, using azide-functionalized hyaluronic acids.^400^ The hydrogel showed a good biodegradation behavior, as it completely degraded in a PBS buffer over a time of 14 days as well as in cell culture media over a period of 4 days. In addition, it showed good cytocompatibility toward mouse fibroblast cells *in vitro*, while subcutaneous and intraperitoneal administration of gel samples into mice did not cause any inflammatory responses. Finally, the hydrogel residue was cleared safely after 21 days of subcutaneous administration, whereas in the case of intraperitoneal administration, it was cleared after 7 days of administration, clearly pointing to usefulness for further tissue engineering studies. In another recent work, Lu and co-workers used SPAAC to couple a hyperbranched poly(ε-caprolactone)—modified with either cyclooctyne or azide groups—to form, upon mixing of these two materials, a hydrophobic scaffold.^401^ The as-synthesized scaffold (named: hyPCL32-BCN-N_3_-hyPCL32) demonstrated excellent mechanical properties (compressive strength ∼2400 kPa) as well as good cytocompatibility toward MC3T3 preosteoblast cells. *In vitro* cytocompatibility studies over a period of 5 days suggested that the scaffold is noncytotoxic. To further confirm the cytocompatibility of their hydrophobic scaffold, they performed fluorescence imaging studies of several cell components including the cell nucleus, cytoskeleton, and F-actin, as well as immunostaining of vinculin proteins that are present in the cells. These studies revealed that MC3T3 preosteoblast cells proliferated robustly to high-density populations with a clear development of vinculins, as shown in Figure 61, which confirmed its potential for bone tissue engineering applications.

Fu et al. also investigated the cytocompatibility of injectable HA/PEG hydrogels cross-linked via the SPAAC reaction toward COS-7 cells.^192^ Their hydrogels showed short gelation times, good mechanical properties, excellent cytocompatibility, and slow degradation behavior, which makes the hydrogels promising candidate for tissue engineering applications.

Liu and co-workers reported an injectable dextran hydrogel, which was made using SPAAC, for cartilage tissue engineering applications.^402^ The hydrogel was synthesized by reacting azadibenzocyclooctyne-modified dextran with azide-modified dextran. Rheological studies revealed that the hydrogels were elastic in nature and had a storage modulus in between 2.1 and 6.0 kPa. The *in vitro* tests revealed that a polymer concentration of 10% supported a high viability of individual rabbit chondrocytes, and that the chondrocyte spheroids well proliferated within a period of 21 days. They also noticed that individual chondrocytes and chondrocyte spheroids in the hydrogel produced cartilage matrices such as collagen and glycosaminoglycans. In addition, the chondrocyte spheroids produced a higher content of matrices than individual chondrocytes. Han et al. also designed injectable hydrogels for cartilage regeneration, using *in situ* cross-linking of HA.^183^ Again, the cross-linking was achieved by a SPAAC reaction, where HA was modified either with a 4-arm PEG azide or with DBCO (2 separate materials), and subsequently these materials were reacted for gel formation. To determine whether the hydrogel is an effective platform to regenerate cartilaginous tissue *in vivo*, they encapsulated chondrocyte cells within the hydrogel samples and injected them into the subcutaneous dorsum of athymic mice. Five weeks after transplantation, the chondrocytes encapsulated within the hydrogel formed solid and milk-white tissue. Histological analysis by H&E staining of implants retrieved after 5 weeks of implantation confirmed that chondrocytes encapsulated within the hydrogels regenerated cartilaginous tissue, as evidenced by chondrocytes in lacunae. Similarly, Neves and co-workers employed SPAAC to prepare alginate hydrogel with protease-sensitive domains as cell responsive/instructive 3D microenvironments.^403^ Briefly, to design the hydrogel they exploited SPAAC to graft cyclooctyne-modified alginate (ALG-K) with biazide-functionalized peptides that have a with proline-valine-glycine-leucine-isoleucine-glycine (PVGLIG) sequence, as these sequences can be cleaved by MPPs. The hydrogel with a moderate concentration (125 μM) of PVGLIG exhibited slightly increased stiffness compared to other two compositions made of 50 μM and 250 μM PVGLIG, accompanied by enhanced cell spreading and a greater extent of cell–cell interconnections. This phenomenon is attributed to the cell-driven proteolytic remodeling of the hydrogel network. Furthermore, cells encapsulated within the hydrogel demonstrated the capacity to produce ECM, expressed MMP-2 and -14, and released enzymes that degrade PVGLIG. By mimicking essential features of the ECM, these hydrogels thus offer biologically relevant 3D matrices for soft tissue regeneration.

So in short, there are several studies that display the potential of SPAAC-based injectable hydrogel for tissue engineering application. The absence of any Cu ions is a big plus, but there are some limitations to the use of SPAAC-based click reactions when using cyclooctynes, such as multiple synthesis steps, hydrophobicity of the clicked moiety and—apart from with BCN—formation of various regio-isomers of triazoles.^182^ Use of cyclopropenes (much easier to synthesize, faster reaction in crowded environments, less hydrophobic clicked product)^221^ would overcome most of these drawbacks, but already with these limitations, SPAAC using cyclooctynes offers a fine platform to develop alternative polymeric biomaterials with a great degree of spatiotemporal functionalization and cell adhesive properties for biomedical applications.

#### 3.5.3 Thiol Ene/Yne Click Reactions-Based Hydrogels

Thiol ene/yne click reactions take place between a thiol group and an alkene or alkyne moiety. Such reactions are easy to perform, fast, and provide an excellent product yield, high selectivity, insensitivity toward oxygen and water, the potential for photochemical induction of the reaction, and a good biocompatibility. Therefore, thiol–ene and thiol–yne click reactions are considered among the most effective ways to produce novel polymeric biomaterials.

In 2011, Lin et al. reported a PEG-based hydrogel cross-linked via a thiol–ene click reaction for regenerative medicine applications.^404^ The synthesis of this hydrogel involved a step-growth thiol-click reaction using 4-arm PEG-norbornene macromers and a thiol-modified peptide. The hydrogel exhibited improved physical properties and viability for a 3D culture of pancreatic b-cells as compared to the control, a PEG diacrylate hydrogel: 93% for the hydrogel synthesized via thiol–ene click chemistry vs 45% for the control hydrogel. The improved cell viability for thiol–ene hydrogel is attributed to the faster gelation time of step-growth photopolymerizations, as compared to chain-growth photopolymerizations, thereby limiting the cellular damage caused by the radical species. Notably, when the cells were cultured in these two gel systems for extended period of time (10 days), cells in the thiol–ene hydrogels survived and proliferated, whereas cells in the PEG diacrylate hydrogel died off rapidly. Furthermore, the authors noticed that cells encapsulated in the thiol–ene hydrogels formed spherical clusters naturally, and were retrieved via rapid chymotrypsin-mediated gel erosion. Finally, and on top of that, the recovered cell spheroids also released insulin in response to a glucose treatment, demonstrating the cytocompatibility of this PEG-based thiol–ene hydrogel. Hsu and co-workers reported a glucose-sensitive self-healing hydrogel to prepare a vascularized construct.^405^ For the synthesis they used PEG diacrylate and dithiothreitol to form a long-chain polymer hydrogel. To introduce the glucose responsiveness they used borax, which is a well-recognized glucose-responsive motif. Additionally, borax also acts as a cross-linker, which increases the mechanical strength of the hydrogel as shown by the high storage modulus and loss modulus of ∼10 kPa and ∼2 kPa, respectively, The hydrogel is fully glucose responsive, as it completely dissolved in a glucose solution (4.5 g/L) within 1 h. Furthermore, upon seeding vascular endothelial cells in the lumen of the channels by perfusion, the authors observed that cells are lined on the channel wall and migrate into the nonsacrificial hydrogel after 3 days. In the case of the long-term study (∼14 days), it was found that the endothelial cells form capillary-like structures (vascular network), while neural stem cells form a neurosphere-like structure (neural development), which confirmed the formation of “vascularized neural tissue” and its usability in the field of regenerative medicine. Bown et al. reported a photopolymerized biocompatible hydrogel using a light-induced thiol–ene reaction by reacting an 8-arm PEG thiol macromer with thioester di(vinyl ether) as a cross-linker, and some norbornene-RGD for cell adhesion.^406^ An *in vitro* culture of hMSCs on the hydrogel system revealed that the hydrogel is cytocompatible toward hMSCs, which turned out to be well encapsulated in thioester hydrogels. In addition to that, it was observed that hMSCs are elongated in shape and display increased proliferation compared to the control sample.

Ganja and co-workers developed a cell-instructive pectin hydrogel using thiol–norbornene photoclick chemistry for skin tissue engineering applications.^407^ The term “cell-instructive pectin” was used, because pectin has a tendency to attract and bind the cells together. Due to that tendency, pectin has been used inside the hydrogel and helps to encapsulate the cells into the hydrogel. Specifically, they made the hydrogel system using norbornene-functionalized pectin, monocysteine cell-adhesive ligands for integrin attachment, and an enzymatically cleavable biscysteine peptide cross-linker. This system formed the hydrogel upon exposure to 365 nm light for only 20 s, without affecting the viability of embedded dermal fibroblasts. These fibroblasts had a longer morphology and contracted the matrix to a greater extent in more permissive hydrogels. Additionally, they found that the cell-instructive hydrogels with encapsulated dermal fibroblasts and seeded keratinocytes supported the *in vitro* formation of full-thickness skin with the architecture and morphology similar to human skin. Histological analysis revealed that the dermal compartment is uniformly populated with elongated dermal fibroblasts, while a well-defined epidermis was formed on the top of the fibroblast-laden hydrogel. Keratinocytes developed a dense epidermal tissue after 2 weeks of culture with clear cell morphology differences as a function of their location in the epidermis. Moreover, after 14 days two well distinct regions equivalent to the dermal (∼300 μm similar, to papillary dermis layer) and epidermal layers (∼120 μm) were observed, exhibiting the presence of specific markers associated with proliferation and extracellular matrix deposition. This confirms the potential of these cell-instructive hydrogels for skin tissue engineering studies.

In a slightly different type of work, Gautrot and co-workers explored the synthesis of biofunctionalized patterned polymer brushes using thiol–ene click chemistry to control the cell adhesion and formation of human umbilical vein endothelial cells (HUVEC) cell arrays.^408^ Instead of a hydrogel, to study the cell adhesion and proliferation they used a patterned surface of polymer brushes. These were obtained from antifouling alkene-functionalized (allyl and norbornene residues) PEG methacrylate-derived polymer brushes, which were reacted using the thiol–ene coupling with a series of thiols including cell-adhesive peptides RGD and REDV. *In vitro* HUVEC culture studies on such polymer brushes revealed that a RGD-containing polymer surface was superior in terms of cell attachment and spreading compared to REDV-functionalized brushes. This may be due to α_4_β_1_ integrins, which are the receptors of REDV sequences, by themselves not being sufficient to sustain HUVEC spreading, and contrasting to RGD sequences that do bind α_v_β_3_ and α_5_β_1_ integrins.

**Figure 62:**
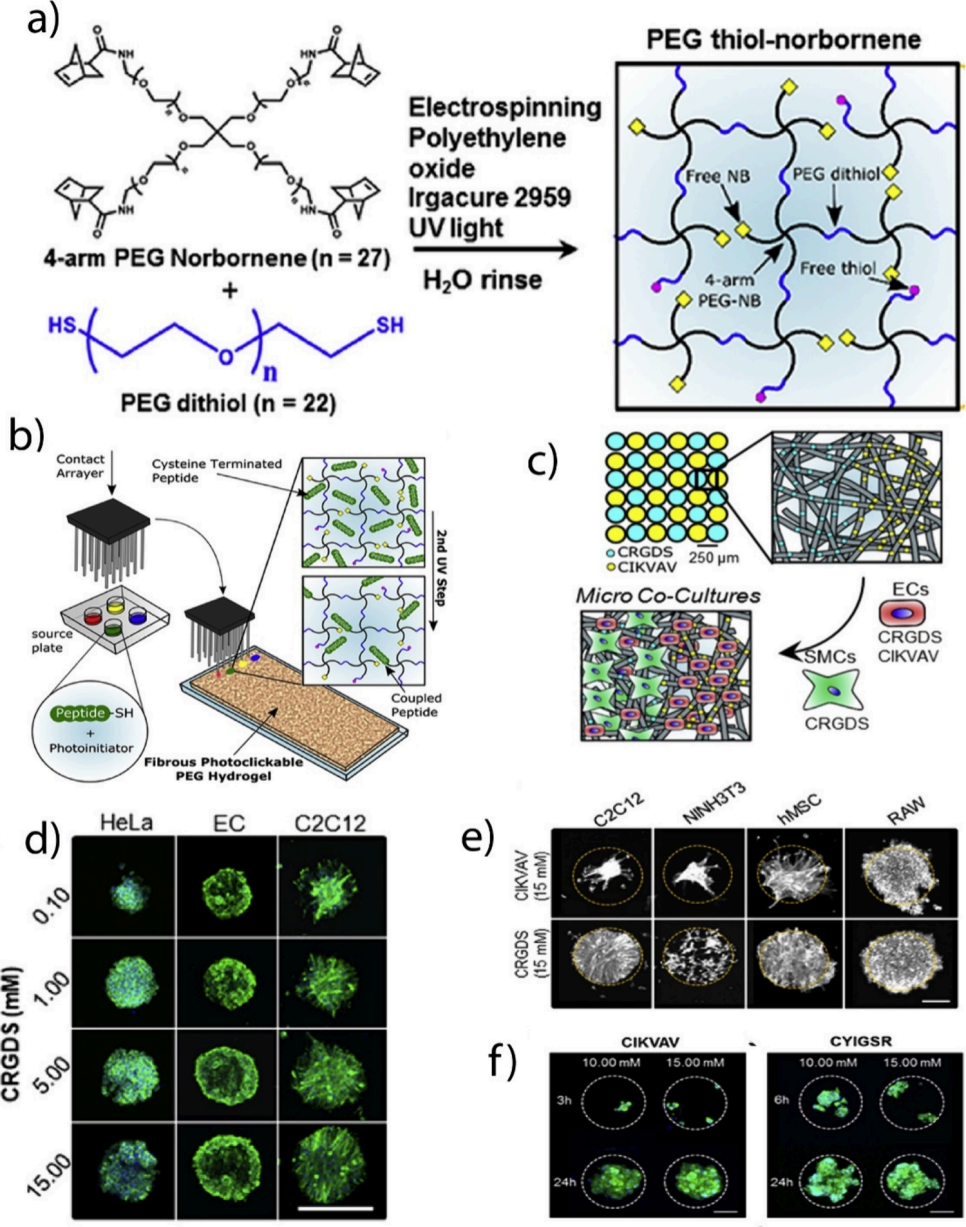
Fabrication and characterization of the photoclickable peptide microarray platform. (a) Four-arm PEG norbornene + dithiol prepolymer solution is electrospun followed by UV polymerization. (b) Contact array printing of cysteine-containing peptides on nanofibrous hydrogel matrices with photoinitatior I2959 permits stabilization of biomolecules via UV light initiated reaction of thiols to free norbornene groups. (c) Design of 3D micro coculture systems using two peptides (CRGDS and CIKVAV) for selective attachment of two cell types (SMCs, ECs). d) Imaging reveals variable cell spreading among various cell types versus initial CRGDS concentration; e) Representative images of microtissue shape versus unique peptide (CRGDS; CIKVAV) for select cell types (C2C12, NIH3T3, hMSC and RAW); dotted circles represent peptide deposition area; scale bar: 100 mm. (f) Extended cell seeding time increases EC attachment to selected peptides (CIKVAV and CYIGSR) at higher concentration (10 mM; 15 mM); scale bars: 100 mm. Adapted with permission from ref (409). Copyright 2017, Elsevier.^409^

Sharma et al. used the thiol–ene reaction of thiol and norbornene functionalities to fabricate a photoclickable peptide microarray platform for facile and rapid screening of 3D tissue microenvironments.^409^ The click reaction was involved to cross-link a 4-arm PEG-norbornene with PEG-dithiol, and produced a fibrous structure via electrospinning. Photoinduced thiol–ene click reactions on still unreacted norbornene present in the hydrogel were subsequently used to introduce peptides within the fibrous PEG structure using thiol-modified cell-adhesive peptide (CRGDS, CRGES, CIKVAV, CYIGSR, CDGEA, and CVAPG peptides). Furthermore, they validated the capabilities of this platform to screen arrays consisting of multiple peptide motifs and concentrations for selectivity to cellular adhesion and morphology. Here, the CRGDS-functionalized hydrogel demonstrated the best results in terms of cell viability and proliferation as shown in Figure 62. They conducted the study with a wide range of cell types (even more than shown in Figure 62). The total number of attached cells and microtissue morphologies that was found in using this new *in vitro* platform correlated well with the *in vivo* ECM composition and microenvironment for the different cell types.

In subsequent work, this group described the creation of biomimetic soft fibrous hydrogels for smooth muscle-like contractile and pharmacological response.^410^ They used a four-arm PEG-norbornene and PEG dithiol and thiol–ene click chemistry for their synthesis, which was also assisted by a photoinitiator. The fibrous hydrogel system displayed a variety of smooth muscle features, including stimuli-responsive mechanical strength, and changeable shape. They demonstrated *in vitro* pharmacological responsiveness and contractility on the hydrogel of human pulmonary artery smooth muscle cells, suggesting that tissue engineering of smooth muscle tissue would be possible. Using this hydrogel, the authors built a cellular array with variation of its thickness and stiffness, and examined the smooth muscle contractility, phenotype, and cell mechanics in dependence of these parameters. Their findings indicate that biomimetic soft, 3D fibrillar environments can improve the pharmacological behavior and contractility of reconstituted synthetic human vascular smooth muscle; additionally, they observed that such tissue was, to some degree, coregulated in the presence of adhesion proteins.

Zhou and co-workers used a photoinduced thiol–ene polymerization to prepare a hydrogel made of maleic chitosan and thiol-linked poly(vinyl alcohol) (TPVA), using a biocompatible photoinitiator, namely Darocur 2959.^411^ The hydrogel showed rapid gelation time (<120 s), good elastic modulus with a range between ∼600 Pa and ∼5500 Pa with an increase in TPVA content from 0 to 2.9%. *In vitro* cytotoxicity assays by fibroblast cells (L929) revealed that the hydrogel is cytocompatible (∼80% cells are viable after 24h) and may be applicable for tissue engineering applications. Toward the same goal, Ding and co-workers reported the synthesis of a pH- and thermosensitive injectable chitosan (CS)-polyNIPAM hydrogel using thiol–ene chemistry.^412^ To synthesize the hydrogel, they reacted C_6_-OH allyl-modified chitosan (OAL-CS) with a thiol-modified PNIPAM, so that the thiol groups of PNIPAM and the allyl groups in OAL-CS rapidly formed a cross-linked hydrogel network. An *in vitro* cytocompatibility study revealed that the hydrogel extract is noncytotoxic (almost 100% viability after 24 h) toward human bone marrow mesenchymal stem cells (hMSCs). Notably, when 50 μL of homogeneous solution of OAL-CS/PNIPAM (10:1 volume ratio) was subcutaneously injected into Kunming mice and exposed to UV for 1 min it formed a hydrogel, confirming the rapid gelation process. Moreover, *in vivo* histological analyses revealed that the subcutaneous tissue displayed no signs of inflammation after 7 days of injection of the hydrogel, indicating good biocompatibility of the hydrogel, which further suggest that the OAL-CS/PNIPAM hydrogel may serve as a promising candidate for tissue engineering studies. On a different note, in a recent study Cianciosi et al.,^413^ exploited allyl-modified gelation and a four-arm thiolated PEG to design a hydrogel. Their studies revealed that, owing to its soft microenvironment (similar to soft tissues), the gelatin-based hydrogel can facilitate the adipogenic differentiation of adipose-derived stromal cells. Based on their findings, they proposed that their developed hydrogel may have broader applications in other soft tissues, including the brain, lung, and endothelial tissue.

From the above studies, it is clear that thiol–ene based click chemistry provides an effective platform to synthesize biocompatible hydrogels with the required properties for tissue engineering applications. Positive features are the typically good biocompatibility of norbornene-based alkene systems, the easy thiol-modification of many biomolecules, and the possibility of patterning of the material via a light-induced click reaction. As always, one should investigate the biocompatibility of all reagents within the gel, especially in regard of inflammation, to develop biomaterials that an be truly useful for tissue engineering applications. Finally, the thiol–yne reaction has, up to now, hardly been explored in the field of tissue engineering, while it offers complementary features to the thiol–ene reaction. For one, it would allow the possibility to further functionalize a hydrogel made using CuAAC that displays excess alkyne moieties, so that, e.g., in a subsequent process global or—when using light-induced reactions—local modification of such a preformed hydrogel is feasible with tailor-made additional functionalities.

#### 3.5.4 Diels–Alder Click Reactions-Based Hydrogels

One of the characteristics of Diels–Alder (DA) reactions between dienes and alkenes (or dienophiles) that is useful for tissue engineering is the acceleration of the reaction in the presence of water due to the hydrophobic effect.^182^ This highly atom-efficient reaction^414,415^ typically also does not produce any toxic byproducts, which makes it useful to produce hydrogels for tissue-engineering application. For example, Bowman and co-workers developed a hydrogel-based on a DA click reaction by reacting a maleimide-PEG macromer with a furan-dexamethasone peptide.^416^ They revealed that, over time, the hydrogel released dexamethasone in a sustainable manner, which resulted in improved osteogenesis of hMSCs. The system effectively works in both 2D and 3D cell culture platforms, and demonstrated superior alkaline phosphatase activity (6 times more) and increased mineralization compared to a control sample. They proposed that their hydrogel system can effectively work as a drug delivery carrier as well as for tissue regeneration. Nimmo et al. also developed a PEG-based hydrogel using DA reactions for tissue engineering applications.^203^ Briefly, they prepared the hydrogel by reacting a bis-maleimide-terminated PEG with furan-modified HA in a MES buffer at room temperature. The HA-PEG hydrogels showed an elastic modulus similar to that of central nervous system tissue and demonstrated minimal swelling. In addition to that, the hydrogel exhibited a steady degradation in a PBS buffer at 37 °C, which can be finely tuned by adjusting the furan-to-maleimide ratio. Notably, the hydrogel prepared with 1:0.5 molar ratio of furan/maleimide degraded faster, with a rate of 3.4% per hour, than a hydrogel made with 1:2 molar ratio of furan/maleimide exhibited (degradation with a rate of 2.1% per hour). While hydrogel prepared while use of the intermediate 1:1 molar ratio of furan:maleimide displayed indeed an intermediate degradation profile (degradation rate 2.2% per hour). Subsequent *in vitro* cell viability studies by live–dead assays revealed that the hydrogel is cytocompatible toward MDA-MB-231 cells (more than >98% cells are viable after 14 days of culture), and all taken together is likely useful for further soft tissue engineering studies. In similar work, Shoichet and co-workers reported a HA and PEG-based hydrogel which resembles an extracellular matrix-like structure.^417^ The hydrogel possess a furan-modified HA backbone whereas bis-maleimide-PEG acts as a cross-linker via DA click reactions. Interestingly, the synthetic route toward their hydrogel allows control over many facets, such as photopatterning of biomolecules within it by two-photon laser processing, which resulted in spatially defined growth factor gradients. Additionally, the mechanical properties of the hydrogel are fine-tuned by either changing the furan-substitution on the HA backbone. The addition of galactose, added as a colligative depressant inhibits ice recrystallization, further influenced the porosity, pore size, and Young’s modulus of the hydrogel. Chen and co-workers developed a tissue-adhesive DA-based multifunctional, dual-cross-linked hydrogel for cartilage tissue engineering applications.^415^ The dual-cross-linked network hydrogel was synthesized by reacting HA with furan adipic dihydrazide, and HA with furan aldehyde, followed by the addition of dimaleimide PEG. The hydrogel exhibited moderate mechanical properties (∼32 kPa compressive strength at ∼55% strain), self-healing, and swelling behavior. It also showed good cell-adhesive properties, with an adhesive strength of the dual cross-linked hydrogel of 10.3 kPa, which was significantly higher than the control hydrogel (1.2 kPa), due to the formation of covalent bonds between the hydrogel and cartilage. The latter is, of course, favorable for cartilage tissue regeneration.

**Figure 63:**
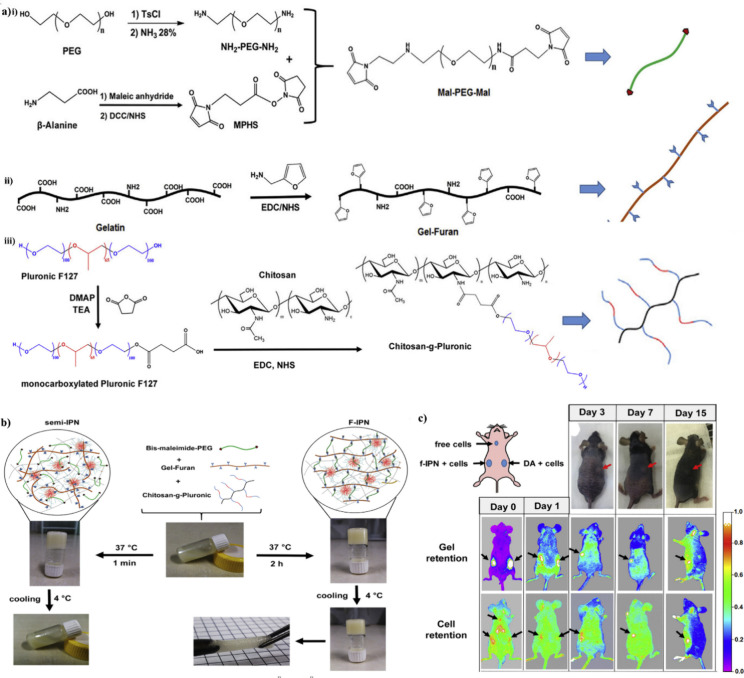
a) Synthesis and material properties of DA-cross-linked hydrogel (i) Synthesis of bis-maleimide PEG. (ii) Synthesis of gelatin-furan and (iii) chitosan-pluronic hydrogel; b) semi-IPN and f-IPN hydrogel were obtained via sequential network formation by homogeneously dissolving of polymers in PBS at 37 °C; c) Fluorescence imaging process of injected zones of nude mice on 3, 7, and 15 days postinjection of cardiomyocytes (CMs) within f-IPN hydrogel. Adapted with permission from ref (419). Copyright 2018 Elsevier.^419^

Stepping up from insightful, but of course intrinsically limited, *in vitro* studies, several groups used DA-based hydrogels also for *in vivo* and implantation studies. Work done by Bai et al. evaluated the efficiency of a self-reinforcing injectable hydrogel based on a DA reaction for bone tissue engineering application.^418^ The hydrogel displayed a high water content (>98%) and high mechanical strength (∼25 MPa under dynamic conditions). They prepared the hydrogel through the supramolecular interaction between cyclodextrin and adamantane, and the sol–gel transition of PNIPAM, enabling hydrogel formation *in situ* after injection. The covalent cross-linking was effected by reacting furfurylaminegrafted chondroitin sulfate (ChS-F) and maleimido-terminated PEG. The dual (chemical and physical) cross-linking strategy increased the mechanical strength of the hydrogel significantly, while the hydrogel remained highly cytocompatible toward rabbit mesenchymal stem cells (>80% cells are viable after 24 h at a hydrogel concentration of 100 mg/mL). In fact, when the authors injected 0.05 mL of the hydrogel at a defect site of the right limb in Kunming mice, they noticed that the hydrogel induced significant bone regeneration without any added cells or growth factors (studied after 14 days of injection). They proposed that, the chondroitin sulfate (ChS) incorporated in the hydrogel, which is a key polysaccharide found in bone, cartilage, and connective tissue, plays a significant role in promoting chondrogenesis, and facilitates the hydrogel to bone regeneration. Furthermore, favorable histological evaluation confirmed that the hydrogel does not impose any inflammation at the implantation site. Work by Baharvand and co-workers reported an *in situ*-forming interpenetrating hydrogel using sequential thermal (physical) and DA click cross-linking for enhanced retention of transplanted cells.^419^ In detail, they prepared the hydrogel (f-IPN) by using furan-functionalized gelatin, chitosan-grafted pluronic and maleimide-functionalized PEG, and this mixture offered both noncovalent and covalent cross-linking (Figure 63a). The hydrogel showed good mechanical properties upon compression, self-healing properties (stratching up to 200% feasible for previously cleaved components), excellent thermosensitivity at 37 °C (Figure 63b), and good cytocompatibility (>95%) toward cardiac progenitor cells as confirmed by 3-(4,5-dimethylthiazol-2-yl)-5-(3-carboxymethoxyphenyl)-2-(4-sulfophenyl)-2H-tetrazolium (MTS) assay and live–dead assays. In addition to that, further *in vivo* implantations of cardiomyocytes (CMs)-laden hydrogels into nude mice revealed, after 2 weeks, enhanced cell retention (more than 60%) and survival compared to controls, suggesting good a biocompatibility of the hydrogel. The increase in cell number after 2 weeks appears to be due to a reduction in rapid cell elution from the injection site, enhanced cell proliferation, and/or decreased cell apoptosis, along with the notable gel retention properties. On top of that, the presence of cell-adhesive biopolymers, such as gelatin and chitosan, in combination with the use of FDA-approved polymers, may positively influence cell retention and survival. Moreover, the cardiomyocytes’ identity of retained cells was confirmed by the detection of human and cardiac-related markers (Figure 63c). Their results indicate that their hybrid thermosensitive hydrogel can be a promising candidate for further cell therapy and tissue engineering studies.

Lu et al. also combined *in vitro* and *in vivo* studies on their injectable hydrogel, which was made using two sequential click reactions: first, a DA reaction involving *N*-maleoyl alanine-terminated pluronic F127 and furan-grafted ChS to make F127@furan-modified chondroitin sulfate (F127@ChS/furan). This F127@ChS/furan was in the second step functionalized with hydrazide groups, and then reacted with oxidized chondroitin sulfate to synthesize the hydrogel.^420^ Their study revealed that the obtained hydrogel possesses highly tunable viscoelastic and rheological properties, proper swelling and degradation behavior, and a favorable injectability and self-healing ability. The hydrogel showed increased viability (80.1%, 109%, and 144% after 24 h, 48, and 72 h, respectively) and reduced apoptosis of rat mesenchymal stem cells, and an excellent tissue adhesive ability *in vivo.* Additionally, the *in vivo* evaluation of the BMP-4-loaded hydrogel as scaffold exhibited significant new bone tissue formation in the cranial bone defect of a Kunming mouse model after 12 weeks postimplantation, highlighting its potential for bone tissue regeneration. The authors proposed that the accelerated bone formation is driven by the progenitor cells from the cranial bone, which are attracted by the BMP-4 gradient and migrate toward the hydrogel. There they adhere due to its chemotactic properties. These cells then penetrate the hydrogel and differentiate into bone cells. As they mature, they produce an organic matrix, including collagen fibers, which initiates angiogenesis and the formation of bone marrow-like tissue. Over time, this tissue mineralizes into bone, gradually extending toward the scaffold and replacing the hydrogel.

DA chemistry was also used by Li et al., who designed catechol-modified *N*-(furfural) chitosan (CFC)-based dual cross-linked (co-ordination bond between Fe^3+/^ catechol moiety and DA click chemistry) hydrogel and explored their physicochemical properties.^130^ The coordination bond between Fe^3+^ ions and catechol offers the ability to control the self-healing of the gel and degree of cross-linking. The dual cross-linking strategy has as particularly positive element the good mechanical properties it effected within the hydrogel. Shoichet and co-workers also made a DA- cross-linked hydrogels consisting of methyl-furan grafted hyaluronan and bismelamide-terminated PEG, which can form gels at physiological conditions, unlike hyaluronan-furan, which can form gel only at acidic pH.^421^ These newly developed click-cross-linked hydrogels exhibit rapid gelation at physiological pH, making them ideal for encapsulating viable cells. This capability was demonstrated through the successful 3D culture of five different cancer cell lines, including MCF7, BT474, T47D, G523, and G411. Moreover, they noticed that more than 80% of the encapsulated cells remained viable after a 7-day period, as confirmed by a live/dead assay. Notably, MCF7 cells formed characteristic spheroids within the hydrogel, mirroring their typical *in vivo* morphology which indicates that these hydrogels may be useful for further tissue engineering and 3D cell culture studies.

Based on these studies it seems clear that DA-based polymers offer some unique advantages over other click reactions, although, of course, several characteristic challenges still exist. On the one hand, DA-based polymers have been used relatively frequently in a successful manner for *in vivo* and implantation studies. The absence of any required catalyst and low acute and long-term toxicity of the frequently used maleimide and furan components make this an interesting class of materials. However, DA-based materials do generally require longer gelation times, compared to some other click reactions such as oxime/hydrazone click or the photoinduced thiol–ene reactions, and might suffer from a reduced solubility of the functional groups and in some cases, less efficacy toward the formation of injectable gels under physiological conditions. Yet, these hydrogels have already repeatedly been used in several research studies to regenerate cartilage, cardiac, adipose, and cervical bone defects, which makes them strong candidates for potential clinical translation.^422−425^ In addition, the use of faster DA chemistries like the SPOCQ reaction allows to form such gels in appreciately shorter times, and as such also improve their usability.^426,427^

#### 3.5.5 Oxime/Hydrazone Click Reactions-Based Hydrogels

**Figure 64:**
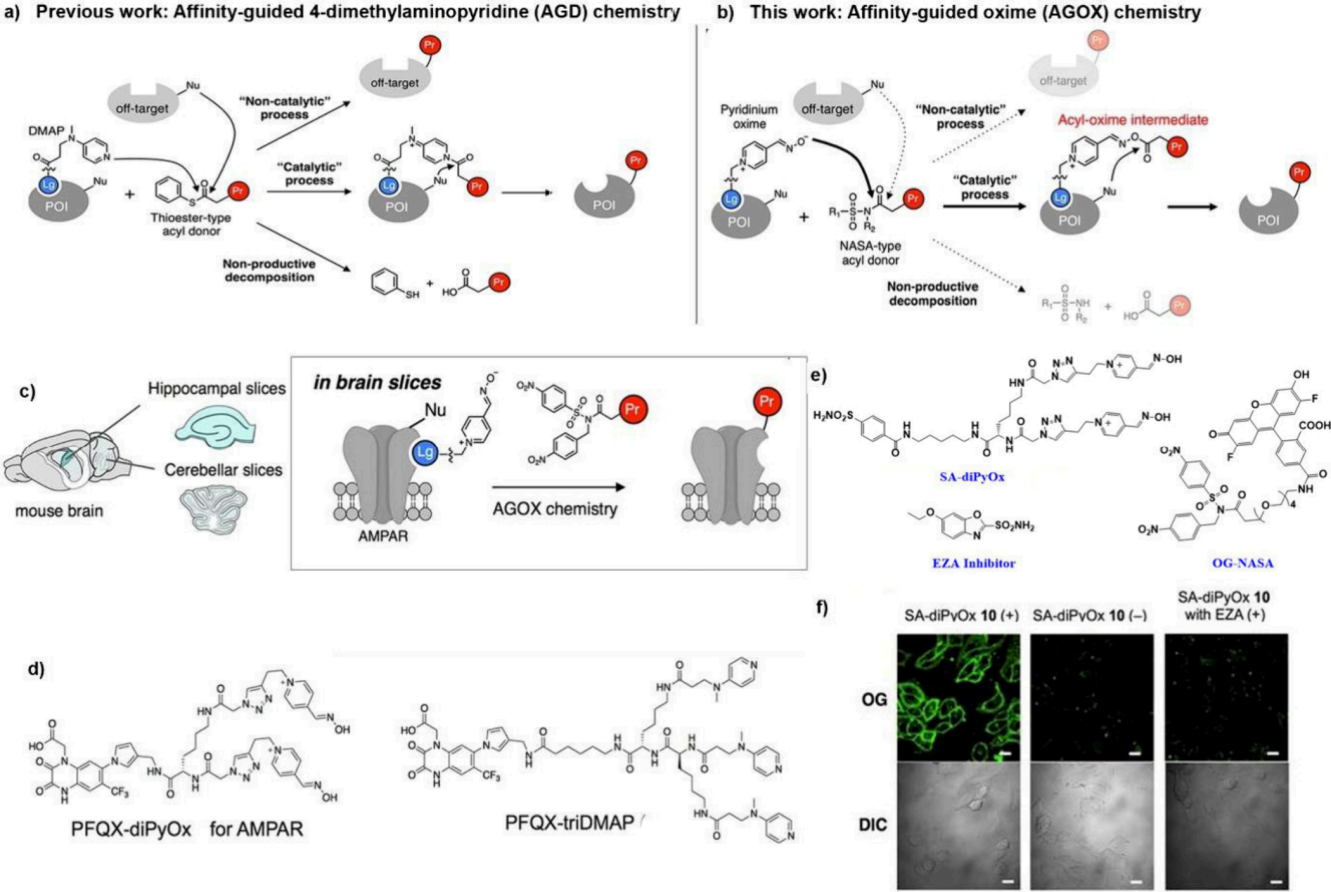
Schematic representation of the advantage of affinity-guided oxime reactions over affinity-guided DMAP based reactions and its application toward the brain slices for the deep tissue labelings. (a) Schematic illustration comparing the protein chemical labeling by affinity-guided DMAP (AGD) chemistry and (b) affinity-guided oxime (AGOX) chemistry. NASA, *N*-acyl-*N*-alkyl sulfonamide; POI, protein of interest; Pr, probe; Lg, ligand; and Nu, nucleophilic amino acid (c) Schematic illustration of chemical protein labeling in brain slices by AGOX chemistry. (d) Molecular structures of PFQX-diPyOx and PFQX-triDMAP for AMPAR labeling. (e) Molecular structure of SA-diPyOx, EZA inhibitor, and OG-appended NASA acyl donor. (f) Confocal micrograph images of A549 cells labeled with SA-diPyOx and OG-NASA. Adapted with permission from ref (432). Copyright 2017, ACS.^432^

Oxime click reactions are part of the class of dynamic covalent reactions, and take place between an aldehyde/ketone group and an amino-oxy group. These reactions are fast (within minutes) and can features several types of orthogonal functionalities as are present in cells and biomolecules. Like SPAAC and many DA reactions, oxime click reactions do not require any catalyst, UV light or any external stimulus (e.g., raised temperature) to proceed to completion, which makes them highly suitable for biology-relevant applications. Furthermore, the typical byproduct of the reaction is simply water. The products obtained display imine hydrazone and oxime chemical bonds, which are stable in physiological conditions, making them attractive to modify a wide variety of biomacromolecules, including proteins, peptides and DNA.^428^ Additionally, this reaction is also used as a tool for polymer–protein ligation, cell surface modification and *in vivo* labeling of deep tissues.^429^ For example, Grover et al. synthesized polymer–peptide (RGD) conjugates using oxime ligation, and explored its cytocompatibility toward mouse mesenchymal stem cells (MSCs).^429^ They observed that MSCs that were encapsulated within the oxime cross-linked PEG hydrogel were viable and metabolically active for at least 7 days, which suggest significant biocompatibilty and usefulness for further tissue engineering studies. In similar work, Hardy and co-workers designed an oxime-cross-linked hydrogel composed of PEG, HA, and collagen, and subsequently evaluated its potential toward soft tissue engineering application.^430^ Briefly, they prepared the hydrogel by reacting aminooxy-terminated linear PEGs and aldehyde-modified HA. The cross-linked hydrogel showed tunable mechanical and swelling properties analogous to soft tissues such as those found in the central or peripheral nervous system. The incorporation of collagen-1 further enhanced the cytocompatibility of the hydrogel toward hMSCs, as confirmed by a live–dead assay. More specifically, cells were seeded on gels composed of various ratios of aminooxy-terminated PEGs, aldehyde-modified HA, and collagen-1, and showed spread and healthy morphologies, and the cell viability was more than 88% for all of the formulations tested. DeForest and co-workers reported a biorthogonal oxime click chemistry-based hydrogel for 3D cell culture-related applications.^431^ In their work, they used mild UV light photopolymerization to produce a cell-encapsulated hydrogel. For this they used multiarm PEG cross-linkers end-functionalized with either 2-(2-nitrophenyl) propyloxycarbonyl (NPPOC)-modified alkoxyamine or benzaldehyde moieties. The photomediated oxime reaction enabled them to immobilize proteins with alkoxyamines at the sites exposed to UV light: upon photo irradiation (365 nm at 10 mW/cm^2^ for 10 min for the hydrogel synthesis and 5 min for the encapsulation of cells) the photoresponsive NPPOC group dissociates and yields alkoxyamine groups free to react with benzaldehyde groups. *In vitro* cell culture studies then revealed that the resulting hydrogel possessed a good cytocompatibility toward NIH3T3 fibroblast cells with a high viability of 90 ± 8% after 24 h of post encapsulation, with no significant decrease in viability seen after 48 h (90 ± 5%) and 72 h (82 ± 8%) time points. Cells within hydrogels adopted rounded morphologies similar to those observed in other hydrogel cell culture platforms, attributed to the physical confinement of cells by the surrounding nondegradable polymer network and the lack of substrates promoting cell adhesion. Such observations indicate that further improvements can be expected by integrating cell adhesive motifs, such as RGD, and facilitating substrate remodeling, ultimately resulting in a well-spread and physiologically relevant cell morphology. Moreover the programmability offered by the photomediated oxime ligation reactions can recapitulate dynamically anisotropic mechanical and biochemical properties of the native ECM, which make it an attractive tool to design novel hydrogels for tissue engineering applications. Tamura et al. reported affinity-guided oxime (AGOX) chemistry for selective protein acylation in live tissue systems.^432^ Their system involves pyridinium oxime and *N*-acyl-*N*-alkylsulfonamide, and can be used to selectively label natural proteins present in test tubes and different cell lysates under physiological conditions. AGOX chemistry (Figure 64b) aims to overcome several limitations regarding to the biocompatibility and bioorthogonality posed by affinity-guided 4-adimethylaminopyridine (DMAP) (AGD) chemistry (Figure 64a). These limitations include (1) Necessity of pH ≥ 8 for efficient labeling because of the low nucleophilicity of DMAP at neutral pH; (2) Undesired (noncatalytic) acylation to nontargeted proteins, which inevitably occurs in crude biological environments because of the inherently high electrophilicity of thioester acyl donors. Therefore, the reaction is usually carried out at low temperature (∼4 °C) to minimize nonspecific labeling. (3) Thioester acyl donors are readily decomposed by esterases in biological samples, which requires high concentrations of reagents. In contrast, an excellent biocompatibility and bioorthogonality of AGOX chemistry was demonstrated by the selective labeling of an endogenous neurotransmitter receptor in mouse brain slices (Figure 64c), which are highly complicated tissue samples. More specifically, the authors selected α-amino-3-hydroxy-5-methyl-4-isoxazolepropionic acid (AMPA)-type glutamate receptors (AMPARs), as target proteins in the brain slices, and designed pyridinium oxime (PyOx) catalyst containing di-PyOx and 6-pyrrolyl-7-trifluoromethyl-quinoxaline-2,3-dione (PFQX) as a ligand as shown in Figure 64d. The confocal laser scanning microscopy analysis revealed a strong fluorescence from the plasma membrane region of live A549 cells labeled with Oregon Green (OG)-labeled phenylsulfonamide divalent-pyridinium aldoxime (SA-diPyOx), i.e., OG-labeled SA-diPyOx. This fluorescence signal was absent in control experiments lacking the catalyst or containing the competitive inhibitor EZA (Figure 64e), suggesting that the observed fluorescence on the cell surface indeed primarily originates from OG-labeled SA-diPyOx (Figure 64f). Confocal micrographs illustrate A549 cells labeled with SA-diPyOx and OG-N-acyl-*N*-alkyl sulfonamide (OG-NASA). Cells were incubated with SA-diPyOx (5 μM) and an (OG)-NASA acyl donor (5 μM) for 1 h at 37 °C in a DMEM-HEPES buffer (pH 7.4) (left panel). Negative controls included the labeling reaction conducted without compound SA-diPyOx (middle panel) or in the presence of EZA (50 μM) (right panel).

These findings suggest that oxime-based click reactions are an attractive alternative of affinity-guided 4-dimethylaminopyridine chemistry for the deep tissue labeling that is often used in the field of regenerative medicine.

Chen and co-workers synthesized a biocompatible polysaccharide-based self-healing hydrogel using this click reaction.^433^ For the synthesis of the hydrogel, they reacted *N*-carboxyethyl chitosan (CEC) and adipic acid dihydrazide (ADH) with oxidized sodium alginate (OSA). The hydrogel showed excellent self-healing ability as revealed by rheological studies, and good cytocompatibility toward NIH3T3 fibroblast cells by live–dead assays, which suggest its potential applicability for tissue engineering studies.

While displaying several cool characteristics (fast, dynamic covalent reaction with water as byproduct, easy), the oxime reaction still has some limitations, including that this reaction requires either neutral or slightly basic (pH) conditions to reduce the possibility of the oxime exchange reaction.^434^ Selectivity toward specific moieties (e.g., aldehydes) often requires oxidation, and then of course this needs itself to be selective to warrant overall selection and biocompatibility. Overall, however, it can be stated that the oxime/hydrazone reactions offer an efficient platform to develop novel biomaterials for tissue engineering applications.

Novel click reactions are, of course, still being developed, and it may take some time before their implementation into the field of tissue engineering has matured. Two examples from this much wider set are SuFEx and nitrile oxide cycloaddition reactions. While examples are thus necessarily still rare, there is one report where SuFEx is used to introduce a sulfonyl fluoride moiety into bovine serum albumin (BSA) protein, which can then further self-condense into a biocompatible hydrogel. Notably, the BSA-SO_2_F hydrogel can facilitate HEK 293 cells without any observed cytotoxicity,^217^ while additionally S(VI) exchange chemistry to, e.g., phenols (as present in, e.g., tyrosine) has been shown to be reversible, opening the way to dynamic covalent use in, e.g., hydrogels.^77^ Since the basic studies of many such click reactions are still ongoing, it is likely that specific advantages for any of these will arise, and then more of such studies are surely to be expected.

Below, to combine brevity and a higher degree of completeness, we have tabulated some more examples (Table 3) of click chemistry-based polymeric biomaterials and their potential application for tissue engineering applications.

**Table 3:** List of Polymeric Platforms Using Various Types of Click Reactions for Tissue Engineering Applications

Platform	Cells used for the study	Click reaction employed	Commitment toward tissue engineering	Outcome	Ref.
Hydrogel composed of (PEG-dithiol) and pentaerythritol tetraacrylate	hMSCs	CuAAC	Multidirectional tissue engineering application	hydrogel with good biocompatibility toward hMSCs	(435)
Mixture of chitosan-*graft*-polycaprolactone polymer and magnesium-doped hydroxyapatite	Osteoblast-like MG63 cells	CuAAC	Bone tissue engineering	Synthesized scaffolds display no cytotoxicity; introduction of triazole ring enhanced bone mineralization ability of the scaffolds	(436)
Hyaluronic acid-collagen interpenetrating (IPN) hydrogel	Human mesenchymal stem cells (hMSCs)	CuAAC, Hydrazone click reaction	Multidirectional tissue engineering	Enhanced focal adhesion, cell spreading, fiber remodeling	(437)
Hydrogel of poly(glycolic acid) (PGA)-(PEG)–PGA-di(but-2-yne-1,4-dithiol) (PdBT) as cross-linker and poly(*N*-isopropylacrylamide-*co*-glycidyl methacrylate) [P(NIPAAm-*co*-GMA)]	Rabbit mesenchymal stem cells (MSCs)	RuAAC	Multidirectional tissue engineering applications	noncytotoxic toward MSCs; readily formable and hydrolytically degradable; suitable candidate for tissue engineering application	(438)
Hydrogel composed of two dextrans, with azadibenzocyclo-octyne or azide modification	Chondrocyte cells	SPAAC	Cartilage tissue engineering	High viability of individual rabbit chondrocytes, and chondrocyte spheroids encapsulated in hydrogel over period of 21 d. Individual chondrocytes and chondrocyte spheroids in the hydrogel produced cartilage matrices such as collagen and glycosamino-glycans.	(402)
Hydrogel composed of (DBCO)-modified hyaluronic acid (HA), 4-arm PEG azide	Chondrocyte cells for *in vitro* study and *in vivo* study on Balb-c mice model	SPAAC	Cartilage tissue engineering	Hydrogel supported cell survival, and the cells regenerated cartilaginous tissue *in vivo*	(183)
Hydrogel composed of hyperbranched poly(ε-caprolactone) 32 functionalized with BCN	Bone (MC3T3 preosteoblast) cells	SPAAC	Bone tissue engineering	Excellent cytocompatibility toward bone cells; excellent support for cellular adhesion and proliferation on the hydrogel surface	(401)
Hydrogel composed of 4-arm DBCO-functionalized PEG, and a 4-arm PEG tetraazide, and a potent neurogenic differentiation factor (interferon-γ)	Neural stem cells	SPAAC	Neural tissue engineering	Excellent cytocompatibility toward neural stem cells; offers ability of immobilized attachment and signaling proteins to direct neural stem cells differentiation to neurons without need for additional supplementation	(439)
Injectable hydrogel composed of poly(propylene fumarate) functionalized with BCN-OH, and a hyper-branched 32-arm poly(ε-caprolactone) (PCL) dendrimer functionalized with azide	Bone (MC3T3 preosteoblast) cells for *in vitro* study and rabbit model for *in vivo* study	SPAAC	Bone tissue engineering	Hydrogel supported excellent proliferation and differentiation of preosteoblast cells on the surface; injectable system fill cavity in rabbit vertebral bodies similar to poly(methyl methacrylate) cement	(440)
Hydrogel of HA- tetrazine and HA-cyclooctene loaded with bone morphogenetic protein-2 (BMP-2) mimetic peptide (BP)	Human dental pulp stem cells (hDPSCs) *in vitro* and mice model for *in vivo* study	SPAAC	Bone tissue engineering	Hydrogel demonstrated excellent cytocompatibility *in vitro;* good biocompatibility *in vivo*; BP-loaded hydrogel induced osteogenic differentiation of hDPSCs *in vitro* and *in vivo*.	(441)
Hydrogel HA-cyclooctene and HA-azide	Mouse fibroblast cells (L929 cells) and human adipose-derived stromal cells (hASCs)	SPAAC	Not mentioned specifically	Hydrogel exhibited minimal swelling, long-term stability, good compatibility toward L929 and hACS cells. HA-based hydrogel also enhanced secretion of immunomodulatory factors by hASCs.	(442)
Interpenetrating (IPN) hydrogel composed of collagen, cross-linked via SPAAC, and HA cross-linked via thiol–ene		SPAAC, thiol–ene	Corneal tissue engineering	IPN hydrogel supported corneal epithelial cell growth on its surface. When applied to corneal stromal defects *in vivo*; IPN avoided epithelial hyperplasia, decreased stromal myofibroblast formation, and increased tight junction in regenerated epithelium	(443)
Hydrogel composed of PEG diacrylate, dithiothreitol, and borax	Endothelial cells and neural stem cells	Thiol–ene	Vascular tissue engineering and neural tissue engineering	Noncytotoxic toward endothelial cells and neural stem cells. Endothelial cells form capillary-like structures (vascular network); neural stem cells form neurosphere-like structures (neural development)	(405)
Hydrogel composed of allyl-modified chitosan and thiol-modified poly(NIPAM)	hMSCs for *in vitro* and mice model for *in vivo* study	Thiol–ene	Multidirectional tissue engineering	nontoxic toward hMSCs; subcutaneous tissue without signs of inflammation after 5 days of injection *in vivo*	(412)
Cell-instructive pectin hydrogels using cell-degradable peptide cross-linkers and integrin-specific adhesive ligands	Fibroblasts, keratinocytes	Thiol–ene	Skin tissue engineering	Tunable properties; capable of modulating behavior of embedded cells, including cell spreading, hydrogel contraction and secretion of matrix metalloproteases	(407)
Hydrogel composed of PEG dimaleimide, MMP-degradable dithiol containing peptide (KCGPQGIAGQCK), cell-adhesive peptide (CGRGDSGC) and norbonene-functionalized peptide (CGK(NB)GC)	Fibroblasts	Thiol–ene	Multidirectional tissue engineering	Tunable mechanical properties within tissue-like ranges, cytocompatibility of hydrogel, and ease of protein attachment make it useful for tissue engineering	(444)
Hydrogel made of norbornene-functionalized chitosan, thiol-functionalized PEG and collagen	Human dermal fibroblast (HDF) cells	Thiol–ene	Skin tissue engineering	Hydrogel pore size ∼20–100 μm; tunable rheological properties, that are favorable for cell adhesion and proliferation. Addition of small amount of collagen drastically improved cytocompatibility of hydrogel.	(445)
Seven different hydrogels made of alkene- or allyl-functionalized polymers and dithiolated PEG	Human umbilical vein endothelial cells (HUVECs)	Thiol–ene	Multipurpose tissue engineering application	Generally good mechanical and cell encapsulation properties show scope of thiol–ene-based hydrogels	(446)
Hydrogel made of acrylate, 3-dimethyl (chloropropyl) ammonium propanesulfonate-modified starch, thiol-modified PEG	A549 cells	Thiol–ene	Soft tissue engineering	Presence of zwitterionic moiety within hydrogel minimize nonspecific binding of proteins; cytocompatible with A549 cells	(447)
Hydrogel prepared of methacrylated HA and multithiol-functionalized zwitterionic CBMA polymer	hMSCs	Thiol–ene	Soft tissue engineering	hMSCs encapsulated within hydrogel remain metabolically active, indicating cytocompatibility. Cytokine secretion assays (IL-6 and TNF-α) support suppression of inflammatory reactions by hydrogel, indicating advantages for *in vivo* biomedical applications	(448)
Hydrogel made of placenta powder, thiol-modified PEG and bis(allyl propionic acid)-modified PEG	Human skin cells (HaCaT) and murine macrophage cells (Raw 264.7)	Thiol–ene	Soft tissue enginnering	Hydrogel exhibited good biocompatibility *in vitro* toward HaCaT and 264.7 cells, with cell viability above 91%.	(449)
Hydrogel of (allyl-terminated PEG)-functionalized polyhedral oligosiloxane and dithiol-functionalized PEG	HUVEC cells and human dermal fibroblast (HDF) cells	Thiol–ene	Soft tissue engineering	Tunable mechanical properties of hydrogel. Hydrogel exhibited good cytocompatibility toward HUVEC and HDF cells indicating potential for tissue engineering	(450)
Hydrogel of methacrylated HA and thiolated HA loaded with epidermal growth factor (EGF)	Mouse fibroblast cells (NIH3T3)	Thiol–ene	Skin tissue engineering	Hydrogel has high cytocompatibility toward NIH3T3 cells. Starch assay revealed good wound healing *in vitro*. Furthermore, in rat wound-healing model *in vivo*, the sustained release of EGF from hydrogel promotes appropriate tissue healing and restores it to normal state.	(451)
Hydrogel of furan-functionalized gelatin, bismaleimide-functionalized PEG and chitosan-*g*-pluronic	Cardiac progenitor cells (CPCs) for *in vitro* study and nude mice model for *in vivo* study	Diels–Alder	Cardiac tissue engineering	Outstanding load-bearing and shape recovery even under high compression. Subcutaneous administration of cardiomyocytes-laden f-IPN hydrogel into nude mice revealed high cell retention and survival after 2 weeks	(419)
Hydrogel of maleimide functionalized star-PEG, gelatin and methyl furfural-functionalized chitosan	Human glioma (U87-MG) cells	Diels–Alder	_	Good 3D printability and cell encapsulation capability without cytotoxicity	(452)
Hydrogel of norborene-functionalized hyaluronan and methyltetrazine-functionalized hyaluronan	Mice retinal (αRPE-19) cells	Diels–Alder	Retinal tissue engineering	Cytocompatible toward mice retinal cells with primary photoreceptors; enables multiphoton imaging of embedded retinal explants much longer (>38 h) than agarose thermogels	(219)
Hydrogel of adipic dihydrazide-modified sodium alginate, oxidized sodium alginate, bioglass and modified chondroitin sulfate	Cranial bone repair in a rat model *in vivo*	Diels–Alder and dynamic hydrazone cross-linking	Bone tissue engineering	Ecellent bone regeneration with generation of “white” new tissues; potentially applicable for bone tissue engineering	(453)
Hydrogel of HA-furan- adipic dihydrazide, HA-furan-aldehyde and PEG-dimaleimide	_	Diels–Alder and dynamic hydrazone formation	Cartilage tissue engineering	Good mechanical properties, tissue adhesiveness, self-healing behavior and pH responsiveness	(415)
Hydrogel composed of HA-aldehyde and HA-hydrazide	Mouse embryonic fibroblasts (NIH3T3) for *in vitro* study and Sprague–Dawley rats for *in vivo* study	Hydrazone	Skin tissue engineering, bone tissue engineering	Cytocompatible toward mouse fibroblast cells; capable of neo-bone formation with highly ordered collagen matrix-mimicking natural bone regeneration	(454)
Hydrogel made of PEG and carboxymethyl chitosan (PEG/CMC hydrogel)	COS-7 cells and HeLa cells for *in vitro* study, BALB/c mice for *in vivo* study	Amino-yne	Soft tissue engineering	Fast gel formation and good cell and tissue biocompatibility	(455)
Hydrogel made of aminooxy-terminated PEG, aldehyde-containing hyaluronic acid and collagen-1	Rat Schwann cells, hMSCs	Oxime	Soft tissue engineering	Noncytotoxic toward Schwann cells; incorporation of collagen-1 within hydrogel supported adhesion of hMSCs	(456)
Hydrogel of norbornene-functionalized gelatin and tetrazine-functionalized gelatin	EGFP 3T3 fibroblast cells and hMSCs for *in vitro* study, and mice model for *in vivo* study	IEDDA	Soft tissue engineering	Supported cell adhesion, proliferation *in vitro* without any cytotoxicity; hydrogel showed minimal inflammatory response in mice and sustained *in vivo* degradation after infiltration by host cells	(457)

## 4 Conclusions and Future Directions

In the last 15 years click reactions have emerged as novel and versatile platforms to develop functional polymeric materials for biomedical applications. The mild reaction conditions, outstanding coupling efficacy, and frequent bioorthogonality led to a wide variety of functional polymeric materials that are compatible with various bioactive moieties, including peptides, proteins, drugs, and genes, but also with cells and many other therapeutics. These versatile features enable applications of these biofunctional polymers as a functional extracellular matrix for tissue engineering, antiviral applications, and target-directed, controlled drug delivery, along with a stimuli-responsive behavior. In addition to that, these biofunctionalized polymers also offer an excellent platform for biosensing of biologically important molecules such as glucose, or any other biomarkers/proteins/genes related to any diseases and bioimaging as discussed in the above sections.

However, there are certain limitations that should be carefully considered before the development of any functional biomaterials using click reactions. Two cases may highlight this: the cytotoxic Cu catalyst in CuAAC is to be fully removed—which is very hard—for application in, e.g. tissue engineering, while for thiol-based hydrogels residual initiators can induce an immunogenic response at the cellular level. Any bioapplications would thus require thorough washing, to remove remaining traces of the catalyst or initiators. For instance, washing or dialysis against EDTA solutions is commonly used to remove even trace amounts of copper ions from CuAAC reaction mixtures and isolated products. However, this method often fails to achieve the desired level of purity.

A more effective strategy might involve immobilizing the catalyst on a support, which allows access to the reactants and can be easily separated from the reaction mixture upon completion. While there have been reports of heterogeneous CuAAC catalysts, this area is still under development.^458,459^ The same challenges apply to RuAAC catalysts as well. Other limitations concern the cost of some of the reagents (cyclooctyne derivatives for SPAAC applications) or the reversibility of certain reactions (e.g., thiol–ene). Such factors indicate that what is an overriding strength of one type of reaction (e.g., the biorthogonality of many strain-promoted click reactions) is not so relevant in another application, for which, e.g., spatial control by light is important, or might even be crucial in its functioning (e.g., the dynamic covalent bond character of certain clicked functional groups).

Another limitation to basically all reactions studied in this review is that until now almost all of the work reported has shown the beauty of click chemistry only in developing novel biomaterials with dimensions ranging from human cells to organs; more specifically these studies mostly focused on *in vitro* studies and proof of concept. There are only few studies that focused on *in vivo* biocompatibility,^460,461^ host response,^462,463^*in vivo* biodegradation,^464,465^ pharmacokinetics,^89,466^ and the fate of these functional polymeric materials^467^ after administration or implantation within the body. Therefore, a lot of research is still needed particularly *in vivo* to take these click-based polymeric materials from the lab to approved clinical applications. Specifically, the toxicity and immune response of such new click-based functional polymeric materials will then be crucial. Finally, an increasing number of studies point to the composite nature of an ideal material. Illustrative examples include the decoration of block copolymers with various different functionalities for the variety of functions that need to be fulfilled, or the advantageous effects of the mixing in of a small portion of collagen in synthetic hydrogels for optimal functioning. Here, both the further extension of synthetic capacibilities and the synergy with naturally occurring materials will likely prove crucial in the decades to come.

So, where do we see the biggest challenges and opportunities? For material studies and *in vitro* experiments, much more is feasible than for *in vivo* applications, so our focus will initially be on those. First, for large-scale *in vivo* applications, heavy metals have to go. So, despite the enormous drive provided by CuAAC to open up this field, metal-free click reactions are the way to go in this field. Here specifically the SPAAC, SPOCQ, IEDDA, and light-induced thiol–ene reaction provide options. The first three, as they are both fast yet uses bench-stable and nontoxic materials–this allows both the *ex vivo* preparation and *in vivo* application (as, e.g., done for the case of SPAAC or SPOCQ-based antibody–drug conjugates),^468,469^ or—in case a polymer needs to be formed within an organism after application—the *in vivo* formation (e.g., for the formation of wound-healing hydrogels). For such *in vivo* applications, a high reaction rate is likely crucial, but for *in vitro* studies this may be less important, and then expensive cyclooctynes can be replaced by much cheaper cyclopropenes, which react at least as efficiently in crowded environments, just slightly more slowly.^221^ The light-induced thiol–ene reaction can be used to rapidly form the polymer of interest at the location of interest, but here evidently also other, nonclick alternatives such as PET-RAFT^470^ may be used as well for the polymerization. When speaking about high reaction rates, of course, the initial applications of the up-to-now fastest class of click reactions, the TCO-tetrazine IEDDA reactions, in the field of PET imaging come to mind. Here the high reaction rate is simply essential and already impressive, but hopefully in the future this high reactivity can be combined with reagents with a slightly higher bench stability.

Second, the orthogonality of click reactions can be used to a higher degree, so as to allow the formation of more highly functionalized biopolymeric materials. This can illustrated with a reference to bispecific antibodies, which typically use one click reaction (e.g., IEDDA with cyclooctene reactants) for the coupling to one antibody, and another (e.g., SPOCQ) for the couplic to another.^471^ Such use of mild, fast, and yet highly efficient coupling reactions will open the door to multifunctional materials. In this regard one can, e.g., think about the efficient formation materials that are both antibacterial and antiviral, using different specifically active moieties clicked onto a generic backbone, or about the formation of materials with, e.g., antibacterial or antiviral action against a broader scope of such disease-causing entities. It is likely that this field develops further, as recent findings indicate that the barrier for many strain-promoted click reactions is predominantly entropic rather than enthalpic, and that differences between the various click reactions relate to differences in the activation entropy Δ*S*^#^.^227^ Further finetuning of the reactive strain-loaded moiety may thus be focus on reduction of the latter, with perhaps some reduction of the ring strain. The latter will likely lead to more (enthalpically) stable strained ring system, which will only increase the compatibility with other functionalities in the bioactive polymer, and thus further improve the orthogonality toward other moieties present.

Third, one can think of the use of click chemistries to couple tailor-made polymers onto biomolecules to either provide them with time-delayed and/or prolonged action. A point of reference here can be the classic paper of Keeffe and Jiang, which uses NHS-chemistry to couple zwitterionic polymers onto a bioactive protein,^472^ which significantly prolongs the activity of the protein. However, the NHS-chemistry used is not amino acid-residue specific, and the number of attached polymer chains is difficult to control given the high reactivity of multiple lysine residues. With click chemistries such as cysteine-directed thiol–ene^473^ or tyrosine-directed SPOCQ reactions^427^ or via the use of residue-specific modifications that allow the installation of, e.g., a cyclopropene, *trans*-cyclooctene or cyclooctyne, such improved control can be readily achieved. In addition, the attached polymer itself, such as a zwitterionic chain, can be dectorated itself again with directing moieties using click chemistry, as this is highly efficient and achievable under mild conditions.

Finally, potentially dynamic covalent click chemistries (thiol–ene, IEDDA, SuFEx/SuPhenEx) have the potential to efficiently form bioactive polymers, and yet combine this with a fine-tuned biologically induced decay. Inclusion of such polymers in the polymer backbone would allow for the controlled degradation of the entire polymer into urine-clearable fragments, while in pendent-functionalized polymers this may contribute to improved control over the delivery of the drug load. In both cases, of course, many other reactions display similarly characteristics, but given the fact that dynamic covalent click chemistries are still relatively young, we expect this field to further develop and flourish.

In summary, click-based functional polymeric materials display tremendous potential for real-life clinical applications given their ease of formation, controlled variability and ever-increasing functionalization. Such potential, as demonstrated in the reviewed papers, will hopefully provide the inspiration to future researchers to tuns this potential into practice, especially toward clinical diagnostics and treatment!

## Abbreviations

AA: 11-azido-3,6,9-trioxaundecan-1-amine
ATRP: atom transfer radical polymerization
BCN: bicyclo[6.1.0]non-4-yne
BCN-OH: (1*R*,8*S*,9*S*)-bicyclo[6.1.0]non-4-yn-9-ylmethanol
BPEG: benzoic-imine linkage
BSA: bovine serum albumin
Carb-N3: N_3_-functionalized cyclic carbonate monomer
CCS: core-cross-linked star
CLSM: confocal laser scanning microscopy
CNCs: cellulose nanocrystals
CNS: central nervous system
CPPA: 4-cyano-(phenylcarbonothioylthio) pentanoic acid
CS: chondroitin sulfate
CTA: chain transfer agent
CuAAC: copper-catalyzed alkyne–azide cycloaddition
DA: Diels–Alder reaction
DAA: direct-acting antiviral agents
DBCO: dibenzylcyclooctyne
DENV2: Dengue virus envelope protein 2
DHPA: 3,4-dihydroxyphenylacetic acid
DMA: *N*,*N*-dimethylacrylamide
dPG: dendritic polyglycerol
DTT: dithiothreitol
ECM: extracellular matrix
EGDMA: ethylene glycol dimethacrylate
EPR: enhanced permeability and retention effect
ER: estrogen receptor
ETFE: poly(ethylene-*alt*-tetrafluoroethylene)
EUV: extreme ultraviolet lithography technique
FACS: fluorescence-activated cell sorting facility.
FMMA: ferrocenylmethyl metharylate polymer
FRET: fluorescence resonance energy transfer
FRI: fluorescence reflectance imaging
G: gelatin
GSH: glutathione
GQDs: gelatin quantum dots
HA: hyaluronic acid
HBD: helix zone-binding domain.
HIV: human immunodeficiency virus
hMSCs: human mesenchymal stem cells
HPMAmLac1: *N*-2-hydroxypropyl methacrylamide monolactate
HPMAmLac2: *N*-2-hydroxypropyl methacrylamide dilactate
HSA: human serum albumin
HTLV-II: human T-lymphotropic virus types II
HUVEC: human umbilical vein endothelial cells
IEDDA: inverse-electron demand Diels–Alder reaction
IPN: interpenetrating network hydrogel
LBL: layer-by-layer assembly technique
LDI: l-lysine ethyl ester diisocyanate
LRP: living radical polymerization
MeOx-OTf: *N*-methyl-2-methyl-2-oxazolinium triflate
MES: 2-(*N*-morpholino)ethanesulfonic acid
MOB-Sox: 2[2-(4-methoxybenzylsulfanyl)ethyl]-2-oxazoline
MPC: 2-methacryloyloxyethyl phosphorylcholine
MPU: multifunctional polyurethane
MRI: magnetic resonance imaging
MSCs: mesenchymal stem cells
N3-PEG-N3: poly(ethylene glycol)-diazide
NaCp: sodium cyclopentadienide
NIPAM: *N*-isopropylacrylamide
NMP: nitroxide-mediated polymerization.
OTO: 1,4-oxathiepan-7-one
P(EAEP-PPA): poly(phosphoester-*co*-propynylamine)
P(TMCC-*co*-LA): poly(2-methyl-2 carboxytrimethylene carbonate-*co*-D, l-lactide)
PA: propionic acid
PAG-*b*-PFMA: poly[2-(acrylamido) glucopyranose]-*b*-poly(furfuryl methacrylate)
PAMIs: poly(aminomaleimide)s
PCL: poly(ε-caprolactone)
PCL-propargyl: propargyl poly(-caprolactone)
PDA: polydiacetylene
PEDOT: poly(3,4-ethylenedioxy-thiophene)
PEEP: poly(ethyl ethylene phosphate)
PEG: poly(ethylene glycol)
PEG-*b*-P(DPA-*co*-FMA: poly(diisopropylaminoethyl methacrylate-*co*-furfuryl methacrylate)
PEG-*co*-PPG: α,ω-diazido poly[(ethylene glycol)-*co*-(propylene glycol)]
PEGMA-AGE-N3- azide-modified: (PEG-methacrylate)-*co*-poly(allyl glycidyl ether)
PEGOL-60: polyethylene glycol-based dendrimer
PET: positron emission tomography
PGMA: poly(glycidyl methacrylate)
PGN: polyglycidyl nitrate
PhA: *N*-phenyl-acrylamide
PHEMA: poly(2-hydroxyethyl methacrylate)
PhE-OE: 10-cetyl-10H-phenothiazine-3,7-(4,4′-aminophenyl)acetonitrile
PI-CMRDRP: photoinduced copper mediated reversible deactivation radical polymerization
PmBA-(PCL)2: poly(α-methyl β-alanine)-poly(ε-caprolactone)
p-PgMAMPC: poly[(propargyl methacrylate)-*ran*-(2-methacryloyloxyethyl phosphoryl-choline)]
PPLG: poly(g-propargyl-l-glutamate)
PU–PEG: polyurethane-poly(ethylene glycol) hydrogels
PVA-HA: poly(vinyl alcohol)-hyaluronic acid microgel
RAFT: reversible addition–fragmentation chain transfer polymerization
RAFT-SVCP: RAFT-self-condensing vinyl polymerization
RGD: Arginine-Glycine-Aspartate
ROMP: ring-opening metathesis polymerization
SA: streptavidin
SET-LRP: single-electron transfer living radical polymerization
SPAAC: strain-promoted alkyne–azide cycloaddition
SPOCQ: strain-promoted oxidation-controlled cyclooctyne-1,2-quinone cycloaddition
SPR: surface plasmon resonance
SS-Az: reduction-cleavable cross-linker
SuFEx: sulfur(VI) fluoride exchange click reaction
TAD: 1,2,4-triazoline-3,5-dione
TMC: trimethylene carbonate
TMS-N3/TBAF: azido-trimethyl silane-tetrabutylammonium fluoride
TPOM: tetrakis(2-propynyloxymethyl) methane
VS: vinyl sulfones
α-CD: α-cyclodextrin
